# Radiochemical Synthesis of Alkyl Geminal ^18^F-Difluoroalkyl Motifs Mediated by Silver(I) Oxide

**DOI:** 10.1021/acs.orglett.5c00187

**Published:** 2025-02-06

**Authors:** Pawan Mishra, Jasmine Hind, Ian A. Fallis, Matthew Tredwell

**Affiliations:** † School of Chemistry, Cardiff University, Main Building, Park Place, Cardiff CF10 3AT, U.K.; ¶ Wales Research and Diagnostic PET Imaging Centre, Cardiff University, University Hospital of Wales, Heath Park, Cardiff CF14 4XN, U.K.

## Abstract

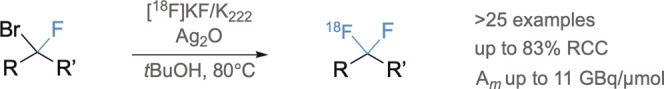

^18^F radiotracers are used in positron emission tomography imaging for medical diagnosis and drug development. Current methods to synthesize ^18^F-polyfluorinated functional groups are limited in substrate scope and result in radiotracers with low molar activities (*A*_*m*_). We disclose the synthesis of a range of ^18^F-difluoroalkyl groups by nucleophilic substitution of geminal bromofluoroalkyl electrophiles with [^18^F]fluoride mediated by Ag_2_O. The utility of this transformation to support (pre)clinical imaging is demonstrated by translation to an automated synthesizer.

Fluorine-18 (^18^F) is a radionuclide that is routinely used to label small molecules for positron emission tomography (PET) imaging applications in medical diagnosis and drug development (half-life *t*_1/2_ = 109.8 min; positron energy Eβmax = 0.63 MeV).^1^ To date, a majority of the ^18^F radiotracers used for (pre)clinical studies have been synthesized via nucleophilic displacement reactions of activated secondary and primary alkyl electrophiles with [^18^F]fluoride to give the corresponding alkyl ^18^F-fluorides.^2^ Other fluorinated motifs such as the geminal difluoroalkyl group, which are commonly found in pharmaceuticals, are rarely utilized as a site for radiolabeling with ^18^F. While significant progress has been made on the radiochemical synthesis of ^18^F-trifluoromethyl groups,^3^ the synthesis of ^18^F-difluoroalkyl groups is less well developed with most methods directed at ^18^F-difluoromethylarenes.^4^ Given the ubiquitous presence of non-aryl geminal difluoroalkyl motifs in pharmaceuticals, and the ability of the CF_2_ group to favorably modify physiochemical properties,^5^ a general method to create the ^18^F-CF_2_ functional group would be advantageous for radiotracer development (Figure 1).

**Figure 1:**
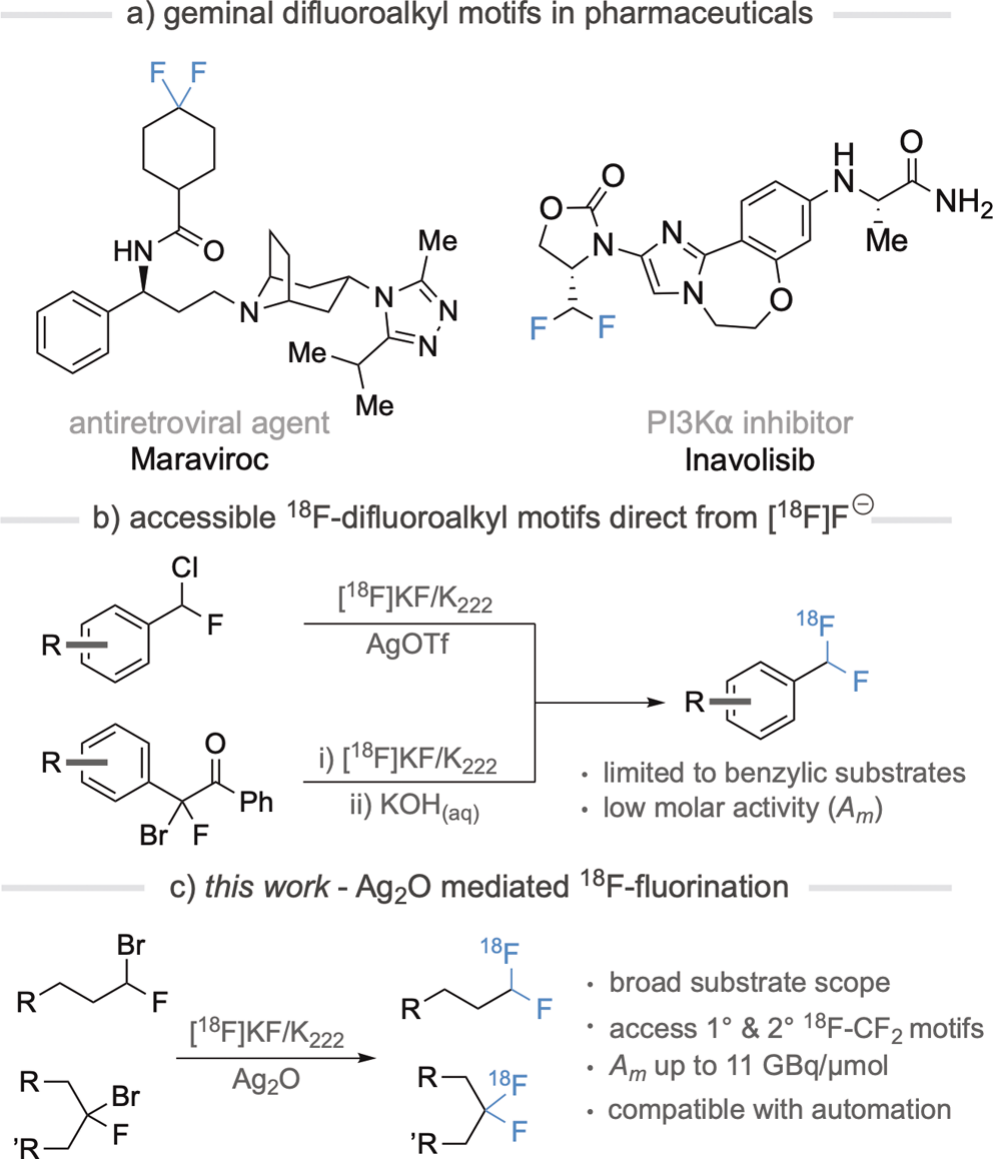
(a) Examples of Food and Drug Administration-approved pharmaceuticals containing the CF_2_ motif. (b) Currently accessible ^18^F-difluoroalkyls from [^18^F]fluoride. (C) Ag_2_O-mediated nucleophilic substitution (this work).

Herein, we describe a silver(I) oxide-mediated ^18^F fluorination of alkyl bromofluoroalkanes to give access to a broad range of geminal ^18^F-difluoroalkyl functionalities. We show the utility of this method to support (pre)clinical studies with the translation of this methodology onto a commercial automated radiochemical synthesizer. To date, examples of nucleophilic ^18^F fluorination of α-fluoroalkyl electrophiles have been limited to activated systems in which competitive E_2_ elimination is prevented by the lack of a β-hydrogen (Figure 1b).^6^ These transformations typically require high temperatures or the use of additives to promote product formation.^4,7,8^ To develop general ^18^F polyfluorination methods, radiochemists have circumvented this challenging C–^18^F bond-forming step and developed ^18^F analogues of known trifluoromethylation and difluoromethylation agents with a good scope by forming C–C^18^F_*n*_ bonds.^3,4b,4d^ One drawback to this ^18^F reagent-based approach is the additional time and complexity needed to first synthesize the radiolabeled reagent before it can be used for radiotracer synthesis, negatively impacting the radiochemical yield (RCY) and molar activity (*A*_*m*_).

Reported methods toward geminal ^18^F-difluoroalkyl motifs are scarce. Haufe and co-workers reported an oxidative desulfurization process of a single aryl ether substrate in the presence of carrier-added pyridine·[^18^F]HF on a single substrate in low RCY and radiochemical purity (RCP).^9^ The reduction of a 1,1-^18^F-difluoroalkene to the corresponding ^18^F-difluoroalkane was also shown to be possible by Frost *et al.*(10) Recently, an oxidative decarboxylation approach that allowed access these motifs from α-fluorocarboxylic acids using a manganese catalyst and PhIO was reported.^11^ While this latter report is the first method to synthesize a range of motifs, we envisaged that a direct, single-step, nucleophilic substitution of a fluorinated electrophile by [^18^F]fluoride would offer significant advantages for the radiotracer development and clinical translation. As highlighted, the key challenge with this approach is how to activate the system toward nucleophilic substitution in light of facile elimination.^6^ To this end, we initiated a study to investigate the use of activating agents that would promote nucleophilic substitution of α,α-fluorohaloalkyl electrophiles with [^18^F]fluoride, mindful of the unique reaction conditions under which radiochemists work (nanomoles to picomoles of [^18^F]fluoride) and the contrasting reactivity often observed between ^18^F and ^19^F transformations.^12^ Realization of this goal would allow access to a broad range of geminal ^18^F-difluoroalkyl groups and expand the chemical space for the development of new PET imaging agents.

Our initial experiments began using (3-bromo-3-fluoropropyl)benzene (**1a**, 0.04 mmol) with aliquots of azeotropically dried [^18^F]KF/K_222_ (20–30 MBq). Thermal activation, or the use of *t*BuOH that has been shown to enhance the nucleophilicity of fluoride,^13^ was found to be ineffective in promoting the desired substitution reaction (Table 1 entry 1 or 2, respectively).^14^ We tested a range of silver salts that were shown to be successful in promoting ^18^F fluorination in other halogen exchange reactions. However, the homogeneous Ag(I) sources AgOTf, [^18^F]AgF,^15^ AgBF_4_, and AgPF_6_ (entries 3–6, respectively) were all ineffective. Pleasingly, heterogeneous Ag_2_O (0.04 mmol, 9 mg) in dichloroethane (DCE) at 80 °C gave the desired product [^18^F]**2a** in 25% RCC (entry 7). From this initial hit, we screened a range of solvents, with acetonitrile giving [^18^F]**2a** in 35% RCC (entry 8), while *t*BuOH gave an improved RCC of 68% (entry 9). Decreasing the quantity of Ag_2_O (0.02 mmol, 5 mg) resulted in a minor decrease in the level of conversion (entry 10), while increasing the solvent volume from 150 to 300 μL resulted in a slight decrease in RCC (entry 11). This was an encouraging result as larger volumes are needed for subsequent automated syntheses. Changing the bromide leaving group to chloride resulted in no reaction under the optimal conditions, while the corresponding iodide gave an improved RCC of 83%. Despite this higher level of conversion for the fluoroiodo alkyl electrophiles, further studies described herein focus on the bromofluoro substrates due to their higher stability, synthetic accessibility, and ease of handling.

**Table 1:**
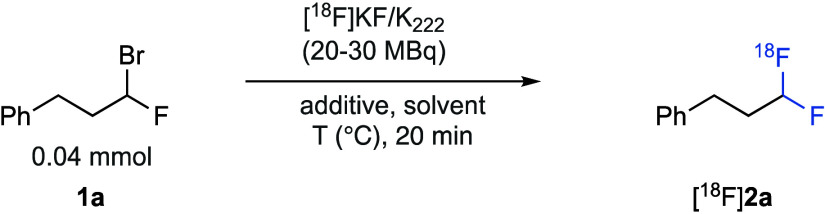
Optimization of the Reaction Conditions

entry	solvent^a^	additive (mg)	temp (°C)	RCC (%)^b^
1	MeCN	none	100	<1
2	*t*BuOH	none	100	<1
3	DCE	AgOTf (10)	80	0
4	MeCN	[^18^F]AgF	90	<1
5	DCE	AgBF_4_ (8)	80	0
6	DCE	AgPF_6_ (10)	80	0
7	DCE	Ag_2_O (9)	80	25 ± 7
8	MeCN	Ag_2_O (9)	80	35 ± 11
9	*t*BuOH	Ag_2_O (9)	80	68 ± 12
10	*t*BuOH	Ag_2_O (5)	80	55 ± 14
11^c^	*t*BuOH	Ag_2_O (5)	80	47 ± 1

a Volume of 150 μL.

b *n* = 3.

c Volume of 300 μL.

Acetonitrile is commonly employed as a solvent in clinical radiotracer production, so it is noteworthy that for a majority of the substrates tested, the use of Ag_2_O (9 mg) in MeCN at 80 °C gave the desired ^18^F products in RCCs sufficient to support (pre)clinical imaging studies (entry 8). However, given the yield improvements observed with *t*BuOH, we investigated the scope of this transformation and its generality and tolerance to functional groups commonly found in small molecule pharmaceuticals and radiotracers under the conditions described in entry 11 and the examples shown in Scheme 1. A series of primary carbon electrophile precursors were synthesized bearing alkyl, aryl, bromo, chloro, fluoro, and iodo substituents, which gave the corresponding radiolabeled products ([^18^F]**2b**–[^18^F]**2h**) in RCCs comparable to that of parent compound [^18^F]**2a**. The presence of ketone and ester functionalities ([^18^F]**2i** and [^18^F]**2j**, respectively) were well tolerated, in contrast to the carboxylic acid ([^18^F]**2o**) that was esterified under the reaction conditions. Variation in the length in the alkyl chain had a limited effect on RCCs with [^18^F]**2k**–[^18^F]**2n** being produced in useful quantities. Pleasingly, nitrogen-containing and heterocyclic structures ([^18^F]**2p**–[^18^F]**2s**) were successfully fluorinated, demonstrating the applicability of this method to more structurally complex molecules. In addition to producing radiolabeled primary [^18^F]CF_2_H motifs, the reaction was also successful on secondary substrates with ketones [^18^F]**2t** and [^18^F]**2u** formed in 9 ± 3% and 22 ± 6% RCCs, respectively. The non-activated secondary alkyl CF_2_ motifs commonly found in medicinal chemistry programs ([^18^F]**2v**–[^18^F]**2x**) were also successfully radiolabeled with ^18^F, albeit with an efficiency lower than that of the primary electrophiles. The transformation was successful on an activated benzylic substrate giving [^18^F]**2y** in 83 ± 3% RCC (*n* = 3), a significant increase compared to that of the previously reported silver triflate-mediated method.^4h^ Extension of the methodology to the less reactive difluorobromo electrophiles was unsuccessful, with no trace of [^18^F]**2z** under the optimal conditions.

**Scheme 1:**
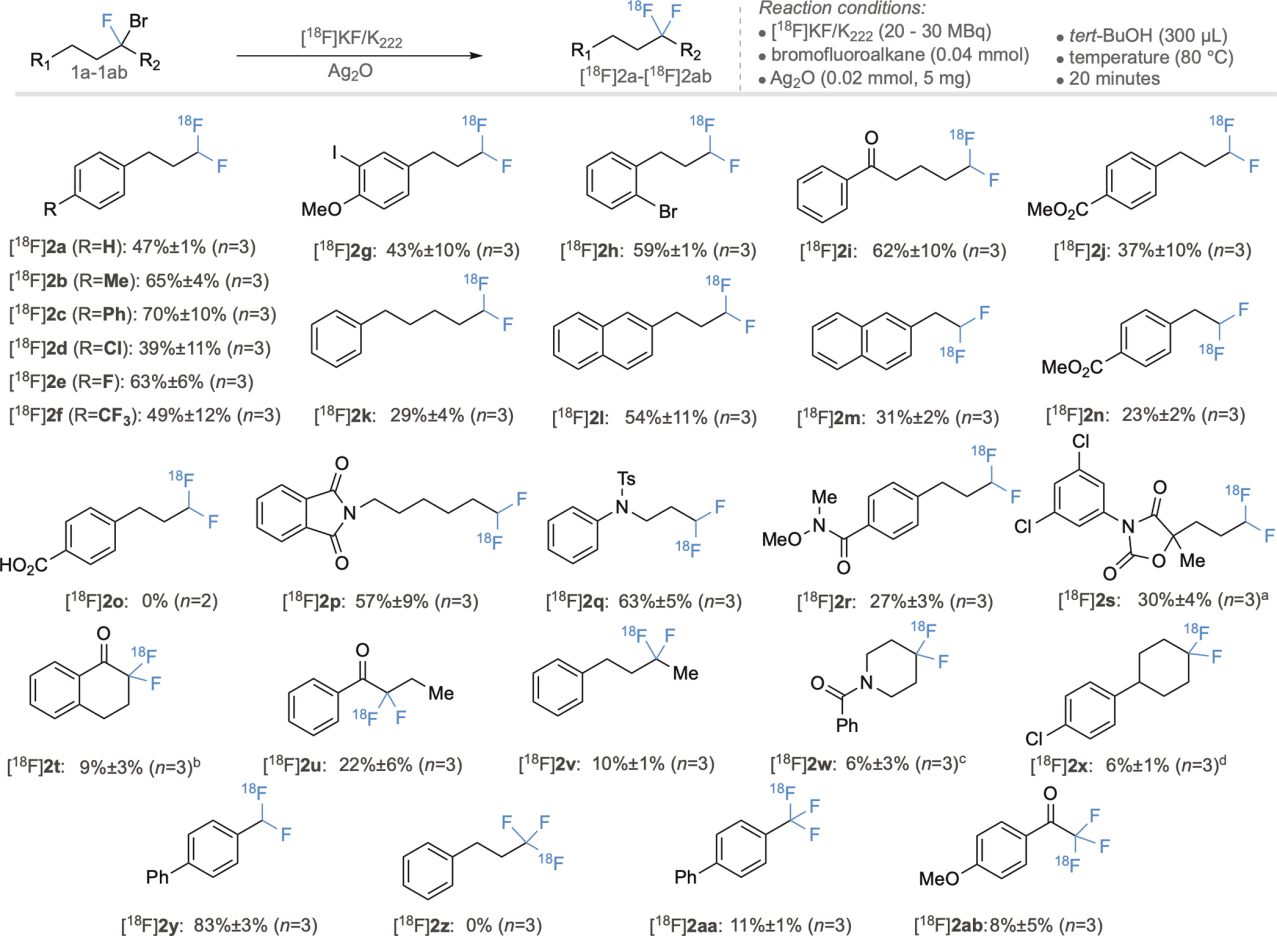
Substrate Scope of Ag_2_O-Mediated ^18^F Fluorination The starting material was a 1:1 mixture of diastereoisomers. With MeCN as the solvent. The reaction was performed for 40 min at 120 °C. The reaction time was 40 min, and the starting material was a 3:2 mixture of diastereoisomers. Yields are RCCs.

However, the presence of an arene, or carbonyl group, proximal to the reactive site was sufficiently activating to allow the formation of [^18^F]**2aa** or [^18^F]**2ab** in 11 ± 1% or 8 ± 5% RCC, respectively (*n* = 3). These results further demonstrate the utility of this method for the synthesis of ^18^F-polyfluorinated functionalities for radiotracer development.

On the basis of these results and the apparent enhanced reactivity of these fluorinated electrophiles toward [^18^F]fluoride, in the presence of Ag_2_O, we were curious about whether a similar effect would be observed for the parent alkyl bromides.^16^ While the nucleophilic displacement of primary alkyl electrophiles with [^18^F]fluoride is well-established,^17^ there are relatively few examples of non-activated secondary electrophiles, despite the prevalence of 2-[^18^F]fluoro-2-deoxyglucose in clinical imaging.^18^ The secondary bromide (**4a**) was treated with [^18^F]KF/K_222_ (20–30 MBq) in *t*BuOH at 80 °C for 20 min, resulting in a RCC of 3 ± 1% (*n* = 3). This result highlights that access to secondary ^18^F-alkyl fluorides from the corresponding bromides can be nontrivial under typical ^18^F radiolabeling conditions. Repeating this reaction in the presence of 9 mg of Ag_2_O gave the desired compound [^18^F]**5a** in 16 ± 3% RCC (*n* = 3) (Scheme 2). This enhancement in RCC suggests that this Ag_2_O-mediated ^18^F transformation may have an application more generally in the synthesis of ^18^F-alkyl fluorides for PET radiotracer synthesis.

**Scheme 2:**
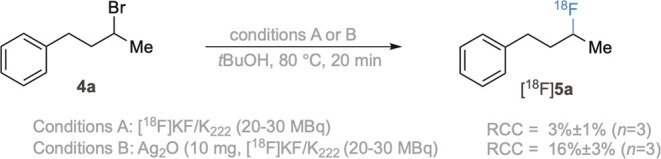
^18^F Fluorination of Secondary Alkyl Bromide

Automation of a PET radiosynthesis protocol is crucial to the development of the ^18^F methodology to support (pre)clinical imaging studies. Automated synthesizers increase reproducibility while decreasing the radiation dose to users and aid in validation.^19^ We adapted our optimized reaction conditions for use on a Trasis All-In-One synthesizer for fully automated radiosynthesis. For the automated synthesis, the quantities of the precursor (0.04 mmol) and Ag_2_O (5 mg) were kept the same as under the previously described conditions, as were the reaction temperature and time (Scheme 3). The reaction volume was increased to 500 μL; notably, despite the absence of stirring on the automated synthesizer, the reactions afforded RCCs similar to those of manual reactions. Starting with 18.1 GBq of [^18^F]fluoride afforded [^18^F]**2e** after HPLC purification in an activity yield of 4.8 GBq (27% RCY, non-decay-corrected). The total synthesis and purification time was 65 min. The molar activity (*A*_*m*_) of [^18^F]**2e** was calculated to be 8.1 GBq/μmol. Increasing the starting radioactivity to 43.5 GBq resulted in an activity yield of 11.6 GBq (27% RCY, non-decay-corrected) and an increased *A*_*m*_ of 11.2 GBq/μmol. We also performed an automated synthesis of [^18^F]**2u**, starting with 49.3 GBq of [^18^F]fluoride. We were able to isolate 3.3 GBq (7% RCY) with an *A*_*m*_ of 2.4 GBq/μmol.

**Scheme 3:**
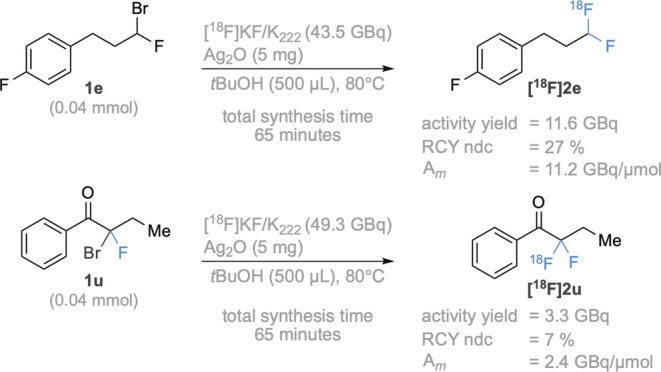
Scale-up and Automation of ^18^F Transformation

These values are consistent with other reactions toward ^18^F-polyfluorinated substrates in which the precursor acts as a source of ^19^F. These reproducible results validate the observed RCCs under our manual conditions and demonstrate that the use of our heterogeneous silver catalyst allows for the isolation of multipatient doses of the radiotracer.^8b^ We tested the purified radiotracers for the presence of residual silver using ICP-MS (4 ppm), demonstrating that residual silver can be effectively removed. These results demonstrate the potential of this method to support clinical imaging studies.

In conclusion, we have developed a silver-mediated synthesis of geminal ^18^F-difluorinated and ^18^F-trifluorinated motifs of fluorobromo electrophiles. This general method allows access to a wide range of substrates by nucleophilic substitution with [^18^F]fluoride. Commonly employed silver salts were found to be ineffective, with only Ag_2_O providing the desired geminal ^18^F-difluorinated compounds. This transformation was also found to be effective in promoting the synthesis of a secondary ^18^F-alkyl fluoride. The practical simplicity of the method facilitated the translation to an automated radiochemical synthesizer, highlighting the potential of this ^18^F radiochemical method for radiotracer development in PET radiopharmacies, with the isolation of ^18^F compounds in activity yields and radiochemical purities sufficient to support (pre)clinical imaging studies.

## Data Availability

The data underlying this study are available in the Cardiff University data catalogue at: 10.17035/cardiff.28343480 and the Supporting Information.

## Supporting Information Available

Preparation of labeling precursors and reference compounds, synthetic methods, radiochemistry methods, (radio)HPLC data, and NMR spectra (PDF)
