# Supplementary material for: Radiochemical Synthesis of Alkyl Geminal 18F-Difluoroalkyl Motifs Mediated by Silver(I) Oxide
Source: Org Lett. 2025 Feb 6;27(7):1724–8. doi: 10.1021/acs.orglett.5c00187 (PMC11852199; doi:10.1021/acs.orglett.5c00187)

Supporting Information

Radiochemical synthesis of alkyl geminal ^{18}F-difluoroalkyl motifs mediated by silver(I) oxide

Pawan Mishra,[a] Jasmine Hind,[a] Ian A. Fallis,[a] Matthew Tredwell[a,b,]*

[a] School of Chemistry, Cardiff University, Main Building, Park Place, Cardiff, CF10 3AT, U.K.

[b] Wales Research and Diagnostic PET Imaging Centre, Cardiff University, University Hospital of Wales, Heath Park, Cardiff, CF14 4XN, U.K.

Email: tredwellm@cardiff.ac.uk

**Contents**	**Page No.**
**General Information**	S2 - S3
**Preparation of starting materials and references**	S4
Preparation of labelling precursors	S4 - S19
Synthesis of 1,1-[^{19}F]difluoroalkane references	S20 – S31
**Radiochemistry**	S32
General information	S32
HPLC conditions	S32-S33
Optimisation reactions	S33
Manual radiofluorination of 1-bromo-1-fluoroalkane substrates	S34
Automated radiosynthesis	S35 – S42
RadioHPLC overlays and radiochemical conversions	S43 - S69
**References**	S70
**NMR spectra**	S71 - S143

General Experimental

General information: ^{1}H, ^{13}C and ^{19}F NMR spectra were obtained on either a Bruker Avance 300 (300 MHz ^{1}H, 75 MHz ^{13}C), Bruker Avance 400 (400 MHz ^{1}H, 101 MHz ^{13}C, 376 MHz ^{19}F) or a Bruker Avance 500 (500 MHz ^{1}H, 126 MHz ^{13}C, 471 MHz ^{19}F) spectrometer at rt in the solvent stated. Chemical shifts for protons and carbons are reported in ppm relative to the residual solvent signal. Data has been reported as follows: chemical shift, multiplicity (s = singlet, brs = broad singlet, d = doublet, t = triplet, q = quartet, m = multiplet, dd = doublet of doublet, dt= doublet of triplet, ddd = doublet of doublets of doublets), coupling constants in Hertz and integration. High resolution mass spectrometry (HRMS, m/z) data was acquired at Cardiff University. ES/EI/CI HRMS data was collected on a Thermo Scientific Exactive GC machine with an orbitrap mass analyser or a Waters Xevo G2XS. ICP-MS data was collected on an Agilent 7900 machine. TLC analysis was performed on commercially prepared 60 F_{254} silica gel plates and visualized by ultraviolet light (254 nm), followed by staining with 1% aqueous $KMnO_4$ solution. Preparative TLC Flash chromatography used silica gel 60 (230-400 mesh) in the solvent system stated. IUPAC names were obtained using the ChemDraw service. Weighing was performed with a 4 or 5 decimal place balance.

All reagents were used directly as obtained commercially unless otherwise noted. Silver(I) oxide was purchased and used directly (Fluorochem, Product code F044724, purity >99%), anhydrous forms of dimethylformamide (DMF), dimethylsulfoxide (DMSO), dichloroethane (DCE), tetrahydrofuran (THF) and dichloromethane (DCM), *t*-butanol, acetonitrile and methanol were purchased from Acros Organics. Alkyl bromides, tetraethylammonium bromide, diethylaminosulfur trifluoride (DAST), dimethyl 2-fluoromalonate were purchased from Fluorochem and Apollo Scientific. 3-Diiodo-5,5-dimethylhydantoin (DIH) and blue LED (Aldrich® Micro Photochemical Reactor blue LED (ALDKIT001) photoreactor were purchased from Merck. Unless otherwise stated, all glassware was dried in a 125 °C oven before use and all reactions were performed under an atmosphere of nitrogen.

Photochemical Reactor Aldrich® Micro Photochemical Reactor, blue LED lights (ALDKIT001- 1EA). LED light is IP68 double density 12vdc waterproof blue light with spectral range of 435-445 nm with wall plug power supply 500 mA with 5-6 watts. The irradiation vessel material is borosilicate glass. The distance of irradiation vessel from light source is 5cm and all glassware was borosilicate. There is no use of any kind of filters.

For manual radiochemistry experiments: [^{18}F]Fluoride was produced in an IBA Cyclon 18/9 cyclotron using by the $^{18}O(p,n)^{18}F$ reaction and delivered as [^{18}F]fluoride in [^{18}O]water.

Radiosynthesis and azeotropic drying were performed on an AllinOne radiosynthesizer (TRASIS).

For automated radiochemistry experiments: [^{18}F]Fluoride was produced in an IBA Cyclon 18/9 cyclotron using by the ^{18}O(p,n)^{18}F reaction and delivered as [^{18}F]fluoride in [^{18}O]water. All experiments were performed on an AllinOne radiosynthesizer (Trasis, Belgium). All isolated activity yields are non-decay corrected (n.d.c.). All molar activities are decay corrected to the end of synthesis (EOS), unless stated otherwise.

Preparation of starting materials and references

Preparation of labelling precursors

General Procedure A: Synthesis of 1,1-bromofluoroalkanes: The synthesis of these compounds followed a modified reported procedure[1].

First step:

Synthesis of α-fluorocarboxylic acid:

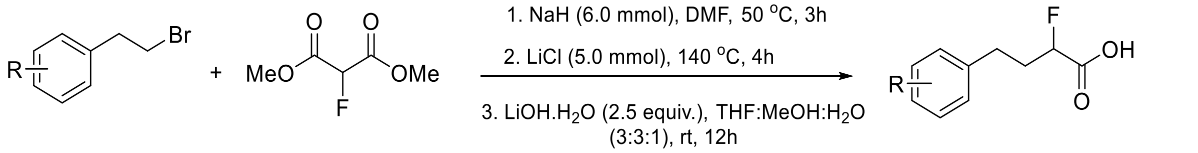

60-75% (*over three steps*)

A Schlenk tube was purged with NaH (60% dispersion in mineral oil) (240 mg, 6.0 mmol, 60% in mineral oil) and *N,N*-dimethylformamide (10 mL). The mixture was cooled to 0 °C and dimethyl 2-fluoromalonate (6.0 mmol) was added dropwise. After stirring for 20 minutes, the bromide (5.0 mmol) was added. The mixture was then heated to 50 °C for 3 hours or until complete consumption of the alkyl bromide by TLC analysis.

Lithium chloride (5.0 mmol) was then added to the reaction mixture and the mixture heated using heating mantle at 140 °C for 4 hours. The reaction was quenched with the addition of saturated $NH_4Cl_{(aq)}$ solution (10 mL) and diluted with ethyl acetate (30 mL). The organic phase was washed with H_2O (3 x 20 mL), saturated brine (20 mL) and dried over anhydrous $MgSO_4$, filtered, and concentrated in vacuo to give the crude. The crude was directly subjected to the next step without purification.

To a solution of the crude methyl-ester in 3:3:1 mixture of THF:MeOH:H_2O [0.36 M], lithium hydroxide (monohydrate) (12.5 mmol, 2.5 equiv.) was added in one portion and stirred overnight at room temperature. After full conversion, the reaction mixture was concentrated, diluted with H_2O (20 mL), acidified to pH 1 with 1M $HCl_{(aq)}$ and extracted with ethyl acetate (3 × 50 mL). The combined organic phases were washed with saturated brine (20 mL), dried over anhydrous $MgSO_4$, filtered and concentrated under reduced pressure. The residue was purified by flash column chromatography using (2:5 ethyl acetate/petroleum ether (40-60 °C) or hexane) to afford the corresponding carboxylic acid.

Second Step: Synthesis of 1,1-bromofluoroalkane:

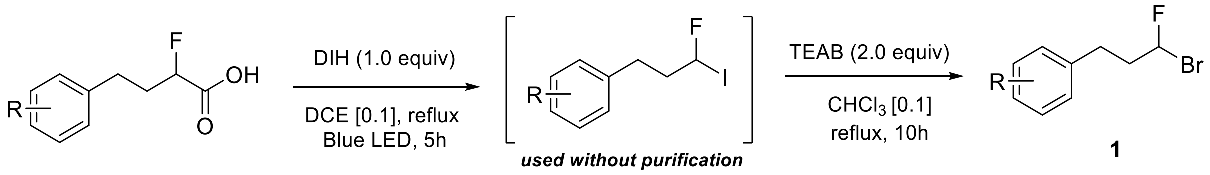

A mixture of 2-fluorocarboxylic acid (2.0 mmol), 1,3-diiodo-5,5-dimethyl hydantoin (DIH) (2.0 mmol) were dissolved in 1,2-dichloroethane (10 mL). The mixture was purged with nitrogen, heated on an oil bath under reflux, and irradiated with blue visible light from a distance of 5 cm for 5 h (Aldrich® Micro Photochemical Reactor blue LED (ALDKIT001) was used as the blue LED light source). The reaction mixture was cooled to rt and quenched with 1 M $Na_2SO_{3(aq)}$ (20 mL). The organic layer was washed further with $Na_2SO_{3(aq)}$ (2 x 20 mL), dried over anhydrous $MgSO_4$, filtered and concentrated under reduced pressure. The crude was directly subjected to the next step without purification.

The crude iodide and tetraethylammonium bromide (TEAB) (2.0 mmol) in chloroform (10 mL) were refluxed on heating mantle for 10 h. After complete conversion, the reaction mixture was cooled to room temperature and quenched with 1 M $Na_2SO_{3(aq)}$ (10 mL). The mixture was diluted with DCM (20 mL) and the organic layer was washed with Na_2SO_3 (1 x 20 mL), dried over anhydrous $MgSO_4$, filtered and concentrated under reduced pressure. The crude was purified by flash column chromatography using (02:98 ethyl acetate/petroleum ether (40-60 °C) or hexane) to afford the corresponding 1,1-bromofluoroalkane.

4-([1,1'-Biphenyl]-4-yl)-2-fluorobutanoic acid (A1)

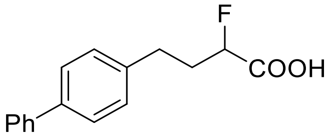

The title compound **A1** was prepared following the synthetic method (step 1) of general procedure **A**. Purification by column chromatography using (4:6 ethyl acetate/petroleum ether (40-60 °C)) as eluent, yellow gummy liquid, 840 mg, 65% yield; **^{1}H NMR** (400 MHz, $CDCl_3$) δ 10.65 (brs, 1H), 7.63 – 7.53 (m, 4H), 7.47 – 7.43 (m, 2H), 7.37 – 7.33 (m, 1H), 7.31 – 7.29 (m, 2H), 5.13 – 4.82 (m, 1H), 2.93 – 2.85 (m, 2H), 2.36 – 2.24 (m, 2H). **^{13}C NMR** (101 MHz, $CDCl_3$) δ 175.9 (d, J = 24.1 Hz), 141.0, 139.6, 138.9, 129.2, 128.9, 127.5, 127.3, 127.1, 87.4 (d, J = 185.5 Hz), 33.8 (d, J = 20.8 Hz), 30.2 (d, J = 3.1 Hz); **^{19}F{^{1}H}**

NMR (376 MHz, $CDCl_3$) δ -193.80 (s). **HRMS** [ESN]: *m/z* $[M-H]^-$ cal. $C_{16}H_{14}O_2F$, 257.0982, observed 257.0978.

2-Fluoro-4-(4-fluorophenyl)butanoic acid (A2)

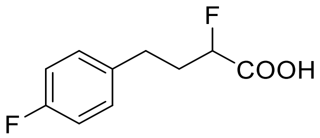

The title compound **A2** was prepared following the synthetic method (step 1) of general procedure **A**. Purification by column chromatography using (4:6 ethyl acetate/petroleum ether (40-60 °C)) as eluent, yellow gummy liquid, 710 mg, 70% yield; **^{1}H NMR** (400 MHz, $CDCl_3$) δ 11.02 (s, 1H), 7.19 – 7.15 (m, 2H), 7.02 – 6.96 (m, 2H), 5.00 – 4.85 (m, 1H), 2.85 – 2.76 (m, 2H), 2.28 – 2.17 (m, 2H); **^{13}C NMR** (101 MHz, $CDCl_3$) δ 175.9 (d, *J* = 24.1 Hz), 161.7 (d, *J* = 244.3 Hz), 135.5 (d, *J* = 3.2 Hz), 130.1 (d, *J* = 7.9 Hz), 115.5 (d, *J* = 21.2 Hz), 87.3 (d, *J* = 185.3 Hz), 33.9 (d, *J* = 20.8 Hz), 29.7 (d, *J* = 3.1 Hz); **^{19}F{^{1}H} NMR** (376 MHz, $CDCl_3$) δ -116.71 (s), -193.85 (s). **HRMS** (ESN): *m/z* $[M-H]^-$ cal. $C_{10}H_9O_2F_2$, 199.0577, observed 199.0571.

2-Fluoro-4-(4-(trifluoromethyl)phenyl)butanoic acid (A3)

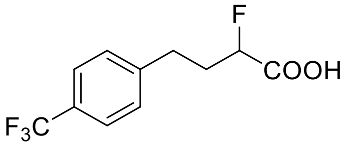

The title compound **A3** was prepared following the synthetic method (step 1) of general procedure **A**. Purification by column chromatography using (4:6 ethyl acetate/petroleum ether (40-60 °C)) as eluent, yellow gummy liquid, 930 mg, 74% yield; **^{1}H NMR** (400 MHz, $CDCl_3$) δ 10.69 (s, 1H), 7.57 (d, *J* = 8.1 Hz, 2H), 7.31 (d, *J* = 8.1 Hz, 2H), 4.94 (dt, *J* = 12.1, 5.8 Hz, 1H), 2.93 – 2.88 (m, 2H), 2.32 – 2.22 (m, 2H). **^{13}C NMR** (101 MHz, $CDCl_3$) δ 175.8 (d, *J* = 24.1 Hz), 144.0 (app.d, *J* = 1.2 Hz), 129.0, 129.0 (q, *J* = 32.4 Hz), 125.7 (q, *J* = 3.8 Hz), 124.3 (q, *J* = 271.9 Hz) , 87.2 (d, *J* = 186.0 Hz), 33.5 (d, *J* = 20.9 Hz), 30.4 (d, *J* = 3.2 Hz); **^{19}F{^{1}H} NMR** (376 MHz, $CDCl_3$) δ -62.43 (s), -193.97 (s). HRMS [ESN]: *m/z* $[M-H]^-$ cal. $C_{11}H_9O_2F_4$, 249.0539, observed 249.0538.

2-Fluoro-4-(4-methoxyphenyl)butanoic acid (A4)

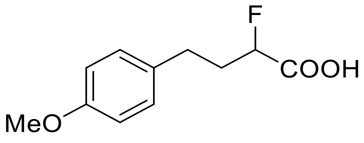

The title compound **A4** was prepared following the synthetic method (step 1) of general procedure **A**. Purification by column chromatography using (4:6

ethyl acetate/petroleum ether (40-60 °C)) as eluent, yellow gummy liquid, 680 mg, 64% yield; **^{1}H NMR** (400 MHz, $CDCl_3$) δ 8.13 (s, 1H), 7.12 (d, J = 8.6 Hz, 2H), 6.84 (d, J = 8.6 Hz, 2H), 4.99 – 4.84 (m, 1H), 3.79 (s, 3H), 2.82 – 2.67 (m, 2H), 2.26 – 2.11 (m, 2H); **^{13}C NMR** (101 MHz, $CDCl_3$) δ 175.7 (d, J = 24.1 Hz), 158.2, 132.0, 129.7, 114.1, 87.4 (d, J = 185.0 Hz), 55.4, 34.1 (d, J = 20.8 Hz), 29.6 (d, J = 3.1 Hz); **^{19}F{^{1}H} NMR** (376 MHz, $CDCl_3$) δ -193.62 (s). **HRMS** (ESN): *m/z* $[M-H]^-$ cal. $C_{11}H_{12}O_3F$, 211.0778, observed 211.0770.

4-(2-Bromophenyl)-2-fluorobutanoic acid (A5)

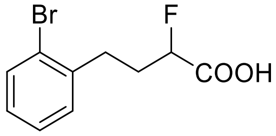

The title compound **A5** was prepared following the synthetic method (step 1) of general procedure **A**. Purification by column chromatography using (4:6 ethyl acetate/petroleum ether (40-60 °C)) as eluent, yellow gummy liquid, 945 mg, 72% yield; **^{1}H NMR** (400 MHz, $CDCl_3$) δ 10.98 (s, 1H), 7.59 (d, J = 7.8 Hz, 1H), 7.30 – 7.28 (m, 2H), 7.16 – 7.12 (m, 1H), 5.02 (ddd, J = 48.9, 7.9, 4.2 Hz, 1H), 3.07 – 2.94 (m, 2H), 2.44 – 2.23 (m, 2H). **^{19}F{^{1}H} NMR** (376 MHz, $CDCl_3$) δ -193.45 (s). **^{13}C NMR** (101 MHz, $CDCl_3$) δ 175.8, 139.4, 133.2, 130.8, 128.4, 127.8, 124.5, 87.6 (d, J = 185.8 Hz), 32.2 (d, J = 20.7 Hz), 31.2 (d, J = 3.2 Hz).; **HRMS [ESN]** m/z: $[M-H]^-$ Calcd. for $C_{10}H_9{}^{79}BrFO_2$, 258.9770; Found: 258.9770.

2-Fluoro-4-(4-(methoxycarbonyl)phenyl)butanoic acid (A6)

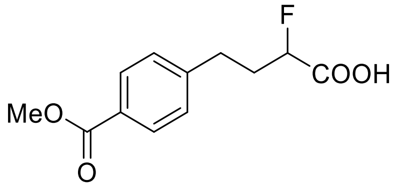

The title compound **A6** was prepared following the synthetic method (step 1) of general procedure **A**. Purification by column chromatography using (5:5 ethyl acetate/petroleum ether (40-60 °C)) as eluent, yellow gummy liquid, 900 mg, 75% yield; ^{1}H NMR (400 MHz, $CDCl_3$) δ 7.97 (d, J = 8.3 Hz, 2H), 7.28 (d, J = 8.3 Hz, 2H), 6.91 (br s, 1H), 5.01 – 4.86 (m, 1H), 3.90 (s, 3H), 2.88 (td, J = 8.1, 1.7 Hz, 2H), 2.32 – 2.21 (m, 2H). **^{19}F{^{1}H} NMR** (376 MHz, $CDCl_3$) δ -193.42 (s). **^{13}C NMR** (101 MHz, $CDCl_3$) δ 174.5 (d, J = 24.1 Hz), 167.3, 145.5, 130.1, 128.7, 128.6, 87.4 (d, J = 185.6 Hz), 52.3, 33.5 (d, J = 20.9 Hz), 30.6 (d, J = 3.2 Hz). **HRMS** [ESN]: *m/z* $[M-H]^-$ cal. $C_{12}H_{12}O_4F$, 239.0723, observed 239.0720.

2-Fluoro-3-(4-(methoxycarbonyl)phenyl)propanoic acid (A7)

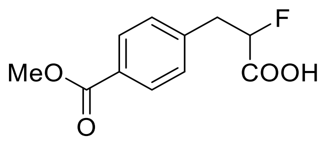

The title compound **A1** was prepared following the synthetic method (step 1) of general procedure **A**. Purification by column chromatography using (5:5 ethyl acetate/petroleum ether (40-60 °C)) as eluent, yellow gummy liquid, 770 mg, 68% yield; **^{1}H NMR** (400 MHz, $CDCl_3$) δ 8.00 (d, J = 8.3 Hz, 2H), 7.62 (br s, 1H), 7.35 (d, J = 8.2 Hz, 2H), 5.19 (ddd, J = 48.4, 7.7, 3.9 Hz, 1H), 3.91 (s, 3H), 3.41 – 3.19 (m, 2H); **^{19}F{^{1}H} NMR** (376 MHz, $CDCl_3$) δ -190.51 (s); **^{13}C NMR** (101 MHz, $CDCl_3$) δ 173.5 (d, J = 24.3 Hz), 167.2, 140.2, 130.1, 129.7, 129.4, 88.4 (d, J = 188.6 Hz), 52.4, 38.4 (d, J = 20.7 Hz). **HRMS** [ESP]: *m/z* $[M+H]^+$ cal. $C_{11}H_{12}O_4F$, 227.0728, observed 227.0720.

2-Fluoro-2-methyl-4-phenylbutanoic acid (A8)

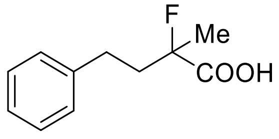

To a solution of diisopropylamine (0.85 mL, 6.0 mmol) in THF (10 mL) was added *n*-BuLi (1.6 M in hexanes, 3.5 mL) at -78 °C. After 10 minutes, a solution of methyl 2-fluoro-4-phenylbutanoate (980 mg, 5.0 mmol) in THF (10 mL) was added dropwise. The mixture was stirred for 10 minutes at -78 °C, then MeI (0.5 mL, 6.5 mmol) was added. The mixture was stirred at -78 °C until full conversion. After completion, the mixture was poured into H_2O (50 mL) and extracted with EtOAc (3 x 40 mL). The combined organic layers were dried over anhydrous $MgSO_4$, filtered and concentrated under reduced pressure. The crude was directly subjected to the next step without purification.

To a solution of the crude methyl-ester in 3:3:1 mixture of THF:MeOH:H_2O [0.36 M], lithium hydroxide (monohydrate) (12.5 mmol, 2.5 equiv.) was added in one portion and stirred overnight at room temperature. After the full conversion, the reaction mixture was concentrated, diluted with H_2O (20 mL), acidified to pH 1 with 1 M HCl and extracted with ethyl acetate (3 × 50 mL). The combined organic phases were washed with saturated brine (20 mL), dried over anhydrous $MgSO_4$, filtered and concentrated under reduced pressure. The residue was purified by flash column chromatography using (5:2 ethyl acetate/petroleum ether (40-60 °C)) to afford 2-fluoro-2-methyl-4-phenylbutanoic acid. Yellow gummy liquid, 780 mg, 80% yield; **^{1}H NMR** (400 MHz, $CDCl_3$) δ 9.55 (s, 1H), 7.31 – 7.28 (m, 2H), 7.22 – 7.18 (m,

3H), 2.91 – 2.63 (m, 2H), 2.38 – 2.10 (m, 2H), 1.68 (d, J = 21.2 Hz, 3H). **^{19}F{^{1}H} NMR** (376 MHz, $CDCl_3$) δ -157.45 (s). **^{13}C NMR** (101 MHz, $CDCl_3$) δ 177.6 (d, J = 26.4 Hz), 140.6, 128.7, 128.5, 126.4, 94.6 (d, J = 185.5 Hz), 40.1 (d, J = 22.2 Hz), 29.7 (d, J = 3.7 Hz), 23.8 (d, J = 24.1 Hz); **HRMS [ESN]** m/z: $[M-H]^+$ Calcd. for $C_{11}H_{12}FO_2$, 195.0829; Found:195.0821.

(3-bromo-3-fluoropropyl)benzene (1a)

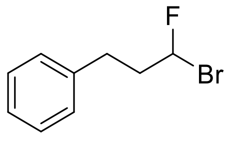

The title compound **1a** was prepared following the synthetic method (step 2) of general procedure **A**. Purification by column chromatography using hexane as eluent, colourless oil, 340 mg, 78% yield; **^{1}H NMR** (400 MHz, $CDCl_3$) δ 7.26 – 7.07 (m, 5H), 6.30 (dt, J = 50.2, 5.5 Hz, 1H), 2.74 (t, J = 7.6 Hz, 2H), 2.53 – 2.28 (m, 2H). **^{19}F{^{1}H}NMR** (376 MHz, $CDCl_3$) δ -132.44 (s). All spectroscopic data were in accordance with the literature.[1]

(3-Iodo-3-fluoropropyl)benzene (1a′)

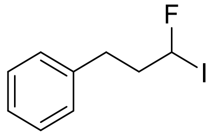

The title compound **1a′** was prepared following the synthetic method (step 2) of general procedure **A**. The crude was purified through a short alumina pad, concentrated in vacuo to gave the pure product. Colourless oil, 450 mg, 84% yield; **^{1}H NMR** (400 MHz, $CDCl_3$) δ 7.37 – 7.32 (m, 2H), 7.29 – 7.22 (m, 3H), 6.80 (ddd, J = 50. 6. 5 Hz 1H), 2.88 – 2.77 (m, 2H), 2.72 – 2.46 (m, 2H). **^{19}F{^{1}H}NMR** (376 MHz, $CDCl_3$) δ -138.23 (s). All spectroscopic data were in accordance with the literature.[1]

(3-Chloro-3-fluoropropyl)benzene (1a″)

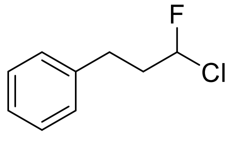

The (3-iodo-3-fluoropropyl)benzene (1.0 mmol) and tetrabutylammonium chloride (2.0 mmol) were refluxed in chloroform (10 mL) for 5 h. After full conversion, the reaction mixture was cooled to room temperature and quenched with 1 M aq. Na_2SO_3 (10 mL). The mixture was diluted with DCM (20 mL) and the organic layer was washed with Na_2SO_3 (1 x 20 mL), dried over anhydrous $MgSO_4$, filtered and concentrated under reduced pressure. The crude was purified by flash column chromatography using (hexane as eluent) to afford the

product. Colourless oil, 130 mg, 75% yield; **^{1}H NMR** (400 MHz, $CDCl_3$) δ 7.29 – 7.10 (m, 5H), 6.05 (dt, J = 50.8, 5.4 Hz, 1H), 2.76 (t, J = 8.1 Hz, 2H), 2.45 – 2.19 (m, 2H). **$^{19}F\{^1H\}$NMR** (376 MHz, $CDCl_3$) δ -131.57 (s). All spectroscopic data were in accordance with the literature.[1]

1-(3-Bromo-3-fluoropropyl)-4-methylbenzene (1b)

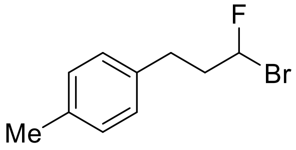

The title compound **1b** was prepared following the synthetic method (step 2) of general procedure **A**. Purification by column chromatography using hexane as eluent. Colourless oil, 370 mg, 80% yield; **^{1}H NMR** (400 MHz, $CDCl_3$) δ 7.15 – 7.07 (m, 4H), 6.40 (dt, J = 50.3, 5.5 Hz, 1H), 2.80 (t, J = 7.7 Hz, 2H), 2.61 – 2.37 (m, 2H), 2.33 (s, 1H). **$^{19}F\{^1H\}$NMR** (376 MHz, $CDCl_3$) δ -131.97 (s). All spectroscopic data were in accordance with the literature.[1]

4-(3-Bromo-3-fluoropropyl)-1,1'-biphenyl (1c)

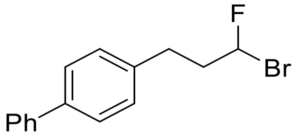

The title compound **1c** was prepared following the synthetic method (step 2) of general procedure **A**. Purification by column chromatography using hexane as eluent. Colourless sticky solid, 400 mg, 70% yield; **^{1}H NMR** (400 MHz, $CDCl_3$) δ 7.61 – 7.55 (m, 4H), 7.47 – 7.43 (m, 2H), 7.37 – 7.34 (m, 1H), 7.30 –7.28 (m, 2H), 6.46 (dt, J = 50.2, 5.5 Hz, 1H), 2.90 (t, J = 7.7 Hz, 1H), 2.68 – 2.44 (m, 1H); **^{13}C NMR** (101 MHz, $CDCl_3$) δ 140.9, 139.6, 138.6, 129.0, 128.9, 127.6, 127.4, 127.1, 94.8 (d, J = 252.3 Hz), 42.2 (d, J = 19.2 Hz), 31.0 (d, J = 4.5 Hz); **$^{19}F\{^1H\}$NMR** (376 MHz, $CDCl_3$) δ -132.33 (s); **HRMS [EI$^+$]** $[M]^+$ Calcd. for $C_{15}H_{14}{}^{79}BrF$ 292.0257; Found: 292.0255.

1-(3-Bromo-3-fluoropropyl)-4-chlorobenzene (1d)

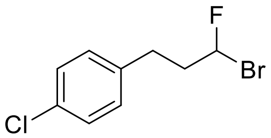

The title compound **1d** was prepared following the synthetic method (step 2) of general procedure **A**. Purification by column chromatography using hexane as eluent. Colourless oil, 385 mg, 76% yield; **^{1}H NMR** (400 MHz, $CDCl_3$) δ 7.28 (d, J = 8.4 Hz, 2H), 7.14 (d, J = 8.6 Hz, 2H), 6.40 (dt, J = 50.2, 5.4 Hz, 1H), 2.82 (t, J = 7.6 Hz, 2H), 2.60 –

2.37 (m, 2H). **^{19}F{^{1}H}NMR** (376 MHz, $CDCl_3$) δ -132.76 (s). All spectroscopic data were in accordance with the literature.[1]

1-(3-Bromo-3-fluoropropyl)-4-fluorobenzene (1e)

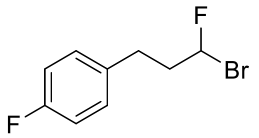

The title compound **1e** was prepared following the synthetic method (step 2) of general procedure **A**. Purification by column chromatography using hexane as eluent. Yellow oil, 365 mg, 78% yield; **^{1}H NMR** (400 MHz, $CDCl_3$) δ 7.19 – 7.13 (m, 2H), 7.03 – 6.96 (m, 2H), 6.40 (dt, J = 50.2, 5.4 Hz, 1H), 2.82 (t, J = 7.7 Hz, 2H), 2.61 – 2.36 (m, 2H); **^{13}C NMR** (101 MHz, $CDCl_3$) δ 161.8 (d, J = 244.6 Hz), 135.2 (d, J = 3.2 Hz), 130.0 (d, J = 7.9 Hz), 115.6 (d, J = 21.3 Hz), 94.6 (d, J = 252.3 Hz), 42.3 (d, J = 19.3 Hz), 30.6 (d, J = 4.5 Hz); **^{19}F{^{1}H}NMR** (376 MHz, $CDCl_3$) δ -116.57 (s), -132.71 (s). **HRMS [EI^{+}]** m/z: $[M]^{+\cdot}$ Calcd. for $C_9H_9{}^{79}BrF_2$ 233.9850; Found: 233.9846.

1-(3-Bromo-3-fluoropropyl)-4-(trifluoromethyl)benzene (1f)

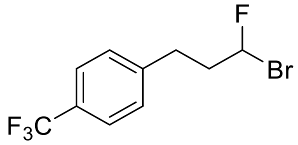

The title compound **1f** was prepared following the synthetic method (step 2) of general procedure **A**. Purification by column chromatography using hexane as eluent. Yellow oil, 364 mg, 64% yield; **^{1}H NMR** (400 MHz, $CDCl_3$) δ 7.58 (d, J = 8.0 Hz, 2H), 7.33 (d, J = 8.0 Hz, 2H), 6.43 (dt, J = 50.1, 5.3 Hz, 1H), 2.92 (t, J = 7.3 Hz, 2H), 2.63 – 2.41 (m, 2H). **^{13}C NMR** (101 MHz, $CDCl_3$) δ 143.7, 129.5 (app. d, J = 32.4 Hz), 128.9, 125.8 (q, J = 3.8 Hz), 124.3 (q, J = 271.9 Hz), 94.3 (d, J = 252.4 Hz), 41.8 (d, J = 19.5 Hz), 31.1 (d, J = 4.4 Hz); **^{19}F{^{1}H} NMR** (376 MHz, $CDCl_3$) δ -62.47 (s), -132.91 (s). **HRMS [EI^{+}]** $[M]^{+}$ Calcd. for $C_{10}H_9{}^{79}BrF_4$ 283.9818; Found: 283.9822.

4-(3-Bromo-3-fluoropropyl)-2-iodo-1-methoxybenzene (1g)

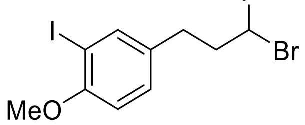

The title compound **1g** was prepared following the synthetic method (step 2) of general procedure **A**. Purification by column chromatography using (1:9 ethyl acetate/petroleum ether (40-60 °C)) as eluent. Yellow oil, 298 mg, 40% yield; **^{1}H NMR** (400 MHz, $CDCl_3$) δ 7.62 (d, J = 2.2 Hz, 1H), 7.13 (dd, J = 8.3, 2.2 Hz, 1H), 6.76 (d, J = 8.4 Hz,

1H), 6.40 (dt, J = 50.2, 5.4 Hz, 1H), 3.86 (s, 3H), 2.74 (t, J = 7.5 Hz, 2H), 2.58 – 2.34 (m, 2H); ^{13}**C NMR** (101 MHz, $CDCl_3$) δ 157.0, 139.4, 133.7, 129.6, 111.1, 94.5 (d, J = 252.3 Hz), 86.3, 56.5, 42.2 (d, J = 19.2 Hz), 29.9 (d, J = 4.5 Hz). ^{19}**F{^{1}H} NMR** (376 MHz, $CDCl_3$) δ -132.66 (s); **HRMS [EI$^+$]** $[M]^+$ Calcd. for $C_{10}H_{11}{}^{79}BrIFO$, 371.9016; Found: 371.9012.

1-Bromo-2-(3-bromo-3-fluoropropyl)benzene (1h)

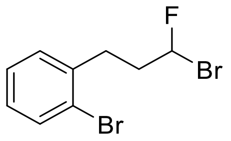

The title compound **1h** was prepared following the synthetic method (step 2) of general procedure **A**. Colourless oil, 399 mg, 68% yield; ^{1}**H NMR** (400 MHz, $CDCl_3$) δ 7.58 (d, J = 7.7 Hz, 1H), 7.33 – 7.24 (m, 2H), 7.15 – 7.11 (m, 1H), 6.49 (dt, J = 50.2, 5.4 Hz, 1H), 3.02 – 2.97 (m, 2H), 2.67 – 2.42 (m, 2H); ^{13}C NMR (101 MHz, $CDCl_3$) δ 139.0, 133.3, 130.7, 128.5, 127.9, 124.5, 94.6 (d, J = 252.6 Hz), 40.4 (d, J = 19.3 Hz), 31.9 (d, J = 4.3 Hz); ^{19}**F NMR** (376 MHz, $CDCl_3$) δ -132.16 (m); **HRMS [EI$^+$]** $[M]^+$ Calcd. for $C_9H_9{}^{79}Br_2F$, 293.9050; Found: 293.9050.

5-Bromo-5-fluoro-1-phenylpentan-1-one (1i)

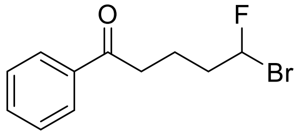

The title compound **1i** was synthesized according to literature procedure.[1] Purification by column chromatography using (03:97 ethyl acetate/petroleum ether (40-60 °C)) as eluent. (5-Bromo-5-fluoropentyl)benzene was used as the starting material. Yellow oil, 337 mg, 65% yield; ^{1}**H NMR** (400 MHz, $CDCl_3$) δ 7.98 – 7.93 (m, 2H), 7.61 – 7.54 (m, 1H), 7.51 – 7.44 (m, 2H), 6.52 (dt, J = 50.3, 5.3 Hz, 1H), 3.07 (t, J = 7.1 Hz, 2H), 2.42 – 2.15 (m, 2H), 2.06 – 1.92 (m, 2H); ^{19}**F{^{1}H}NMR** (376 MHz, $CDCl_3$) δ -130.91 (s). All spectroscopic data were in accordance with the literature.[1]

Methyl 4-(3-bromo-3-fluoropropyl)benzoate (1j)

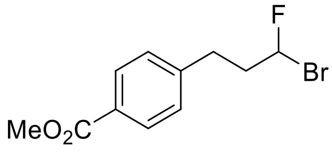

The title compound **1j** was prepared following the synthetic method (step 2) of general procedure **A**. Purification by column chromatography using (2:8 ethyl acetate/petroleum ether (40-60 °C)) as eluent. Colourless oil, 370 mg, 67% yield; ^{1}**H NMR** (400 MHz, $CDCl_3$) δ 7.91 (d, J = 8.3 Hz, 2H), 7.20 (d, J = 8.4 Hz, 2H), 6.34 (dt, J = 50.1, 5.4 Hz,

1H), 3.83 (s, 3H), 2.82 (t, *J* = 7.4 Hz, 2H), 2.58 – 2.32 (m, 2H); **^{19}F NMR** (376 MHz, $CDCl_3$) δ -132.71 (d, *J* = 2.1 Hz); **^{13}C NMR** (101 MHz, $CDCl_3$) δ 167.0, 144.9, 130.1, 128.7, 128.6, 94.4 (d, *J* = 252.4 Hz), 52.2, 41.7 (d, *J* = 19.4 Hz), 31.3 (d, *J* = 4.4 Hz); **HRMS [EI$^+$]** $[M]^+$ Calcd for $C_{11}H_{12}{}^{79}BrFO_2$, 273.9999; Found: 274.0001.

(5-Bromo-5-fluoropentyl)benzene (1k)

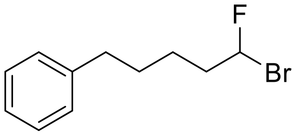

The title compound **1k** was prepared following the synthetic method (step 2) of general procedure **A**. Purification by column chromatography using hexane as eluent. Colourless oil, 392 mg, 80% yield; **^{1}H NMR** (400 MHz, $CDCl_3$) δ 7.32 – 7.27 (m, 2H), 7.23 – 7.16 (m, 3H), 6.45 (dt, *J* = 50.4, 5.4 Hz, 1H), 2.65 (t, *J* = 7.4 Hz, 2H), 2.36 – 2.10 (m, 2H), 1.74 – 1.66 (m, 2H), 1.62 – 1.51 (m, 2H); **^{19}F{^{1}H}NMR** (376 MHz, $CDCl_3$) δ -130.41 (s) All spectroscopic data were in accordance with the literature.[1]

2-(3-Bromo-3-fluoropropyl)naphthalene (1l)

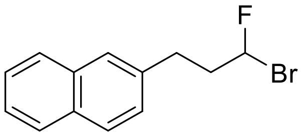

The title compound **1l** was prepared following the synthetic method (step 2) of general procedure **A**. Purification by column chromatography using hexane as eluent. Colourless oil, 438 mg, 82% yield; **^{1}H NMR** (400 MHz, $CDCl_3$) δ 7.84-7.79 (m, 3H), 7.66 (s, 1H), 7.52 – 7.43 (m, 2H), 7.34 (dd, *J* = 8.5, 1.7 Hz, 1H), 6.45 (dt, *J* = 50.2, 5.5 Hz, 1H), 3.02 (t, *J* = 7.7 Hz, 2H), 2.74 – 2.48 (m, 2H). **^{19}F{^{1}H}NMR** (376 MHz, $CDCl_3$) δ -132.38 (s) All spectroscopic data were in accordance with the literature.[1]

2-(2-Bromo-2-fluoroethyl)naphthalene (1m)

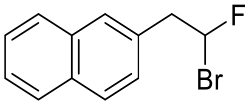

The title compound **1m** was prepared following the synthetic method (step 2) of general procedure **A**. Purification by column chromatography using hexane as eluent. Off-white solid, 380 mg, 75% yield; **^{1}H NMR** (400 MHz, $CDCl_3$) δ 7.86 – 7.82 (m, 3H), 7.73 (s, 1H), 7.53 – 7.46 (m, 2H), 7.37 (dd, *J* = 8.4, 1.2 Hz, 1H), 6.65 (ddd, *J* = 50.1, 6.6, 4.7 Hz, 1H), 3.80 – 3.54 (m, 2H); **^{19}F{^{1}H}NMR** (376 MHz, $CDCl_3$) δ -131.22 (s) .All spectroscopic data were in accordance with the literature.[1]

Methyl 4-(2-bromo-2-fluoroethyl)benzoate (1n)

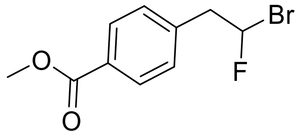

The title compound **1b** was prepared following the synthetic method (step 2) of general procedure **A**. Purification by column chromatography using (2:8 ethyl acetate/petroleum ether (40-60 °C)) as eluent. Colourless oil, 318 mg, 61% yield; **^{1}H NMR** (400 MHz, $CDCl_3$) δ 8.01 (d, J = 8.3 Hz, 2H), 7.33 (d, J = 8.1 Hz, 2H), 6.57 (ddd, J = 50.0, 6.4, 4.7 Hz, 1H), 3.91 (s, 3H), 3.65 – 3.45 (m, 2H). **^{19}F{^{1}H}NMR** (376 MHz, $CDCl_3$) δ -131.91 (s); **^{13}C NMR** (101 MHz, $CDCl_3$) δ 166.9, 139.6 (d, J = 3.5 Hz), 130.1, 129.9, 129.7, 93.6 (d, J = 254.6 Hz), 52.3, 46.9 (d, J = 20.1 Hz); **HRMS [ESP]** $[M+H]^+$ Calcd. for $C_{10}H_{11}{}^{79}BrO_2F$, 260.9934; Found: 260.9926.

4-(3-Bromo-3-fluoropropyl)benzoic acid (1o)

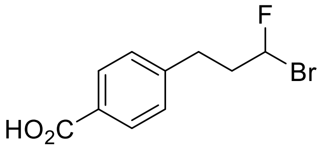

The title compound **1o** was prepared by the ester hydrolysis of **1j (**1.0 mmol) used following general procedure **A**. Purification by column chromatography using (3:7 ethyl acetate/petroleum ether (40-60 °C)) as eluent. White solid, 220 mg, 84%; **^{1}H NMR** (400 MHz, $CDCl_3$) δ 8.07 (d, J = 8.3 Hz, 2H), 7.32 (d, J = 8.3 Hz, 2H), 6.44 (dt, J = 50.1, 5.3 Hz, 1H), 2.95 – 2.91 (m, 2H), 2.65 – 2.43 (m, 2H). **^{19}F NMR** (376 MHz, $CDCl_3$) δ -132.75 (ddd, J = 36.6, 19.1, 16.9 Hz). **^{13}C NMR** (101 MHz, $CDCl_3$) δ 172.3, 146.1, 130.8, 128.8, 127.9, 94.3 (d, J = 252.4 Hz), 41.7 (d, J = 19.5 Hz), 31.4 (d, J = 4.4 Hz).; **HRMS [ESN]** $[M-H]^-$ Calcd. for $C_{10}H_9{}^{79}BrFO_2$, 258.9780; Found: 258.9770.

2-(6-Bromo-6-fluorohexyl)isoindoline-1,3-dione (1p)

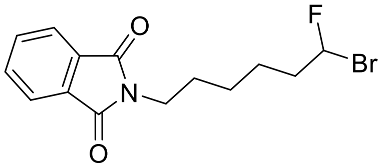

The title compound **1p** was prepared following the synthetic method (step 2) of general procedure **A**. Purification by column chromatography using (1:4 ethyl acetate/petroleum ether (40-60 °C)) as eluent. Yellow oil, 440 mg, 67% yield; **^{1}H NMR** (400 MHz, $CDCl_3$) δ 7.84 (dd, J = 5.5, 3.0 Hz, 2H), 7.72 (dd, J = 5.4, 3.1 Hz, 2H), 6.44 (dt, J = 50.4, 5.4 Hz, 1H), 3.69 (t, J = 7.2 Hz, 2H), 2.34 – 2.06 (m, 2H), 1.74 – 1.67 (m, 2H), 1.58 –

1.52 (m, 2H), 1.47 – 1.35 (m, 2H); **^{19}F{^{1}H}NMR** (376 MHz, $CDCl_3$) δ -130.63 (s). All spectroscopic data were in accordance with the literature.[1]

_N_-(3-bromo-3-fluoropropyl)-4-methyl-_N_-phenylbenzenesulfonamide (1q)

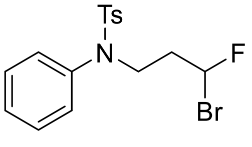

The title compound **1q** was prepared following the synthetic method (step 2) of general procedure **A**. Purification by column chromatography using (1:4 ethyl acetate/petroleum ether (40-60 °C)) as eluent. Yellow oil, 572 mg, 74% yield; **^{1}H NMR** (400 MHz, $CDCl_3$) δ 7.46 (d, J = 8.3 Hz, 2H), 7.36 – 7.30 (m, 3H), 7.28 – 7.23 (m, 2H), 7.06 – 7.01 (m, 2H), 6.54 (dt, J = 50.4, 5.4 Hz, 1H), 3.73 (dd, J = 13.3, 6.5 Hz, 2H), 2.54 – 2.25 (m, 5H); **^{19}F{^{1}H}NMR** (376 MHz, $CDCl_3$) δ -134.02 (s). All spectroscopic data were in accordance with the literature.[1]

4-(3-Bromo-3-fluoropropyl)-_N_-methoxy-_N_-methylbenzamide (1r)

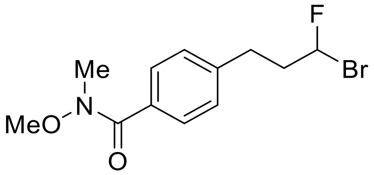

The title compound **1r** was prepared by adding $SOCl_2$ (0.6 mmol) and 2 drops of DMF to a solution of 4-(3-bromo-3-fluoropropyl)benzoic acid (0.5 mmol) in DCM (10 mL). The mixture was stirred at rt for 1 h, then concentrated under reduced pressure. To a solution of *N*-methoxymethylamine hydrochloride salt (0.6 mmol) and triethylamine (1.2 mmol) in DCM (10 mL), the crude acid chloride in DCM (10 mL) was added dropwise at 0 °C. After 1 hour the mixture was quenched with saturated $NH_4Cl_{(aq)}$ (10 mL). The organic layer was washed with 1M HCl (2 x 15 mL), dried over anhydrous $MgSO_4$, filtered and concentrated under reduced pressure. The residue was purified by flash column chromatography using (1:5 ethyl acetate/petroleum ether (40-60 °C)) to afford **1r**. Yellow oil, 137 mg, 90% yield; **^{1}H NMR** (400 MHz, $CDCl_3$) δ 7.64 (d, J = 8.2 Hz, 2H), 7.23 (d, J = 8.1 Hz, 2H), 6.41 (dt, J = 50.2, 5.4 Hz, 1H), 3.55 (s, 3H), 3.35 (s, 3H), 2.87 (t, J = 7.4 Hz, 2H), 2.64 – 2.38 (m, 2H); **^{13}C NMR** (101 MHz, $CDCl_3$) δ 169.8, 142.3, 132.5, 128.8, 128.2, 94.5 (d, J = 252.3 Hz), 61.2, 41.8 (d, J = 19.4 Hz), 33.9, 31.2 (d, J = 4.4 Hz) ; **^{19}F{^{1}H}NMR** (376 MHz, $CDCl_3$) δ -132.70 (s); **HRMS [ESP]** m/z: $[M+H]^+$ Calcd. for $C_{12}H_{16}{}^{79}BrFNO_2$, 304.0348; Found: 304.0357.

5-(3-Bromo-3-fluoropropyl)-3-(3,5-dichlorophenyl)-5-methyloxazolidine-2,4-dione (1s)

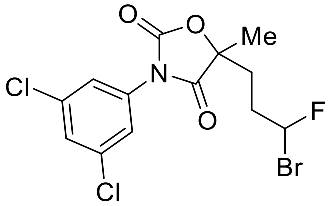

The title compound **1s** (1:1 d.r.) was prepared according to literature procedure using vinclozoline 0.4 mmol as starting substrate.[2] Purification by column chromatography using (1:5 ethyl acetate/petroleum ether (40-60 °C)) as eluent. White solid, 113 mg, 73% yield; **^{1}H NMR** (400 MHz, $CDCl_3$) δ 7.45 – 7.44 (m, 2H), 7.44 – 7.41 (m, 1H), 6.60– 6.44 (m, 1H), 2.48 – 2.33 (m, 1H), 2.32 – 2.14 (m, 3H), 1.70 (s, 3H); **^{19}F{^{1}H}NMR** (376 MHz, $CDCl_3$) δ -132.92 (s,), -133.26 (s). All spectroscopic data were in accordance with the literature.[2]

2-Bromo-2-fluoro-3,4-dihydronaphthalen-1(2H)-one (1t)

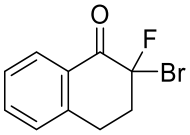

The title compound **1t** was prepared using 3,4-dihydronaphthalen-1(2H)-one (1.0 mmol) as starting substrate according to literature procedure.[3] Purification by column chromatography using (2:8 ethyl acetate/petroleum ether (40-60 °C)) as eluent. White solid, 185 mg, 76% yield; **^{1}H NMR** (400 MHz, $CDCl_3$) δ 8.15 (dd, J = 7.9, 1.2 Hz, 1H), 7.58 (td, J = 7.5, 1.4 Hz, 1H), 7.41 (t, J = 7.6 Hz, 1H), 7.29 (dd, J = 7.7, 0.5 Hz, 1H), 3.39 – 3.25 (m, 1H), 3.14 – 3.06 (m, 1H), 2.89 – 2.83 (m, 1H), 2.68 – 2.59 (m, 1H); **^{19}F{^{1}H}NMR** (376 MHz, $CDCl_3$) δ -114.50 (s). All spectroscopic data were in accordance with the literature.[3]

2-Bromo-2-fluoro-1-phenylbutan-1-one (1u)

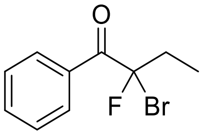

The title compound **1u** was prepared using 1-phenylbutanone (5.0 mmol) according to literature procedure.[4] Purification by column chromatography using (2:8 ethyl acetate/petroleum ether (40-60 °C)) as eluent. Colorless oil, 532 mg, 43% yield; **^{1}H NMR** (400 MHz, $CDCl_3$) δ 8.21 – 8.14 (m, 2H), 7.64 – 7.58 (m, 1H), 7.52 – 7.45 (m, 2H), 2.70 – 2.46 (m, 2H), 1.17 (t, J = 7.3 Hz, 3H); **^{19}F{^{1}H}NMR** (376 MHz, $CDCl_3$) δ -118.10 (s). All spectroscopic data were in accordance with the literature.[4]

(3-Bromo-3-fluorobutyl)benzene (1v)

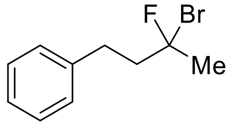

The title compound **1v** was prepared according to literature procedure.[5] A mixture of 2-fluoro-2-methyl-4-phenylbutanoic acid (0.8 mmol), oxalyl chloride (0.5 mL, 5.7 mmol) and 1-2 drops of DMF in DCM (3 mL) was stirred at room temperature. After 2.5 h, the reaction mixture was concentrated to dryness under reduced pressure to yield the crude acid chloride. The mixture of acid chloride, sodium salt of *N*-hydroxy-2-thiopyridone (120 mg, 0.8 mmol) and (4-dimethylamino)pyridine (DMAP) (25 mg, 0.2 mmol) in bromotrichloromethane (5 mL) was heated at reflux for 2 h. After completion, the solvent was removed under vacuo and crude was purified by flash column chromatography using (03:97 ethyl acetate/petroleum ether (40-60 °C)) to afford **1v**. Colourless oil, 112 mg, 60% yield; **^{1}H NMR** (400 MHz, $CDCl_3$) δ 7.27 – 7.18 (m, 2H), 7.18 – 7.12 (m, 3H), 2.90 – 2.82 (m, 2H), 2.42-2.32 (m, 2H), 2.08 (d, J = 19.7 Hz, 3H); **^{13}C NMR** (101 MHz, $CDCl_3$) δ 140.4, 128.7, 128.5, 126.4, 109.6 (d, J = 253.7 Hz), 48.0 (d, J = 21.3 Hz), 33.1 (d, J = 23.4 Hz), 31.7 (d, J = 4.2 Hz); **^{19}F{^{1}H}NMR** (376 MHz, $CDCl_3$) δ -90.54 (s). **HRMS [EI$^+$]** m/z: $[M]^+$ Calcd. for $C_{10}H_{12}{}^{79}BrF$ 230.0100; Found: 230.0102.

(4-Bromo-4-fluoropiperidin-1-yl)(phenyl)methanone (1w)

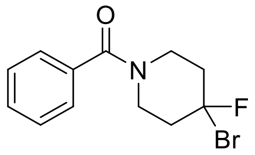

The title compound **1w** was prepared following procedure.[5] A mixture of 1-benzoyl-4-fluoropiperidine-4-carboxylic acid (0.8 mmol), oxalyl chloride (0.5 mL, 5.7 mmol) and 1-2 drops of DMF in DCM (3 mL) was stirred at room temperature. After 2.5 hours the reaction mixture was concentrated to dryness under reduced pressure to yield the crude acid chloride. The mixture of acid chloride, sodium salt of *N*-hydroxy-2-thiopyridone (120 mg, 0.8 mmol) and DMAP (25 mg, 0.2 mmol) in bromotrichloromethane (5 mL) was heated at reflux for 2 h. After completion, the solvent was removed under vacuo and crude was purified by flash column chromatography using (1:5 ethyl acetate/petroleum ether (40-60 °C)) to afford **1w**. Colourless gummy liquid, 103 mg, 45% yield. **^{1}H NMR** (400 MHz, $CDCl_3$) δ 7.43 – 7.37 (m, 5H), 3.86 – 3.56 (m, 4H), 2.47 – 2.30 (m, 4H).; **^{19}F{^{1}H} NMR** (376 MHz, $CDCl_3$) δ -97.74 (s); **^{13}C NMR** (101 MHz, $CDCl_3$) δ 170.6, 135.2, 130.2, 128.7, 127.0, 107.2 (d, J = 255.4 Hz),

45.2, 42.5, 41.9, 39.8. **HRMS [ESP]** m/z: $[M+H]^+$ Calcd. for $C_{12}H_{14}{}^{81}BrFNO$, 288.0222; Found: 288.0.233.

1-(4-Bromo-4-fluorocyclohexyl)-4-chlorobenzene (1x)

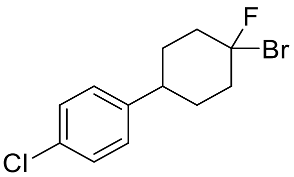

The title compound **1x** (3:2 d.r.) was prepared following procedure.[5] A mixture of 4-(4-chlorophenyl)-1-fluorocyclohexane-1-carboxylic acid (0.8 mmol), oxalyl chloride (0.5 mL, 5.7 mmol) and 1-2 drops of DMF in DCM (3 mL) was stirred at room temperature. After 2.5 h, the reaction mixture was concentrated to dryness under reduced pressure to yield the crude acid chloride. The mixture of acid chloride, sodium salt of *N*-hydroxy-2-thiopyridone (120 mg, 0.8 mmol) and DMAP (25 mg, 0.2 mmol) in bromotrichloromethane (5 mL) was heated at reflux for 2 h. After completion, the solvent was removed under vacuo and crude was purified by flash column chromatography using (02:98 ethyl acetate/petroleum ether (40-60 °C)) to afford **1x**. Colourless gummy liquid, 152 mg, 65% yield. **^{1}H NMR** (400 MHz, $CDCl_3$) δ 7.23 – 7.03 (m, 4H), 2.73 – 2.65 (m, 1H), 2.57 – 2.45 (m, 2H), 2.41 – 2.14 (m, 1H), 2.04 – 1.62 (m, 5H); **^{19}F{^{1}H}NMR** (376 MHz, $CDCl_3$) δ -84.88 (s), -103.14 to -104.72 (m); **^{13}C NMR** (101 MHz, $CDCl_3$) δ 143.8, 143.6 (d, J = 2.5 Hz), 132.3, 132.3, 128.8, 128.8, 128.3, 128.2, 113.0 (d, J = 256.5 Hz), 108.2 (d, J = 254.2 Hz), 43.1 (d, J = 22.0 Hz), 42.3 (d, J = 1.7 Hz), 42.1 (d, J = 18.5 Hz), 41.7, 31.4, 31.3, 31.2; **HRMS [EI$^+$]** m/z: $[M]^+$ Calcd. for $C_{12}H_{13}{}^{79}BrFCl$, 289.9867; Found: 289.9867.

4-(Bromofluoromethyl)-1,1'-biphenyl (1y)

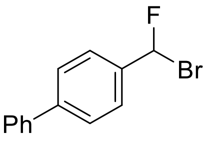

The title compound **1y** was prepared following the procedure.[6] A mixture of 2-([1,1'-biphenyl]-4-yl)-2-fluoroacetic acid (2.0 mmol), bromoisocyanurate (1.0 mmol), tetrabutylammonium tribromide (0.5 mmol) in 1,2-dichloromethane (10 mL) was stirred at rt under fluorescent room light irradiation for 3 h. The reaction mixture was quenched with 1 M aq. Na_2SO_3 (20 mL). The organic layer was washed further with Na_2SO_3 (2 x 20 mL), dried over anhydrous $MgSO_4$, filtered and concentrated under reduced pressure. The crude was purified by flash column chromatography using hexane as eluent to afford **1y**. Colourless oil, 212 mg, 40% yield. **^{1}H NMR** (400 MHz, $CDCl_3$) δ 7.69 – 7.63 (m, 2H), 7.63 – 7.58 (m, 3H), 7.55 (d, J = 19.9 Hz, 1H), 7.49-7.37 (m, 4H); **^{13}C NMR** (101 MHz, $CDCl_3$) δ 143.3 (d, J = 1.3

Hz), 140.22, 137.7 (d, J = 19.8 Hz), 129.1, 128.1, 127.6, 127.3, 125.7 (d, J = 6.0 Hz), 92.1 (d, J = 253.0 Hz); **^{19}F{^{1}H}NMR** (376 MHz, $CDCl_3$) δ -129.72 (s). **HRMS [EI^{+}]** m/z: $[M-Br]^{+}$ Calcd. for $C_{13}H_{10}F$, 185.0761; Found: 185.0761.

(3,3-Difluoro-3-iodopropyl)benzene (1z)

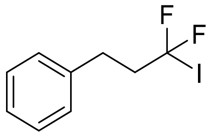

The title compound **1z** was prepared following the synthetic method decarboxylative iodination of 2,2-difluoro-4-phenylbutanoic acid (step 2) of general procedure **A.** Purification by column chromatography using hexane as eluent. colourless oil, 350 mg, 62% yield; **^{1}H NMR** (400 MHz, $CDCl_3$) δ 7.26 – 7.20 (m, 2H), 7.18 – 7.09 (m, 3H), 2.83 – 2.77 (m, 2H), 2.61 – 2.48 (m, 2H). **^{19}F{^{1}H} NMR** (376 MHz, $CDCl_3$) δ -36.56 (s). All spectroscopic data were in accordance with the literature.[7]

4-(Bromodifluoromethyl)-1,1'-biphenyl (1aa)

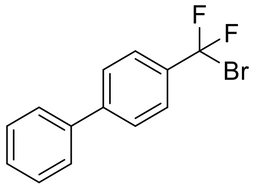

The title compound **1aa** was prepared according to literature procedure using 4-(difluoromethyl)-1,1'-biphenyl (1.0 mmol) starting substrate.[8] Purification by column chromatography using hexane as eluent. White solid, 205 mg, 72%; **^{1}H NMR** (400 MHz, $CDCl_3$) δ 7.66 – 7.65 (m, 4H), 7.60 – 7.57 (m, 2H), 7.48 – 7.44 (m, 2H), 7.41 – 7.37 (m, 1H); **^{19}F{^{1}H} NMR** (376 MHz, $CDCl_3$) δ -43.16 (s). All spectroscopic data were in accordance with the literature.[8]

2-bromo-2,2-difluoro-1-(4-methoxyphenyl)ethan-1-one (1ab)

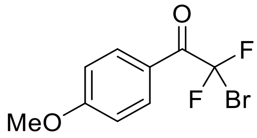

The title compound **1ab** was prepared according to literature procedure using ethyl 2-bromo-2,2-difluoroacetate (2.0 mmol) as starting substrate.[9] Purification by column chromatography using (5:95 ethyl acetate/petroleum ether (40-60 ^{o}C)) as eluent. Colourless oil, 318 mg, 60% yield; **^{1}H NMR** (400 MHz, $CDCl_3$) δ 8.12 (d, J = 9.1 Hz, 2H), 6.98 (d, J = 9.1 Hz, 2H), 3.90 (s, 3H); **^{19}F{^{1}H} NMR** (376 MHz, $CDCl_3$) δ -57.04 (s). All spectroscopic data were in accordance with the literature.[9]

Synthesis of 1,1-[^{19}F]difluoroalkane references

General Procedure B: $AgBF_4$ (0.44 mmol, 1.1 eq.) was added to a solution of an appropriate bromide (0.4 mmol, 1.0 eq.) in DCM (0.10 M) at room temperature. The reaction mixture was stirred at room temperature for 1h. The reaction mixture was filtered through a short pad of silica and concentrated *in vacuo*. The crude product was purified by silica gel column chromatography.

General Procedure C: DAST (0.4 mmol, 1.0 eq.) was added to a solution of an appropriate aldehyde (0.4 mmol, 1.0 eq.) in DCM (0.10 M) at room temperature. The reaction was stirred over night at room temperature before it was quenched with sat. $NaHCO_3$ (10 mL) and extracted with DCM (3 x 10 mL). The combined organic fractions were washed with brine, dried over $MgSO_4$, filtered, and concentrated *in vacuo.* The crude product was purified by silica gel column chromatography.

General Procedure D: A Schlenk tube was charged with Selectfluor (0.4 mmol, 2.0 equiv), silver nitrate (20 mol%) and the appropriate carboxylic acid (0.2 mmol, 1.0 equiv). The system was purged with N_2, then acetone (2 mL) and water (2 mL) were added. The reaction was stirred at 55 °C for 1 h. After completion, water (10 mL) was added and mixture was extracted with DCM (3 × 10 mL), dried over $MgSO_4$, filtered and concentrated *in vacuo.* The crude product was purified by silica gel column chromatography.

(3,3-Difluoropropyl)benzene (2a)

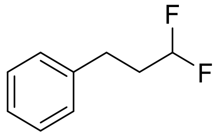

The title compound **2a** was prepared following the general procedure **B** using **1a** as starting substrate (0.4 mmol scale). Purification by column chromatography using hexane as eluent. Colourless oil, 48 mg, 80% yield. **^{1}H NMR** (400 MHz, $CDCl_3$) δ 7.29 – 7.10 (m, 5H), 5.73 (tt, J = 56.7, 4.5 Hz, 1H), 2.71 (t, J = 8.0 Hz, 2H), 2.16 – 1.99 (m, 2H). **^{13}C NMR** (101 MHz, $CDCl_3$) δ 139.9, 128.7, 128.3, 126.4, 116.7 (t, J = 239.0 Hz), 35.7 (t, J = 21.1 Hz), 28.4 (t, J = 6.0 Hz). **^{19}F{^{1}H} NMR** (376 MHz, $CDCl_3$) δ -117.13 (s). All spectroscopic data were in accordance with the literature.[10]

1-(3,3-Difluoropropyl)-4-methylbenzene (2b)

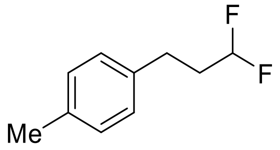

The title compound **2b** was prepared following the general procedure **B** using **1b** as starting substrate (0.4 mmol scale). Purification by column chromatography using hexane as eluent. Colourless oil, 60 mg, 90% yield. **^{1}H NMR** (400 MHz, $CDCl_3$) δ 7.16 – 7.10 (m, 4H), 5.82 (tt, J = 56.7, 4.5 Hz, 1H), 2.80 – 2.73 (m, 2H), 2.35 (s, 3H), 2.25 – 2.07 (m, 2H); **^{13}C NMR** (101 MHz, $CDCl_3$) δ 136.9, 136.1, 129.5, 128.3, 116.9 (t, J = 238.9 Hz), 35.9 (t, J = 21.0 Hz), 28.1 (t, J = 6.0 Hz), 21.1; **^{19}F{^{1}H} NMR** (376 MHz, $CDCl_3$) δ -117.08 (s). All spectroscopic data were in accordance with the literature.[11]

4-(3,3-Difluoropropyl)-1,1'-biphenyl (2c)

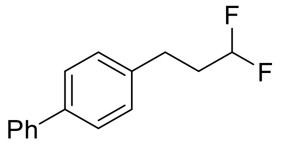

The title compound **2c** was prepared following the general procedure **A** using **1c** as starting substrate (0.4 mmol scale). Purification by column chromatography using hexane as eluent. Colourless solid, 86 mg, 92% yield. **^{1}H NMR** (500 MHz, $CDCl_3$) δ 7.64 – 7.62 (m, 2H), 7.60 – 7.58 (m, 2H), 7.50 – 7.47 (m, 2H), 7.40 – 7.37 (m, 1H), 7.31 (dd, J = 7.9, 0.6 Hz, 2H), 5.88 (tt, J = 56.7, 4.5 Hz, 1H), 2.89 – 2.85 (m, 2H), 2.28 – 2.17 (m, 2H). **^{13}C NMR** (126 MHz, $CDCl_3$) δ 140.9, 139.5, 139.1, 128.9, 128.9, 127.5, 127.3, 127.1, 116.8 (t, J = 239.1 Hz), 35.8 (t, J = 21.1 Hz), 28.1 (t, J = 6.0 Hz). **^{19}F NMR** (471 MHz, $CDCl_3$) δ -116.99 (dt, J = 56.7, 17.1 Hz). **HRMS [EI$^+$]** m/z: [M]$^+$ Calcd. for $C_{15}H_{14}F_2$ 232.1058; Found: 232.1054. All spectroscopic data were in accordance with the literature.[11]

1-Chloro-4-(3,3-difluoropropyl)benzene (2d)

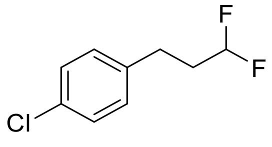

The title compound **2d** was prepared following the general procedure **B** using **1d** as starting substrate (0.4 mmol scale). Purification by column chromatography using hexane as eluent. Colourless oil, 53 mg, 70% yield. **^{1}H NMR** (400 MHz, $CDCl_3$) δ 7.28 (d, J = 8.4 Hz, 2H), 7.14 (d, J = 8.5 Hz, 2H), 5.81 (tt, J = 56.6, 4.4 Hz, 1H), 2.79 – 2.74 (m, 2H), 2.29 – 2.04 (m, 2H); **^{13}C NMR** (101 MHz, $CDCl_3$) δ 138.5, 132.3, 129.8, 128.9, 116.6 (t, J = 239.2 Hz), 35.7 (t, J = 21.2 Hz), 27.9 (t, J = 6.0 Hz); **^{19}F{^{1}H} NMR** (376 MHz, $CDCl_3$) δ -

117.16 (s). **HRMS [EI$^+$]** m/z: $[M]^+$ Calcd. for $C_9H_9{}^{35}ClF_2$ 190.0355; Found: 190.0356. All spectroscopic data were in accordance with the literature.[11]

1-(3,3-Difluoropropyl)-4-fluorobenzene (2e)

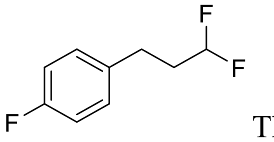

The title compound **2e** was prepared following the general procedure **B** using **1e** as starting substrate (0.4 mmol scale). Purification by column chromatography using hexane as eluent. Colourless oil, 60 mg, 86% yield. **^{1}H NMR** (400 MHz, $CDCl_3$) δ 7.24 – 7.09 (m, 2H), 7.06 – 6.93 (m, 2H), 5.80 (tt, J = 56.6, 4.5 Hz, 1H), 2.87 – 2.66 (m, 2H), 2.25 – 2.02 (m, 2H); **^{13}C NMR** (101 MHz, $CDCl_3$) δ 161.7 (d, J = 244.3 Hz), 135.7 (d, J = 3.3 Hz), 129.9 (d, J = 7.9 Hz), 116.7 (t, J = 239.1 Hz), 115.6 (d, J = 21.2 Hz), 35.9 (t, J = 20.7 Hz), 27.7 (t, J = 5.9 Hz); **^{19}F{^{1}H} NMR** (376 MHz, $CDCl_3$) δ -116.82 (s), -117.21 (s). All spectroscopic data were in accordance with the literature.[11]

1-(3,3-Difluoropropyl)-4-(trifluoromethyl)benzene (2f)

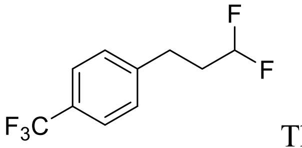

The title compound **2f** was prepared following the general procedure **B** using **1f** as starting substrate (0.4 mmol scale). Purification by column chromatography using hexane as eluent. Colourless oil, 58 mg, 65% yield. **^{1}H NMR** (400 MHz, $CDCl_3$) δ 7.57 (d, J = 8.0 Hz, 2H), 7.32 (d, J = 8.0 Hz, 2H), 5.83 (tt, J = 56.5, 4.3 Hz, 1H), 2.92 – 2.79 (m, 2H), 2.27 – 2.08 (m, 2H); **^{13}C NMR** (101 MHz, $CDCl_3$) δ 144.2, 129.0 (q, J = 32.4 Hz), 128.8, 125.8 (q, J = 3.8 Hz), 124.4 (q, J = 272.9 Hz), 116.4 (t, J = 239.4 Hz), 35.5 (t, J = 21.4 Hz), 28.3 (t, J = 6.0 Hz); **^{19}F{^{1}H} NMR** (376 MHz, $CDCl_3$) δ -62.47 (s), -117.23 (s). All spectroscopic data were in accordance with the literature.[11]

4-(3,3-Difluoropropyl)-2-iodo-1-methoxybenzene (2g)

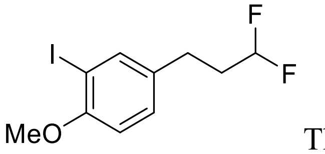

The title compound **2g** was prepared following the general procedure **B** using **1g** as starting substrate (0.4 mmol scale). Purification by column chromatography using hexane/ethyl acetate (10/1) as eluent. Colourless oil, 80 mg, 64% yield. **^{1}H NMR** (300 MHz, $CDCl_3$) δ 7.62 (d, J = 2.2 Hz, 1H), 7.13 (dd, J = 8.4, 2.2 Hz, 1H), 6.76 (d, J = 8.4 Hz, 1H), 5.79

(tt, J = 56.6, 4.4 Hz, 1H), 3.86 (s, 3H), 2.74 – 2.64 (m, 2H), 2.19 – 2.01 (m, 2H); **^{13}C NMR** (75 MHz, $CDCl_3$) δ 156.9, 139.3, 134.2, 129.5, 116.6 (t, J = 239.2 Hz), 111.1, 86.2, 56.5, 35.9 (t, J = 21.1 Hz), 27.1 (t, J = 6.0 Hz); **^{19}F NMR** (471 MHz, $CDCl_3$) δ -117.19 (dt, J = 54.7, 17.2 Hz). **HRMS [EI$^+$]** m/z: $[M]^+$ Calcd. for $C_{10}H_{11}OF_2I$, 311.9817; Found: 311.9811.

1-Bromo-2-(3,3-difluoropropyl)benzene (2h)

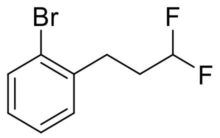

The title compound **2h** was prepared following the general procedure **B** using **1h** as starting substrate (0.4 mmol scale). Purification by column chromatography using hexane as eluent. Colourless oil, 82 mg, 87% yield. **^{1}H NMR** (400 MHz, $CDCl_3$) δ 7.61 – 7.50 (m, 1H), 7.30 – 7.21 (m, 2H), 7.17 – 7.04 (m, 1H), 5.86 (tt, J = 56.6, 4.4 Hz, 1H), 2.96 – 2.88 (m, 2H), 2.27 – 2.09 (m, 2H); **^{13}C NMR** (101 MHz, $CDCl_3$) δ 139.4, 133.2, 130.5, 128.4, 127.9, 124.4, 116.7 (t, J = 239.3 Hz), 34.1 (t, J = 21.2 Hz), 29.1 (t, J = 6.1 Hz). **^{19}F{^{1}H} NMR** (376 MHz, $CDCl_3$) δ -116.87 (s). **HRMS [EI$^+$]** m/z: $[M]^+$ Calcd. for $C_9H_9{}^{79}BrF_2$ 233.5850; Found: 233.5852.

5,5-Difluoro-1-phenylpentan-1-one (2i)

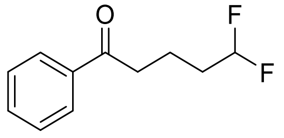

The title compound **2i** was prepared following the general procedure **B** using **1i** as starting substrate (0.4 mmol scale). Purification by column chromatography using hexane as eluent. Colourless oil, 54 mg, 68% yield. **^{1}H NMR** (400 MHz, $CDCl_3$) δ 7.96 (dd, J = 8.3, 1.2 Hz, 2H), 7.61 – 7.54 (m, 1H), 7.47 (t, J = 7.6 Hz, 2H), 5.87 (tt, J = 56.9, 4.5 Hz, 1H), 3.07 (t, J = 6.7 Hz, 2H), 2.02 – 1.86 (m, 4H); **^{13}C NMR** (101 MHz, $CDCl_3$) δ 199.3, 136.9, 133.3, 128.8, 128.1, 117.3 (t, J = 239.0 Hz), 37.6, 33.5 (t, J = 21.0 Hz), 16.80 (t, J = 5.9 Hz); **^{19}F NMR** (376 MHz, $CDCl_3$) δ -115.78 (dt, J = 34.4, 17.7 Hz); **HRMS [EI$^+$]** m/z: $[M]^+$ Calcd for $C_{11}H_{12}F_2O$, 198.0850; Found: 198.0848.

Methyl 4-(3,3-difluoropropyl)benzoate (2j)

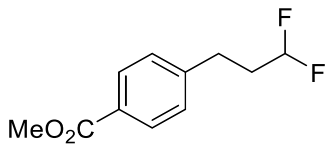

The title compound **2j** was prepared following the general procedure **B** using **1j** as starting substrate (0.4 mmol scale). Purification by column chromatography using

hexane as eluent. Colourless oil, 82 mg, 95% yield. **^{1}H NMR** (400 MHz, $CDCl_3$): δ 8.00 (d, J = 8.3 Hz, 2H), 7.29 (d, J = 8.1 Hz, 2H), 5.84 (tt, J = 56.5, 4.4 Hz, 1H), 3.93 (s, 3H), 2.96 – 2.82 (m, 2H), 2.27 – 2.10 (m, 2H); **^{13}C NMR** (101 MHz, $CDCl_3$) δ 167.0, 145.4, 130.1, 128.6, 128.5, 116.5 (t, J = 239.3 Hz), 52.2, 35.4 (t, J = 21.3 Hz), 28.5 (t, J = 5.9 Hz); **^{19}F{^{1}H} NMR** (376 MHz, $CDCl_3$) δ -117.14 (s); **HRMS [EI^{+}]** m/z: $[M]^{+\cdot}$ Calcd. for $C_{11}H_{12}O_2F_2$, 214.0799; Found: 214.0800.

(5,5-Difluoropentyl)benzene (2k)

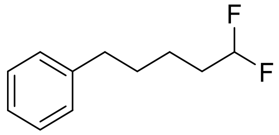

The title compound **2k** was prepared following the general procedure **B** using **1k** as starting substrate (0.4 mmol scale). Purification by column chromatography using hexane as eluent. Colourless oil, 60 mg, 81% yield. **^{1}H NMR** (400 MHz, $CDCl_3$) δ 7.31 – 7.28 (m, 2H), 7.22 – 7.17 (m, 3H), 5.80 (tt, J = 56.9, 4.5 Hz, 1H), 2.66 – 2.63 (m, 2H), 1.93 – 1.79 (m, 2H), 1.73 – 1.66 (m, 2H), 1.54 – 1.47 (m, 2H). **^{13}C NMR** (101 MHz, $CDCl_3$) δ 142.1, 128.5, 126.0, 117.5 (t, J = 238.8 Hz), 35.8, 34.1 (t, J = 20.7 Hz), 31.0, 21.9 (t, J = 5.5 Hz). **^{19}F{^{1}H} NMR** (376 MHz, $CDCl_3$) δ -115.71 (s). All spectroscopic data were in accordance with the literature.[12]

2-(3,3-Difluoropropyl)naphthalene (2l)

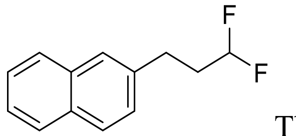

The title compound **2l** was prepared following the general procedure **B** using **1l** as starting substrate (0.4 mmol scale). Purification by column chromatography using hexane as eluent. Colourless oil, 76 mg, 92% yield. **H NMR** (400 MHz, $CDCl_3$) δ 7.81 (dt, J = 9.0, 6.6 Hz, 3H), 7.65 (s, 1H), 7.53 – 7.43 (m, 2H), 7.34 (dd, J = 8.5, 1.7 Hz, 1H), 5.85 (tt, J = 56.7, 4.5 Hz, 1H), 3.01 – 2.93 (m, 2H), 2.34 – 2.17 (m, 2H); **^{13}C NMR** (101 MHz, $CDCl_3$) δ 137.5, 133.7, 132.3, 128.5, 127.8, 127.6, 127.0, 126.7, 126.3, 125.7, 116.8 (t, J = 239.1 Hz), 35.7 (t, J = 21.1 Hz), 28.7 (t, J = 6.0 Hz); **^{19}F NMR** (376 MHz, $CDCl_3$) δ -117.06 (dt, J = 57.0, 17.1 Hz). **HRMS [EI^{+}]** m/z: $[M]^{+}$ Calcd. for $C_{13}H_{12}F_2$ 206.0901; Found: 206.0900. All spectroscopic data were in accordance with the literature.[11]

2-(2,2-Difluoroethyl)naphthalene (2m)

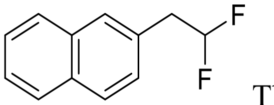

The title compound **2m** was prepared following the general procedure **B** using **1m** as starting substrate (0.4 mmol scale). Purification by column chromatography using hexane as eluent. Colourless oil, 62 mg, 81% yield. **^{1}H NMR** (400 MHz, $CDCl_3$) δ 7.93 – 7.80 (m, 3H), 7.74 (s, 1H), 7.57 – 7.45 (m, 2H), 7.38 (dd, *J* = 8.4, 1.3 Hz, 1H), 6.02 (tt, *J* = 56.6, 4.6 Hz, 1H), 3.32 (td, *J* = 17.2, 4.6 Hz, 2H); **^{13}C NMR** (101 MHz, $CDCl_3$) δ 133.5, 132.7, 130.0 (t, *J* = 5.9 Hz), 128.9, 128.5, 127.8, 127.8, 127.8, 126.5, 126.2, 116.8 (t, *J* = 241.6 Hz), 41.2 (t, *J* = 21.9 Hz); **^{19}F {^{1}H} NMR** (471 MHz, $CDCl_3$) δ -114.53 (s). All spectroscopic data were in accordance with the literature.[13]

Methyl 4-(2,2-difluoroethyl)benzoate (2n)

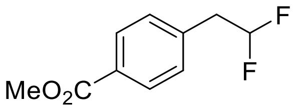

The title compound **2n** was prepared following the general procedure **B** using **1n** as starting substrate (0.4 mmol scale). Purification by column chromatography using hexane as eluent. Colourless oil, 68 mg, 85% yield. **^{1}H NMR** (400 MHz, $CDCl_3$) δ 8.00 (d, *J* = 8.3 Hz, 2H), 7.33 (d, *J* = 8.2 Hz, 2H), 5.94 (tt, *J* = 56.3, 4.5 Hz, 1H), 3.92 (s, 3H), 3.19 (td, *J* = 17.3, 4.4 Hz, 2H); **^{13}C NMR** (101 MHz, $CDCl_3$) δ 167.4, 137.9 (t, *J* = 5.7 Hz), 130.1, 130.0, 129.9, 116.1 (t, *J* = 241.7 Hz), 52.5, 40.9 (t, *J* = 22.2 Hz); **^{19}F NMR** (376 MHz, $CDCl_3$) δ -114.92 (dt, *J* = 56.7, 17.6 Hz); **HRMS [ESP]** m/z: $[M+H]^+$ Calcd. for $C_{10}H_{11}O_2F_2$ 201.0730; Found: 201.0727. All spectroscopic data were in accordance with the literature.[13]

4-(3,3-Difluoropropyl)benzoic acid (2o)

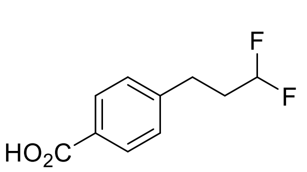

The title compound **2o** was prepared by the ester hydrolysis of **2j** following general procedure **A** with 85% yield. **^{1}H NMR** (400 MHz, $CDCl_3$) δ 8.07 (d, *J* = 8.2 Hz, 2H), 7.32 (d, *J* = 8.2 Hz, 2H), 5.84 (tt, *J* = 56.5, 4.3 Hz, 1H), 2.90 – 2.85 (m, 2H), 2.26 – 2.12 (m, 2H); **^{13}C NMR** (101 MHz, $CDCl_3$) δ 172.3, 146.6, 130.8, 128.7, 127.7, 116.5 (t, *J* = 239.4 Hz), 35.4 (t, *J* = 21.3 Hz), 28.5 (t, *J* = 5.9 Hz); **^{19}F{1H} NMR** (376 MHz, $CDCl_3$) δ -117.14 (s); **HRMS [ESN]** m/z: $[M-H]^+$ Calcd. for $C_{10}H_9F_2O_2$, 199.0571; Found: 199.0578.

.2-(6,6-difluorohexyl)isoindoline-1,3-dione (2p)

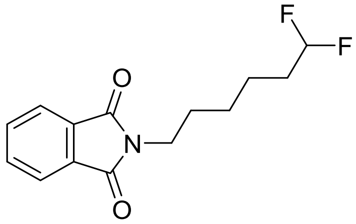

The title compound **2p** was prepared following the general procedure **B** using **1p** as starting substrate (0.4 mmol scale). Purification by column chromatography using hexane/ethyl acetate (5/1) as eluent. Colourless oil, 92 mg, 86% yield. **^{1}H NMR** (400 MHz, $CDCl_3$) δ 7.83 (dd, J = 5.5, 3.0 Hz, 2H), 7.70 (dd, J = 5.4, 3.1 Hz, 2H), 5.78 (tt, J = 56.9, 4.5 Hz, 1H), 3.68 (t, J = 7.2 Hz, 2H), 1.88 – 1.73 (m, 2H), 1.69 (dt, J = 14.8, 7.5 Hz, 2H), 1.53 – 1.35 (m, 4H); **^{13}C NMR** (101 MHz, $CDCl_3$) δ 168.5, 134.1, 132.2, 123.3, 117.3 (t, J = 238.8 Hz), 37.8, 34.0 (t, J = 20.8 Hz), 28.5, 26.4, 21.8 (t, J = 5.5 Hz). **^{19}F{^{1}H} NMR** (376 MHz, $CDCl_3$) δ -115.88 (s). **HRMS [ESP]** m/z: $[M+H]^+$ Calcd. for $C_{14}H_{16}NO_2F_2$, 268.1149; Found: 268.1144. All spectroscopic data were in accordance with the literature.[12]

***N*-(3,3-difluoropropyl)-4-methyl-N-phenylbenzenesulfonamide (2q)**

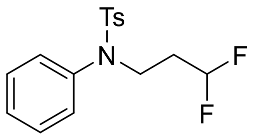

The title compound **2q** was prepared following the general procedure **B** using **1q** as starting substrate (0.2 mmol scale). Purification by column chromatography using hexane/ethyl acetate (5/1) as eluent. Colourless oil, 55 mg, 85% yield. **^{1}H NMR** (400 MHz, $CDCl_3$) δ 7.46 (d, J = 8.3 Hz, 2H), 7.36 – 7.30 (m, 3H), 7.28 – 7.22 (m, 2H), 7.08 – 7.00 (m, 2H), 5.90 (tt, J = 56.2, 4.4 Hz, 1H), 3.71 (t, J = 7.0 Hz, 2H), 2.43 (s, 3H), 2.10 – 1.97 (m, 2H). **^{13}C NMR** (101 MHz, $CDCl_3$) δ 143.9, 139.0, 134.8, 129.7, 129.4, 128.8, 128.4, 127.9, 115.6 (t, J = 239.3 Hz), 44.8 (t, J = 6.9 Hz), 33.7 (t, J = 21.6 Hz), 21.7; **^{19}F NMR** (376 MHz, $CDCl_3$) δ -117.30 (dt, J = 56.9, 17.1 Hz); **HRMS [EI$^+$]** m/z: $[M]^+$ Calcd for $C_{16}H_{17}F_2NO_2S$, 325.0942; Found: 325.0940.

4-(3,3-Difluoropropyl)-*N*-methoxy-*N*-methylbenzamide (2r)

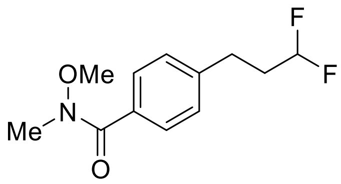

The title compound **2r** was prepared following the general procedure **B** using **1r** as starting substrate (0.4 mmol scale). Purification by column

chromatography using hexane/ethyl acetate (4/1) as eluent. Colourless oil, 76 mg, 78% yield. **^{1}H NMR** (400 MHz, $CDCl_3$) δ 7.64 (d, J = 8.2 Hz, 2H), 7.23 (d, J = 8.4 Hz, 2H), 5.81 (tt, J = 56.6, 4.4 Hz, 1H), 3.55 (s, 3H), 3.35 (s, 3H), 2.85 – 2.75 (m, 2H), 2.26 – 2.07 (m, 2H); **^{13}C NMR** (101 MHz, $CDCl_3$) δ 169.8, 142.8, 132.4, 128.8, 128.1, 116.6 (t, J = 239.2 Hz), 61.2, 35.5 (t, J = 21.2 Hz), 33.9, 28.4 (t, J = 6.0 Hz); **^{19}F NMR** (376 MHz, $CDCl_3$) δ -117.16 (dt, J = 34.1, 17.4 Hz); **HRMS [ESP]** m/z: $[M+H]^+$ Calcd. for $C_{12}H_{16}NO_2F_2$, 244.1151; Found: 244.1149.

3-(3,5-Dichlorophenyl)-5-(3,3-difluoropropyl)-5-methyloxazolidine-2,4-dione (2s)

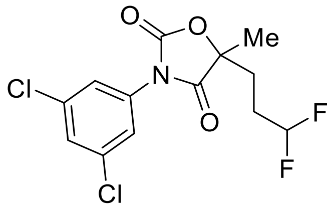

The title compound **2s** was prepared following the general procedure **B** using **1s** as starting substrate (0.2 mmol scale). Purification by column chromatography using hexane/ethyl acetate (95:5) as eluent. White solid, 60 mg, 89% yield. **^{1}H NMR** (400 MHz, $CDCl_3$) δ 7.47 – 7.41 (m, 3H), 5.90 (tt, J = 56.2, 3.8 Hz, 1H), 2.20 – 2.13 (m, 2H), 2.13 – 1.99 (m, 1H), 1.99 – 1.84 (m, 1H), 1.70 (s, 3H); **^{13}C NMR** (126 MHz, $CDCl_3$) δ 173.2, 152.2, 135.8, 132.5, 129.3, 123.8, 115.7 (t, J = 240.2 Hz), 84.9, 29.2 (t, J = 5.6 Hz), 28.1 (t, J = 22.2 Hz), 22.5; **^{19}F{^{1}H} NMR** (376 MHz, $CDCl_3$) δ -116.88 (s). **HRMS [EI$^+$]** m/z: $[M]^+$ Calcd. for $C_{13}H_{11}NO_3F_2{}^{35}Cl_2$, 337.0078; Found: 337.0071. All spectroscopic data were in accordance with the literature.[12]

2,2-Difluoro-3,4-dihydronaphthalen-1(2H)-one (2t)

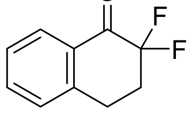

The title compound **2t** was prepared following the general procedure **B** using **1t** as starting substrate (0.4 mmol scale). Reaction time = 12 h. Purification by column chromatography using hexane/ethyl acetate (95:5) as eluent. Colourless oil, 58 mg, 80% yield. **^{1}H NMR** (400 MHz, $CDCl_3$) δ 8.10 (dd, J = 7.9, 1.1 Hz, 1H), 7.58 (td, J = 7.6, 1.4 Hz, 1H), 7.43 – 7.37 (m, 1H), 7.30 (d, J = 7.7 Hz, 1H), 3.19 (t, J = 6.3 Hz, 2H), 2.57 (tt, J = 14.6, 6.3 Hz, 2H); **^{13}C NMR** (101 MHz, $CDCl_3$) δ 185.3 (t, J = 26.0 Hz), 143.0, 135.2, 130.2, 129.1, 128.8, 127.6, 113.8 (t, J = 248.5 Hz), 32.5 (t, J = 22.9 Hz), 25.7 (t, J = 5.7 Hz); **^{19}F NMR** (376 MHz, $CDCl_3$) δ -111.13 (t, J = 14.9 Hz); **HRMS [EI$^+$]** m/z: $[M]^+$ Calcd. for $C_{10}H_8OF_2$, 182.0537; found: 182.0535. All spectroscopic data were in accordance with the literature.[14]

2,2-Difluoro-1-phenylbutan-1-one (2u)

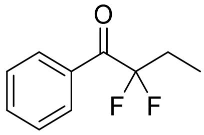

The title compound **2u** was prepared following the general procedure **B** using **1u** as starting substrate (0.4 mmol scale). Reaction time = 12 h. Purification by column chromatography using hexane/ethyl acetate (95:5) as eluent. Colourless oil, 60 mg, 81% yield. **^{1}H NMR** (400 MHz, $CDCl_3$) δ 8.11 (dd, J = 8.4, 1.0 Hz, 2H), 7.69 – 7.56 (m, 1H), 7.56 – 7.45 (m, 2H), 2.22 (tq, J = 17.7, 7.5 Hz, 2H), 1.10 (t, J = 7.5 Hz, 3H); **^{13}C NMR** (101 MHz, $CDCl_3$) δ 189.7 (t, J = 31.4 Hz), 134.4, 132.3 (t, J = 2.5 Hz), 130.3 (t, J = 3.4 Hz), 128.8, 120.1 (t, J = 252.5 Hz), 27.6 (t, J = 23.6 Hz), 5.9 (t, J = 5.6 Hz); **^{19}F{^{1}H} NMR** (376 MHz, CDCl3) δ -102.14 (s). All spectroscopic data were in accordance with the literature.[15]

(3,3-Difluorobutyl)benzene (2v)

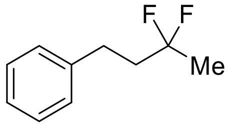

The title compound **2v** was prepared following the general procedure **C** using 4-phenylbutan-2-one as starting substrate (0.4 mmol scale). Purification by column chromatography using hexane as eluent. Colourless oil, 31 mg, 45% yield. **^{1}H NMR** (400 MHz, $CDCl_3$) δ 7.34 – 7.27 (m, 2H), 7.24 – 7.17 (m, 3H), 2.87 – 2.76 (m, 2H), 2.27 – 2.06 (m, 2H), 1.64 (t, J = 18.4 Hz, 3H); **^{13}C NMR** (101 MHz, $CDCl_3$) δ 140.8, 128.7, 128.4, 126.4, 123.9 (t, J = 238.2 Hz), 40.0 (t, J = 25.5 Hz), 29.0 (t, J = 5.0 Hz), 23.6 (t, J = 27.9 Hz); **^{19}F{^{1}H} NMR** (376 MHz, $CDCl_3$) δ -91.36 (s); **HRMS [EI^{+}]** m/z: $[M]^{+}$ Calcd. for $C_{10}H_{12}F_2$, 170.0901; found: 170.0900. All spectroscopic data were in accordance with the literature.[16]

(4,4-Difluoropiperidin-1-yl)(phenyl)methanone (2w)

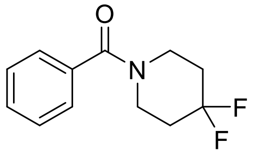

The title compound **2w** was prepared following the general procedure **D** using 1-(cyclohexa-2,4-diene-1-carbonyl)-4-fluoropiperidine-4-carboxylic acid as starting substrate (0.2 mmol scale). Purification by column chromatography using hexane/ethyl acetate (5:1) as eluent. Colourless liquid, 30 mg, 70% yield. **^{1}H NMR** (400 MHz, $CDCl_3$) δ 7.44 – 7.39 (m, 5H), 3.86 – 3.55 (m, 4H), 2.04 – 1.93 (m, 4H); **^{13}C NMR** (101 MHz, $CDCl_3$) δ 170.7,

135.4, 130.2, 128.8, 127.0, 121.7 (t, J = 242.3 Hz), 44.5, 39.2, 34.6, 34.0; **^{19}F{^{1}H} NMR** (376 MHz, $CDCl_3$) δ -97.85 (s). All spectroscopic data were in accordance with the literature.[17]

1-Chloro-4-(4,4-difluorocyclohexyl)benzene (2x)

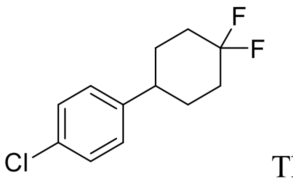

The title compound **2x** was prepared following the general procedure **B** using **1x** as starting substrate (0.2 mmol scale). Purification by column chromatography using hexane/ethyl acetate (50:1) as eluent. Colourless liquid, 35 mg, 76% yield. **^{1}H NMR** (400 MHz, $CDCl_3$) δ 7.31 – 7.23 (m, 2H), 7.19 – 7.12 (m, 2H), 2.68 – 2.46 (m, 1H), 2.28 – 2.13 (m, 2H), 2.02 – 1.69 (m, 6H); **^{19}F NMR** (376 MHz, $CDCl_3$) δ -91.77 (d, J = 236.3 Hz), -102.37 (d, J = 236.3 Hz). **HRMS [EI^{+}]** m/z: $[M]^{+\cdot}$ Calcd. for $C_{12}H_{13}{}^{35}ClF_2$, 230.0668; Found: 230.0667. All spectroscopic data were in accordance with the literature.[17]

4-(difluoromethyl)-1,1'-biphenyl (2y)

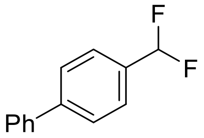

The title compound **2y** was prepared following the general procedure **D** using 2-([1,1'-biphenyl]-4-yl)-2-fluoroacetic acid as starting substrate (0.2 mmol scale). Purification by column chromatography using hexane as eluent. White solid, 36 mg, 90% yield. **^{1}H NMR** (400 MHz, $CDCl_3$) δ 7.68 (d, J = 8.3 Hz, 2H), 7.64 – 7.57 (m, 4H), 7.48 (dd, J = 10.2, 4.7 Hz, 2H), 7.40 (dd, J = 8.4, 6.3 Hz, 1H), 6.70 (t, J = 56.5 Hz, 1H); **^{13}C NMR** (101 MHz, $CDCl_3$) δ 143.9, 140.3, 133.4 (t, J = 22.4 Hz), 129.1, 128.1, 127.6, 127.4, 126.2 (t, J = 6.0 Hz), 114.9 (t, J = 238.5 Hz); **^{19}F NMR** (376 MHz, $CDCl_3$) δ -110.37 (d, J = 56.6 Hz). All spectroscopic data were in accordance with the literature.[18]

(3,3,3-Trifluoropropyl)benzene (2z)

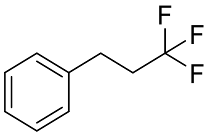

The title compound **2z** was prepared following the general procedure **B** using (3,3-difluoro-3-iodopropyl)benzene as starting substrate (0.4 mmol scale). Purification by column chromatography using hexane as eluent. Colourless liquid, 50 mg, 72% yield. **^{1}H NMR** (400 MHz, $CDCl_3$) δ 7.35 – 7.30 (m, 2H), 7.27 – 7.18 (m, 3H), 2.91 – 2.85 (m, 2H), 2.46 –

2.32 (m, 2H); ^{19}**F{^{1}H} NMR** (376 MHz, $CDCl_3$) δ -66.68 (s). All spectroscopic data were in accordance with the literature.[19]

4-(Trifluoromethyl)-1,1'-biphenyl (2aa)

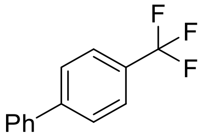

The title compound **2aa** was prepared following the general procedure **B** using **1aa** as starting substrate (0.2 mmol scale). Purification by column chromatography using hexane/ethyl acetate (50:1) as eluent. White solid, 80 mg, 90% yield. ^{1}**H NMR** (400 MHz, $CDCl_3$) δ 7.71 (s, 4H), 7.62 – 7.60 (m, 2H), 7.52 – 7.46 (m, 2H), 7.45 – 7.39 (m, 1H); ^{19}**F{^{1}H} NMR** (376 MHz, $CDCl_3$) δ -62.37 (s). All spectroscopic data were in accordance with the literature.[18]

2,2,2-Trifluoro-1-(4-methoxyphenyl)ethan-1-one (2ab)

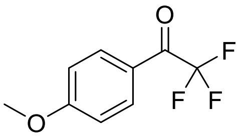

4-Methoxyphenylmagnesium bromide (1 M in THF, 0.5 mL) was added dropwise to a solution of 2,2,2-trifluoro-*N*-methoxy-*N*-methylacetamide (78 mg, 0.5 mmol) in anhydrous THF (5 mL) at 0 °C. The reaction mixture was stirred overnight at rt. The reaction mixture was quenched with sat. NH_4Cl solution (5 mL) and extracted with ethyl acetate (3 x 20 mL). The combined organic phases were washed with saturated brine (20 mL), dried over anhydrous $MgSO_4$, filtered and concentrated under reduced pressure. The residue was purified by flash column chromatography using (50:1) ethyl acetate/petroleum ether (40-60 °C)) to afford product **2ab**. Colourless liquid, 70 mg, 68% yield; ^{1}**H NMR** (400 MHz, $CDCl_3$) δ 8.07 (d, J = 9.0, 2H), 7.02 (d, J = 9.0 Hz, 2H), 3.93 (s, 3H); ^{13}**C NMR** (101 MHz, $CDCl_3$) δ 179.0 (q, J = 34.5 Hz), 165.6, 132.9 (q, J = 2.5 Hz), 122.9 (s), 117.1 (d, J = 291.4 Hz), 114.6, 55.8; ^{19}**F{^{1}H} NMR** (376 MHz, $CDCl_3$) δ -71.03 (s). All spectroscopic data were in accordance with the literature.[20]

(3-Fluorobutyl)benzene (5a)

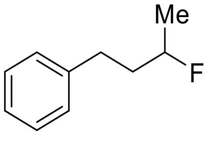

The title compound **5a** was prepared following the general procedure **C** using 4-phenylbutan-2-ol as starting substrate (0.4 mmol scale). Purification by column

chromatography using hexane as eluent. Colourless oil, 31 mg, 45% yield. (^{1}H NMR, 400 MHz, $CDCl_3$): δ 7.32 - 7.19 (m, 5H), 4.77 – 4.57 (m, 1H), 2.85 – 2.66 (m, 2H), 2.20 – 1.74 (m, 2H), 1.35 (dd, J = 23.9, 6.2 Hz, 3H); **^{19}F{^{1}H} NMR** (376 MHz, $CDCl_3$) δ -174.28 (s). All spectroscopic data were in accordance with the literature.[21]

Radiochemistry

General information

For non-automated radiochemistry experiments: [^{18}F]Fluoride was produced in an IBA Cyclon 18/9 cyclotron using the ^{18}O(p,n)^{18}F reaction. Azeotropic drying was performed on a AllinOne radiosynthesiser (Trasis, Belgium). Radio-thin layer chromatography (radio-TLC) were performed on Merck aluminium plates coated with silica (Silica gel 60 F254) and analysed with a Scan-RAM radio-TLC (LabLogic, UK) scanner equipped with a NaI/PMT-radiodetectors. Radio high-performance liquid chromatography (radio-HPLC) (Agilent 1200) were performed on an equipped with a gamma-RAM Model 4 detector (LabLogic UK). Reactions were preformed in Wheaton V-vials (3 mL) with open-top cap and septa purchased from Merck Life Sciences (Part number Z115142).

For automated radiochemistry experiments: [^{18}F]Fluoride was produced in an IBA Cyclon 18/9 cyclotron using the ^{18}O(p,n)^{18}F reaction. All experiments were performed on a AllinOne radiosynthesiser (Trasis, Belgium). Semi-preparative purification of radiolabeled products was achieved using the integrated HPLC system (including UV and radio detectors) of the radiosynthesiser. Radio-thin layer chromatography (radio-TLC) were performed using Merck aluminium plates coated with silica (Silica gel 60 F254) and analysed with a Scan-RAM radio-TLC scanner equipped with a NaI/PMT-radiodetectors (LabLogic, UK). Radio high-performance liquid chromatography (radio-HPLC) (Agilent 1200) were performed on an equipped with a gamma-RAM Model 4 detector (Lablogic, UK).

HPLC conditions

HPLC systems: Analytical HPLC runs were performed on an Agilent 1200 equipped with UV detector and LabLogic gamma-RAM Model 4 detector. Semi-preparative purification of radiolabeled products was achieved using the integrated HPLC system (including UV and radio detectors) of the AllinOne radiosynthesiser (Trasis, Belgium).

Conditions A: Analytical

Flow rate = 1.0 mL/min, temperature = 25 °C, Wavelength = 254nm (unless otherwise specified); column: Agilent Zorbax SB-C18 5 µm 4.6 x 250 mm LC column; HPLC gradient: H_2O/MeCN, 0-1 min (5% MeCN) isocratic, 1-10 min (5% MeCN to 95% MeCN) linear increase, 10-14 min (95% MeCN) isocratic, 14-15 min (95% MeCN to 5% MeCN) linear decrease, 15-17 min (5% MeCN) isocratic.

Conditions B: Semi-preparative purification

Flow rate = 4.0 mL/min; temperature = room temperature; wavelength = 254 nm; column: Phenomenex Luna 5 µm C18(2) 100 Å 10 x 250 mm LC column; isocratic: H2O/MeCN (35:65).

<u>Optimization of manual radiochemistry experiments:</u>

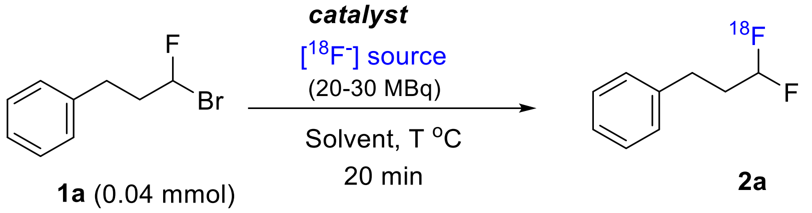

Entry	Catalyst (equiv.)	Solvent[a]	Additives (mol%)	^{18}F- source	Temp (°C)	RCC (%)
1	NIS (0.5)	DCE	-	$[^{18}F]KF/K_{2.2.2}$	60	0 (n=6)
2	NIS (0.5)	DCM	-	$[^{18}F]KF/K_{2.2.2}$	45	0 (n=2)
3	NIS (0.5)	DMF	-	$[^{18}F]KF/K_{2.2.2}$	60	0 (n=2)
4	NIS (0.5)	MeCN	-	$[^{18}F]KF/K_{2.2.2}$	60	0 (n=2)
5	$In(OTf)_3$ (0.2)	DCE	-	$[^{18}F]KF/K_{2.2.2}$	60	0 (n=2)
6	$Ga(OTf)_3$ (0.2)	DCE	-	$[^{18}F]KF/K_{2.2.2}$	60	0 (n=2)
7	$In(OTf)_3$ (0.2)	DCE	$NaBAr_4^F$ (0.2)	$[^{18}F]KF/K_{2.2.2}$	60	0 (n=2)
8	$Ga(OTf)_3$ (0.2)	DCE	$NaBAr_4^F$ (0.2)	$[^{18}F]KF/K_{2.2.2}$	60	0 (n=2)
9	AgOTf (0.2)	DCE	-	$[^{18}F]KF/K_{2.2.2}$	60	0 (n=2)
10	AgOTf (1.0)	DCE	-	$[^{18}F]KF/K_{2.2.2}$	60	0 (n=2)
11	AgOTf (0.2)	DCE	KF (0.12)	$[^{18}F]KF/K_{2.2.2}$	60	<1(n=2)
12	AgOTf (1.0)	DCE	-	$[^{18}F]KF/K_{2.2.2}$	60	0 (n=4)
13	AgOTf (1.0)	DCE	-	$[^{18}F]KF/K_{2.2.2}$	80	0 (n=2)
14	AgOTf (1.0)	DCM	-	$[^{18}F]KF/K_{2.2.2}$	25	0 (n=2)
15	$AgBF_4$ (1)	DCE	-	$[^{18}F]KF/K_{2.2.2}$	80	0 (n=3)
16	$AgPF_6$ (1)	DCE	-	$[^{18}F]KF/K_{2.2.2}$	80	0 (n=3)
17	$AgBF_4$ (1)	DCE		$[^{18}F]KF/K_{2.2.2}$	80	0 (n=3)
18	AgF (1.0)	DCE	-	$[^{18}F]KF/K_{2.2.2}$	60	2 (n=2)
19	AgF (1.0)	MeCN	-	$[^{18}F]KF/K_{2.2.2}$	60	6±1 (n=2)
20[b]	Ag_2O (1)	DCE		$[^{18}F]KF/K_{2.2.2}$	80	25 ± 7 % (n=3)
21[b]	Ag_2O (1)	MeCN	-	$[^{18}F]KF/K_{2.2.2}$	80	35 ± 11 % (n=3)
22[b]	Ag_2O (1)	*t*-BuOH	-	$[^{18}F]KF/K_{2.2.2}$	80	68 ± 12 % (n=3)
23[b]	Ag_2O (0.5)	*t*-BuOH	-	$[^{18}F]KF/K_{2.2.2}$	80	55 ± 14 % (n=3)
24	Ag_2O (0.5)	*t*-BuOH	-	$[^{18}F]KF/K_{2.2.2}$	80	47 ± 1 % (n=3)
25	Ag_2O (1.0)	DMF	-	$[^{18}F]KF/K_{2.2.2}$	100	25±4 (n=3)
26	Ag_2O (1.0)	Toluene	-	$[^{18}F^-]KF/K_{2.2.2}$	100	13±1 (n=2)
27	Ag_2O (1.0)	THF	-	$[^{18}F^-]KF/K_{2.2.2}$	100	2 (n=1)
28	CuO (0.5)	^{t}BuOH	-	$[^{18}F]KF/K_{2.2.2}$	80	6±2 (n=2)
29	-	MeCN	-	$[^{18}F]AgF$	90	2 (n=2)
30	-	MeCN	-	$[^{18}F]AgF$	115	8 (n=1)
31	-	MeCN	-	$[^{18}F]KF/K_{2.2.2}$	80	0 (n=3)
32	-	^{t}BuOH	KF (3.0)	$[^{18}F]TEAF$	100	14 (n=2)
33	-	^{t}AmOH	KF (3.0)	$[^{18}F]TEAF$	115	71 (n=1)
34	-	^{t}AmOH	-	$[^{18}F]TEAF$	100	4 (n=4)
35	-	^{t}BuOH	-	$[^{18}F]TEAF$	100	2 (n=1)

a) volume 300 µl; b) volume 150 µl.

Non-automated radiofluorination of 1-bromo-1-fluoroalkane substrates

[^{18}F]KF elution and drying: [^{18}F]Fluoride was separated from ^{18}O-enriched-water using an anion exchange cartridge (Waters Sep-Pak AccellPlus QMA Carbonate Plus Light Cartridge), activated with MeOH (1 mL) followed by H_2O (10 mL) prior to use and released with a solution of Kryptofix (15 mg), and K_2CO_3 (3 mg) in MeCN/H_2O (1 mL, 4:1, v/v), which was concentrated over a period of 20 min by azeotropic drying using dry MeCN (3 x 0.7 mL) under a flow of N_2 at 110 °C.[22]

^{18}F-fluorination: After azeotropic drying, the aliquots of [^{18}F]KF (20-35 MBq in MeCN) were dispensed into an oven-dried 3 mL Wheaton V-vial, containing Ag_2O (4.6 mg, 0.02 mmol) and a magnetic stirrer bar. Then, MeCN was subsequently removed by drying under a flow of N_2 at 80 °C. Substrate (0.04 mmol) in *t*-BuOH (300 µL) was then added to the 3 mL Wheaton V-vial by syringe. The reaction was heated to 80 °C for 20 minutes. The reaction mixture was then quenched with MeCN/H_2O (200 µL, 1:1 v/v). An aliquot was taken for analysis by radio-TLC and radio-HPLC to calculate the radiochemical conversion.

Radiochemical conversion (RCC) = radiochemical conversion by radio-TLC (*r*-TLC) **X** radiochemical purity by radio-HPLC (*r*-HPLC)

The radiochemical conversion by radio-TLC was determined by integration of the unreacted [^{18}F]fluoride to the total peak area for all other radioactive species. As radio-TLC is not precise enough able to distinguish between multiple radiolabeled organic compounds with similar retention values, we multiply the radio-TLC yield by the radiochemical purity (RCP) measured by radio-HPLC. When determining the RCP by radio-HPLC the eluted [^{18}F]fluoride is not considered as this is more accurately measured by radio-TLC. The reactions were repeated n times. Radio-HPLC analysis performed with HPLC gradient A.

Automated radiosynthesis

General procedure: The automated radiosynthesis of [^{18}F]**2e** and [^{18}F]**2u** was performed with a AllInOne synthesiser (Trasis, Belgium) using an automated programme and pre-built cassette (Figure S1). The vial in slot 2 was charged with Kryptofix® 222 (7.5 mg) and K_2CO_3 (1.5 mg), dissolved in MeCN (600 μL) and H_2O (150 μL). The anion exchange cartridge at slot 5 (Waters Sep-Pak AccellPlus QMA Carbonate Plus Light Cartridge) was activated with MeOH (1 mL), followed by H_2O (10 mL) prior to use. The solvent reservoirs, slots 8, 12 and 16 were charged with anhydrous MeCN (2 mL), anhydrous *t*-BuOH (2 mL) and MeCN: H_2O (1:1 v/v, 5 mL) respectively. The right reactor at slot 10 was charged with Ag_2O (4.6 mg, 0.02 mmol). The syringe at slot 11 was charged with the 1-bromo-1-fluoroalkane (0.04 mmol) in anhydrous *t*-BuOH (300 μL). The PTFE syringe filter (Fisherbrand, 0.2 μm) at slot 14 was activated with MeCN: H_2O (1:1 v/v, 5 mL) prior to use.

After [^{18}F]fluoride in [^{18}O]water was received from the cyclotron, it was trapped on a Waters Sep-Pak AccellPlus QMA Carbonate Plus Light cartridge. [^{18}F]Fluoride was then eluted from the cartridge with a solution of Kryptofix® and K_2CO_3, into the left reactor (slot 7). The [^{18}F]fluoride was then dried using a combination of a flow of N_2 and vacuum, whilst heating at 120 °C for 14 minutes. Once the drying was complete, anhydrous MeCN (0.8 mL) was added to the [^{18}F]fluoride in the left reactor, and the mixture was transferred to the right reactor (slot 10) which contained the Ag_2O. The right reactor was then heated at 80 °C for 2 minutes, under a flow of N_2 to remove the MeCN and re-dry the [^{18}F]fluoride.[22] Once complete, the syringe containing the 1-bromo-1-fluoroalkane (0.04 mmol) in anhydrous *t*-BuOH (300 μL) (slot 11) was added to the right reactor, followed by additional anhydrous *t*-BuOH (200 μL). The ^{18}F-fluorination reaction was run at 80 °C for 20 minutes. The reactor was cooled to 50 °C and the mixture was quenched with MeCN: H_2O (1:1 v/v, 2.5 mL). The mixture was taken up in a 3 mL syringe (slot 11) and transferred back to the reactor for mixing. This step was repeated twice. The reaction mixture was finally taken up in a 3 mL syringe (slot 11) and pushed through the PTFE syringe filter (Fisherbrand, 0.2 μm) via slot 13, and subsequently into the HPLC sample loop (slot 15). The right reactor was then washed further with a solution of MeCN: H_2O (1:1 v/v, 2.5 mL). The mixture was mixed using the 3 mL syringe (slot 11) three times. The mixture was then pushed through the PTFE syringe filter (Fisherbrand, 0.2 μm) via slot 13, and subsequently into the HPLC sample loop (slot 15). The crude mixture was purified by reverse-phase semi-preparative HPLC (MeCN/ H_2O as eluent, Q = 4 mL/min, HPLC conditions B).

The isolated product was collected into a vial and the activity of the product was measured in a dose calibrator.

An aliquot (20-80 MBq) of the isolated product was analysed by radio-HPLC to determine molar activity. HPLC analysis was performed under HPLC conditions A. The molar activity was determined on a chemically and radiochemically pure sample. The isolated product was injected, with the injected activity and time of injection recorded. The UV response corresponding to the desired radiofluorinated product was then integrated, to give the amount of non-radioactive product that was detected. This process was repeated to allow an average molar activity to be calculated.

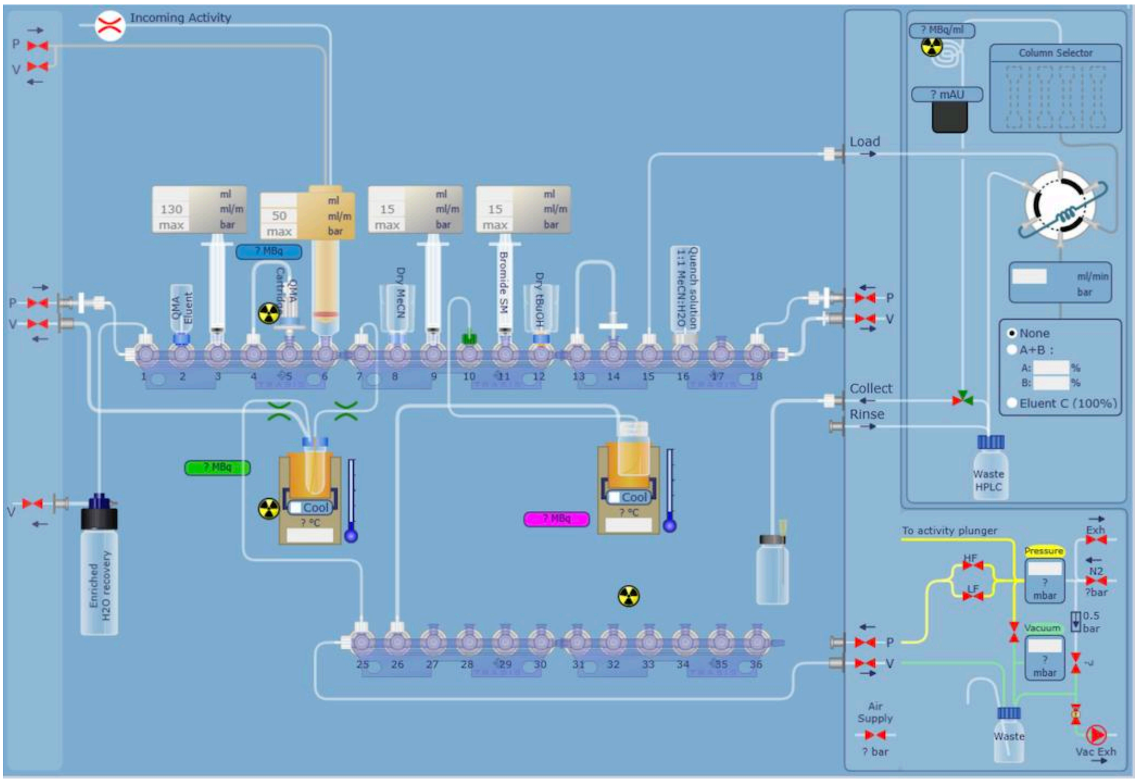

Figure S1: Cassette set-up for the automated radiofluorination of 1-bromo-1-fluoroalkanes using a AllInOne automated platform (Trasis, Belgium).

Automated radiosynthesis of 1-(3,3-[^{18}F]difluoropropyl)-4-fluorobenzene ([^{18}F]2e)

Procedure for radiosynthesis of [^{18}F]2e: The general procedure was followed using 1-(3-bromo-3-fluoropropyl)-4-fluorobenzene (**1e**) (9.4 mg, 0.04 mmol). HPLC purification was carried out using MeCN/H_2O = 65/35, v/v, as eluent, t_R([^{18}F]**2e**) = 10 min. Synthesis data are summarised below (Table S1).

Table S1. Radiosynthesis of **[^{18}F]2e**

Starting activity	AY	Synthesis time	RCP	A_m
43.5 GBq	11.6 GBq	65 min	>99%	11.2 GBq/µmol

Procedure for the calibration curve of 2e: A calibration curve for authentic reference **2e** was recorded by injecting 5 different volumes onto the HPLC of the solution **2e** (1.1 mg) in MeCN (1.5 mL). Using HPLC conditions A, the UV response was measured by integrating the peak of interest. The molar activity was then calculated, the data are summarized in Table S2.

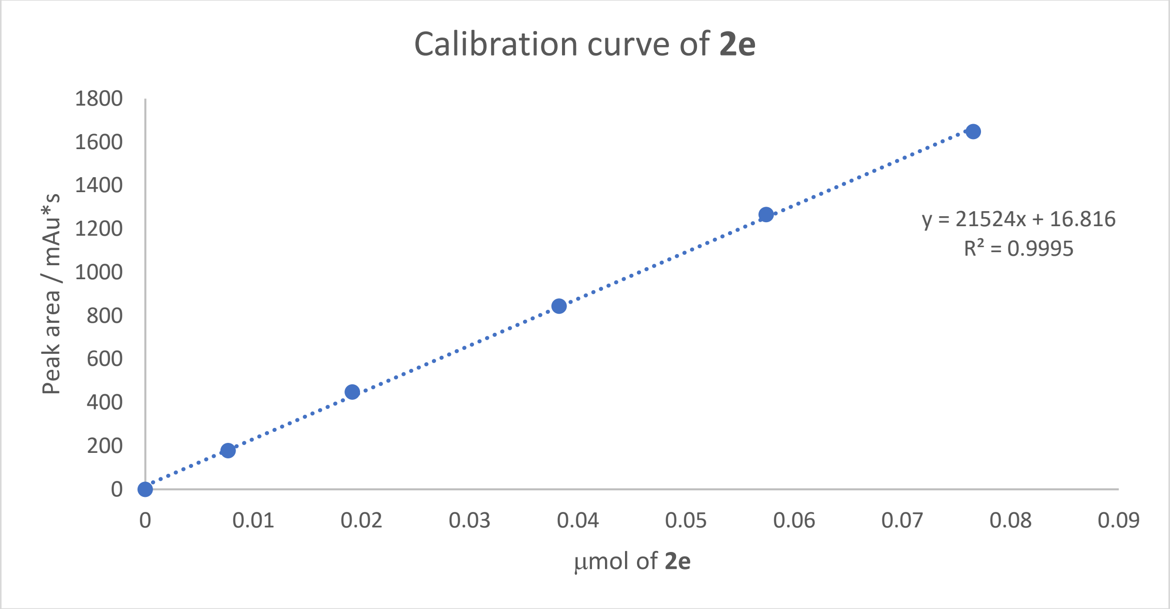

Figure S2: HPLC calibration curve for authentic reference **2e**.

Table S2. Molar activity calculation of [^{18}F]**2e**.

Measurement	Activity injected (MBq, d.c.)	Peak area (mAu*s)	2e injected (µmol)	A_m (GBq/µmol)
1	2.54	6.3	2.93×10^{-4}	8.7
2	0.86	1.3	6.10×10^{-5}	14.2
3	2.16	4.2	1.97×10^{-4}	11.1
			**Average**	**11.3 ± 2.3**

Preparative RadioHPLC trace for [^{18}F]2e

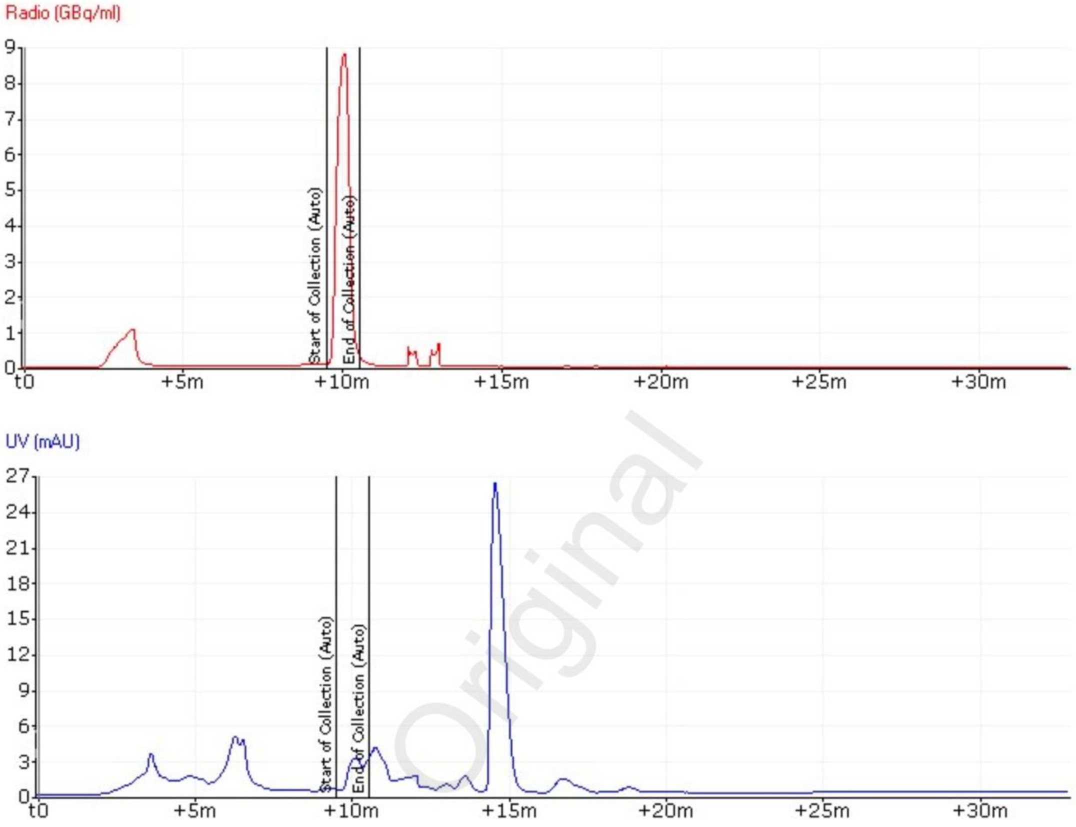

The automated radiosynthesis of [^{18}F]**2e** was also performed with less radioactivity, using the same procedure. The molar activity was calculated, these data are summarised below.

Table S3. Radiosynthesis of **[^{18}F]2e**

**Starting activity**	**AY**	**Synthesis time**	**RCP**	A_m
18.1 GBq	4.8 GBq	65 min	>99%	8.1 GBq/µmol

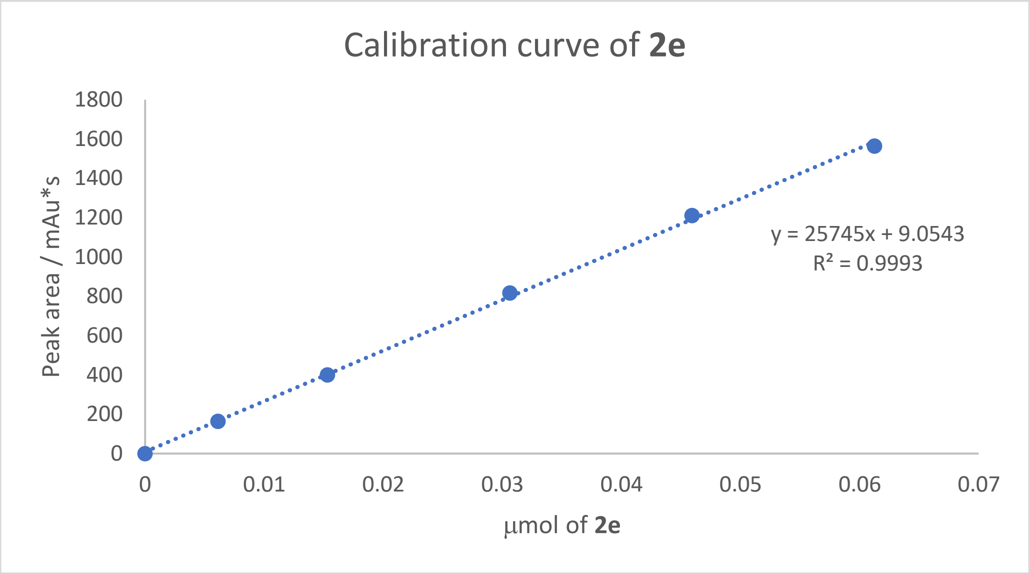

Figure S3: HPLC calibration curve for authentic reference **2e**.

Table S4. Molar activity calculation of [^{18}F]**2e**.

Measurement	Activity injected (MBq, d.c.)	Peak area (mAu*s)	2e injected (µmol)	A_m (GBq/µmol)
1	0.43	1.2	4.66 x 10^{-5}	9.2
2	0.70	2.6	1.01 x 10^{-4}	6.9
			**Average**	**8.1 ± 1.2**

Silver content from automated radiosynthesis of [^{18}F]2e

Quantofix silver test strips. The isolated [^{18}F]**2e** product was allowed to decay and then a semi-quantitative assessment of residual silver content was primarily done using test strips specific to silver (I). The silver test strip was dipped into a small volume of the sample and the excess liquid was shaken off, after 15 seconds the colour was compared against the colour scale (Figure S4).

Figure S4. Semi-quantitative assessment of the residual silver content of purified [^{18}F]**2e** using Quantofix silver test strips.

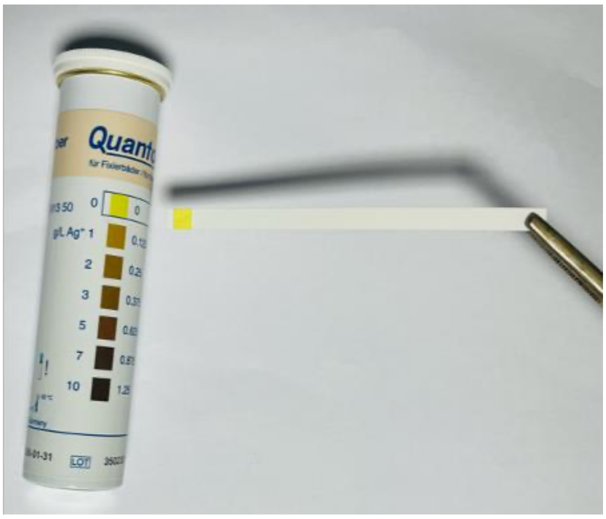

ICP-MS analysis of [^{18}F]2e: The isolated [^{18}F]**2e** product was allowed to decay and then analysed by ICP-MS to determine silver content. The silver content was determined by comparison to a standard curve generated by a series of dilutions of a known silver concentration. From this method the average quantity of ^{107}Ag was determined to be 4372.57 μg/L (4 ppm).

Table S5. ICP-MS analysis of decayed [^{18}F]**2e** to determine the silver content of the sample.

**Measurement**	**Conc. [μg/L]**
1	4360.67
2	4369.62
3	4387.41
**Average**	**4372.57**

Automated radiosynthesis of 2,2-[^{18}F]difluoro-1-phenylbutan-1-one [^{18}F]2u

Procedure for radiosynthesis of [^{18}F]2u: The general procedure was followed using 2-bromo-2-fluoro-1-phenylbutan-1-one (**1u**) (9.8 mg, 0.04 mmol). HPLC purification was carried out using MeCN/H_2O = 65/35, v/v, as eluent, t_R([^{18}F]**2u**) = 11 min. Synthesis data are summarised below (Table S6).

Table S6. Radiosynthesis of **[^{18}F]2u.**

Starting activity	AY	Synthesis time	RCP	A_m
49.3 GBq	3.3 GBq	65 min	>93%	2.4 GBq/µmol

Procedure for the calibration curve of 2u. A calibration curve for authentic reference **2u** was recorded by preparing samples of a range of concentrations by serial dilution, starting with a solution of **2u** (0.7 mg) in MeCN (1.0 mL). Using HPLC conditions A, the UV response was measured by integrating the peak of interest. Molar activity was then calculated. These data are summarized in Table S7.

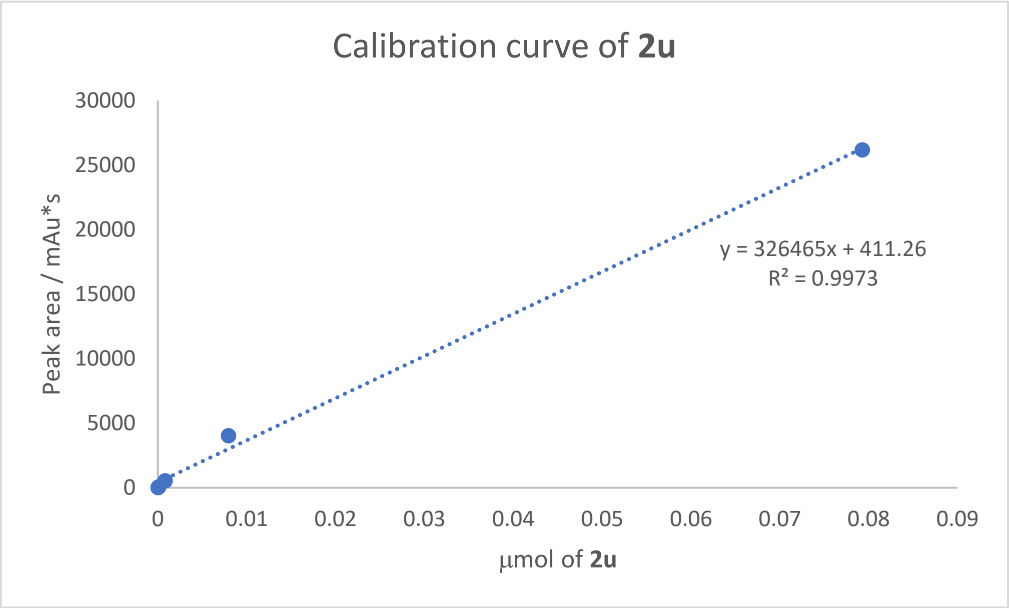

Figure S6: HPLC calibration curve for authentic reference **2**.

Table S7. Molar activity calculation of [^{18}F]**2u**.

Measurement	Activity injected (MBq, d.c.)	Peak area (mAu*s)	2u injected (µmol)	A_m (GBq/µmol)

1	3.03	488.5	1.50 x 10^{-3}	2.0
2	1.66	225.6	6.91 x 10^{-4}	2.4
3	1.08	132.9	4.07 x 10^{-4}	2.7
			**Average**	**2.4 ± 0.3**

Preparative RadioHPLC trace for [^{18}F]2u

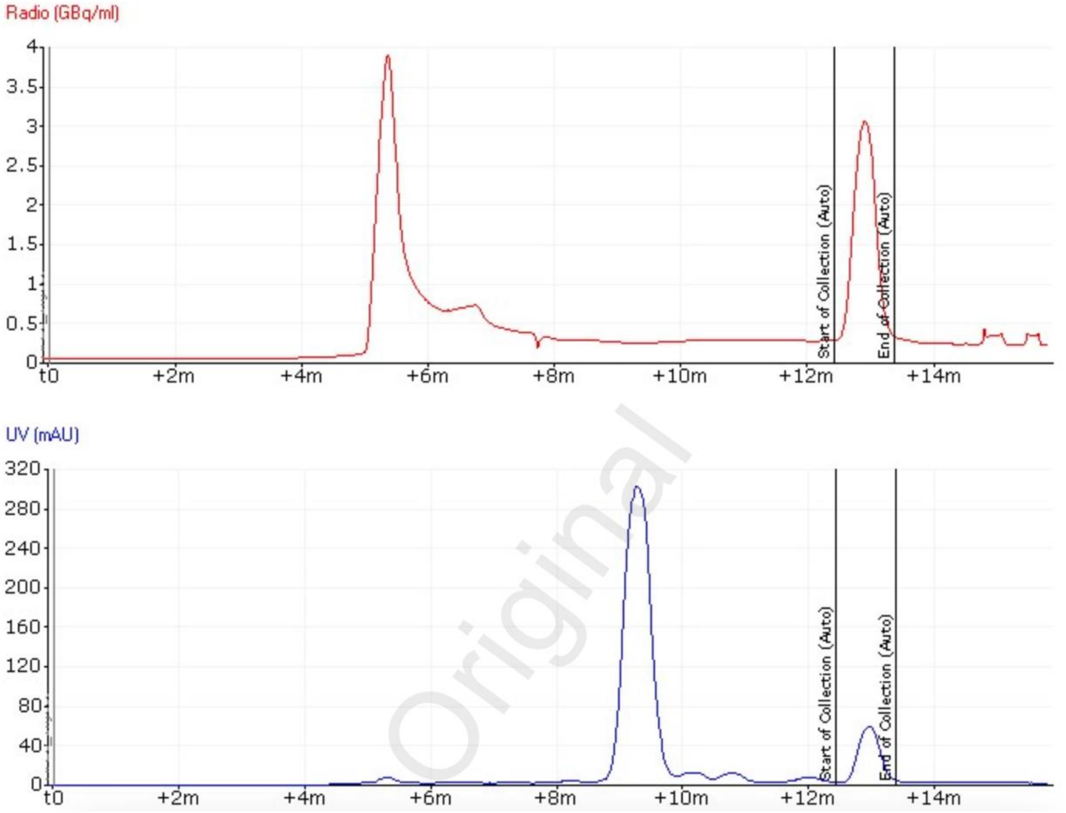

RadioHPLC overlays and radiochemical conversions

Radiochemical conversion (RCC) = radiochemical conversion by radio-TLC (**RCY**) **X** radiochemical purity by radio-HPLC (**RCP**)

UV/radio-HPLC trace overlay for [^{18}F] (3,3-difluoropropyl)benzene ([^{18}F]2a)

Prepared following the general procedure and analysed by (radio)HPLC using conditions A.

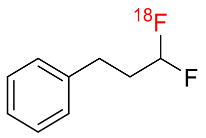

Run	RCY (%) (rTLC)	RCP (%) (rHPLC)	RCC (%)
1	48%	100%	48%
2	46%	100%	46%
3	48%	100%	48%
	**Average RCC**	**47% ± 1% (n=3)**	

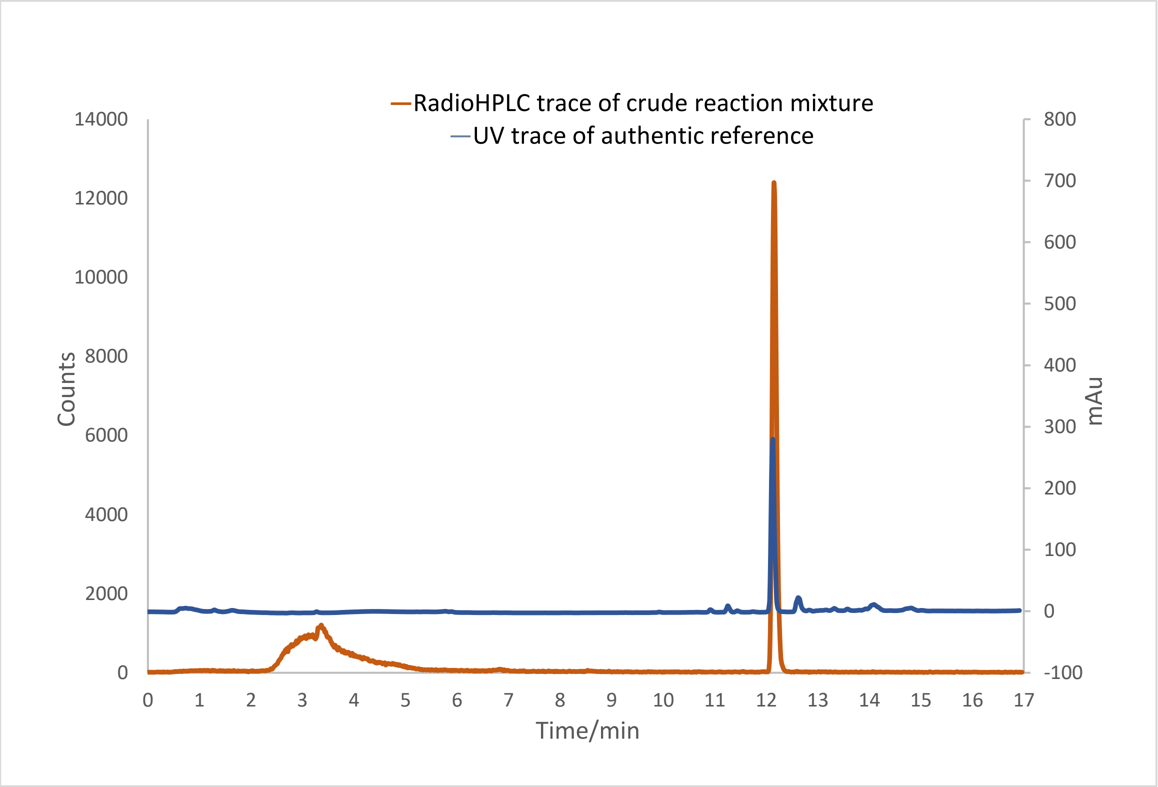

UV/radio-HPLC trace overlay for [^{18}F] 1-(3,3-difluoropropyl)-4-methylbenzene ([^{18}F]2b)

Prepared following the general procedure and analysed by (radio)HPLC using conditions A.

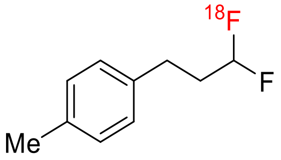

Run	RCY (%) (rTLC)	RCP (%) (rHPLC)	RCC (%)
1	62	100	62
2	63	100	63
3	70	100	70
	**Average RCC**	**65% ± 4% (n=3**	

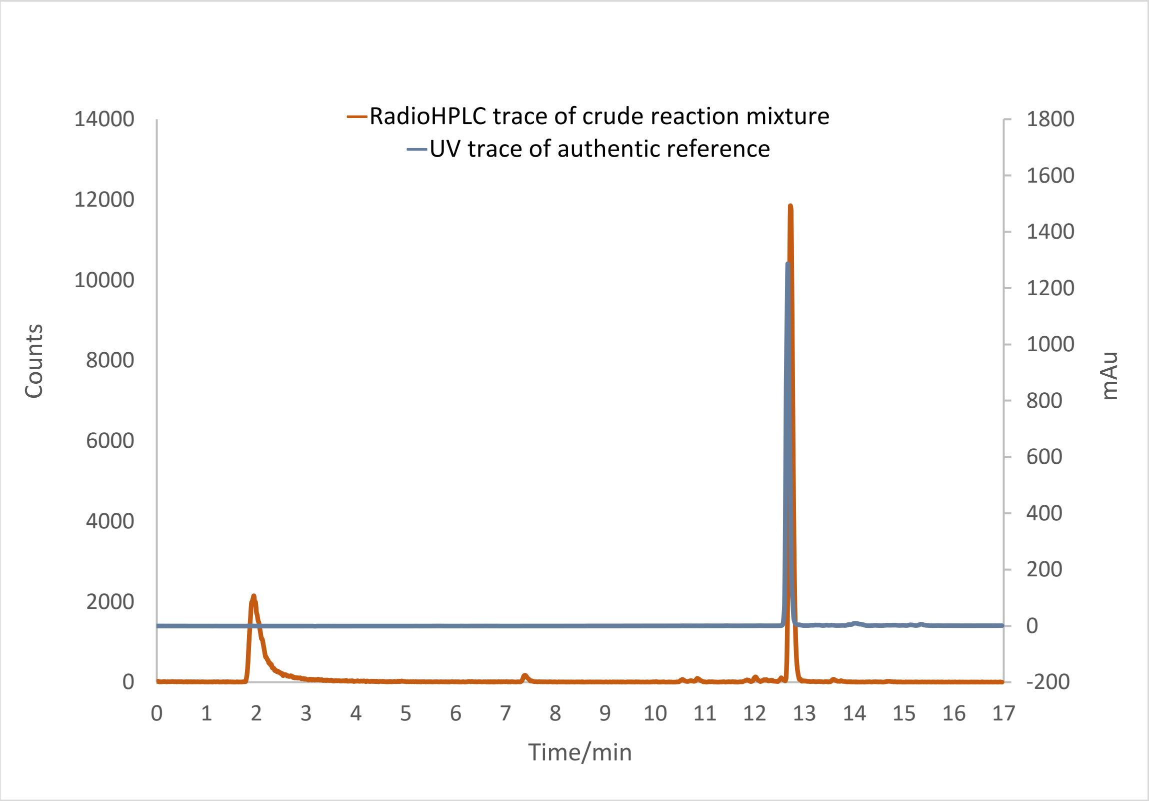

UV/radio-HPLC trace overlay for [^{18}F] 4-(3,3-difluoropropyl)-1,1'-biphenyl ([^{18}F]2c)

Prepared following the general procedure and analysed by (radio)HPLC using conditions A.

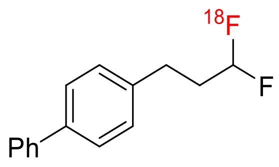

Run	RCY (%) (rTLC)	RCP (%) (rHPLC)	RCC (%)
1	78	100	78
2	77	100	77
3	56	100	56
	**Average RCC**	**70% ± 10% (n=3)**	

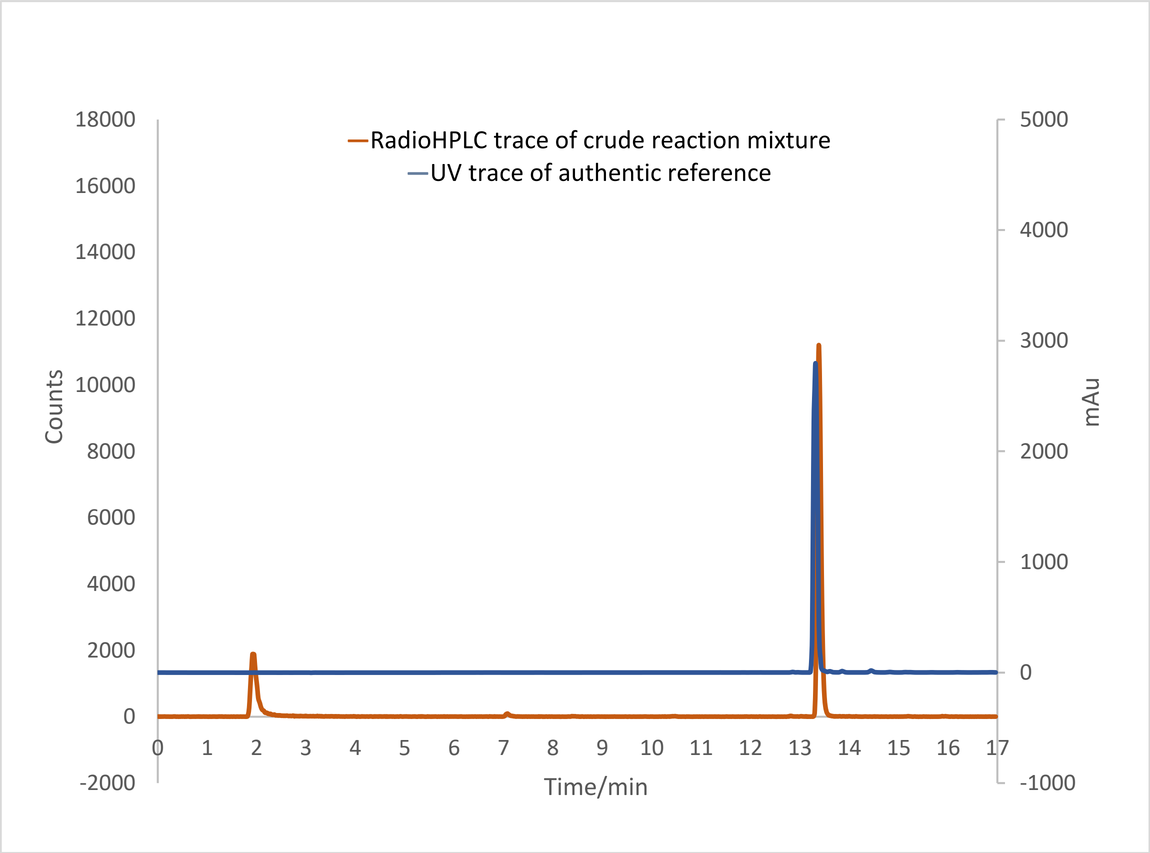

UV/radio-HPLC trace overlay for [^{18}F] 1-chloro-4-(3,3-difluoropropyl)benzene ([^{18}F]2d)

Prepared following the general procedure and analysed by (radio)HPLC using conditions A.

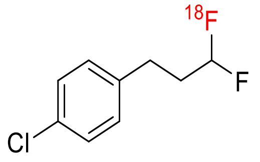

Run	RCY (%) (rTLC)	RCP (%) (rHPLC)	RCC (%)
1	30	100	30
2	31	100	31
3	55	100	55
Average RCC		39% ± 11% (n=3)	

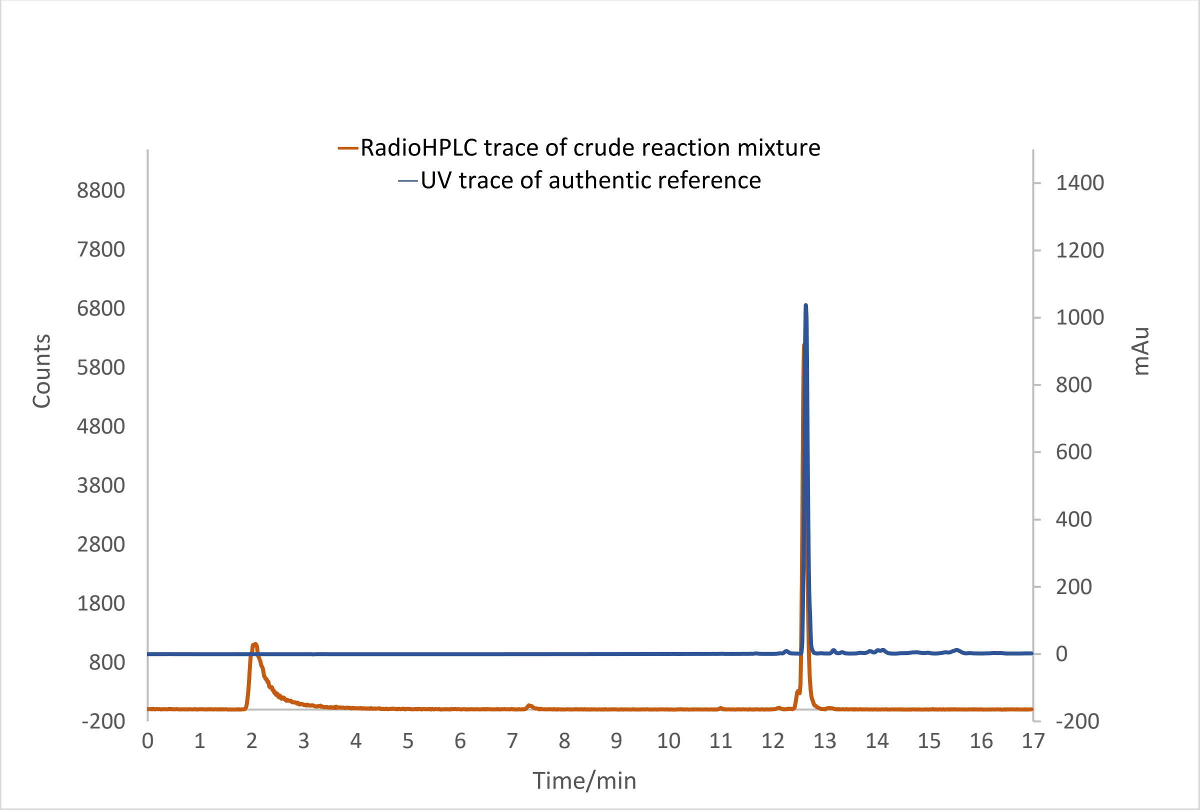

UV/radio-HPLC trace overlay for [^{18}F] 1-(3,3-difluoropropyl)-4-fluorobenzene ([^{18}F]2e)

Prepared following the general procedure and analysed by (radio)HPLC using conditions A.

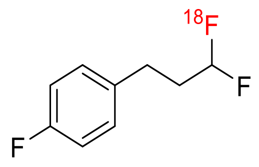

Run	RCY (%) (rTLC)	RCP (%) (rHPLC)	RCC (%)
1	69	100	69
2	65	100	65
3	54	100	54
Average RCC		63% ± 6% (n=3)	

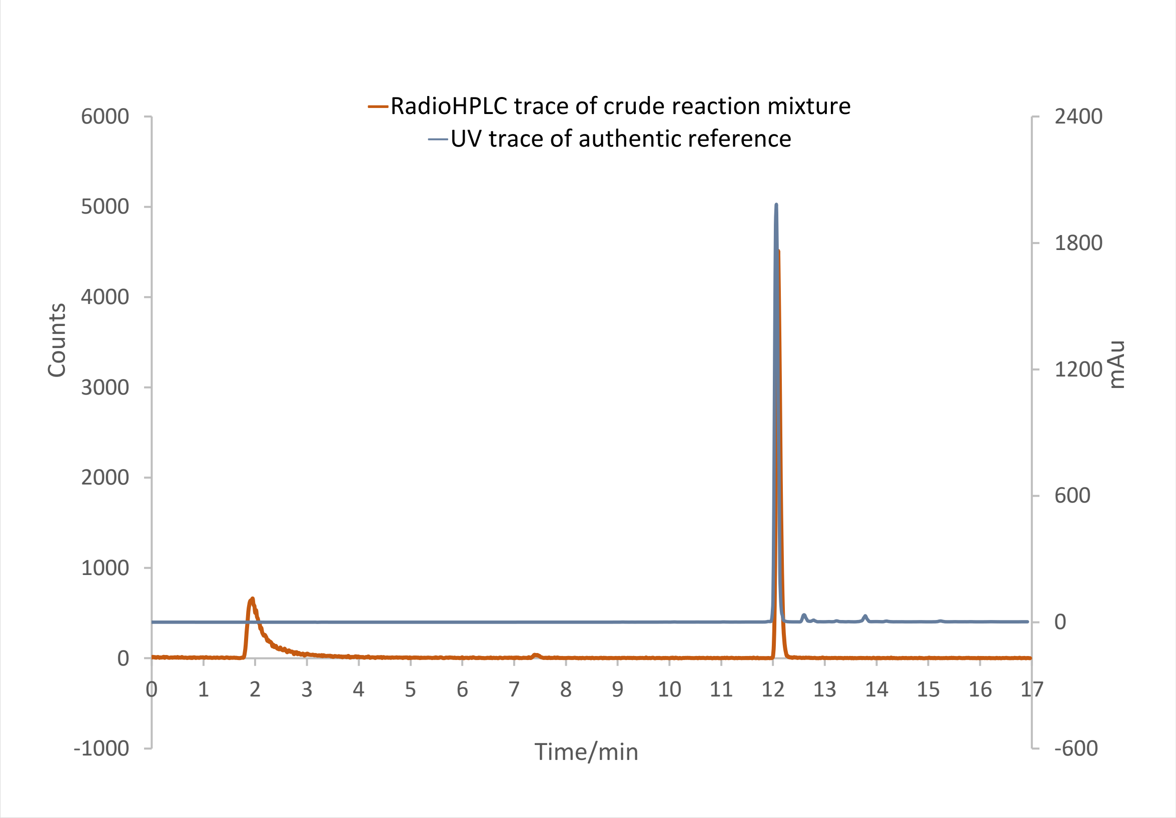

UV/radio-HPLC trace overlay for [^{18}F] 1-(3,3-difluoropropyl)-4-(trifluoromethyl) benzene ([^{18}F]2f)

Prepared following the general procedure and analysed by (radio)HPLC using conditions A.

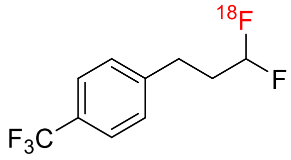

Run	RCY (%) (rTLC)	RCP (%) (rHPLC)	RCC (%)
1	41	100	41
2	40	100	40
3	67	100	67
	**Average RCC**	**49% ± 12% (n=3)**	

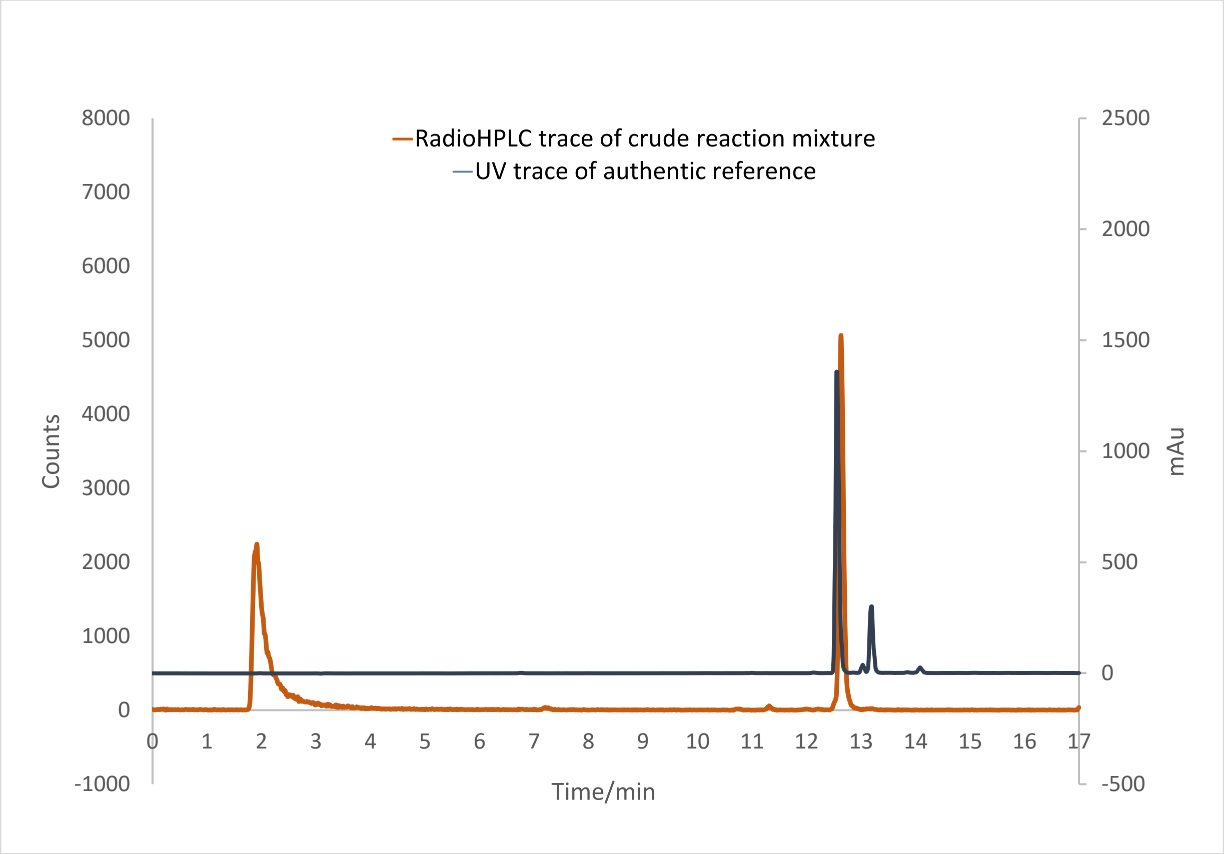

UV/radio-HPLC trace overlay for [^{18}F] 4-(3,3-difluoropropyl)-2-iodo-1-methoxybenzene ([^{18}F]2g)

Prepared following the general procedure and analysed by (radio)HPLC using conditions A.

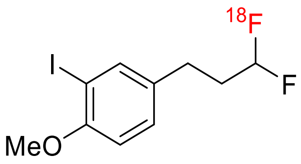

Run	RCY (%) (rTLC)	RCP (%) (rHPLC)	RCC (%)
1	76	69	52
2	69	68	47
3	81	36	29
	**Average RCC**	**43% ± 10% (n=3)**	

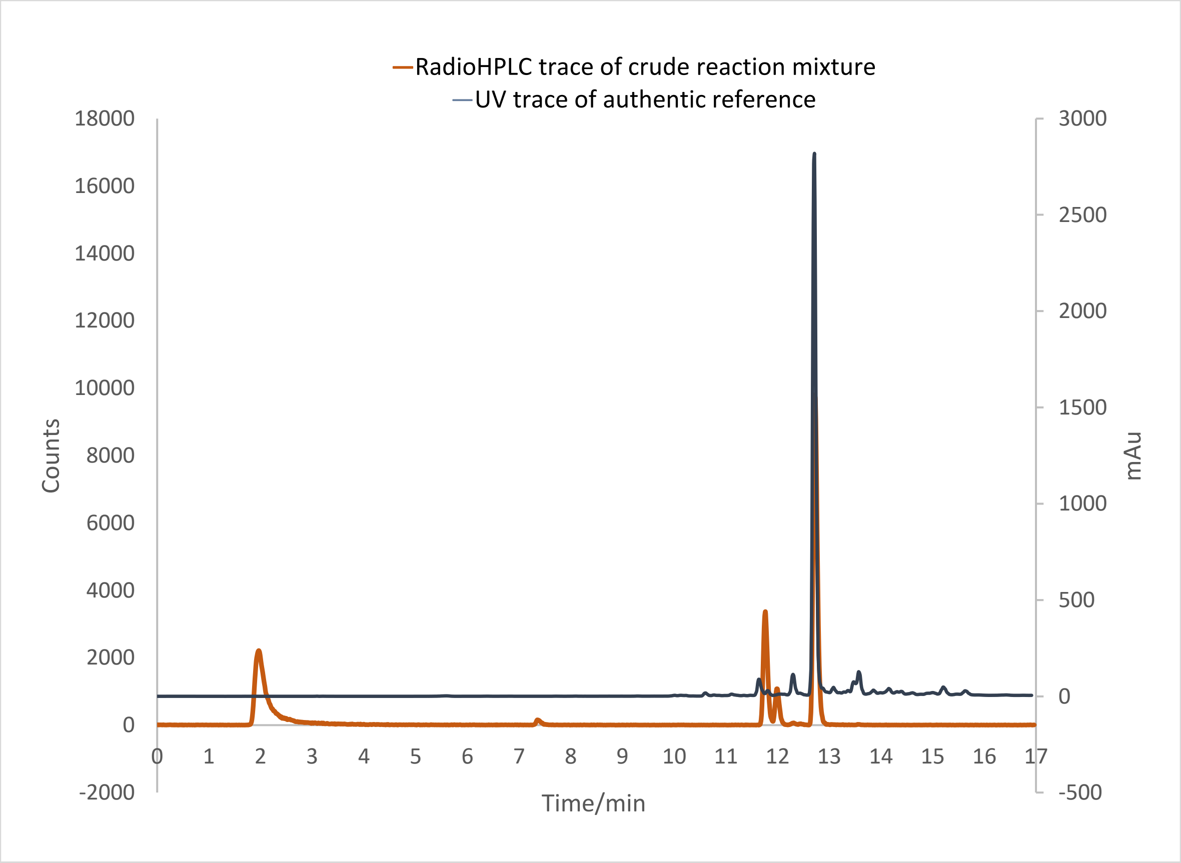

UV/radio-HPLC trace overlay for [^{18}F] 1-bromo-2-(3,3-difluoropropyl)benzene ([^{18}F]2h)

Prepared following the general procedure and analysed by (radio)HPLC using conditions A.

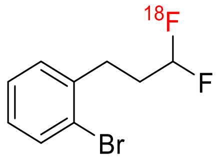

Run	RCY (%) (rTLC)	RCP (%) (rHPLC)	RCC (%)
1	59	100	59
2	57	100	57
3	60	100	60
	Average RCC	59% ± 1% (n=3)	

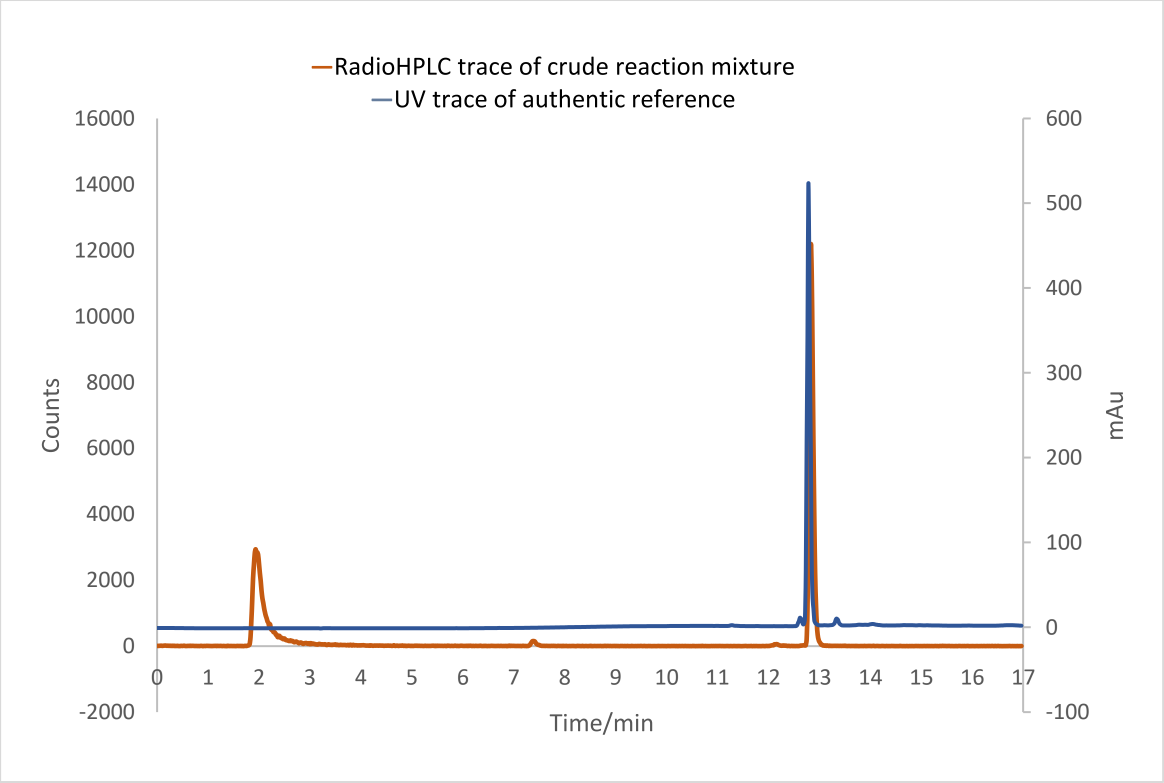

UV/radio-HPLC trace overlay for [^{18}F] (5,5-difluoro-1-phenylpentan-1-one ([^{18}F]2i)

Prepared following the general procedure and analysed by (radio)HPLC using conditions A.

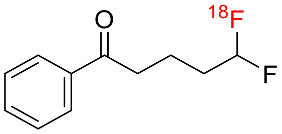

Run	RCY (%) (rTLC)	RCP (%) (rHPLC)	RCC (%)
1	70	84	59
2	77	97	75
3	73	71	52
Average RCC		62% ± 10% (n=3)	

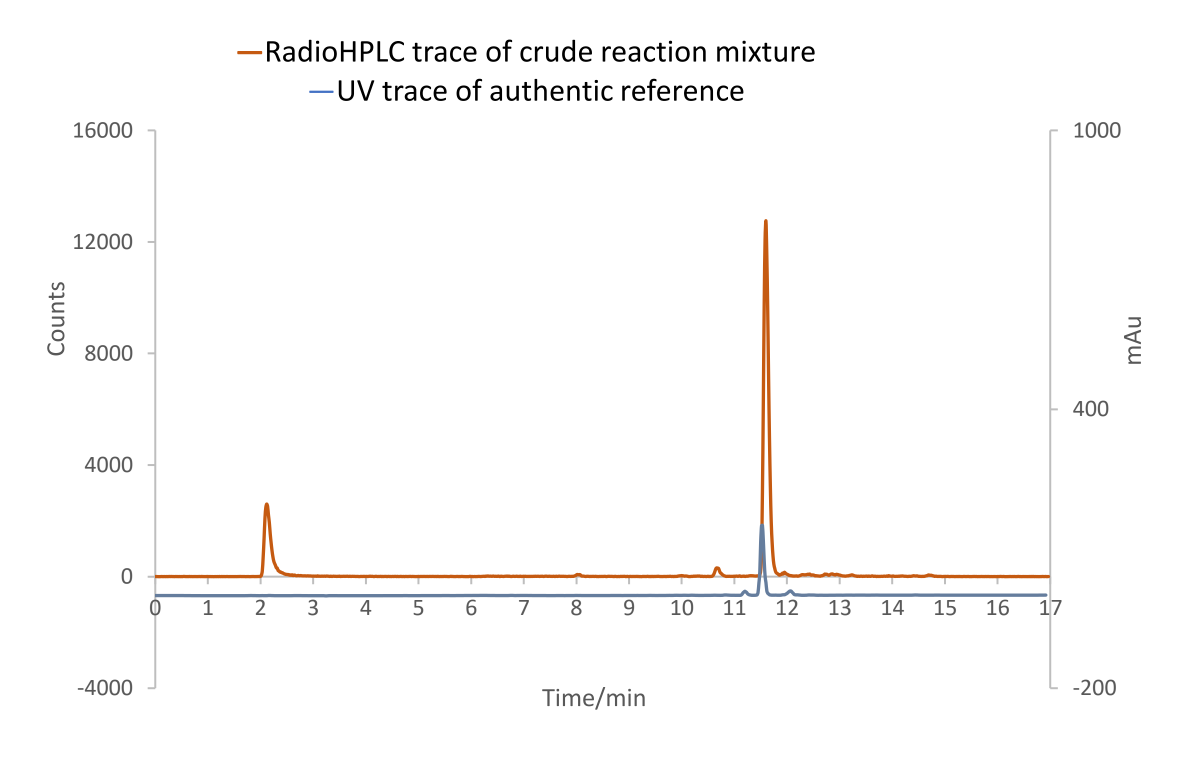

UV/radio-HPLC trace overlay for [^{18}F] methyl 4-(3,3-difluoropropyl)benzoate ([^{18}F]2j)

Prepared following the general procedure and analysed by (radio)HPLC using conditions A.

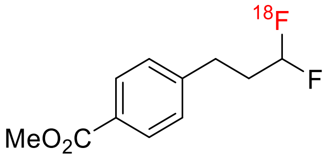

Run	RCY (%) (rTLC)	RCP (%) (rHPLC)	RCC (%)
1	52	100	52
2	32	100	32
3	29	100	29
Average RCC		37% ± 10% (n=3)	

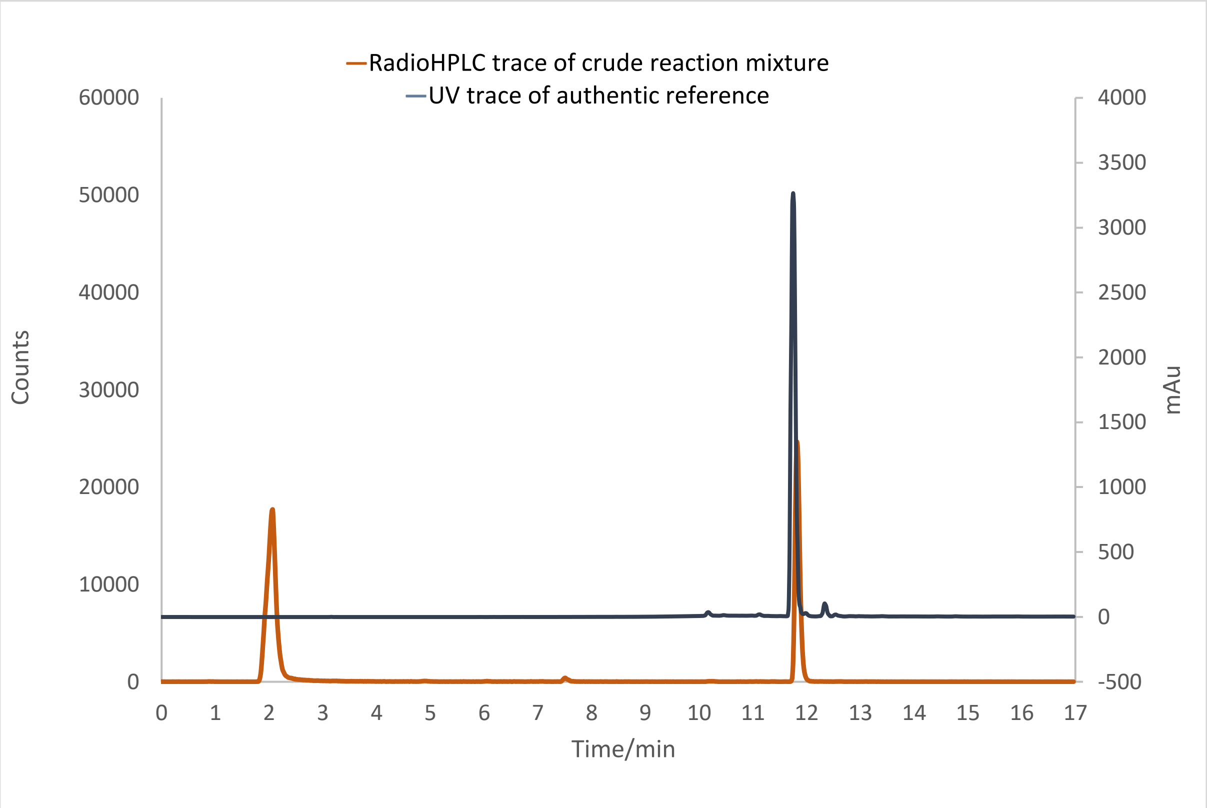

UV/radio-HPLC trace overlay for [^{18}F] (5,5-difluoropentyl)benzene ([^{18}F]2k)

Prepared following the general procedure and analysed by (radio)HPLC using conditions A.

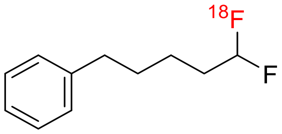

Run	RCY (%) (rTLC)	RCP (%) (rHPLC)	RCC (%)
1	33	100	33
2	30	100	30
3	46	50	23
	**Average RCC**	**29% ± 4% (n=3)**	

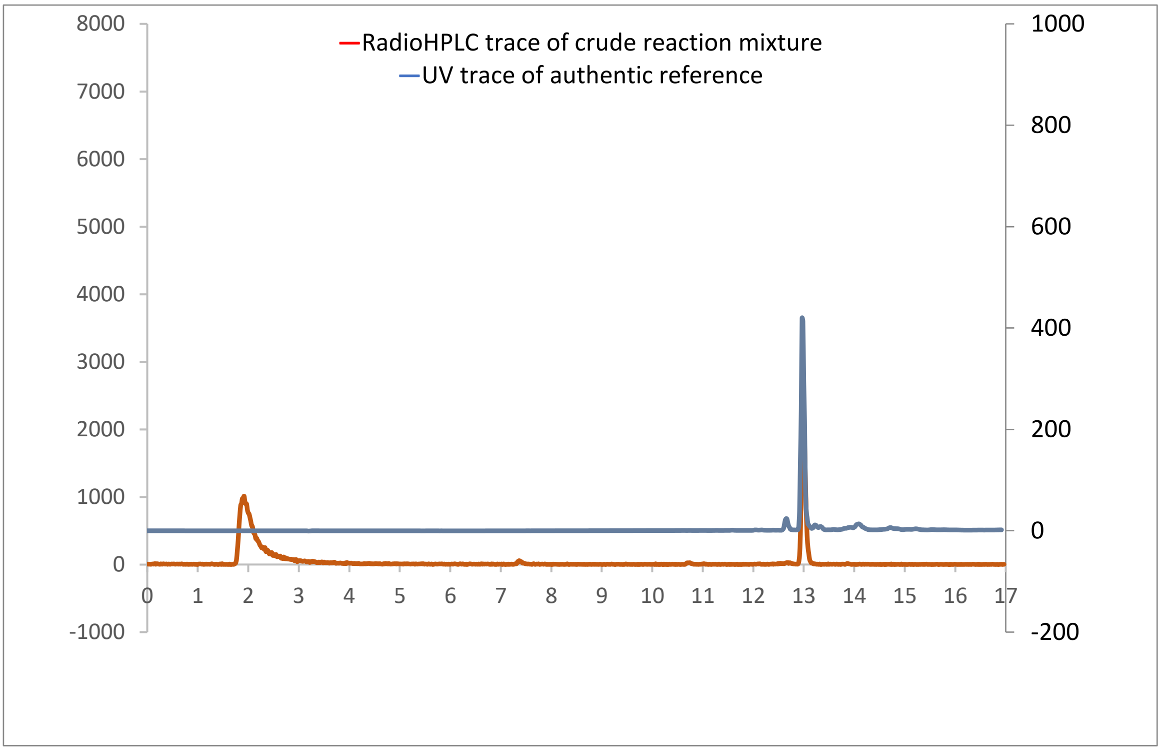

UV/radio-HPLC trace overlay for [^{18}F] 2-(3,3-difluoropropyl)naphthalene ([^{18}F]2l)

Prepared following the general procedure and analysed by (radio)HPLC using conditions A.

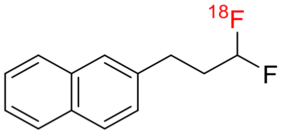

Run	RCY (%) (rTLC)	RCP (%) (rHPLC)	RCC (%)
1	76	82	61
2	66	90	59
3	45	87	39
**Average RCC**		**53% ± 10% (n=3)**	

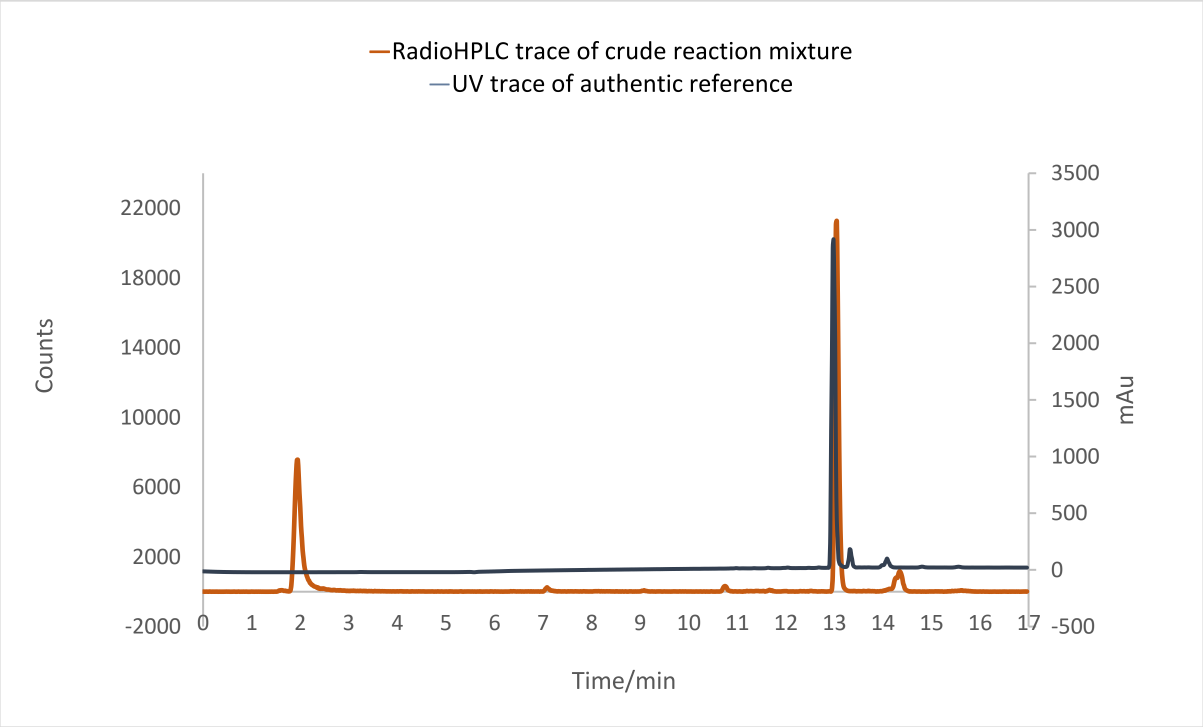

UV/radio-HPLC trace overlay for [^{18}F] 2-(2,2-difluoroethyl)naphthalene ([^{18}F]2m)

Prepared following the general procedure and analysed by (radio)HPLC using conditions A.

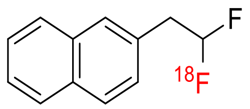

Run	RCY (%) (rTLC)	RCP (%) (rHPLC)	RCC (%)
1	33	100	33
2	30	100	30
3	29	100	29
Average RCC		31% ± 2% (n=3)	

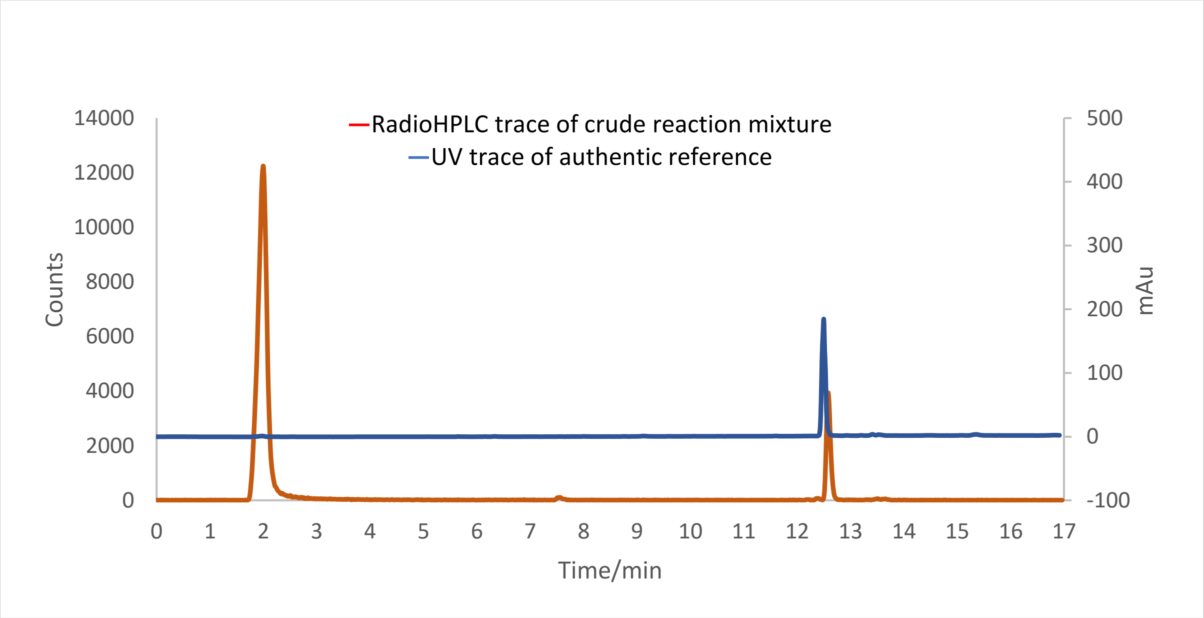

UV/radio-HPLC trace overlay for [^{18}F] methyl 4-(2,2-difluoroethyl)benzoate ([^{18}F]2n)

Prepared following the general procedure and analysed by (radio)HPLC using conditions A.

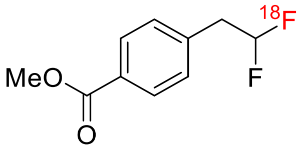

Run	RCY (%) (rTLC)	RCP (%) (rHPLC)	RCC (%)
1	20	100	20
2	25	100	25
3	25	100	25
**Average RCC**		**23% ± 2% (n=3)**	

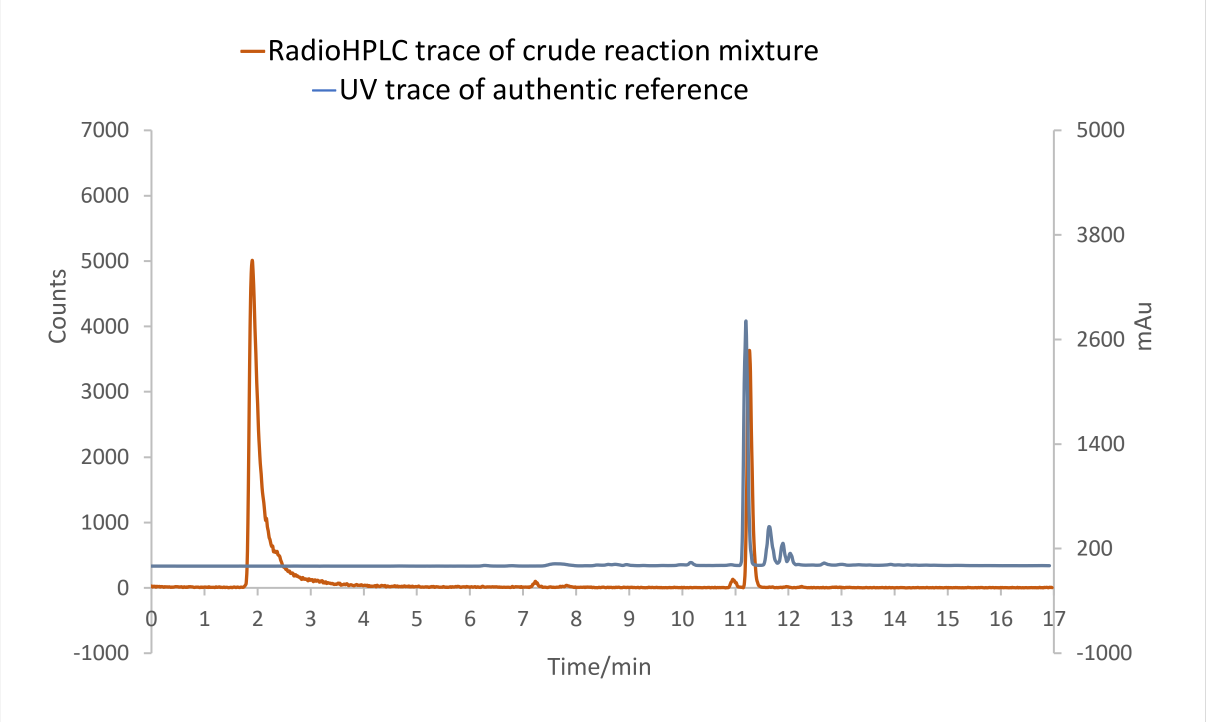

UV/radio-HPLC trace overlay for [^{18}F] 2-(6,6-difluorohexyl)isoindoline-1,3-dione ([^{18}F]2p)

Prepared following the general procedure and analysed by (radio)HPLC using conditions A.

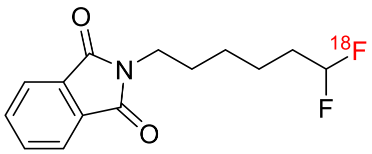

Run	RCY (%) (rTLC)	RCP (%) (rHPLC)	RCC (%)
1	66	100	66
2	61	100	61
3	45	100	45
**Average RCC**		**57% ± 9% (n=3)**	

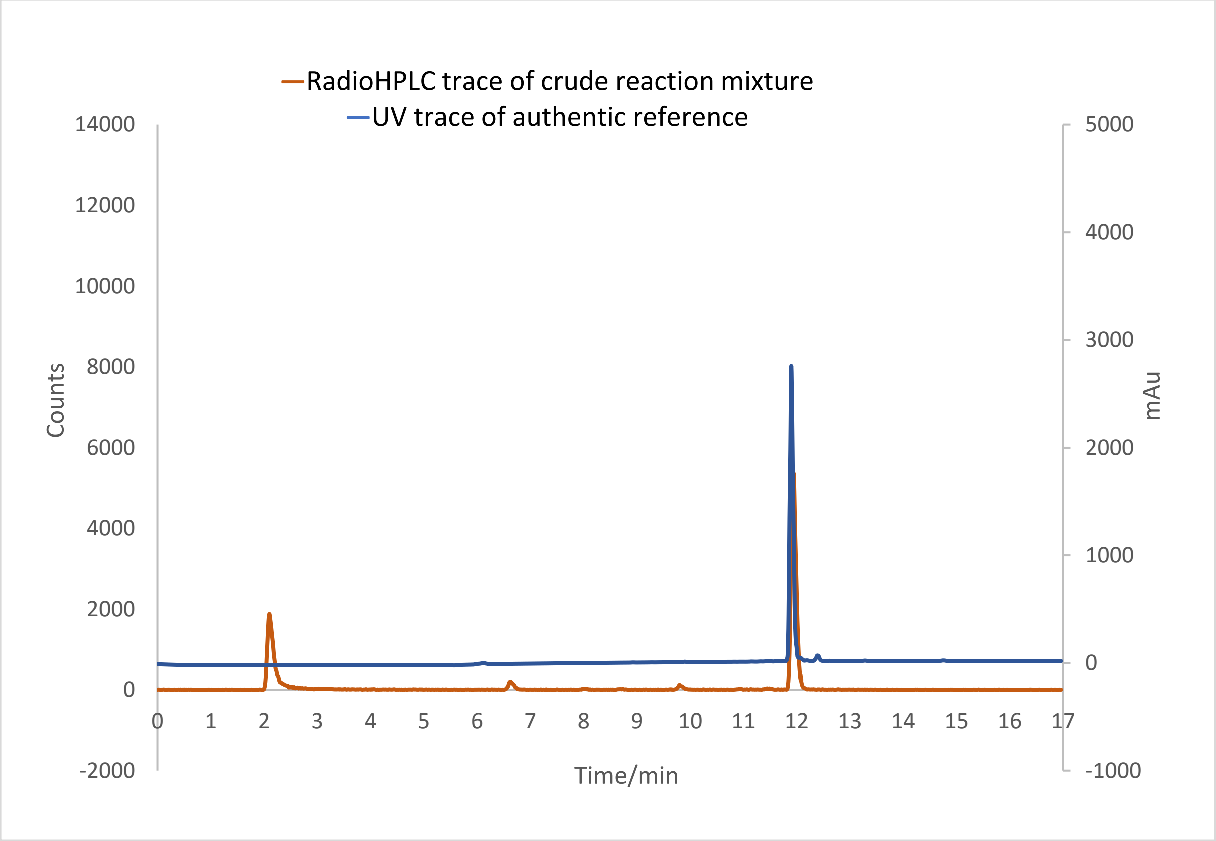

UV/radio-HPLC trace overlay for [^{18}F] *N*-(3,3-difluoropropyl)-4-methyl-*N*-phenylbenzenesulfonamide ([^{18}F]2q)

Prepared following the general procedure and analysed by (radio)HPLC using conditions A.

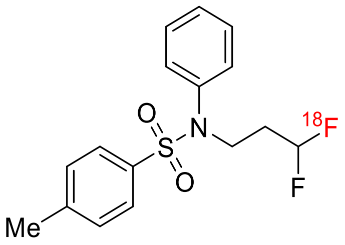

Run	RCY (%) (rTLC)	RCP (%) (rHPLC)	RCC (%)
1	67	100	67
2	65	100	65
3	455	100	55
Average RCC		63% ± 5% (n=3)	

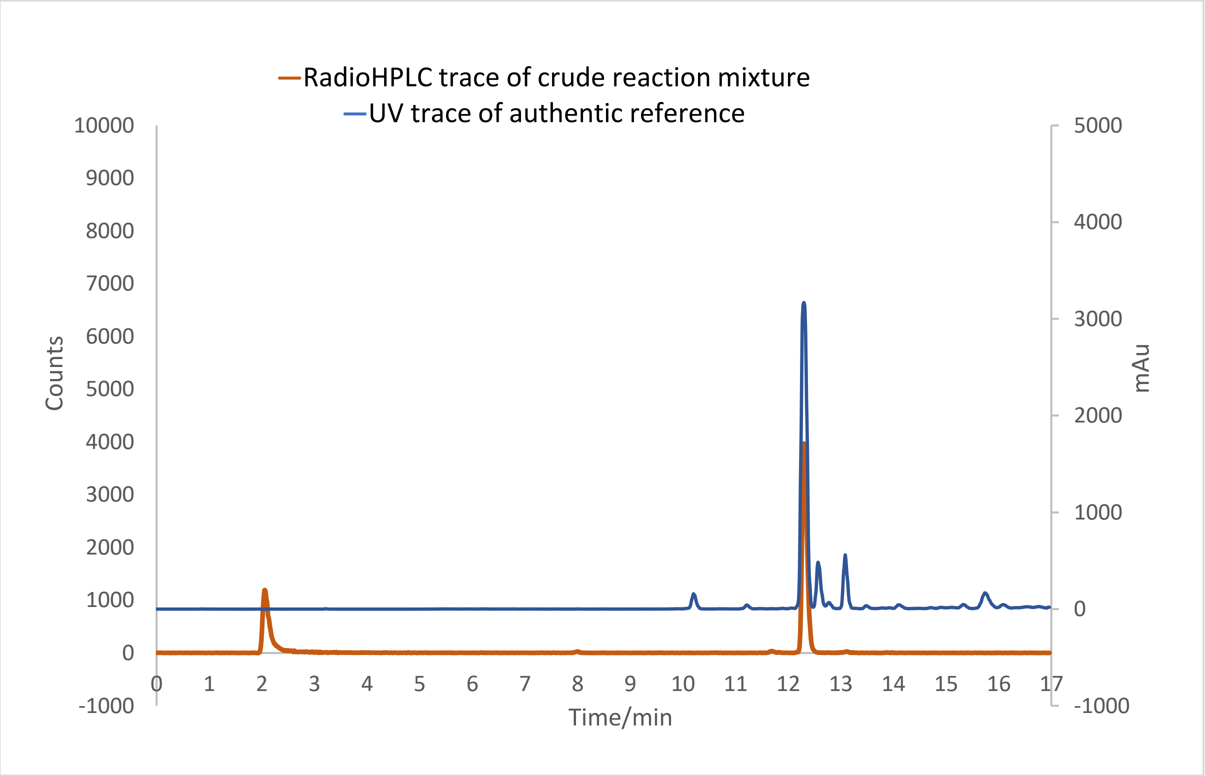

UV/radio-HPLC trace overlay for [^{18}F] 4-(3,3-difluoropropyl)-*N*-methoxy-*N*-methyl benzamide ([^{18}F]2r)

Prepared following the general procedure and analysed by (radio)HPLC using conditions A.

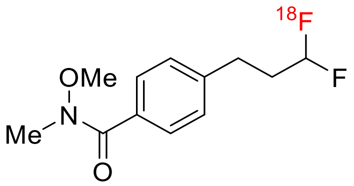

Run	RCY (%) (rTLC)	RCP (%) (rHPLC)	RCC (%)
1	31	100	31
2	29	97	28
3	24	97	23
	Average RCC	27% ± 3% (n=3)	

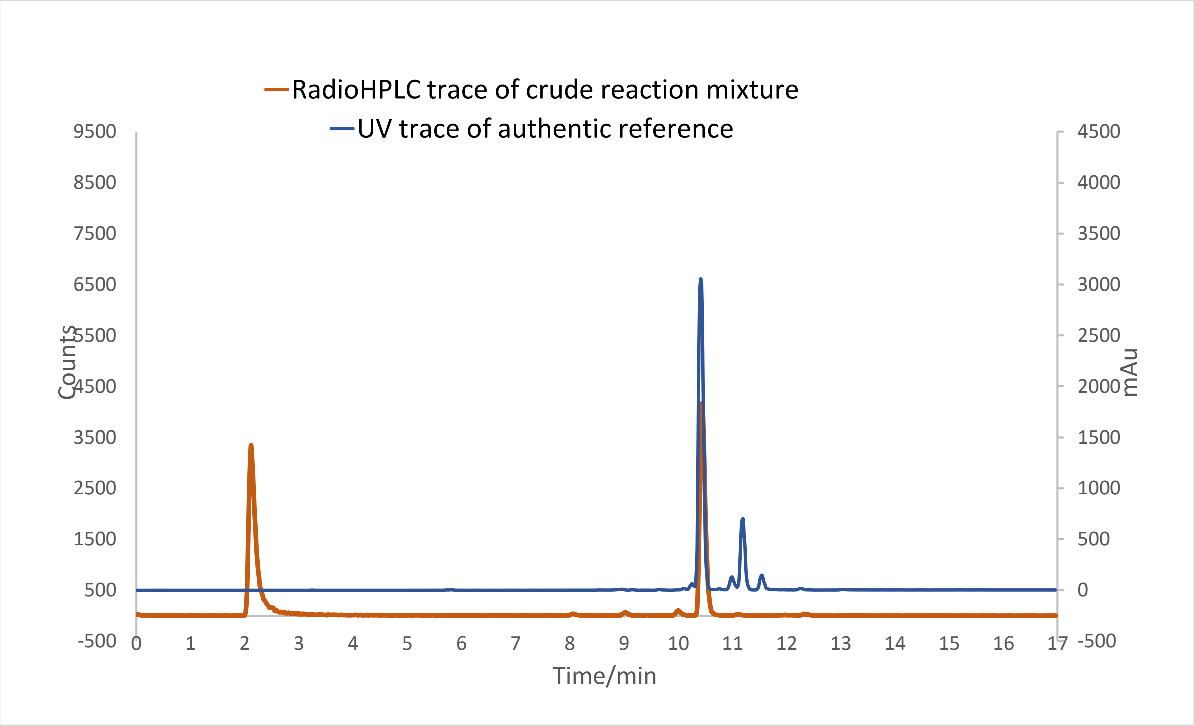

UV/radio-HPLC trace overlay for [^{18}F] 3-(3,5-dichlorophenyl)-5-(3,3-difluoropropyl)-5-methyloxazolidine-2,4-dione ([^{18}F]2s)

Prepared following the general procedure and analysed by (radio)HPLC using conditions A.

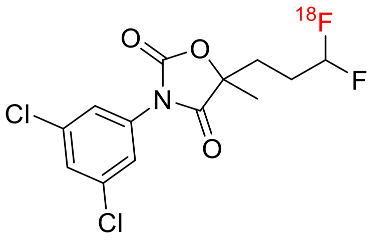

Run	RCY (%) (rTLC)	RCP (%) (rHPLC)	RCC (%)
1	26	95	25
2	34	88	30
3	39	89	35
	Average RCC	30% ± 4% (n=3)	

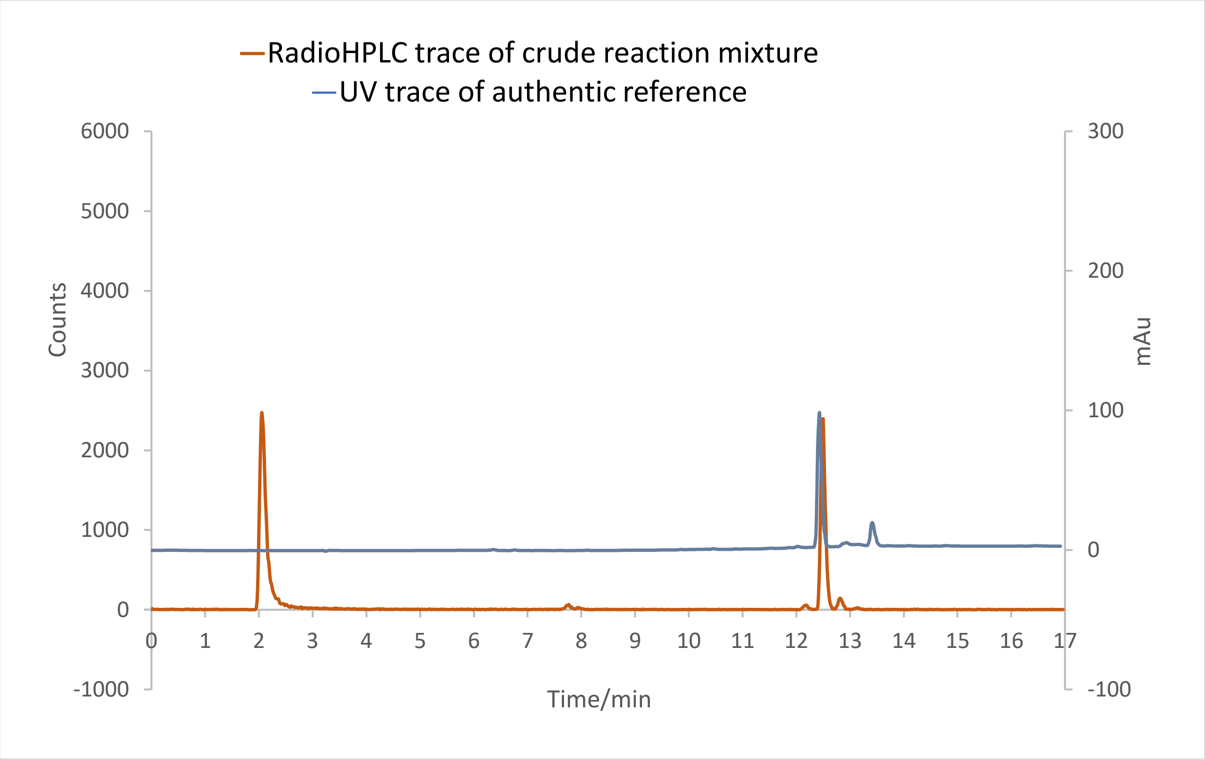

UV/radio-HPLC trace overlay for [^{18}F] 2,2-difluoro-3,4-dihydronaphthalen-1(2H)-one ([^{18}F]2t)

Prepared following the general procedure and analysed by (radio)HPLC using conditions A.

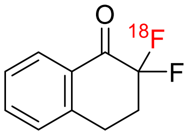

Run	RCY (%) (rTLC)	RCP (%) (rHPLC)	RCC (%)
1	8	95	8
2	12	95	12
3	6	100	6
**Average RCC**		**9% ± 3% (n=3)**	

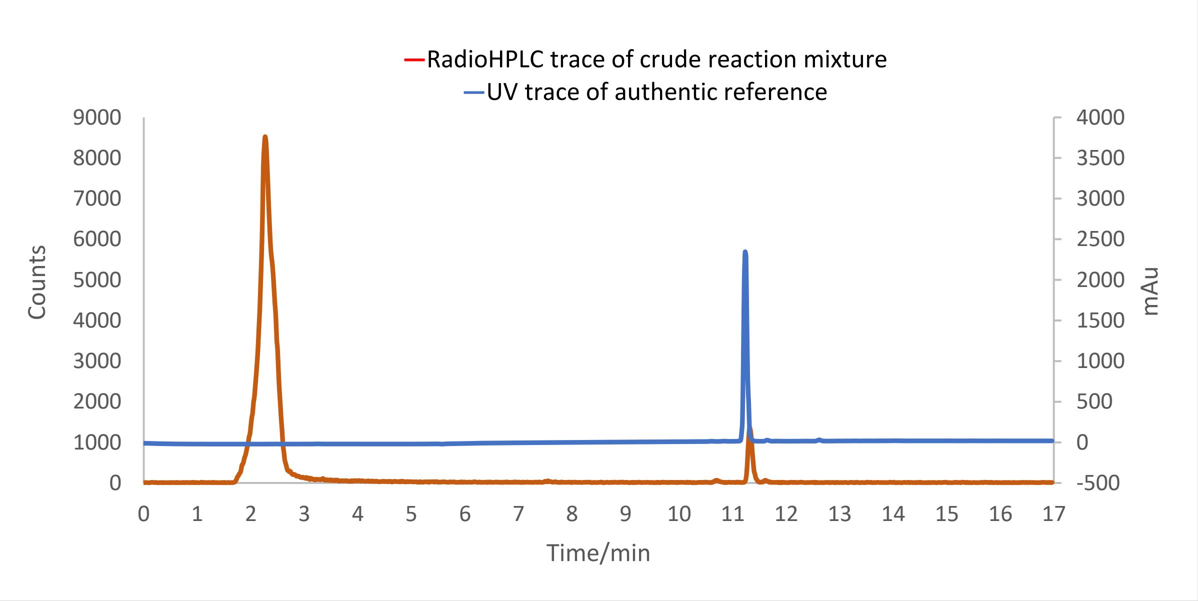

UV/radio-HPLC trace overlay for [^{18}F] 2,2-difluoro-1-phenylbutan-1-one ([^{18}F]2u)

Prepared following the general procedure and analysed by (radio)HPLC using conditions A.

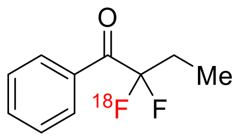

Run	RCY (%) (rTLC)	RCP (%) (rHPLC)	RCC (%)
1	16	100	16
2	19	100	19
3	30	100	30
**Average RCC**		**22% ± 6% (n=3)**	

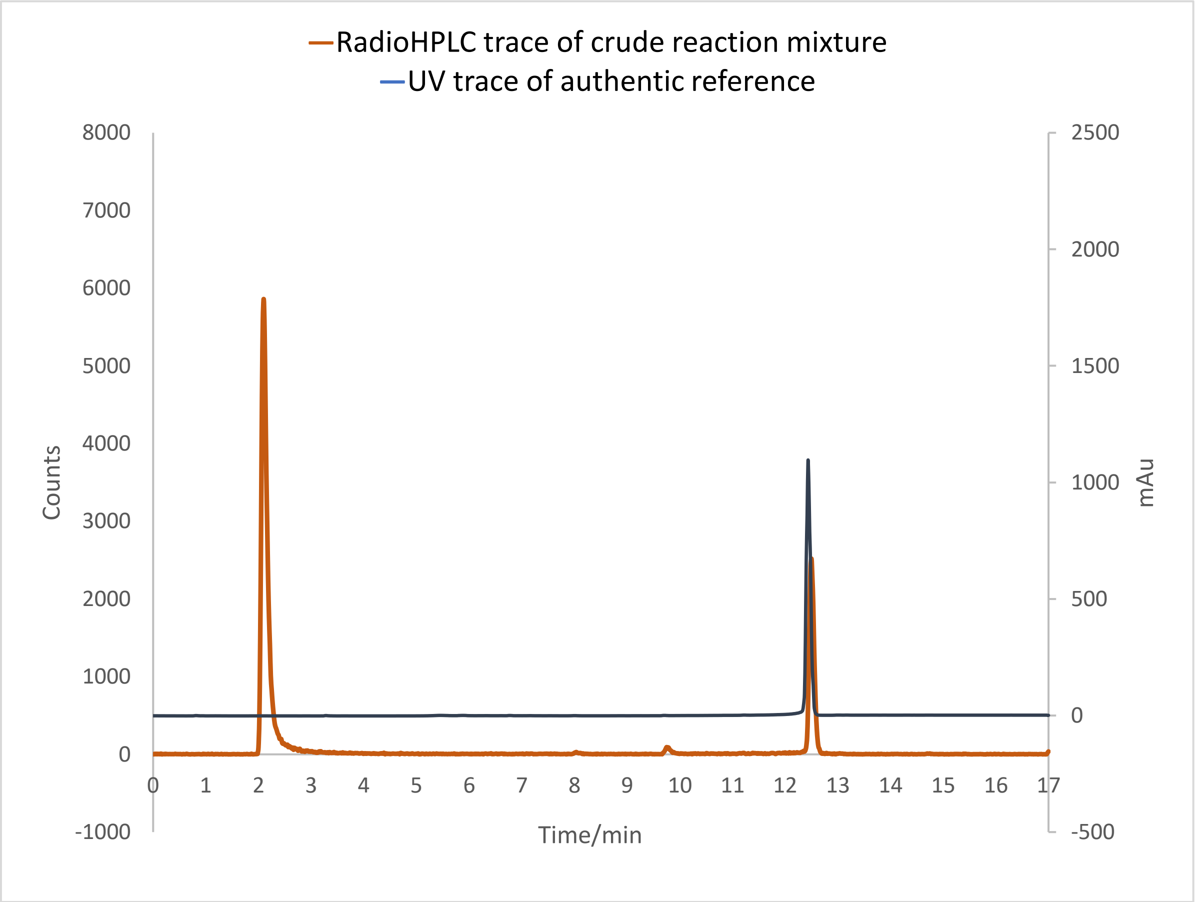

UV/radio-HPLC trace overlay for [^{18}F] (3,3-difluorobutyl)benzene ([^{18}F]2v)

Prepared following the general procedure and analysed by (radio)HPLC using conditions A.

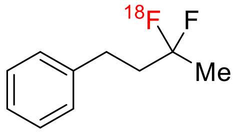

Run	RCY (%) (rTLC)	RCP (%) (rHPLC)	RCC (%)
1	13	73	10
2	12	76	9
3	17	70	12
Average RCC		10% ± 1% (n=3)	

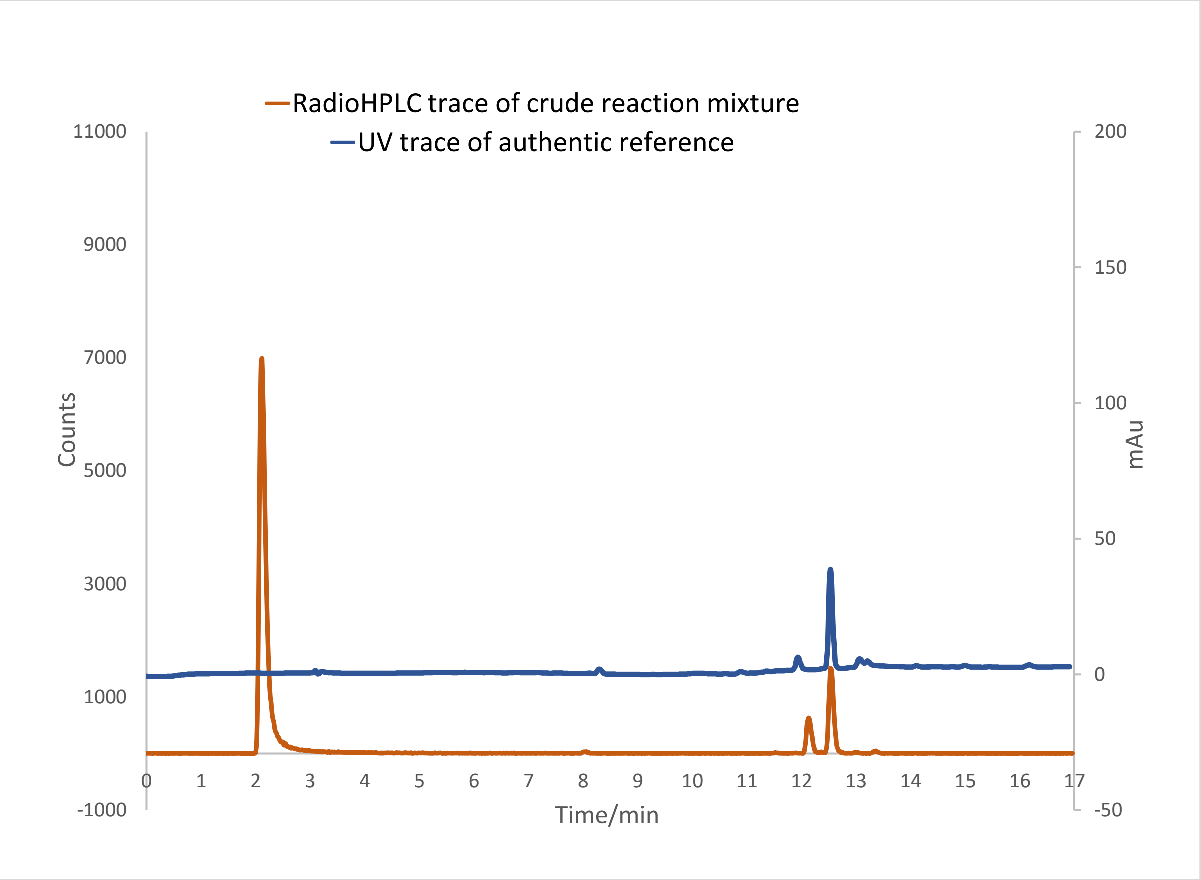

UV/radio-HPLC trace overlay for [^{18}F] (4,4-difluoropiperidin-1-yl)(phenyl)methanone ([^{18}F]2w)

Prepared following the general procedure and analysed by (radio)HPLC using conditions A.

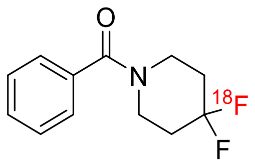

Run	RCY (%) (rTLC)	RCP (%) (rHPLC)	RCC (%)
1	9	55	5
2	16	62	10
3	5	70	4
**Average RCC**		**6% ± 3% (n=3)**	

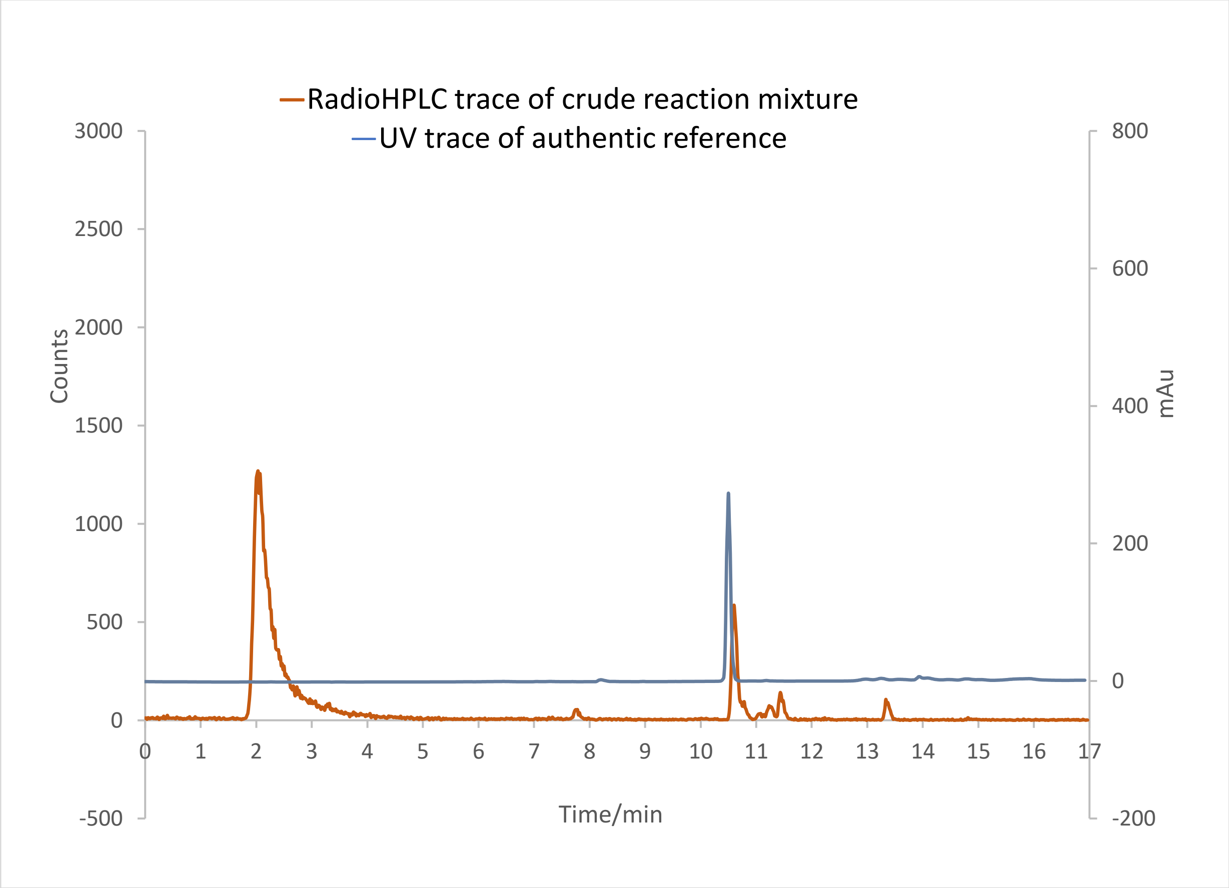

UV/radio-HPLC trace overlay for [^{18}F] 1-chloro-4-(4,4-difluorocyclohexyl)benzene ([^{18}F]2x)

Prepared following the general procedure and analysed by (radio)HPLC using conditions A.

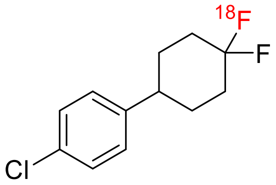

Run	RCY (%) (rTLC)	RCP (%) (rHPLC)	RCC (%)
1	6	100	6
2	5	100	5
3	6	100	6
**Average RCC**		**6% ± 1% (n=3)**	

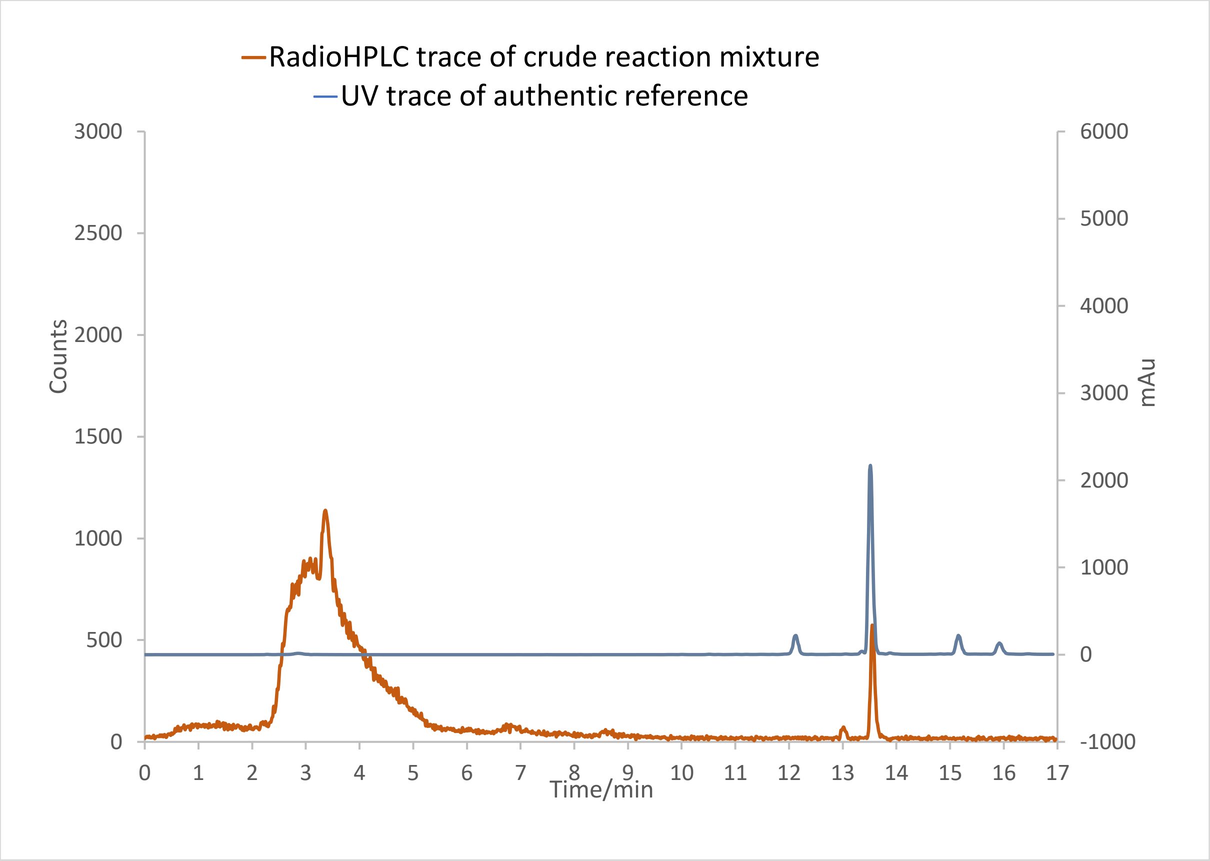

UV/radio-HPLC trace overlay for [^{18}F] 4-(difluoromethyl)-1,1'-biphenyl ([^{18}F]2y)

Prepared following the general procedure and analysed by (radio)HPLC using conditions A.

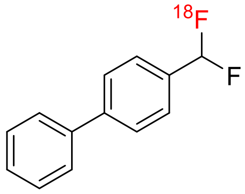

Run	RCY (%) (rTLC)	RCP (%) (rHPLC)	RCC (%)
1	86	100	86
2	84	100	84
3	78	100	78
**Average RCC**		**83% ± 3% (n=3)**	

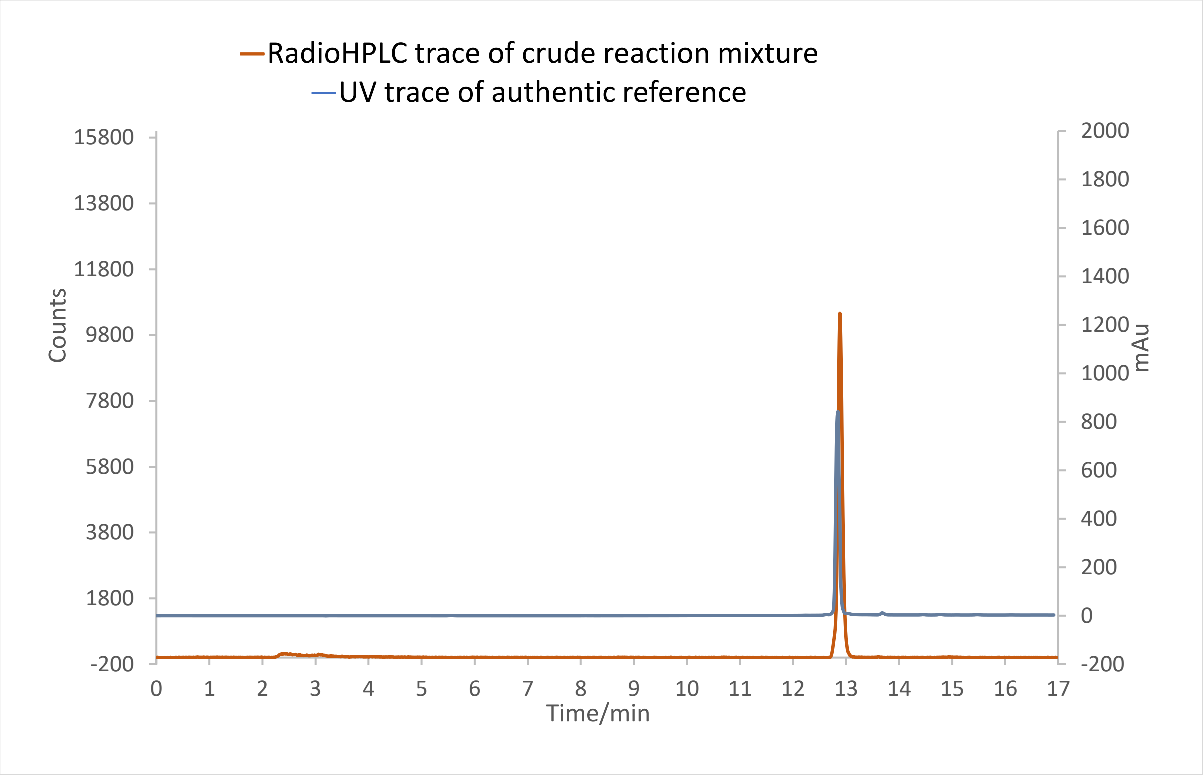

UV/radio-HPLC trace overlay for [^{18}F] 4-(trifluoromethyl)-1,1'-biphenyl ([^{18}F]2aa)

Prepared following the general procedure and analysed by (radio)HPLC using conditions A.

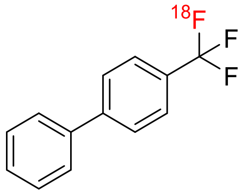

Run	RCY (%) (rTLC)	RCP (%) (rHPLC)	RCC (%)
1	52	24	12
2	53	22	12
3	48	20	10
	**Average RCC**	**11% ± 1% (n=3)**	

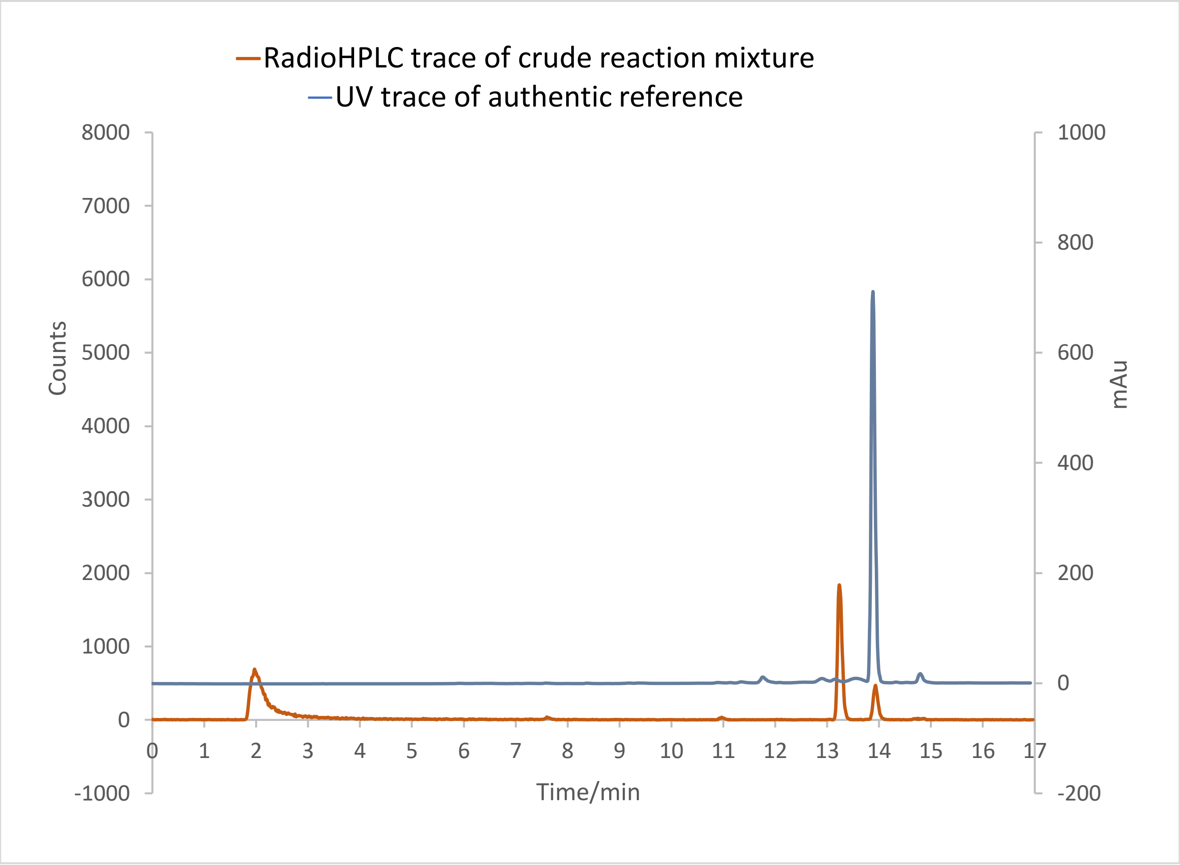

UV/radio-HPLC trace overlay for [^{18}F] 2,2,2-trifluoro-1-(4-methoxyphenyl)ethan-1-one ([^{18}F]2ab)

Prepared following the general procedure and analysed by (radio)HPLC using conditions A.

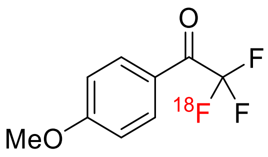

Run	RCY (%) (rTLC)	RCP (%) (rHPLC)	RCC (%)
1	14	43	6
2	10	35	4
3	24	62	15
Average RCC		8% ± 5% (n=3)	

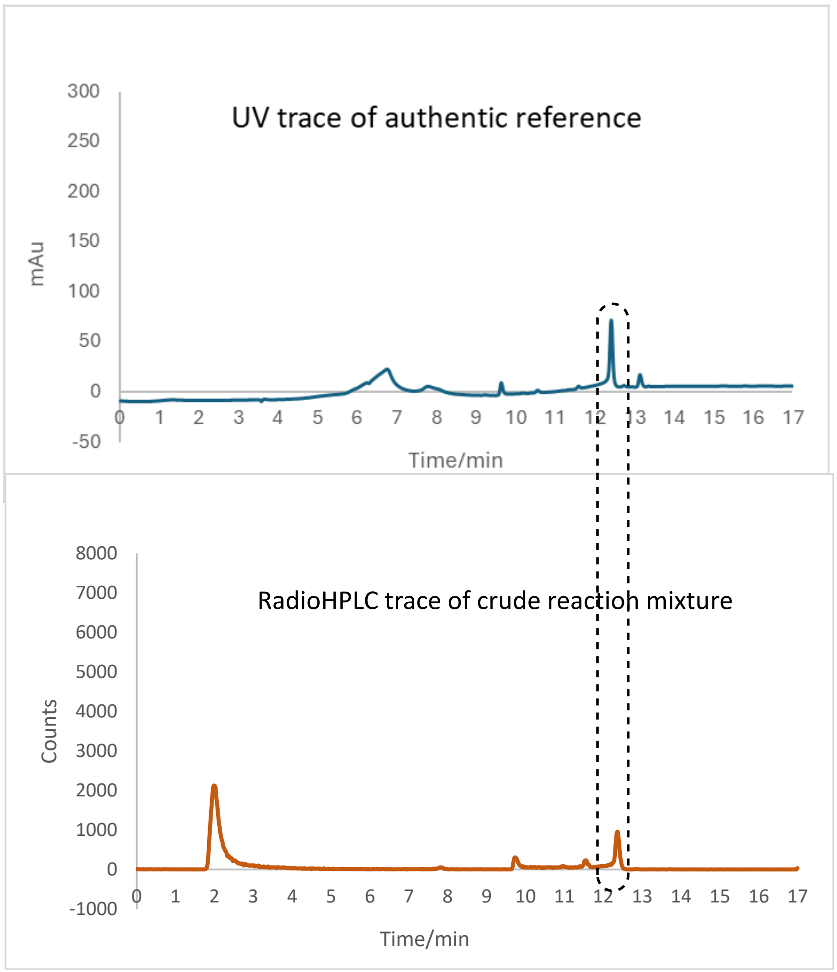

UV/radio-HPLC trace overlay for [^{18}F] (3-fluorobutyl)benzene ([^{18}F]5a)

Prepared following the general procedure and analysed by (radio)HPLC using conditions A.

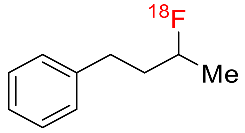

Run	RCY (%) (rTLC)	RCP (%) (rHPLC)	RCC (%)
1	20	100	20
2	14	100	14
3	15	100	15
	**Average RCC**	**16% ± 3% (n=3)**	

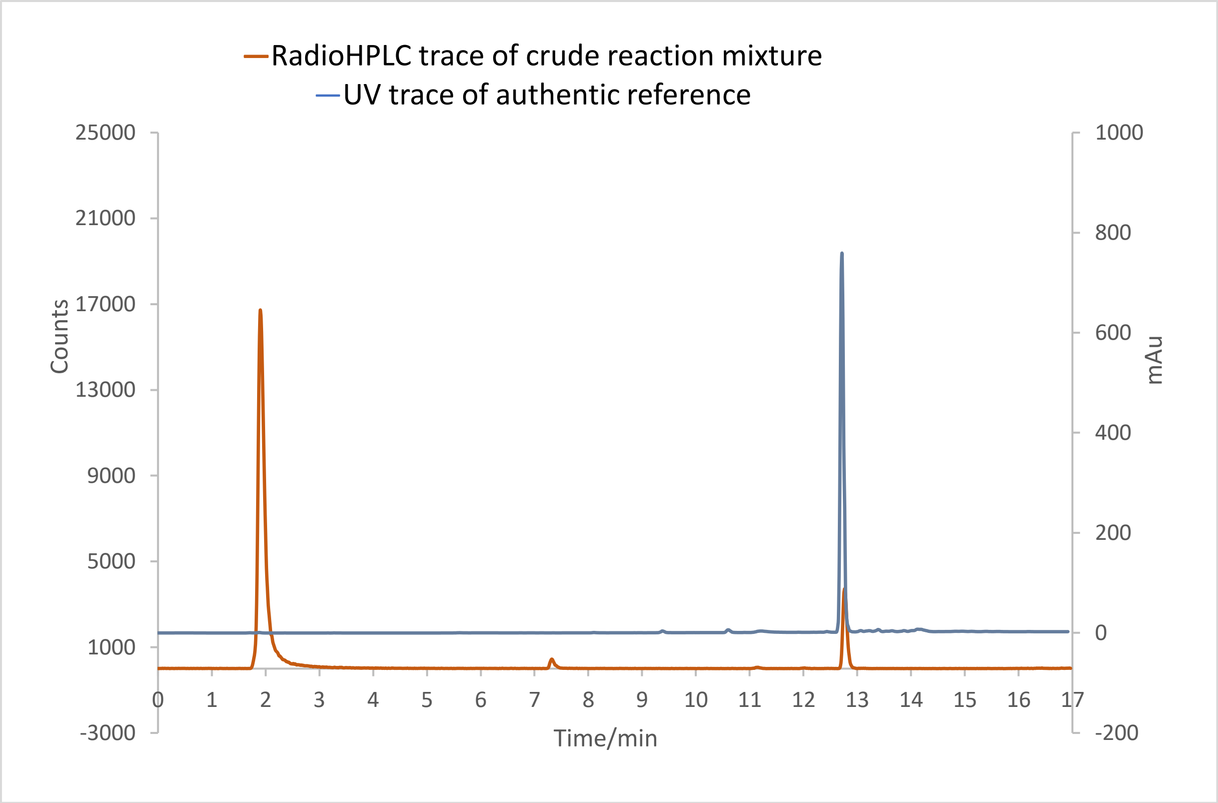

References:

1. (a) Jiang, X.; Sakthivel, S.; Kulbitski, K.; Nisnevich, G.; Gandelman, M. *J. Am. Chem. Soc*. **2014**, *136*, 9548. (b) Jiang, X.; Gandelman, M. *J. Am. Chem. Soc*. **2015**, *137*, 2542. (c) Li, W.; Varenikov, A.; Gandelman, M. *Eur. J. Org. Chem*. **2020**, *2020*, 3138−3141. (d) Zhu, F.; Xu, P. W.; Zhou, F.; Wang, C. H.; Zhou, J. *Org. Lett*. **2015**, *17*, 972–975.
2. Chen, F.; Xu, X.-H.; Qing, F.-L. *Org. Lett.* **2021**, *23*, 2364–2369.
3. Liang, J.; Han, J.; Wu, J.; Wu, P.; Hu, J.; Hu, F.; Wu, F. *Org. Lett*. **2019,** *21*, 6844–6849.
4. Xueling Mo, X.; Huang, H.; Zhang, G. *ACS Catal*. **2022**, *12*, 9944–9952.
5. Takeuchi, Y.; Kanada, A.; Kawahara, S.-i.; Koizumi, T. *J. Org. Chem*. **1993**, *58*, 3483-3485.
6. Gandelman, M; Nisnevich, G.; Kulbitski, K.; Artaryan. A. Process for the preparation of organic bromides. WO 2017/060905 A1, 2017.
7. Zhao, H.-Y.; Zhou, M.; Zhang, X. *Org. Lett*. **2021**, *23*, 9106–911.
8. Gu, J.-W.; Guo, W.-H.; Zhang, X. *Org. Chem. Front*. **2015**, *2*, 38-41.
9. Igarashi, C.; Mayumi, T.; Egami H.; Hamashima, Y. *Org. Lett*. **2024**, *26*, 1723−1727.
10. Dryzhakov, M.; Moran J. *ACS Catal*. **2016**, *6*, 3670-3673.
11. Kim, S.; Hwang, K.H.; Park, H.G.; Kwak, J.; Lee, H.; Kim, H. *Commun. Chem.* **2022***,* *5*, 96.
12. Lin, Q. Y.; Xu, X. H.; Zhang, K.; Qing, F. L. *Angew. Chem. Int. Ed.* **2016**, *55*, 1479−1483.
13. Qi, X.-K.; Yao, L.-J.; Zheng, M.-J.; Zhao, L.; Yang, C.; Guo, L.; Xia,W. *Angew. Chem., Int. Ed.* **2022**, *61*, e202210151.
14. Li, Y. -L.; Li, J.; Deng, J. *Adv. Synth. Catal*. **2017**, *359*, 1407 – 1412.
15. Lina, C.; Lina, Z.; Yea, Z.; Chena, L.; Lia, Y.; Shenc, C.; Zhang, M. *Tetrahedron Lett.* **2022**, *110*, 154197.
16. Wang, X.; Wang, X.; Pan, H.; Ming, X.; Zhang, Z.; Wang, T. *J. Org. Chem*. **2022**, *87*, 10173−10184.
17. Wang, Z.; Guo, C.-Y.; Yang, C.; Chen, J.-P. *J. Am. Chem. Soc*. **2019**, *141*, 5617−5622.
18. Mizuta, S.; Stenhagen, I. S. R.; O'Duill, M.;Wolstenhulme, J.; Kirjavainen, A. K.; Forsback, S. J.; Tredwell, M.; Sandford, G.; Moore, P. R.; Huiban, M.; Luthra, S. K.; Passchier, J.;Solin, O.; Gouverneur, V. *Org. Lett.* **2013**, *15*, 2648−2651.
19. Yang, Y.-F.; Lin, J.-H.; Xiao, J.-C. *Org. Lett*. **2021**, *23*, 9277-92-82.
20. Zhou, G.; Guo, Z.; Liu, S.; Shen X. *J. Am. Chem. Soc*. **2024**, *146*, 4026–4035.
21. Lucas Kane, D.; Figula, B.C.; Balaraman, K.; Bertke, J. A.; Wolf, C. *Nat. Commun*. **2024**, *15*, 1866.
22. Haveman, L.Y.F.; Vugts, D.J.; Windhorst, A.D. *EJNMMI radiopharm. chem*. **2023**, *8*, 28.

NMR Spectra: **¹H NMR (400 MHz, CDCl₃)**

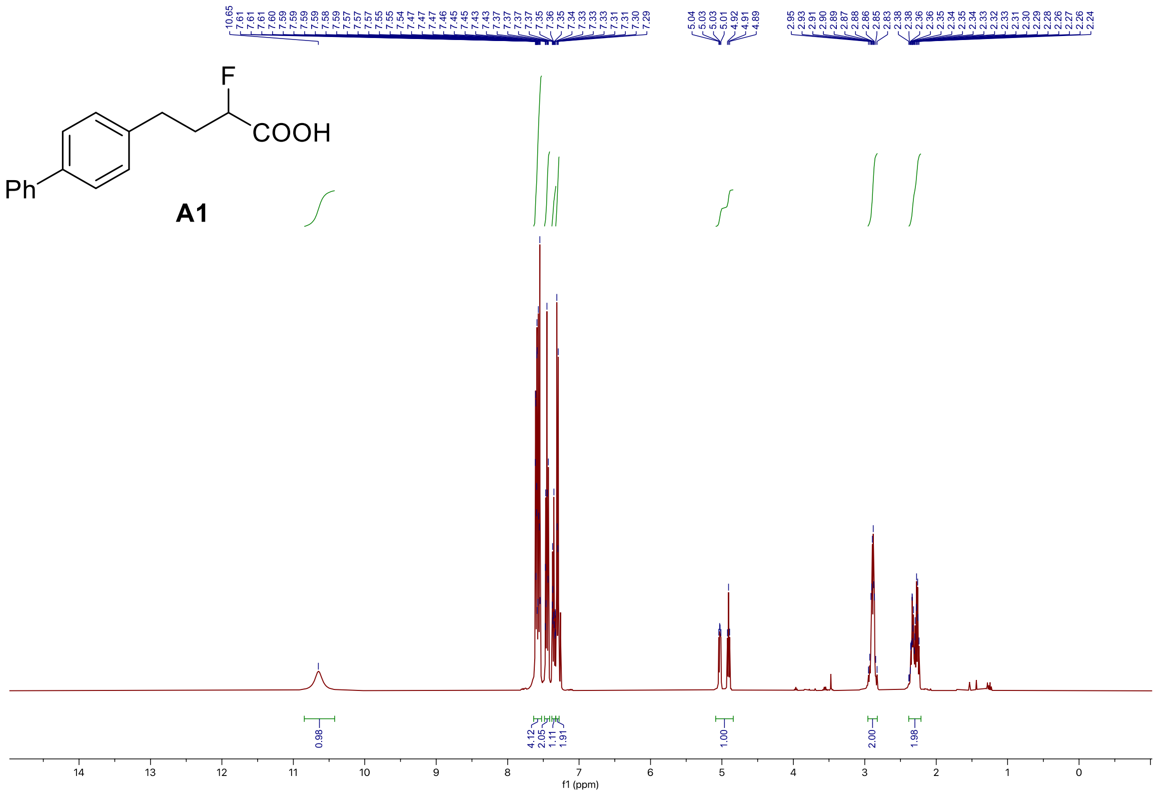

¹⁹F NMR (376 MHz, CDCl₃)

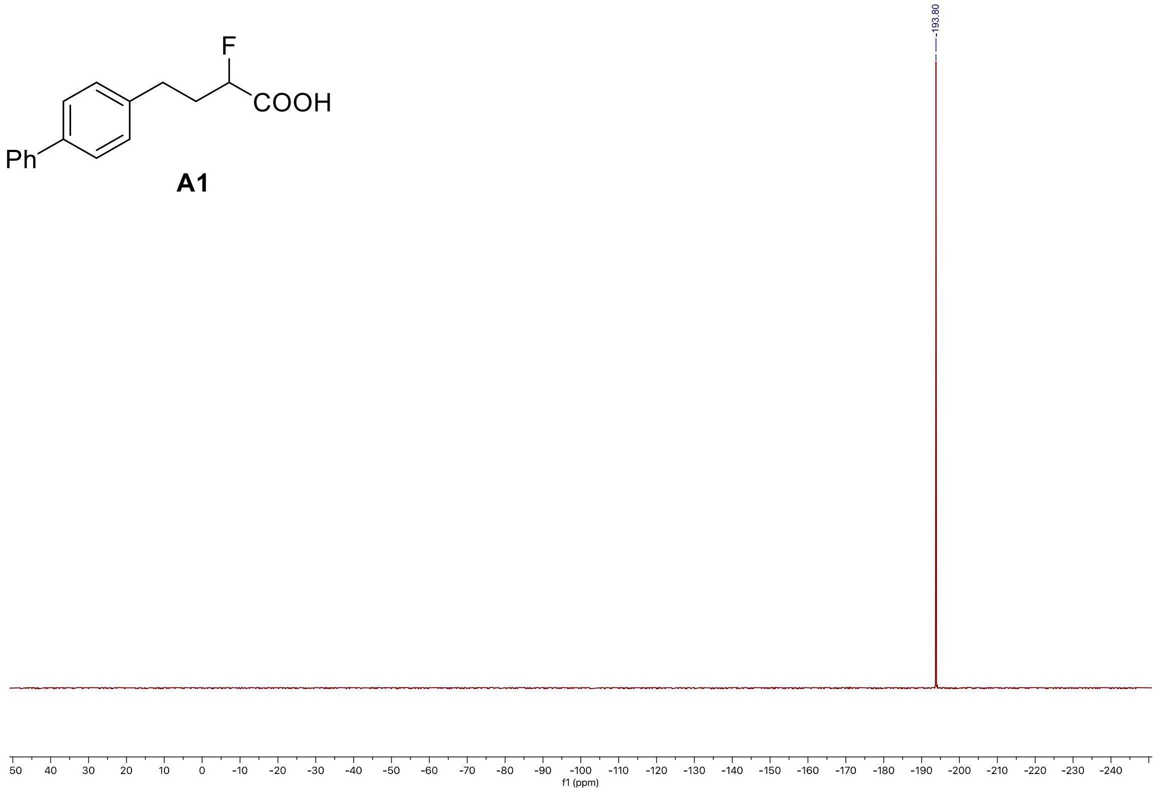

^{13}C NMR (101 MHz, $CDCl_3$)

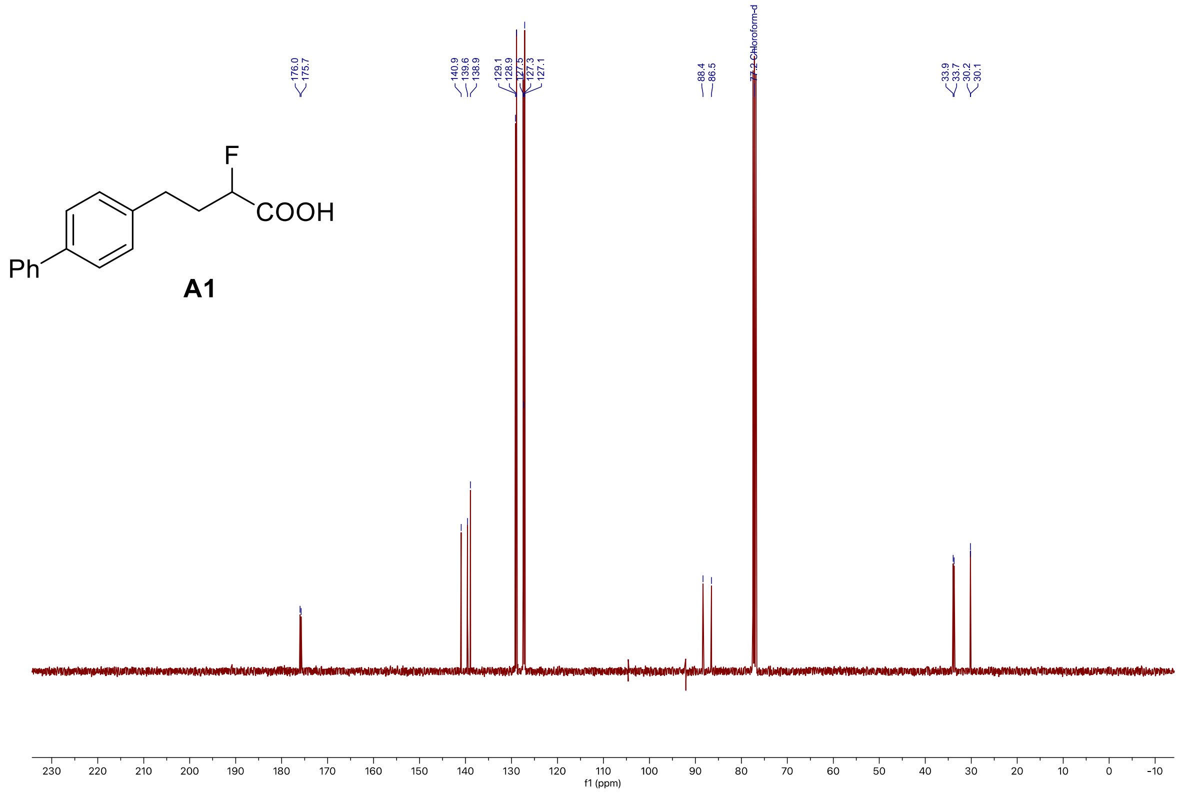

A1

^{1}H NMR (400 MHz, $CDCl_3$)

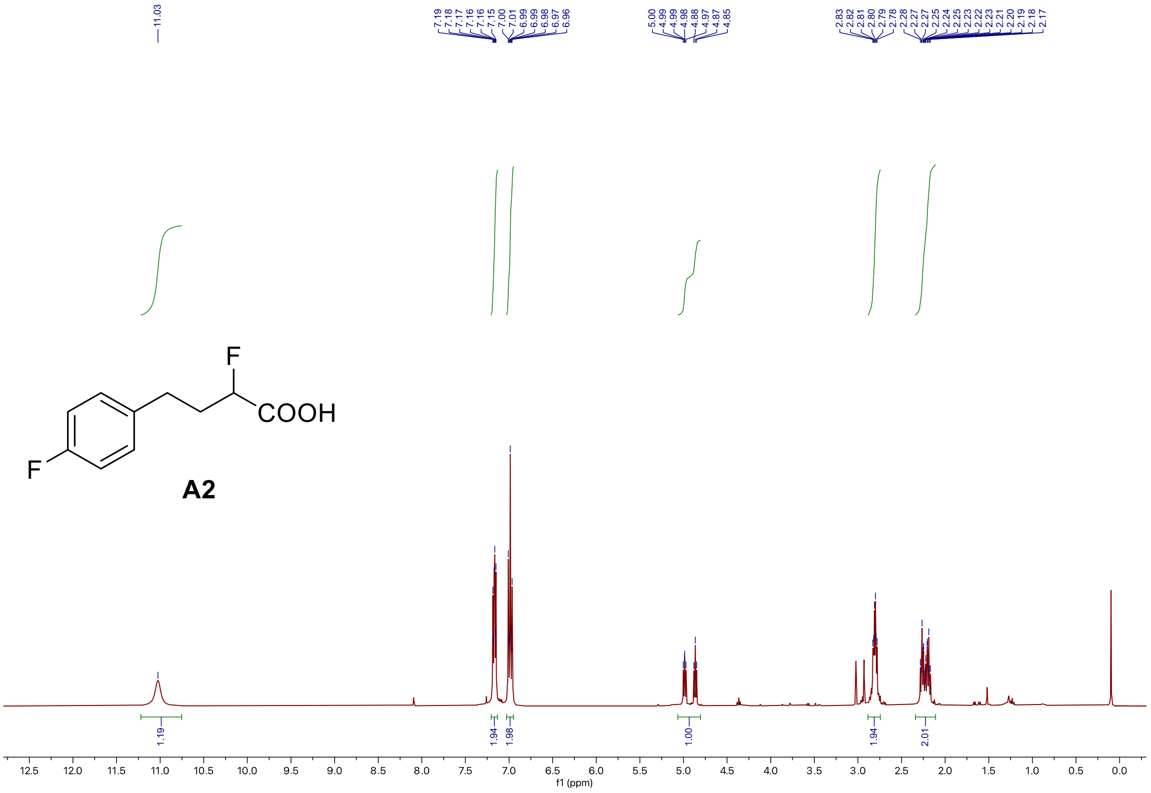

A2

^{19}F NMR (376 MHz, CDCl$_3$)

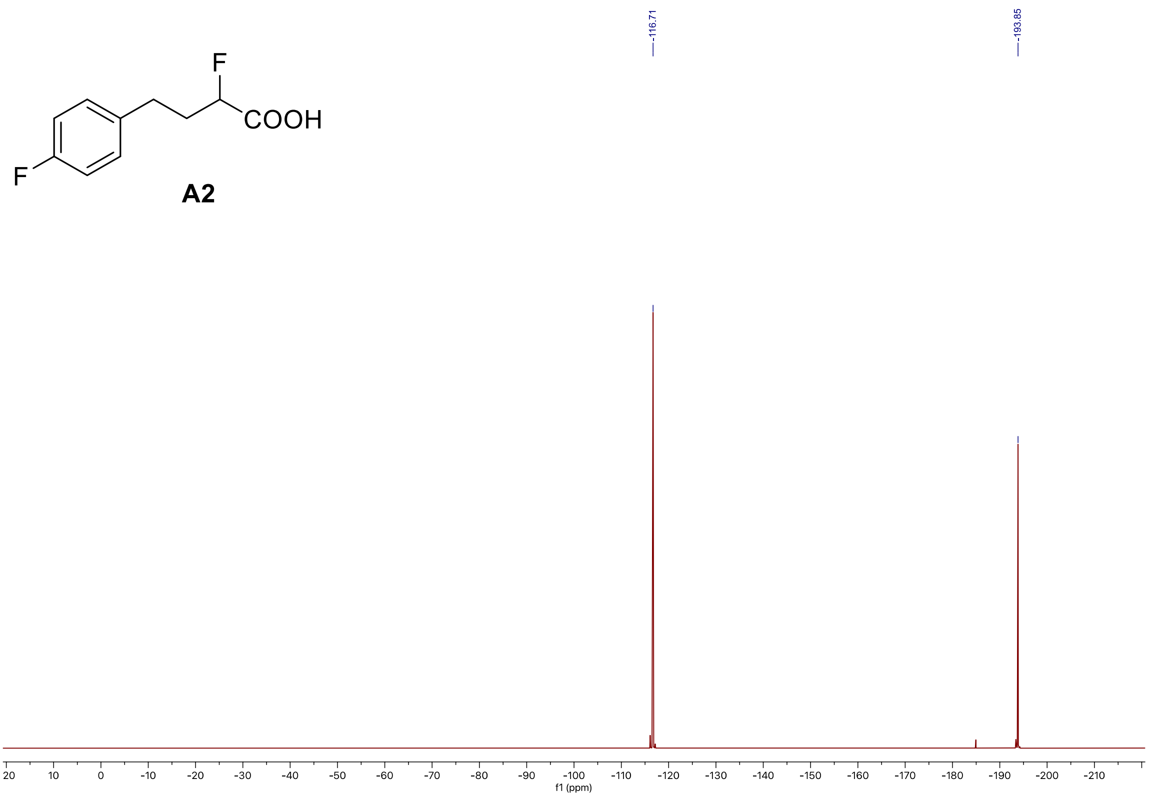

^{13}C NMR (101 MHz, CDCl$_3$)

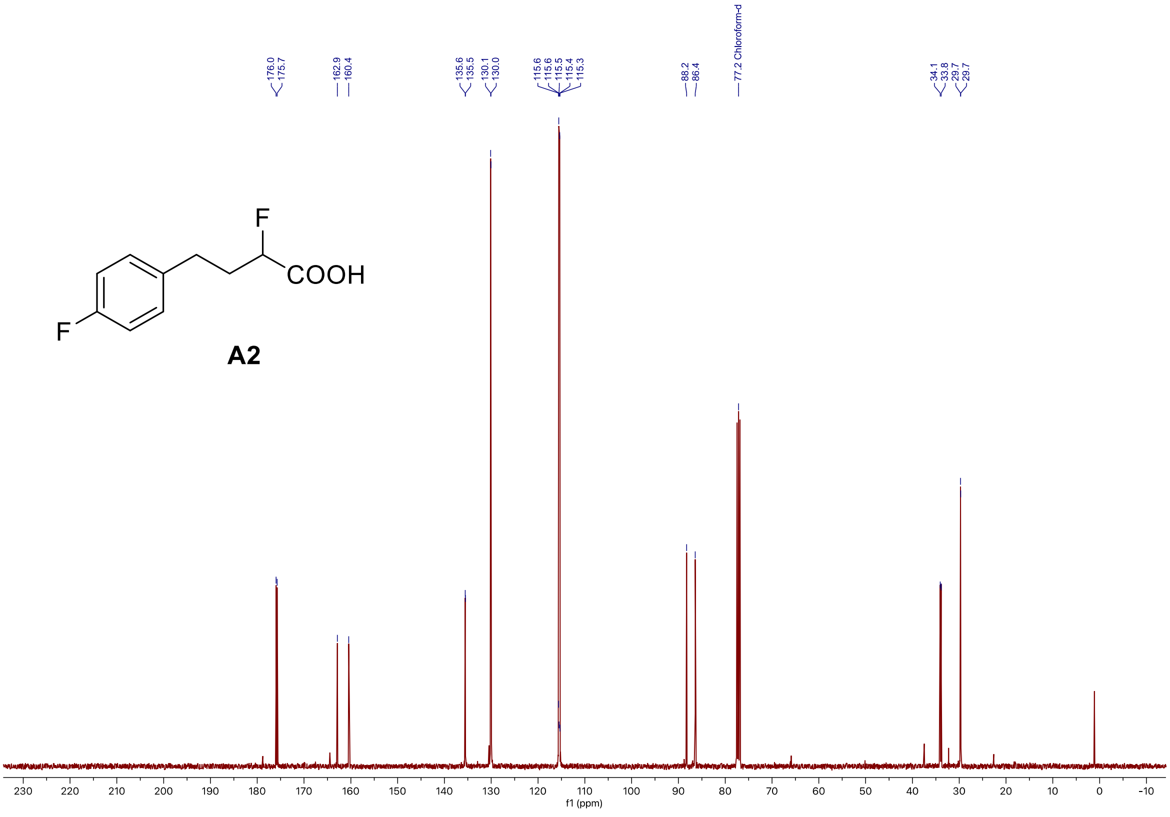

^{1}H NMR (400 MHz, $CDCl_3$)

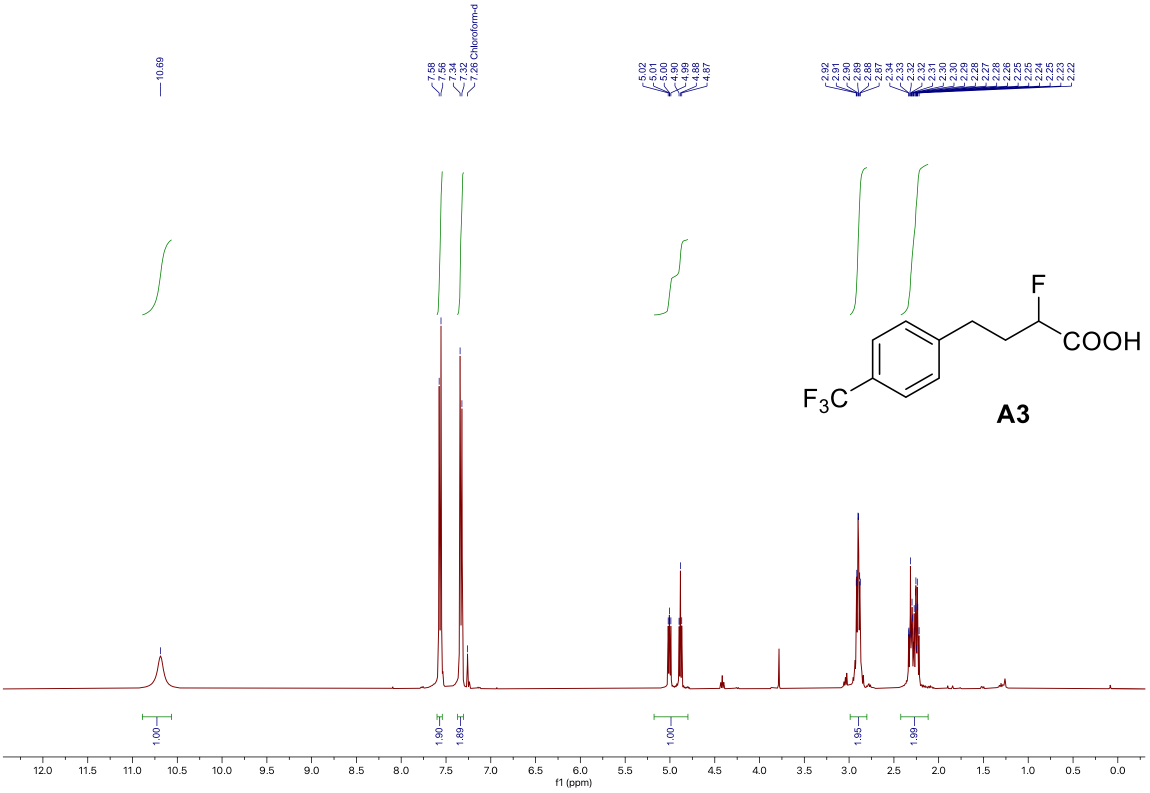

^{19}F NMR (376 MHz, $CDCl_3$)

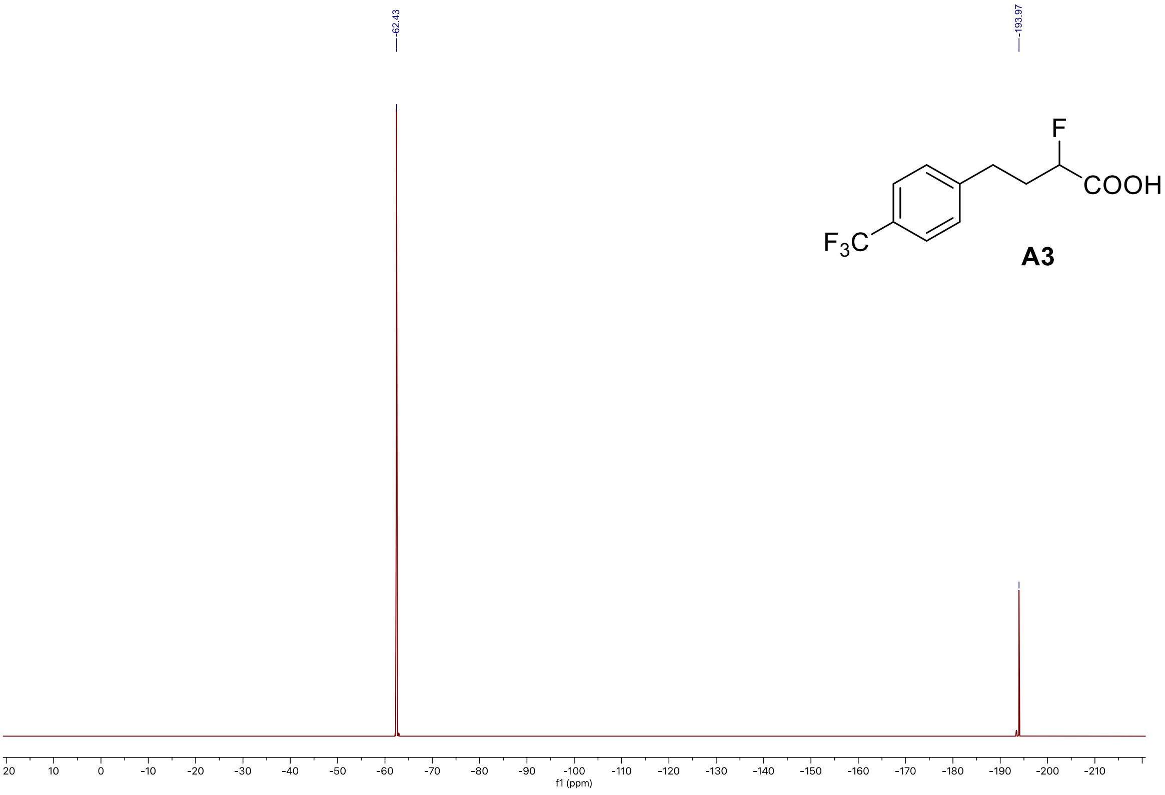

^{13}C NMR (101 MHz, CDCl$_3$)

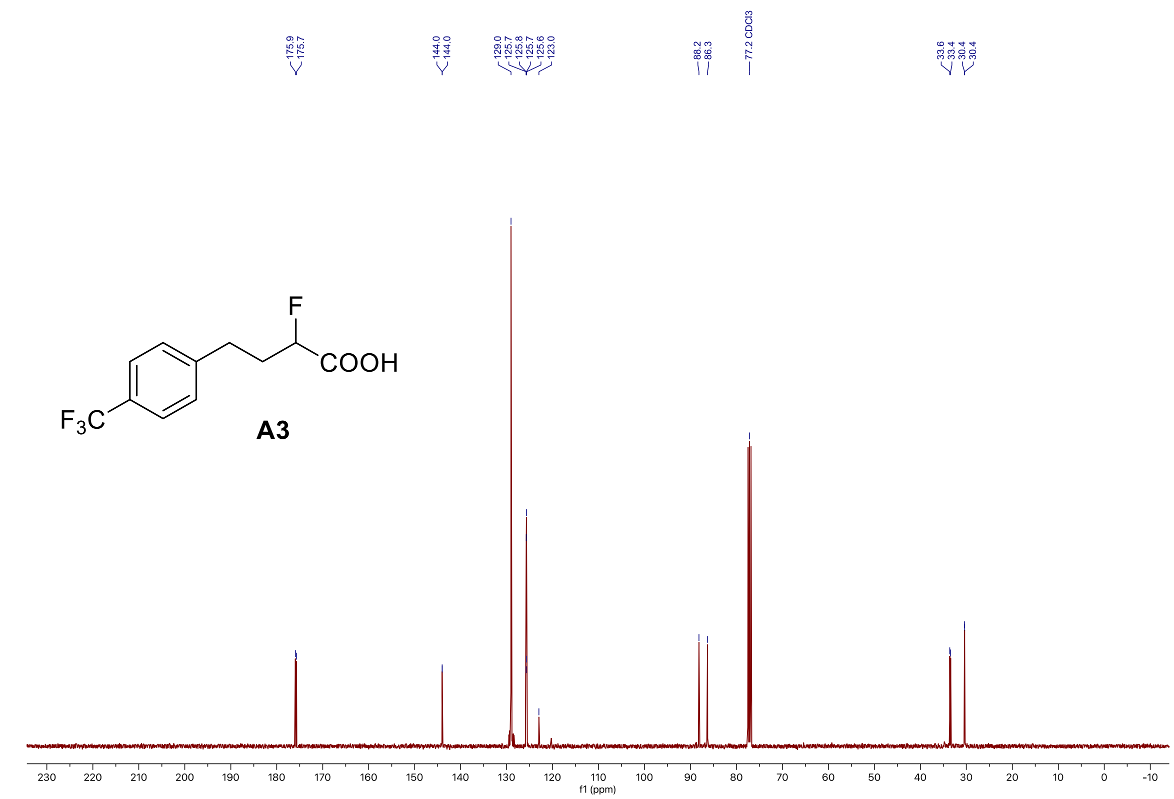

A3

^{1}H NMR (300 MHz, CDCl$_3$)

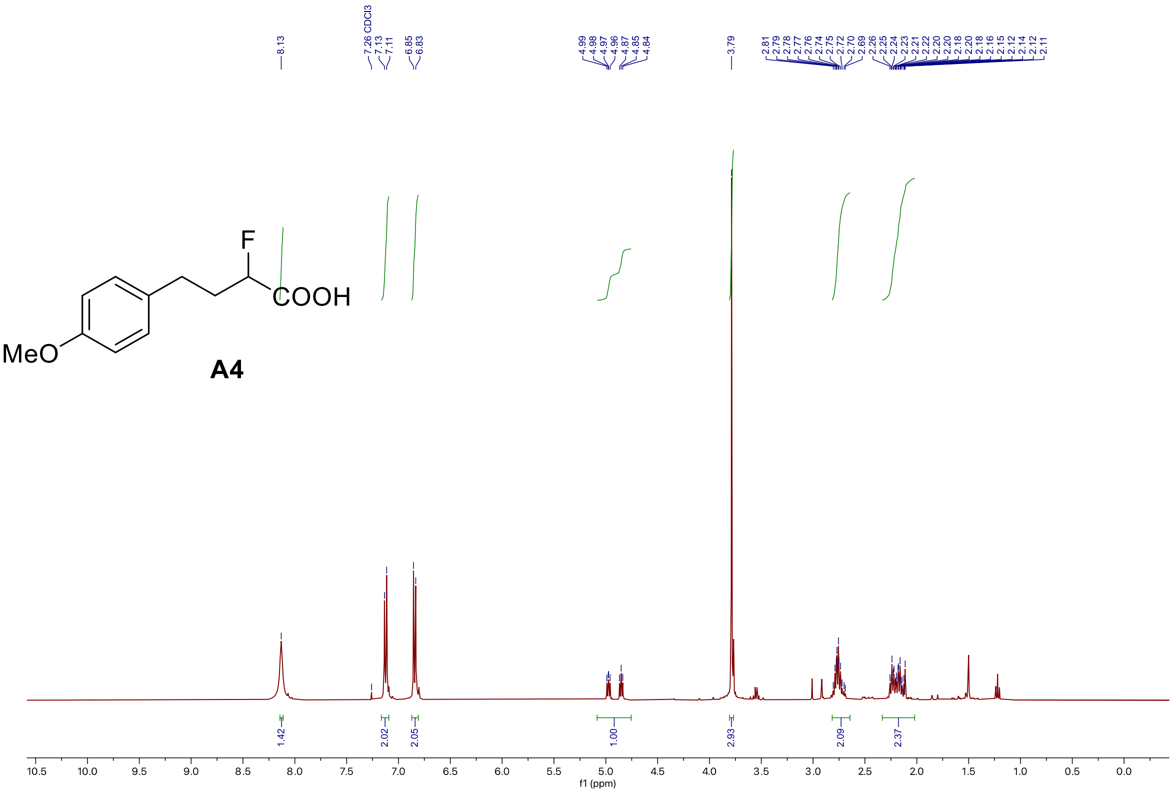

A4

^{19}F NMR (376 MHz, $CDCl_3$)

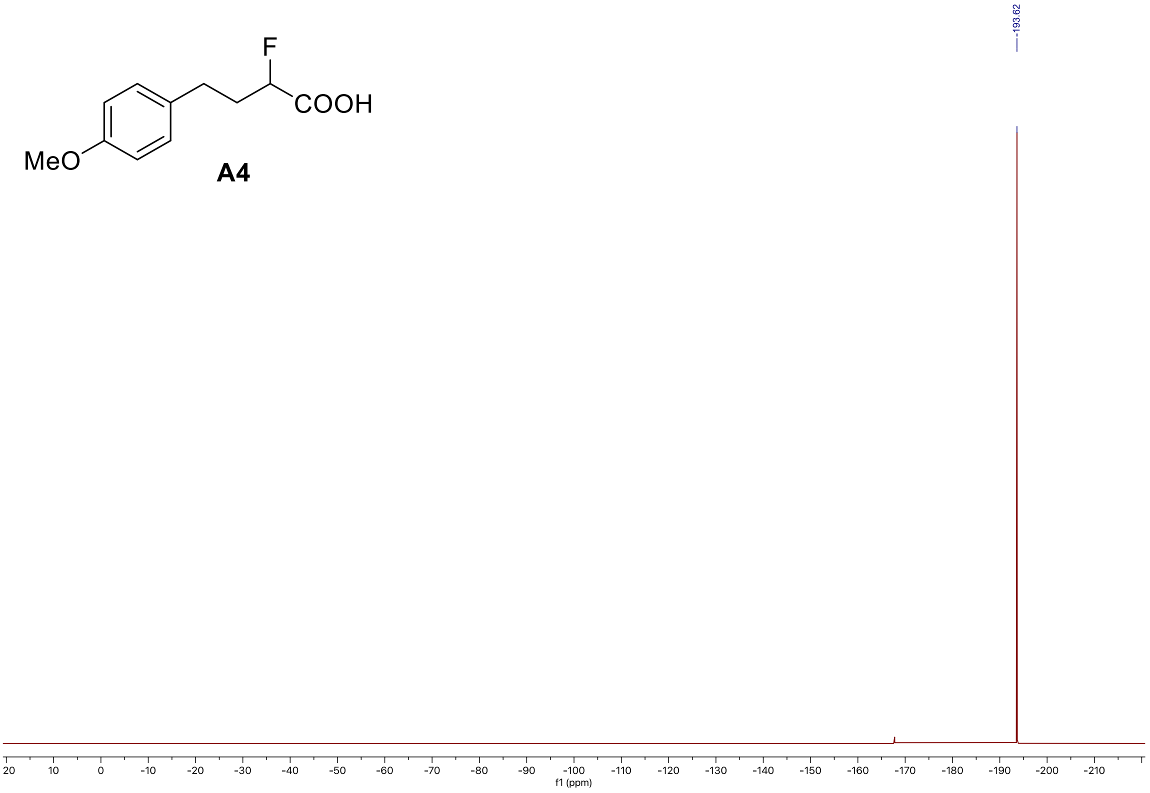

^{13}C NMR (75 MHz, $CDCl_3$)

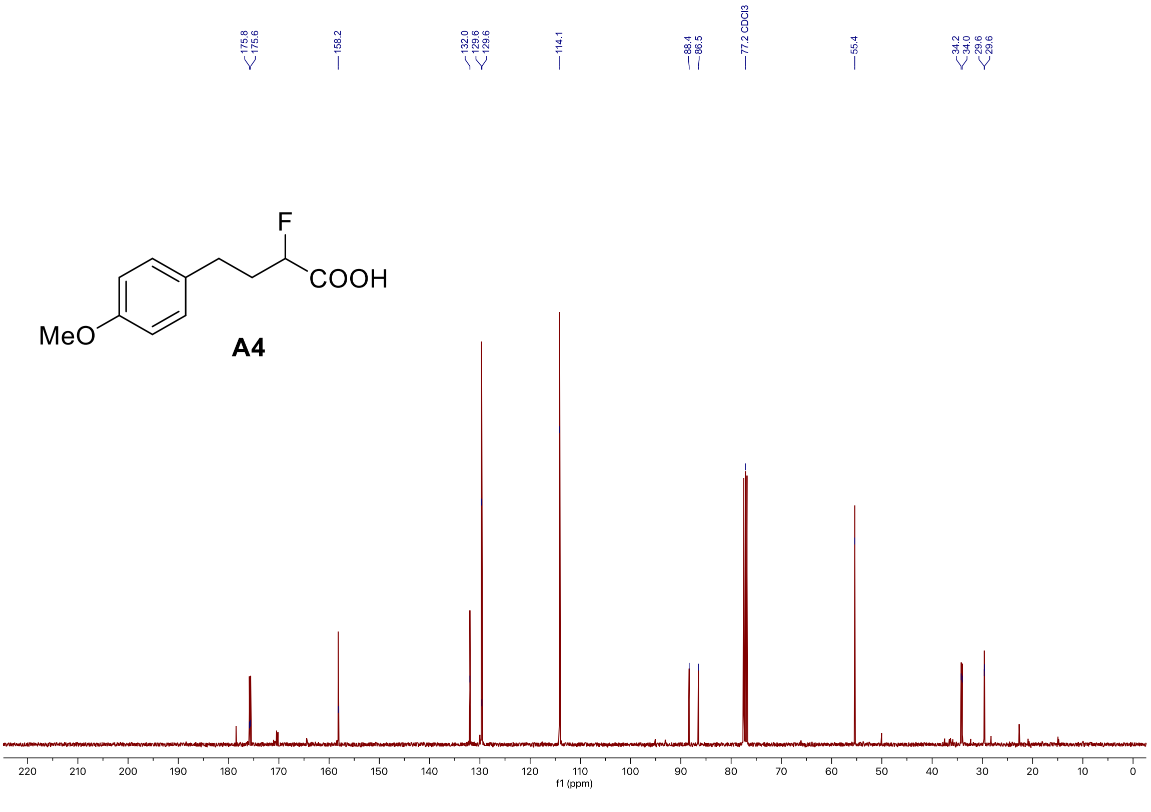

^{1}H NMR (400 MHz, CDCl$_3$)

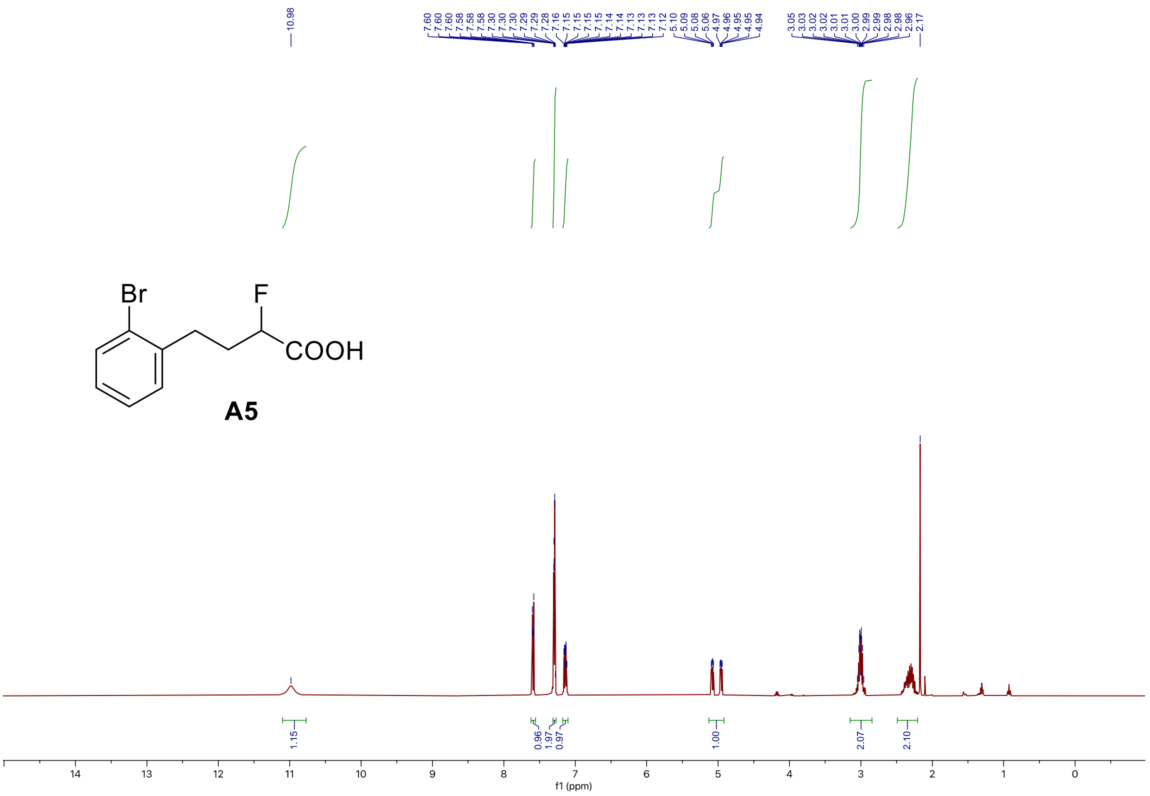

^{19}F NMR (376 MHz, CDCl$_3$)

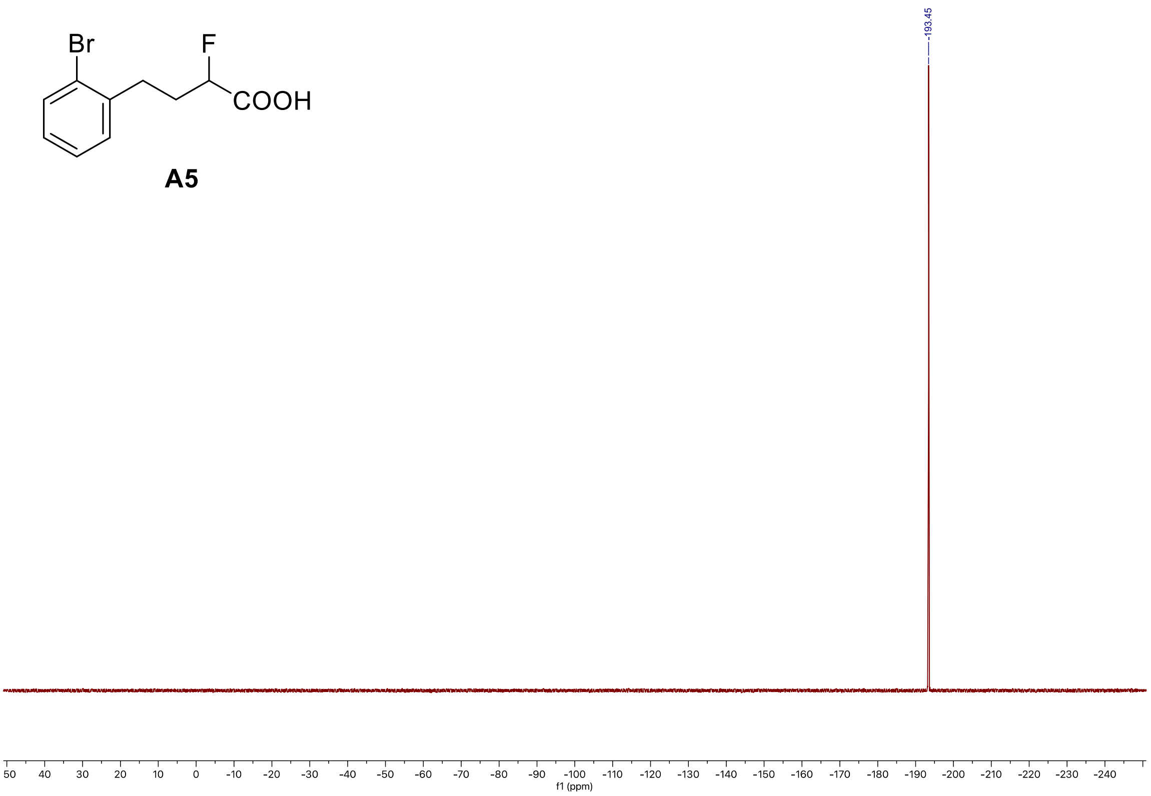

^{13}C NMR (101 MHz, CDCl$_3$)

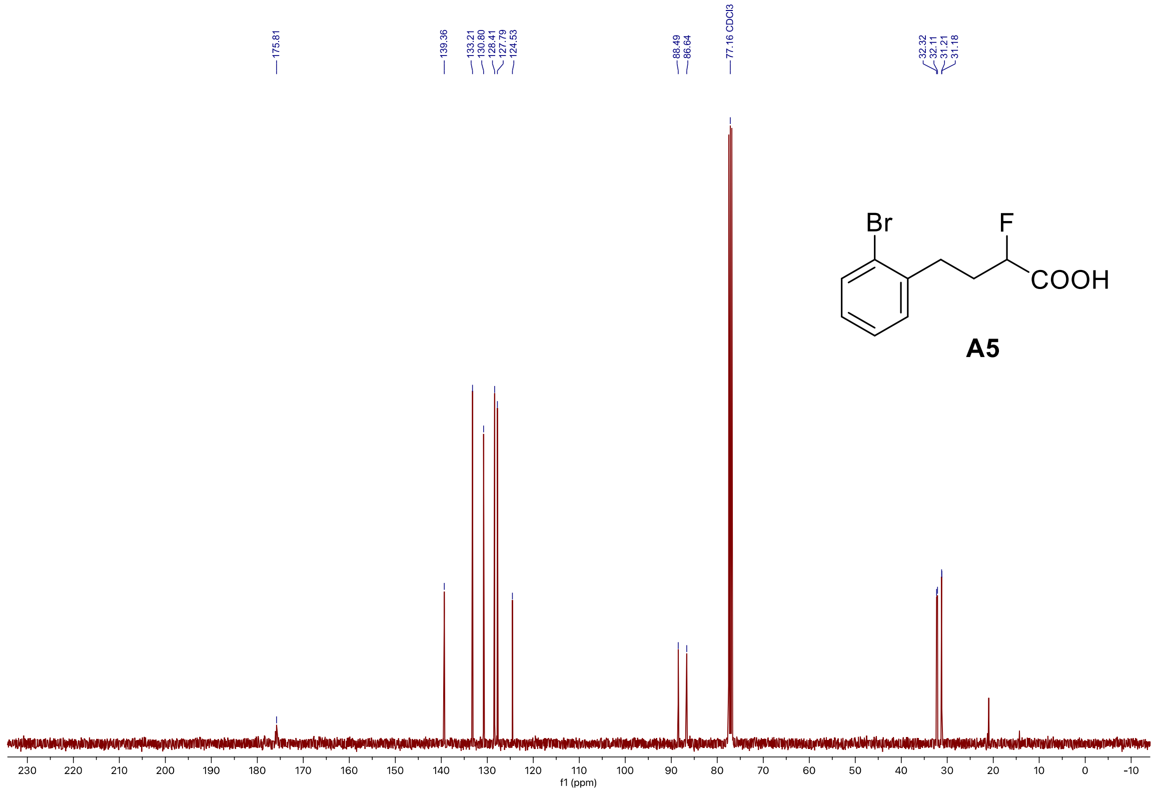

^{1}H NMR (400 MHz, CDCl$_3$)

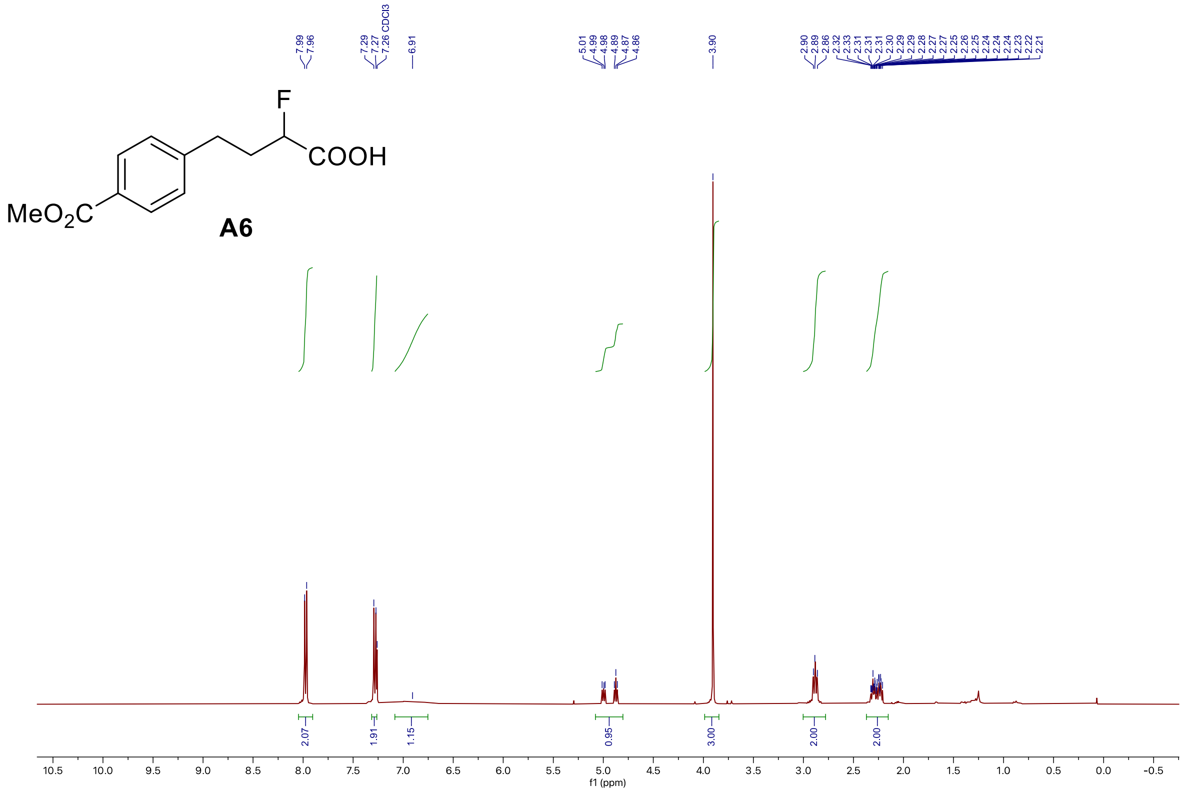

^{19}F NMR (376 MHz, $CDCl_3$)

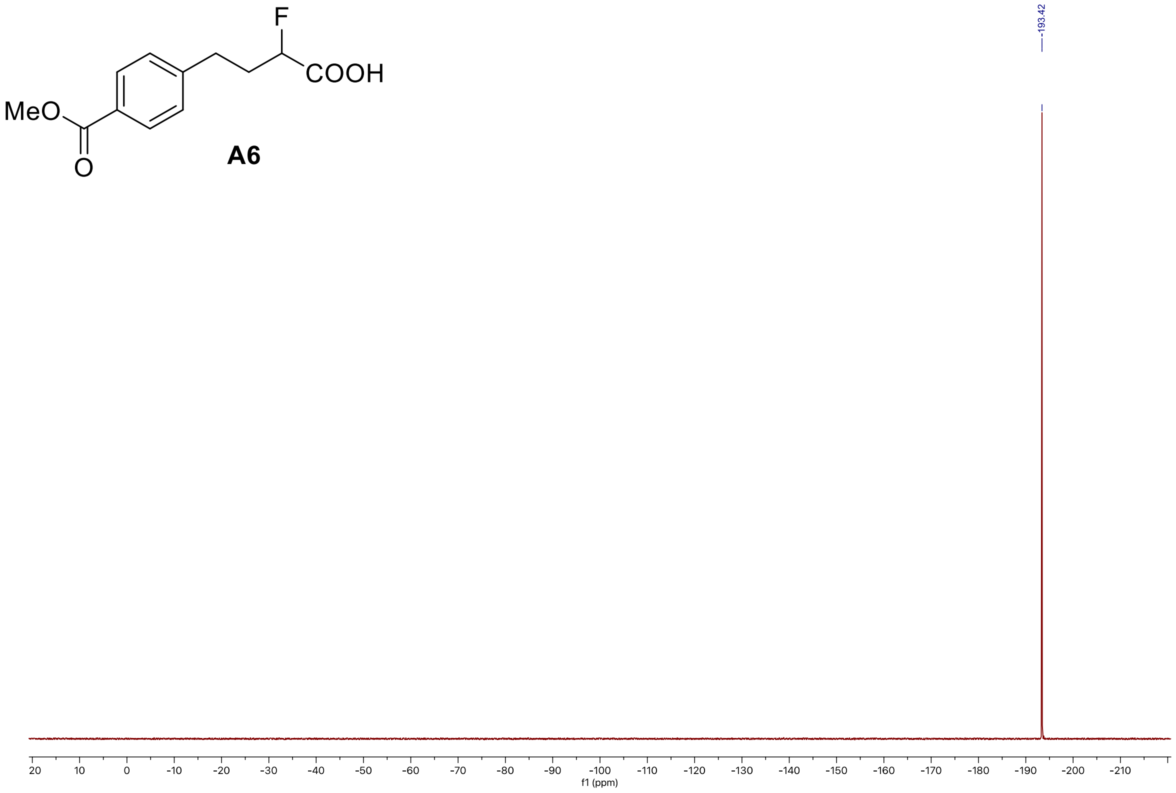

^{13}C NMR (101 MHz, $CDCl_3$)

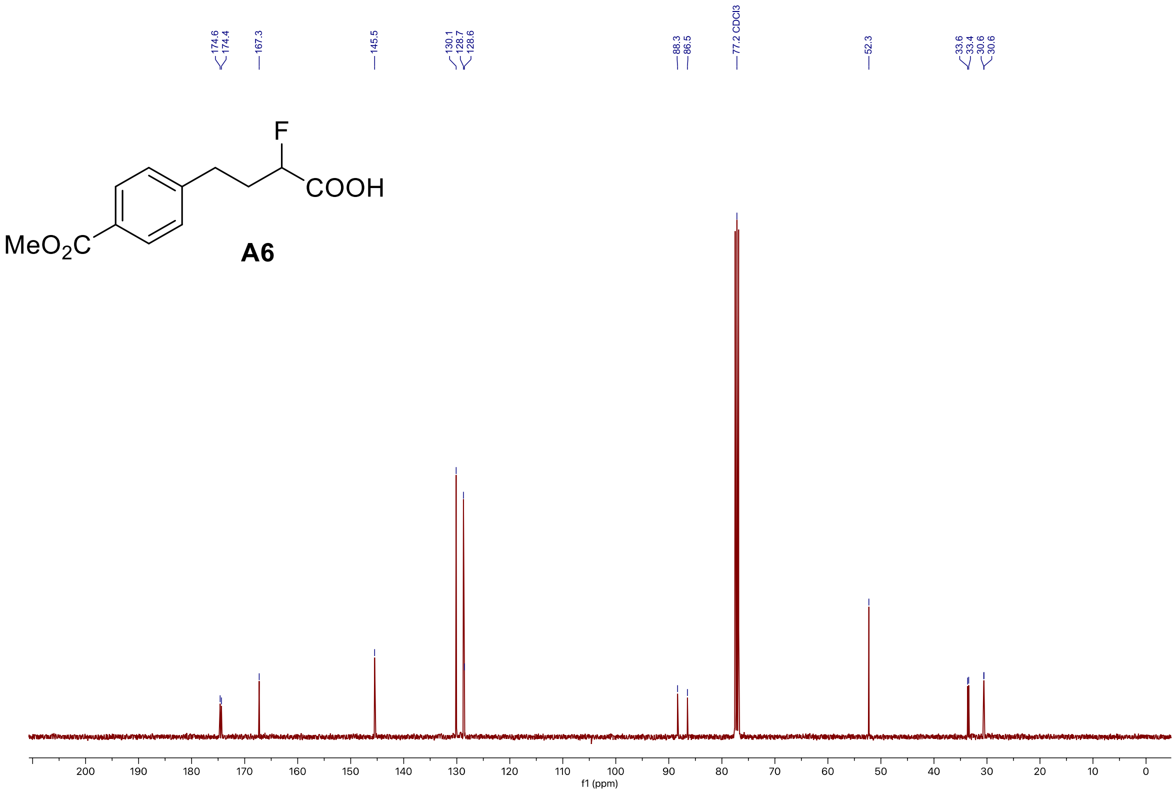

^{1}H NMR (400 MHz, CDCl$_3$)

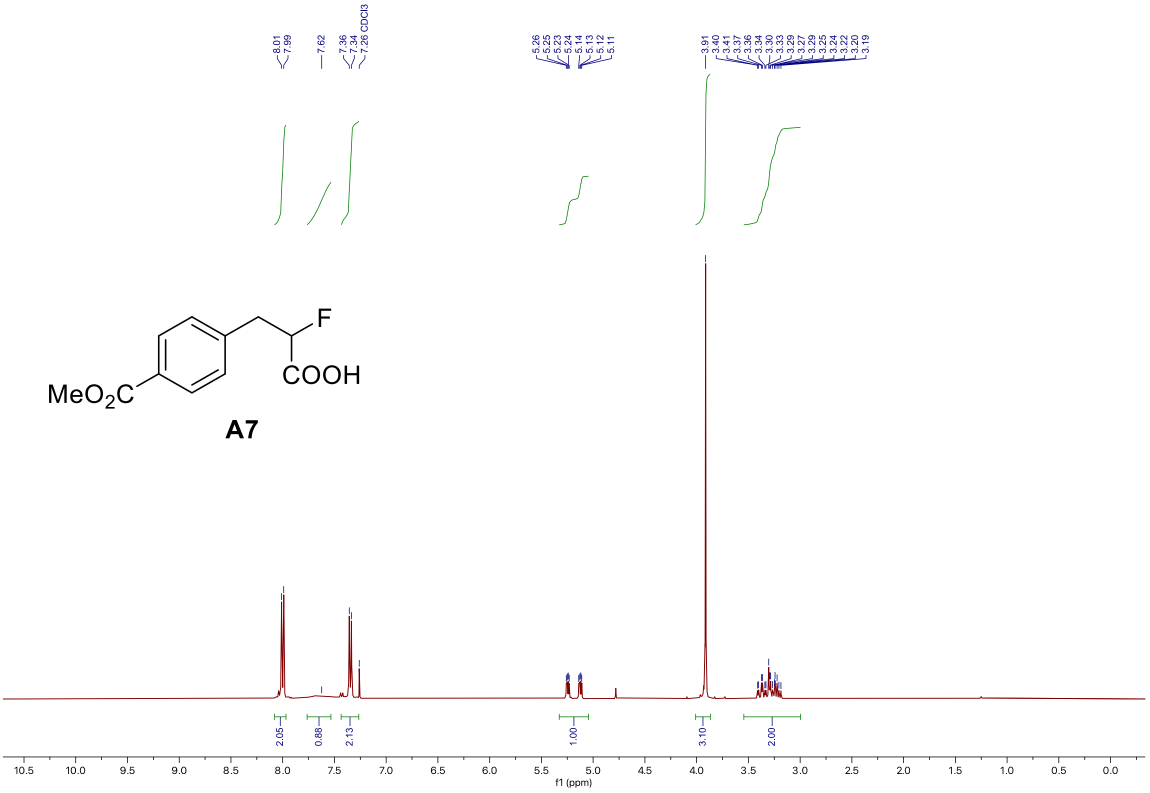

^{19}F NMR (376 MHz, CDCl$_3$)

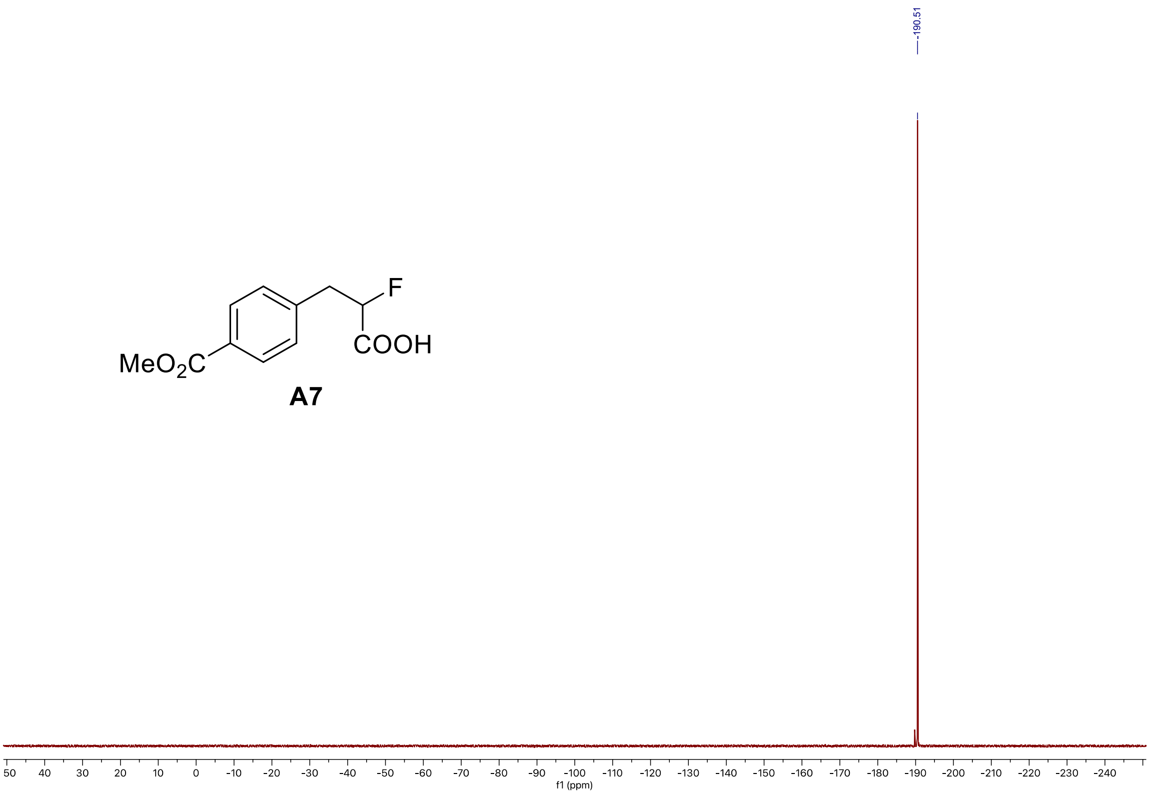

^{13}C NMR (101 MHz, $CDCl_3$)

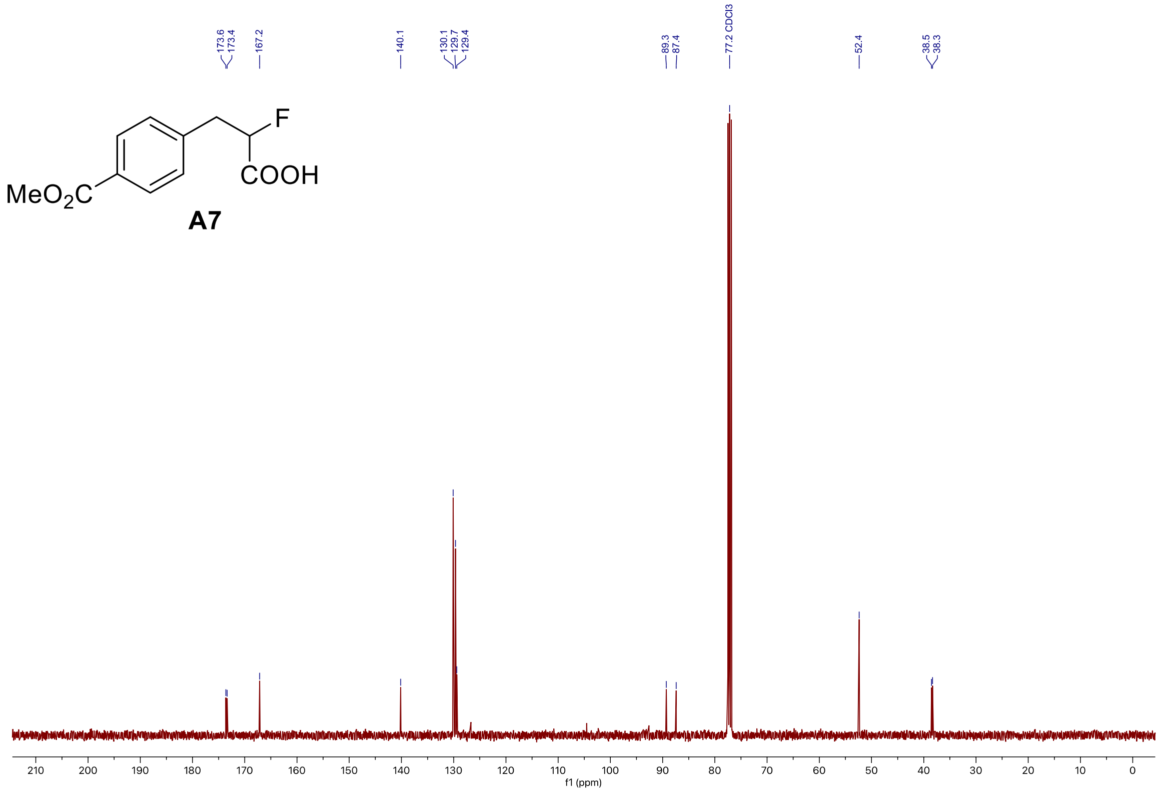

^{1}H NMR (400 MHz, $CDCl_3$)

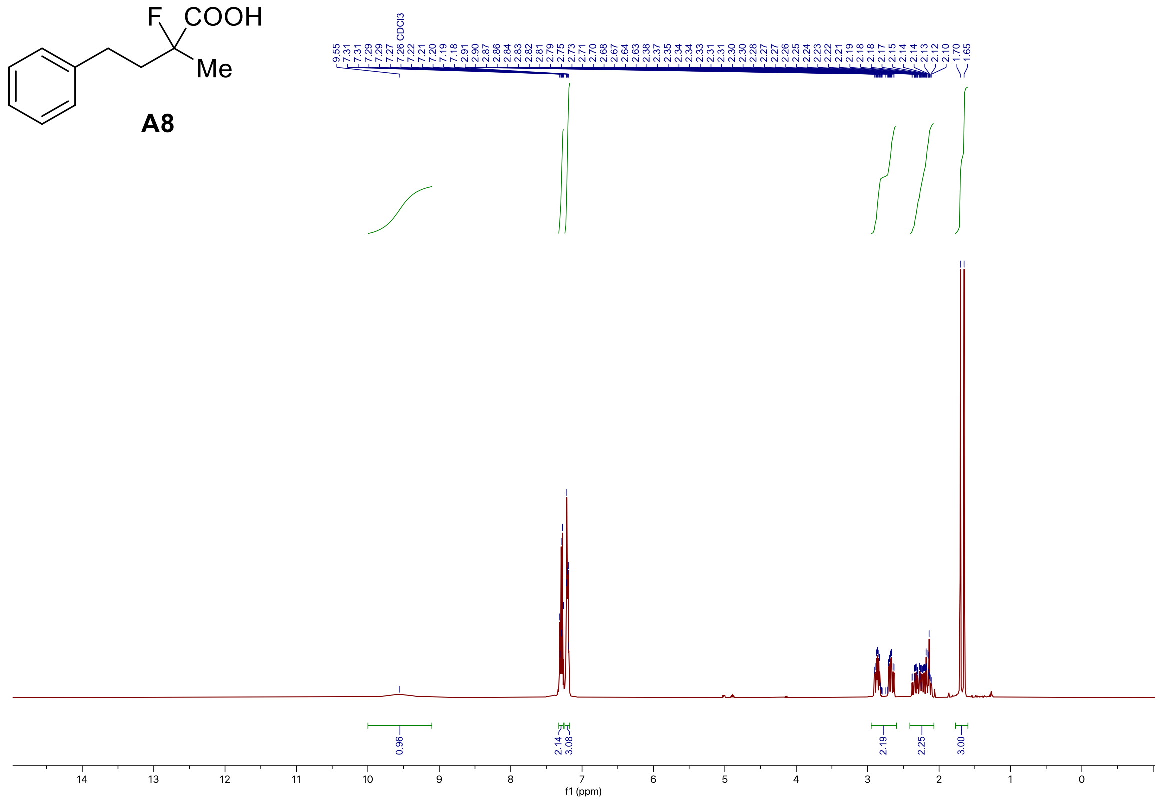

^{19}F NMR (376 MHz, $CDCl_3$)

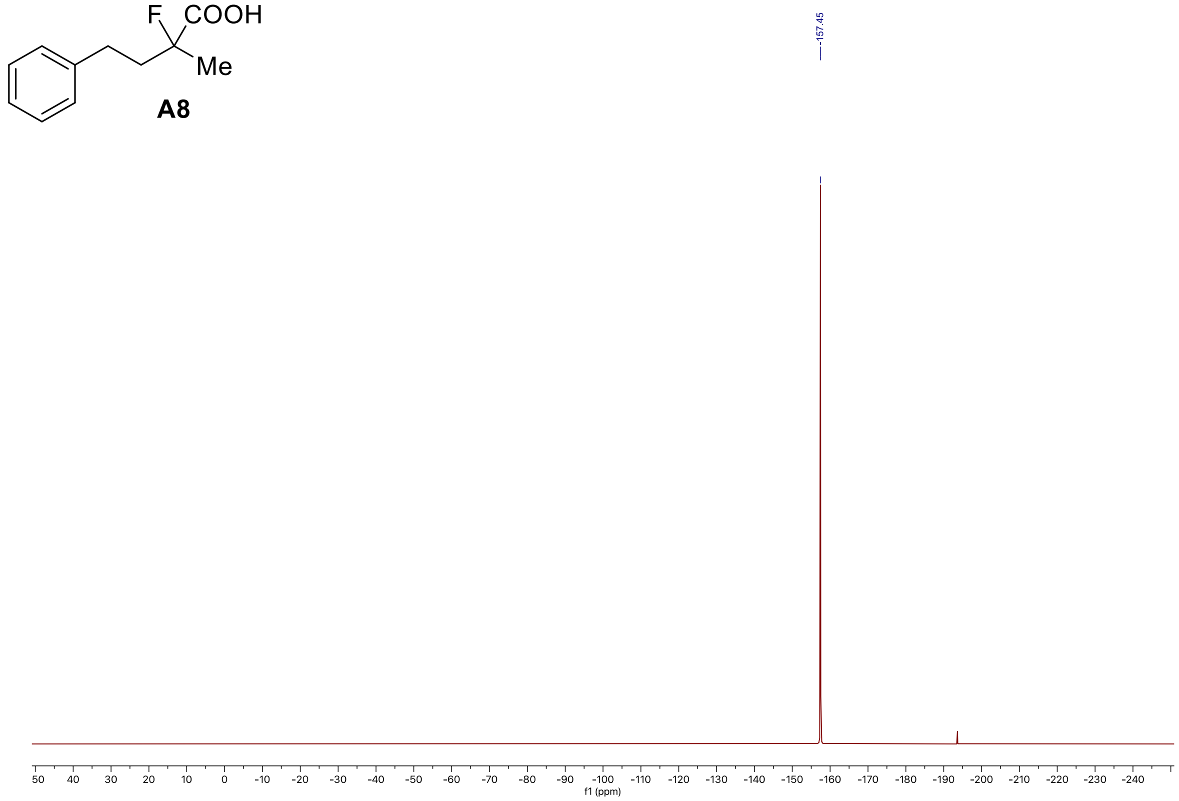

^{13}C NMR (101 MHz, $CDCl_3$)

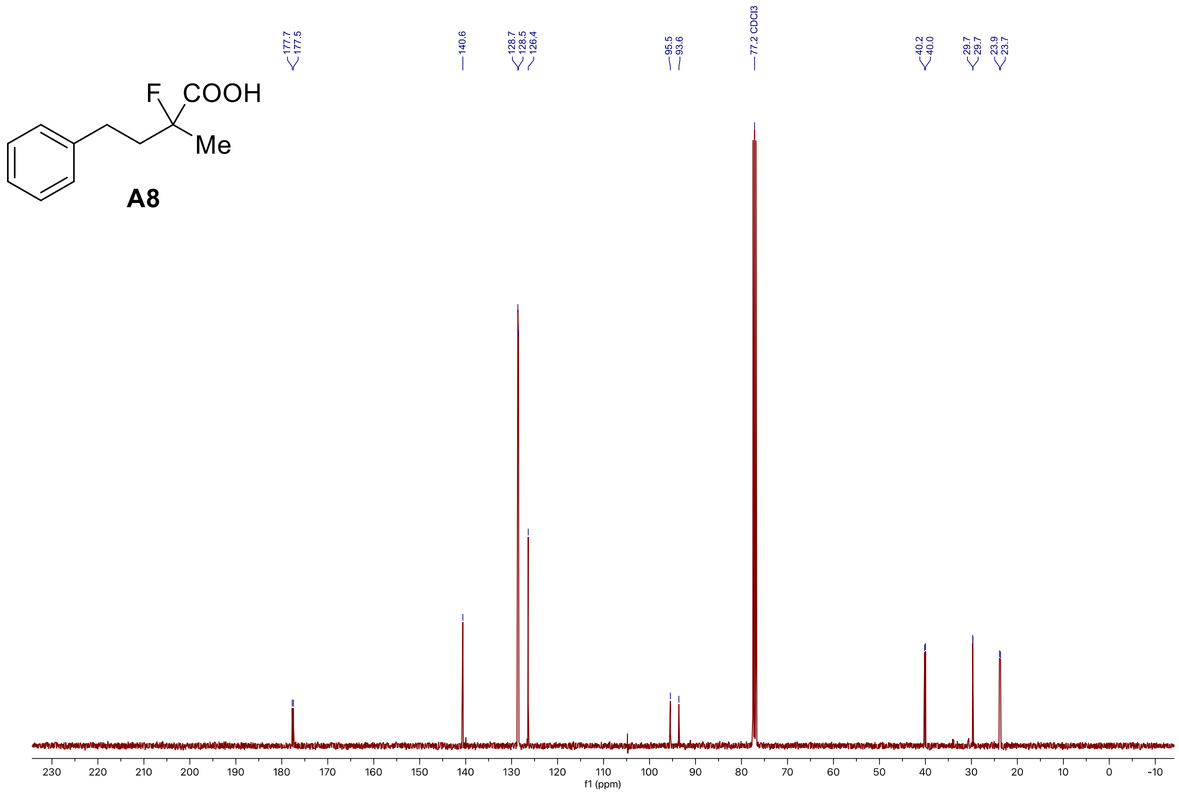

^{1}H NMR (400 MHz, $CDCl_3$)

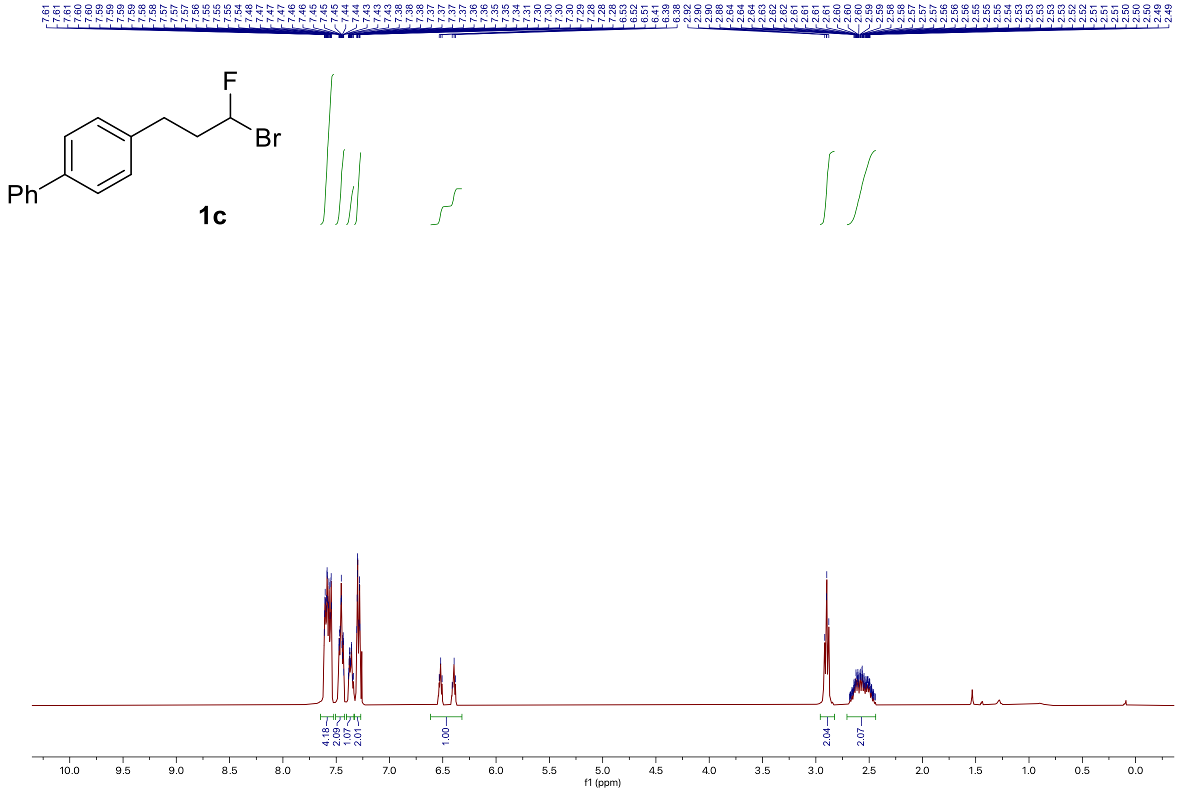

^{19}F NMR (376 MHz, $CDCl_3$)

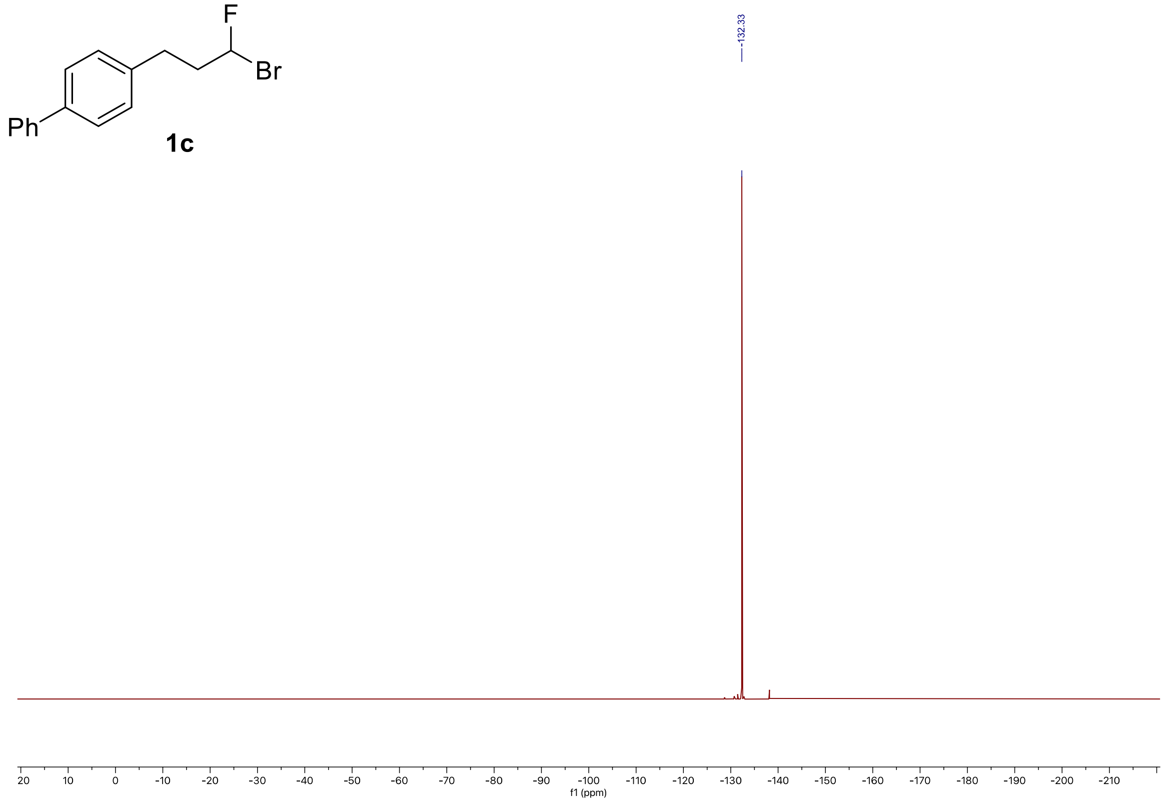

^{13}C NMR (101 MHz, $CDCl_3$)

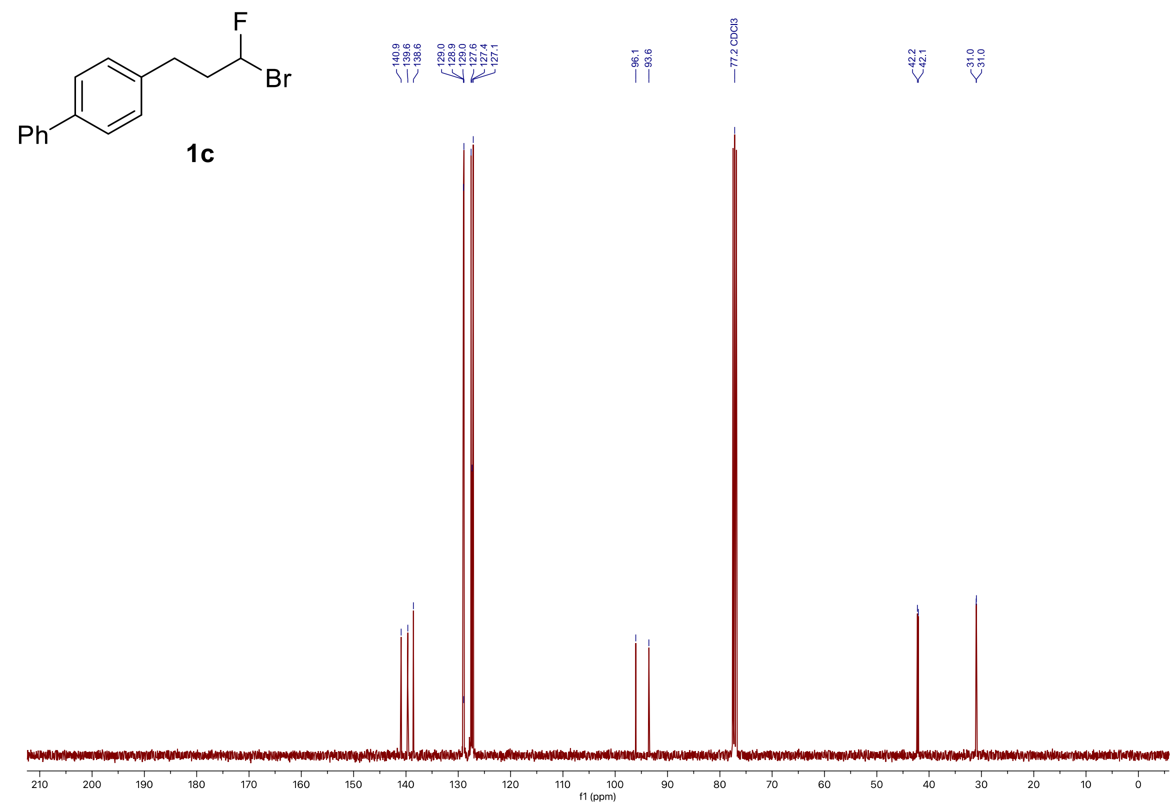

^{1}H NMR (400 MHz, $CDCl_3$)

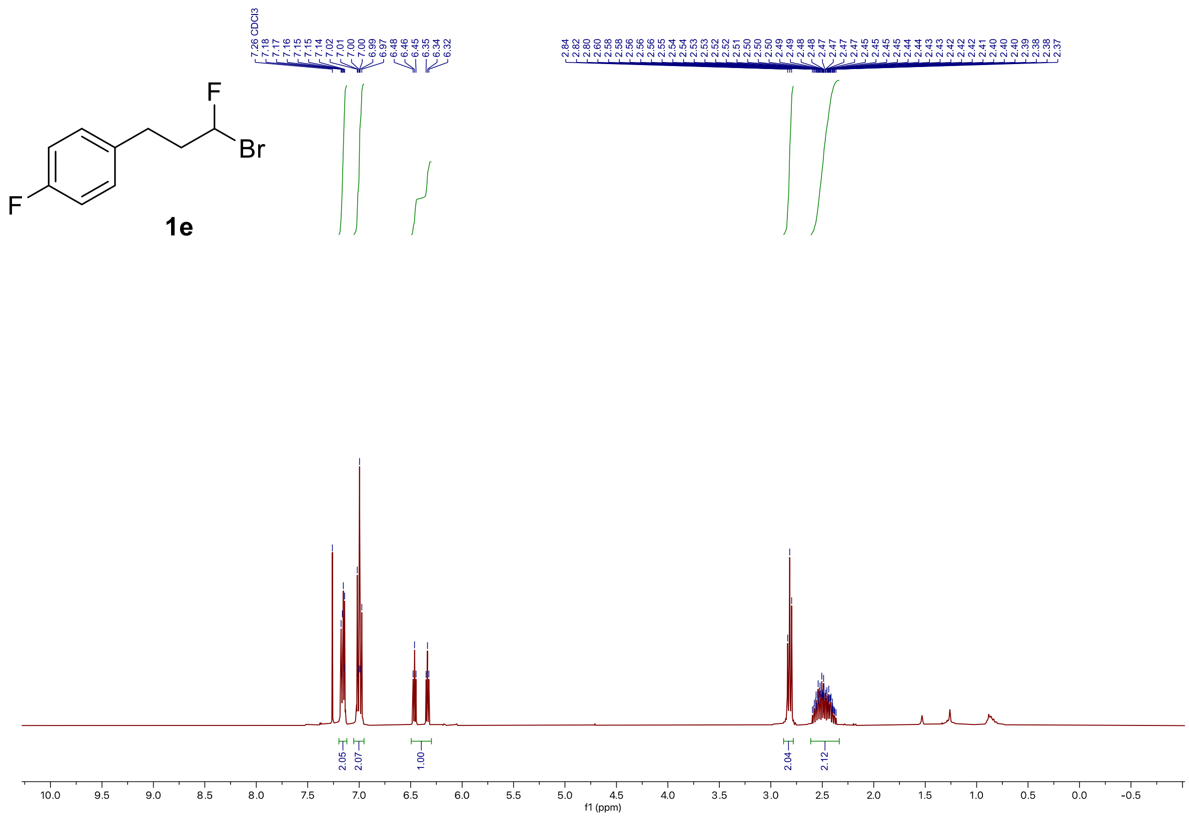

^{19}F NMR (376 MHz, CDCl$_3$)

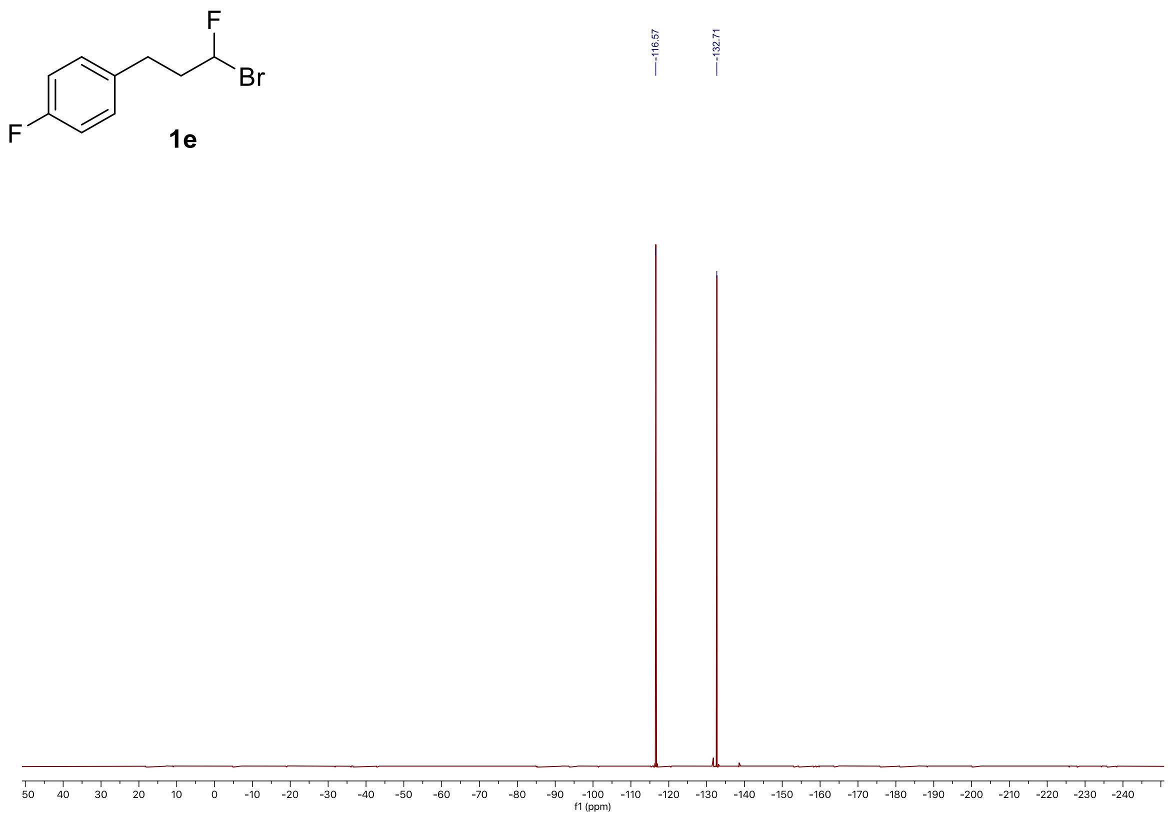

^{13}C NMR (101 MHz, CDCl$_3$)

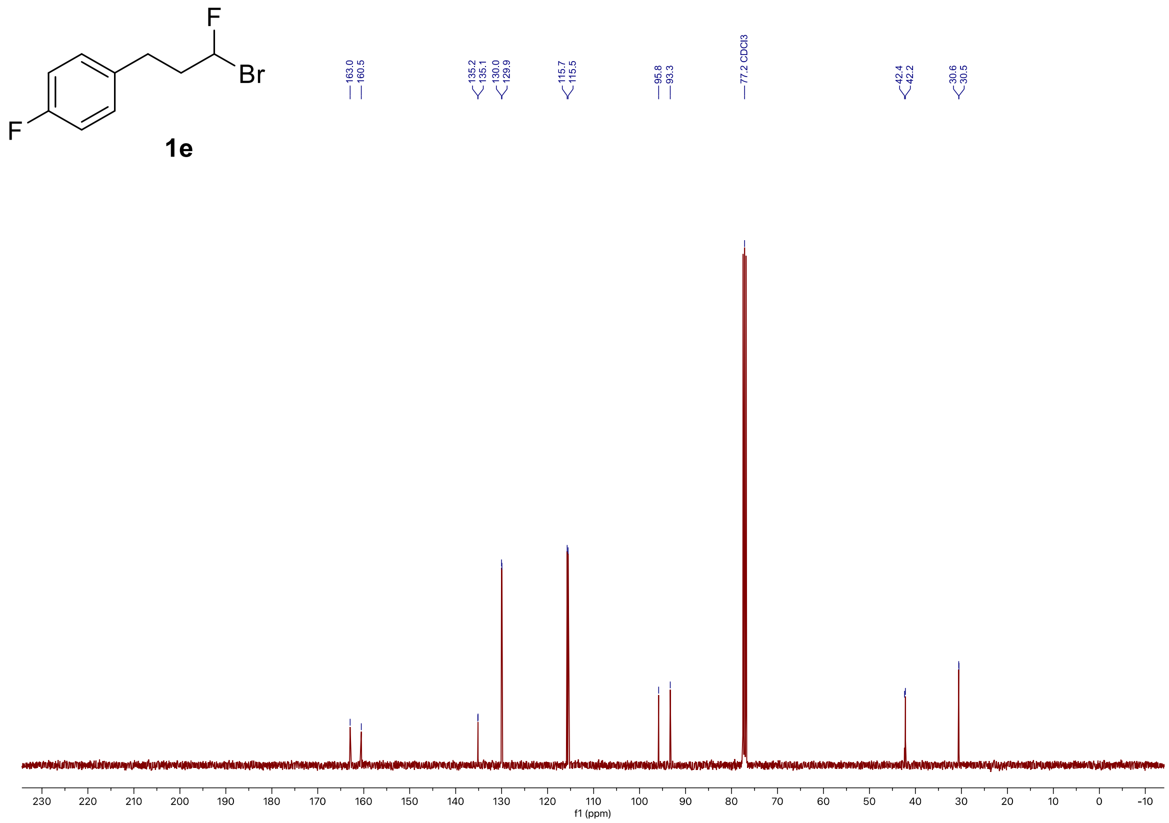

^{1}H NMR (400 MHz, $CDCl_3$)

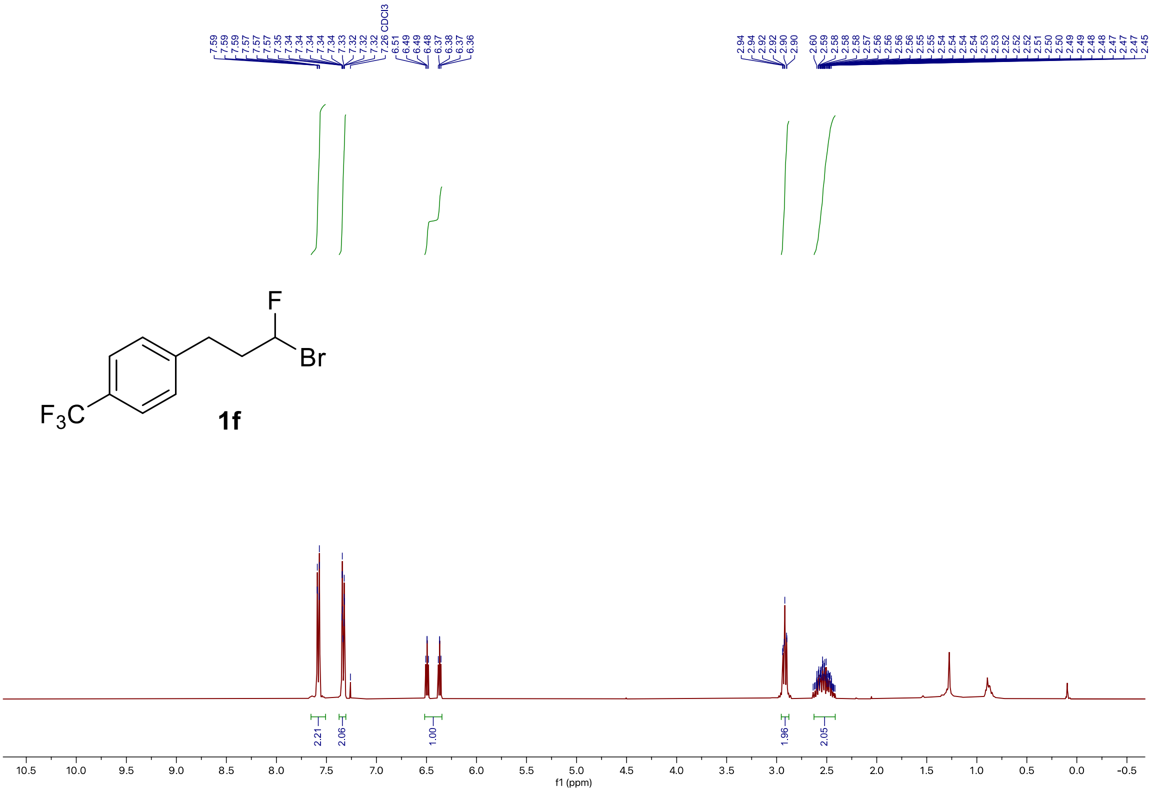

^{19}F NMR (376 MHz, $CDCl_3$)

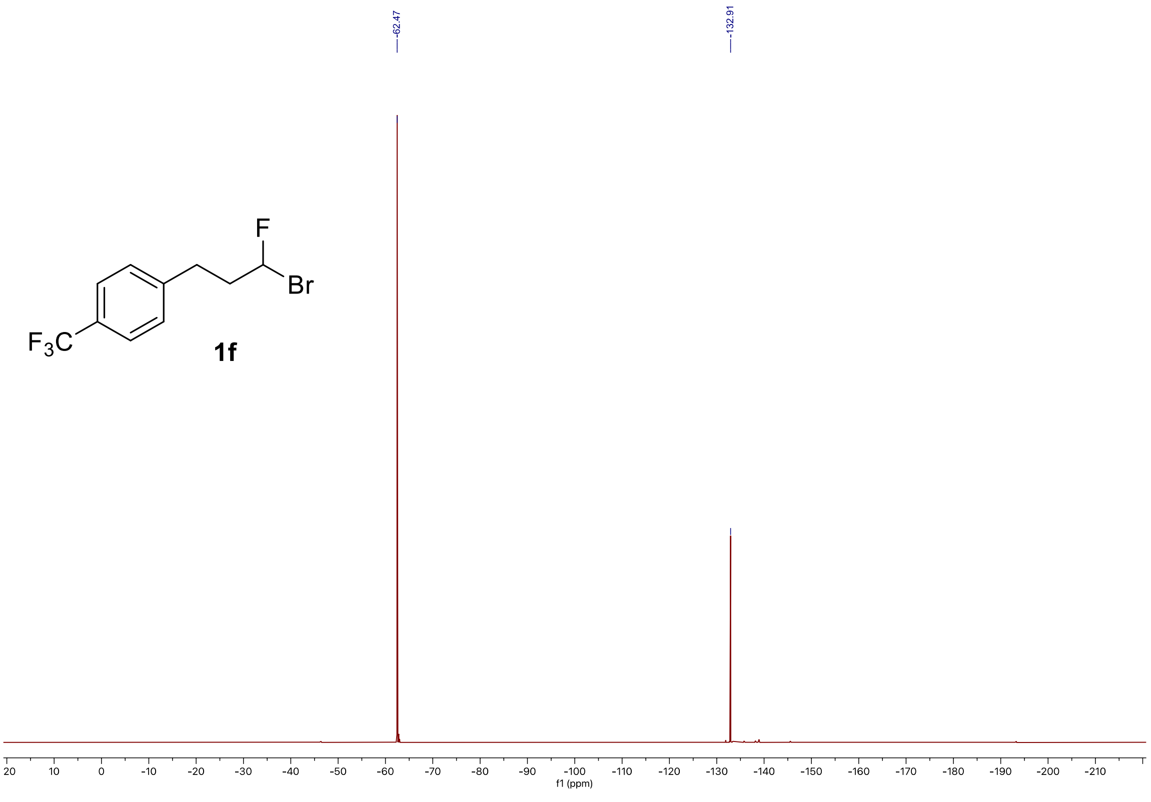

^{13}C NMR (101 MHz, $CDCl_3$)

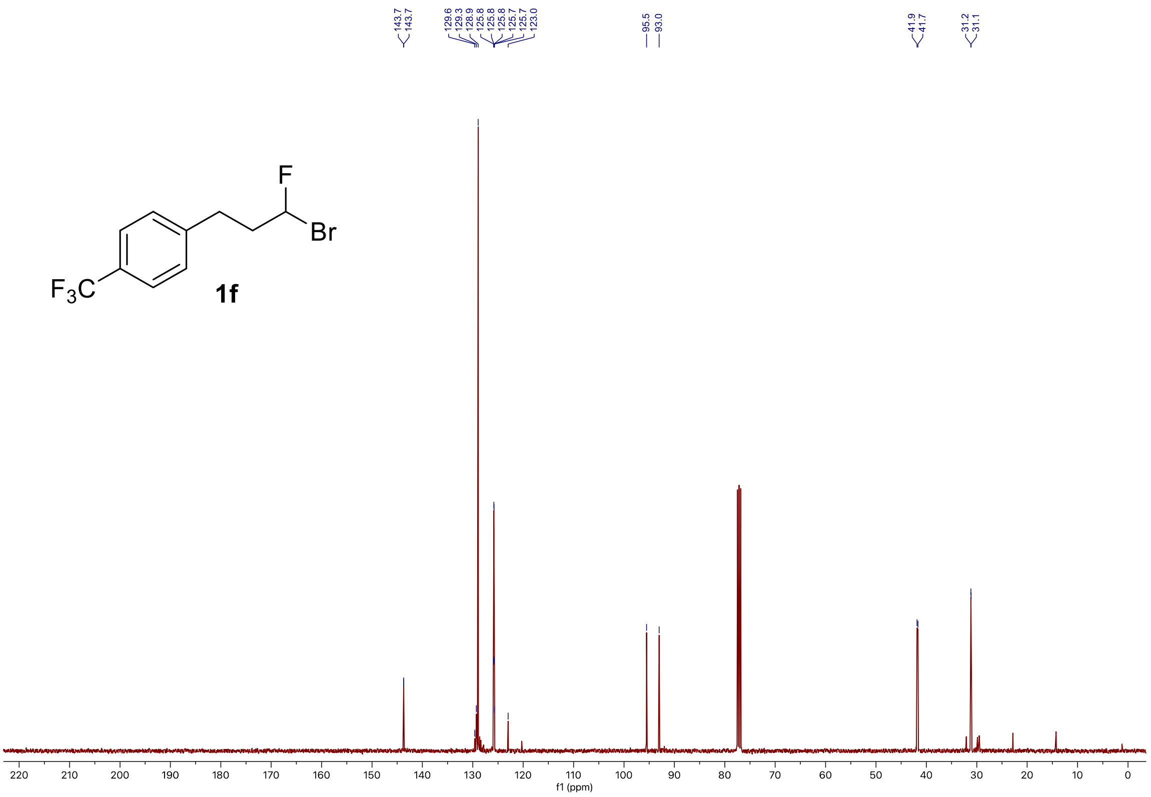

^{1}H NMR (400 MHz, $CDCl_3$)

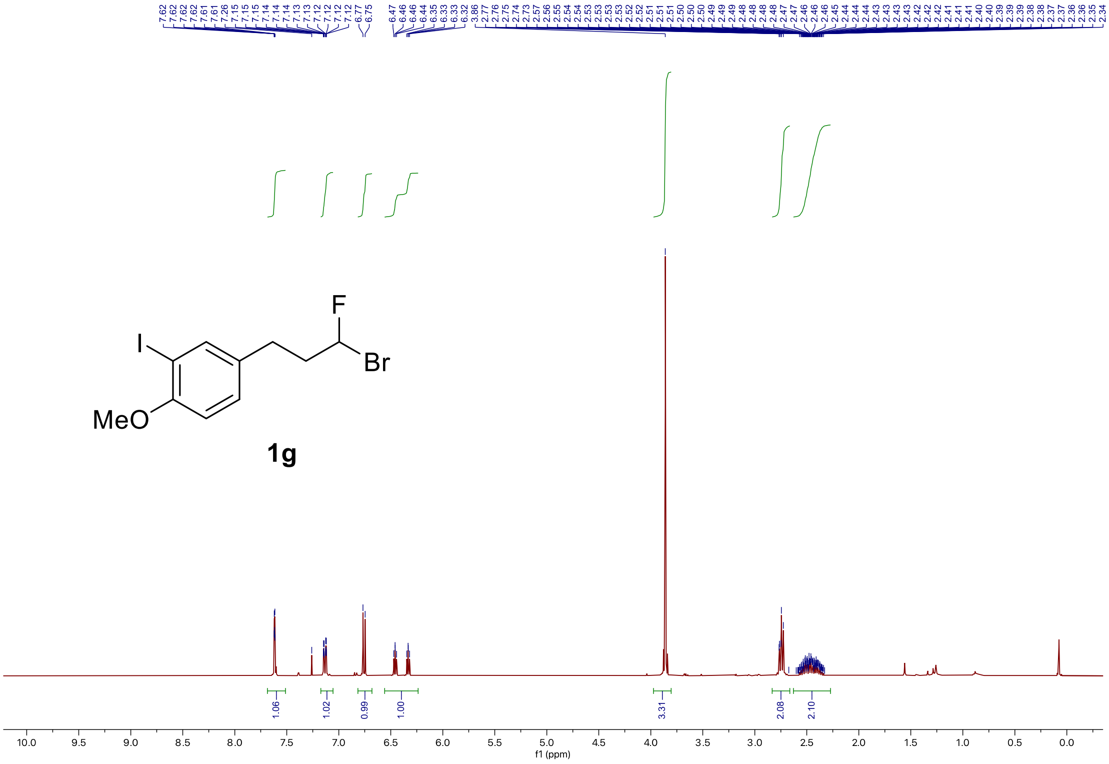

^{19}F NMR (376 MHz, $CDCl_3$)

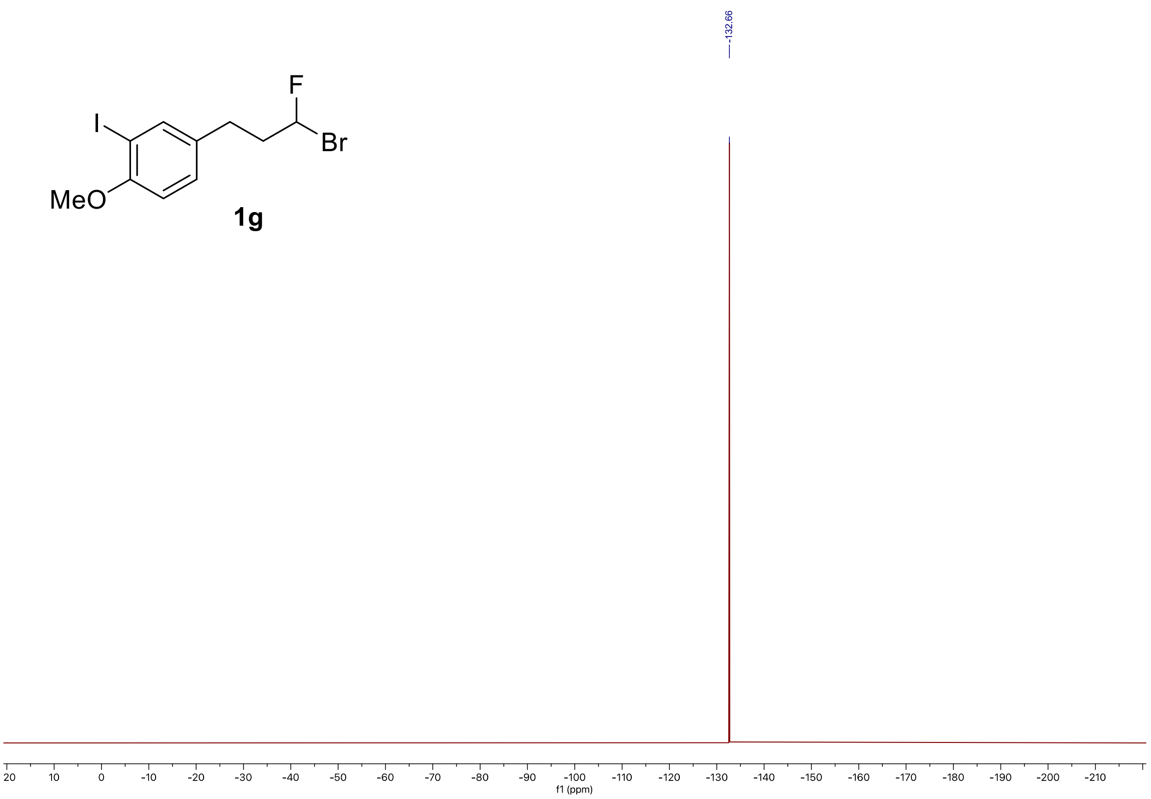

^{13}C NMR (101 MHz, $CDCl_3$)

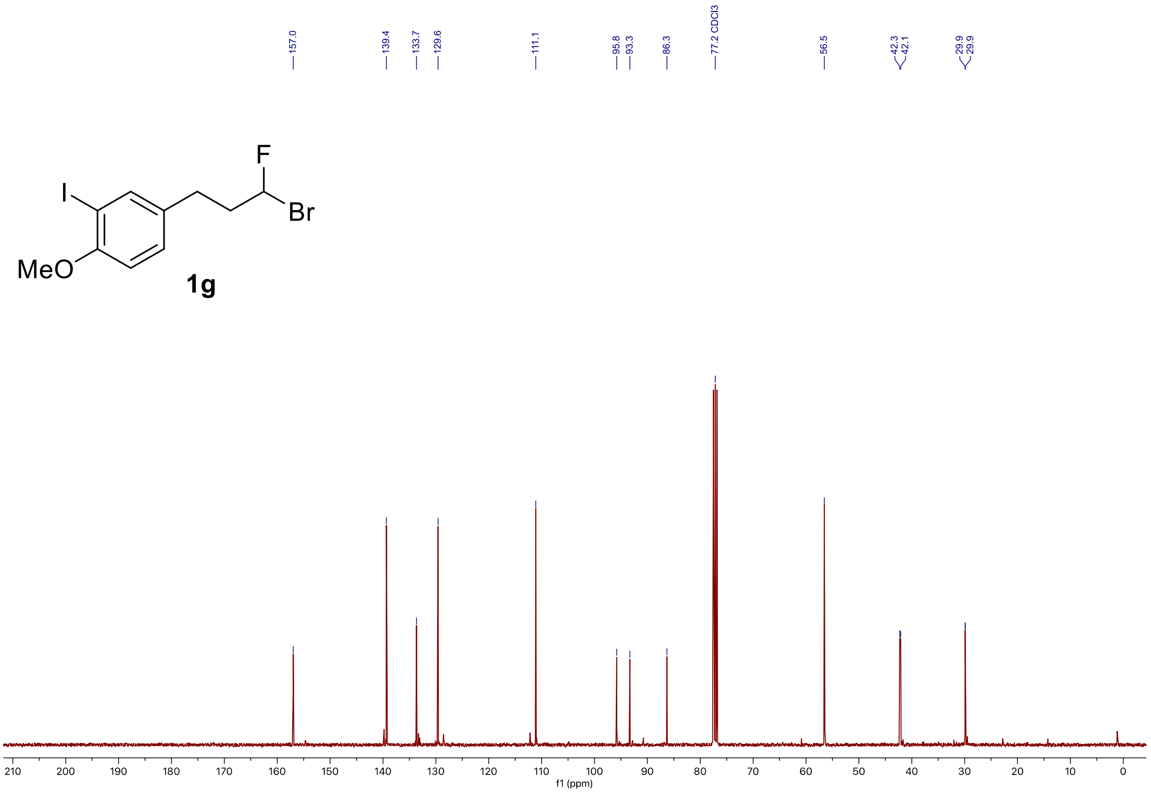

^{1}H NMR (400 MHz, CDCl$_3$)

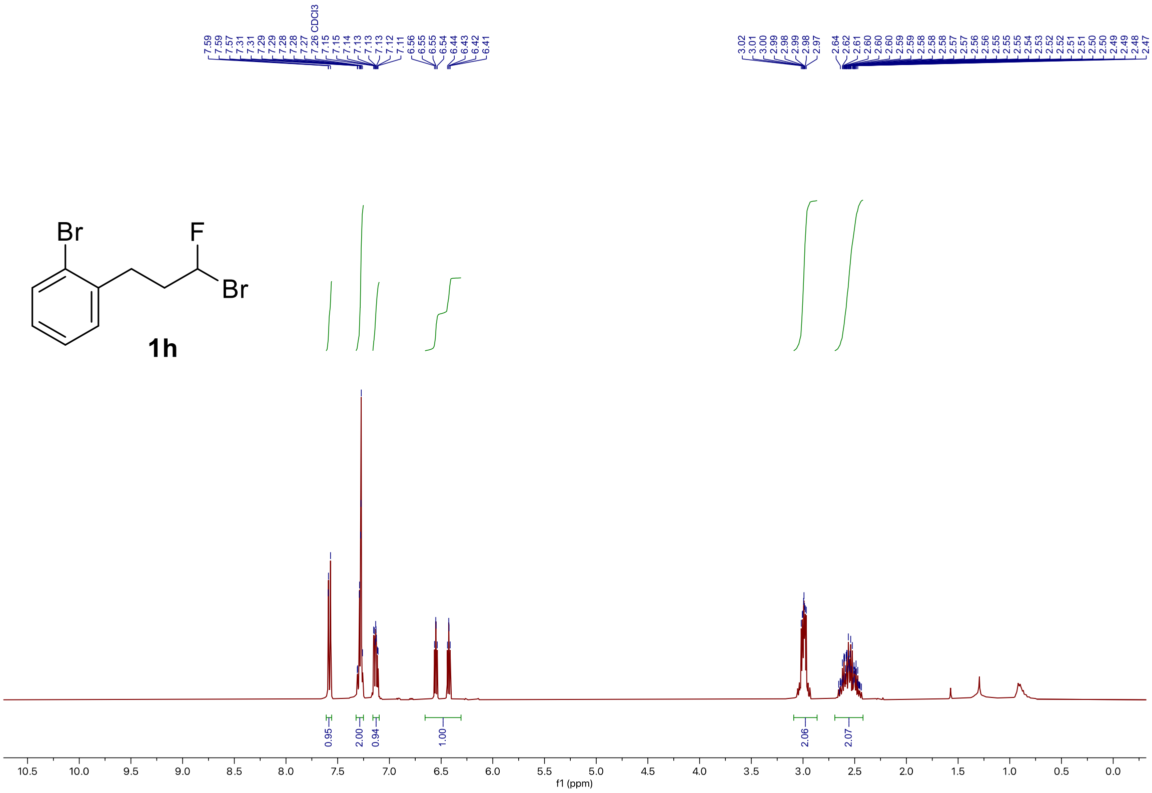

^{19}F NMR (376 MHz, CDCl$_3$)

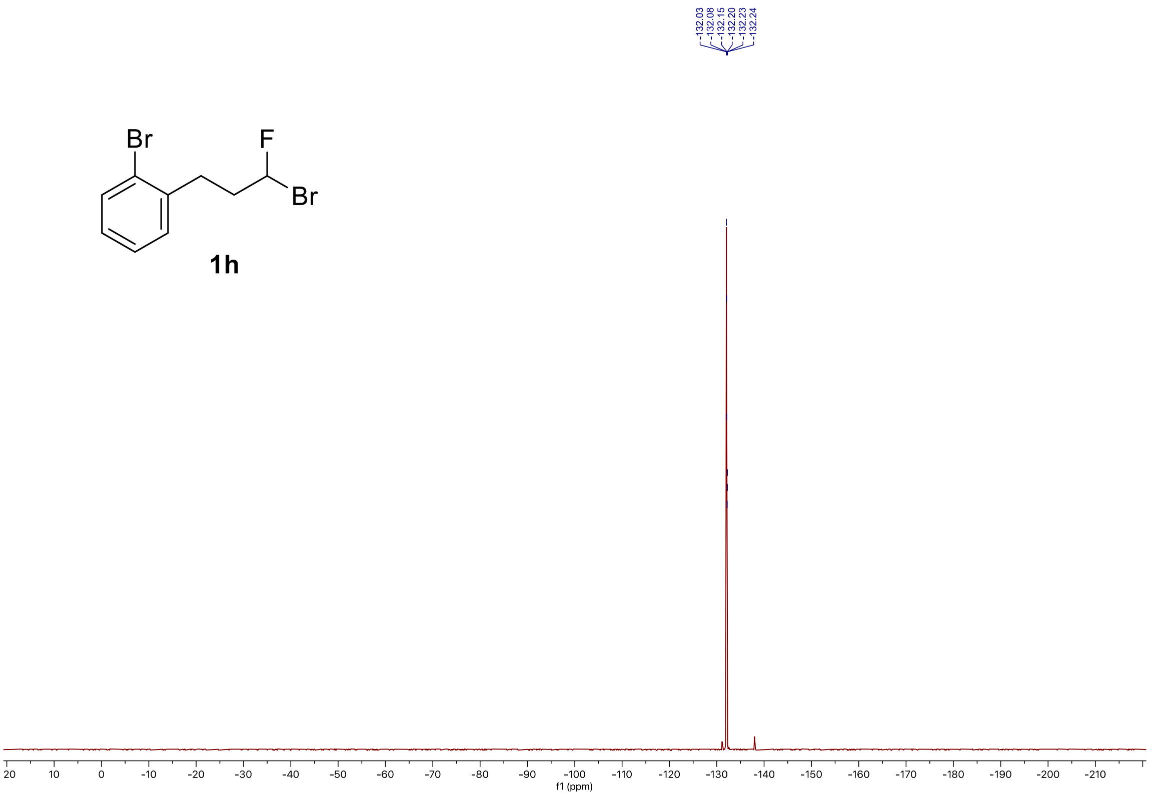

^{13}C NMR (101 MHz, $CDCl_3$)

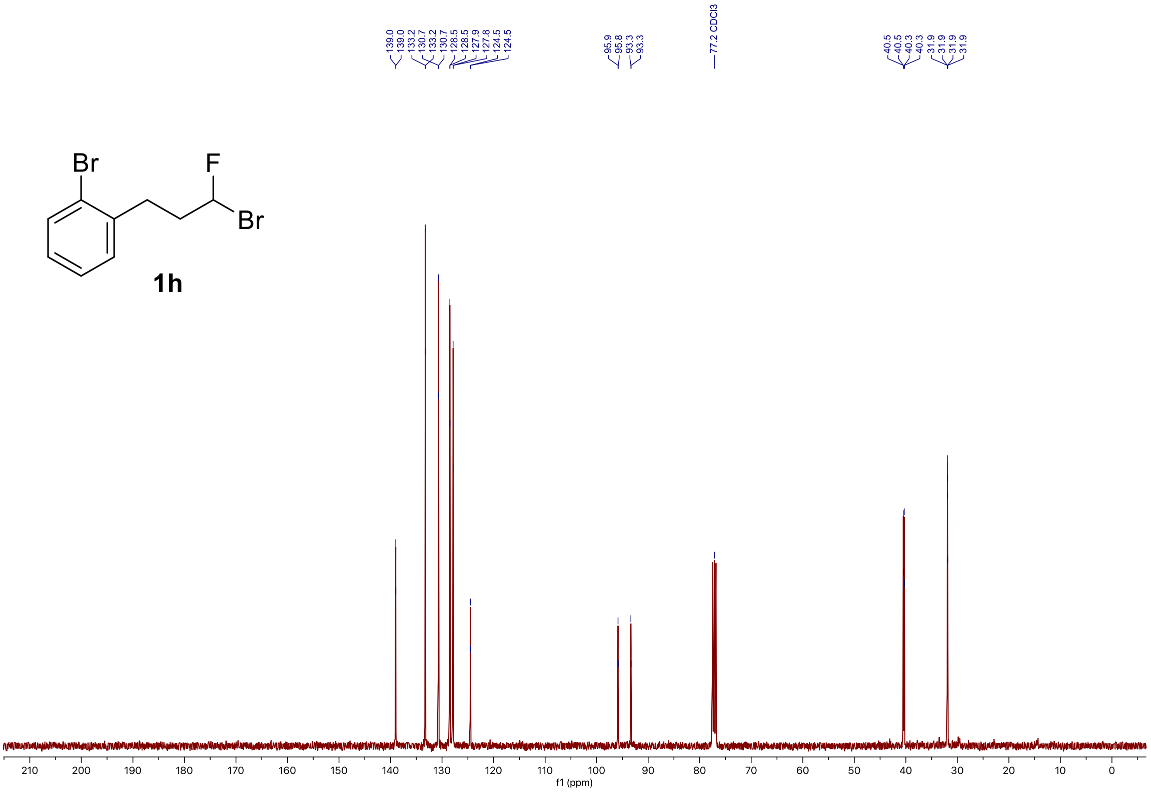

^{1}H NMR (400 MHz, $CDCl_3$)

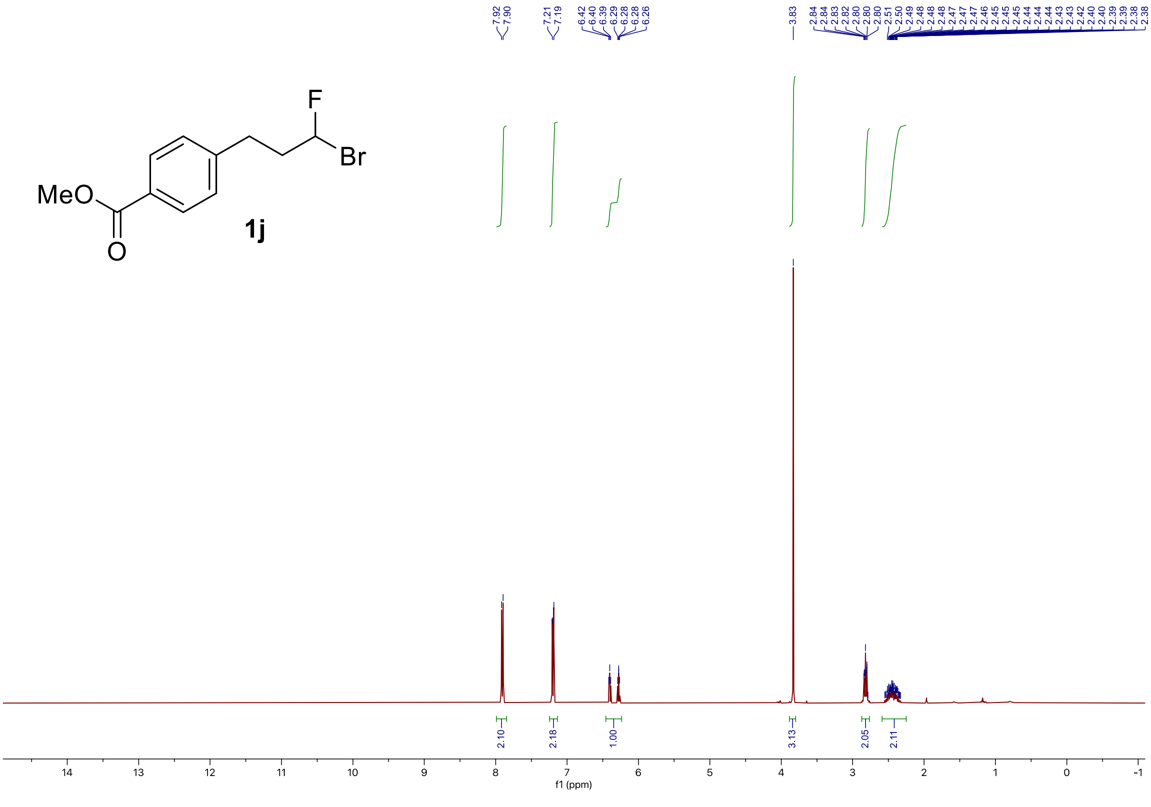

^{19}F NMR (376 MHz, $CDCl_3$)

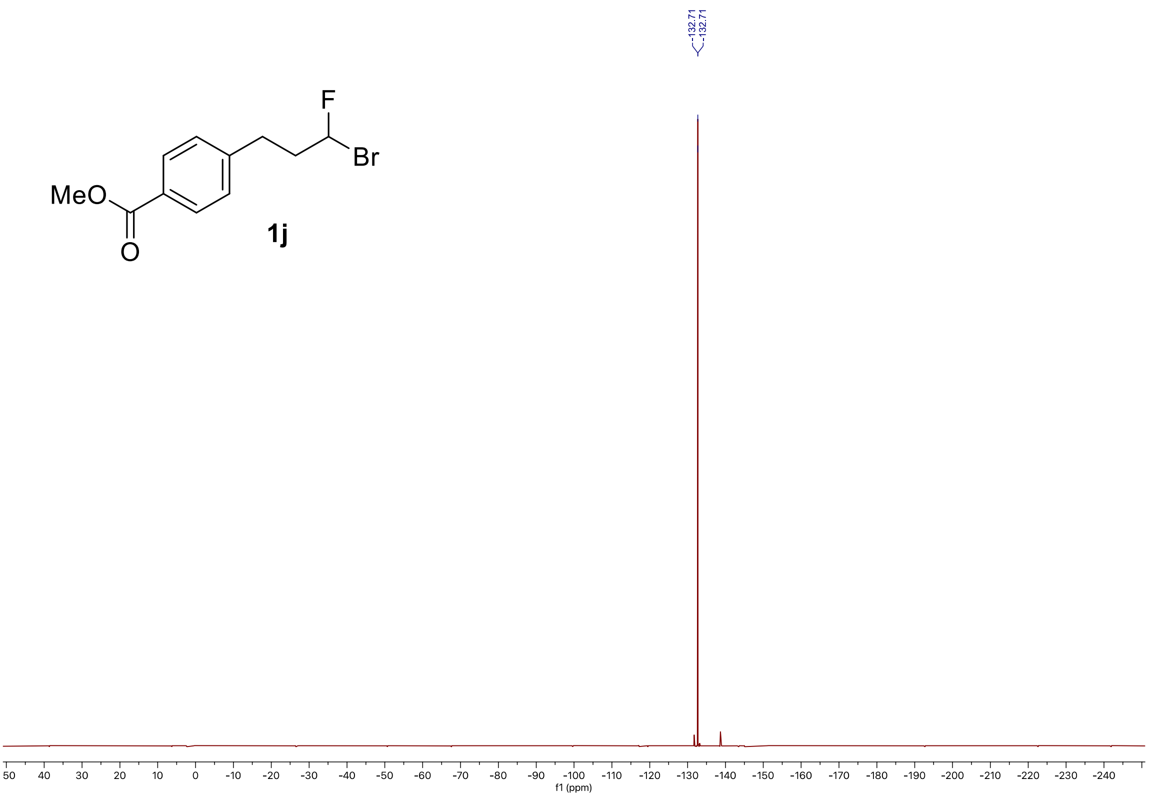

^{13}C NMR (101 MHz, $CDCl_3$)

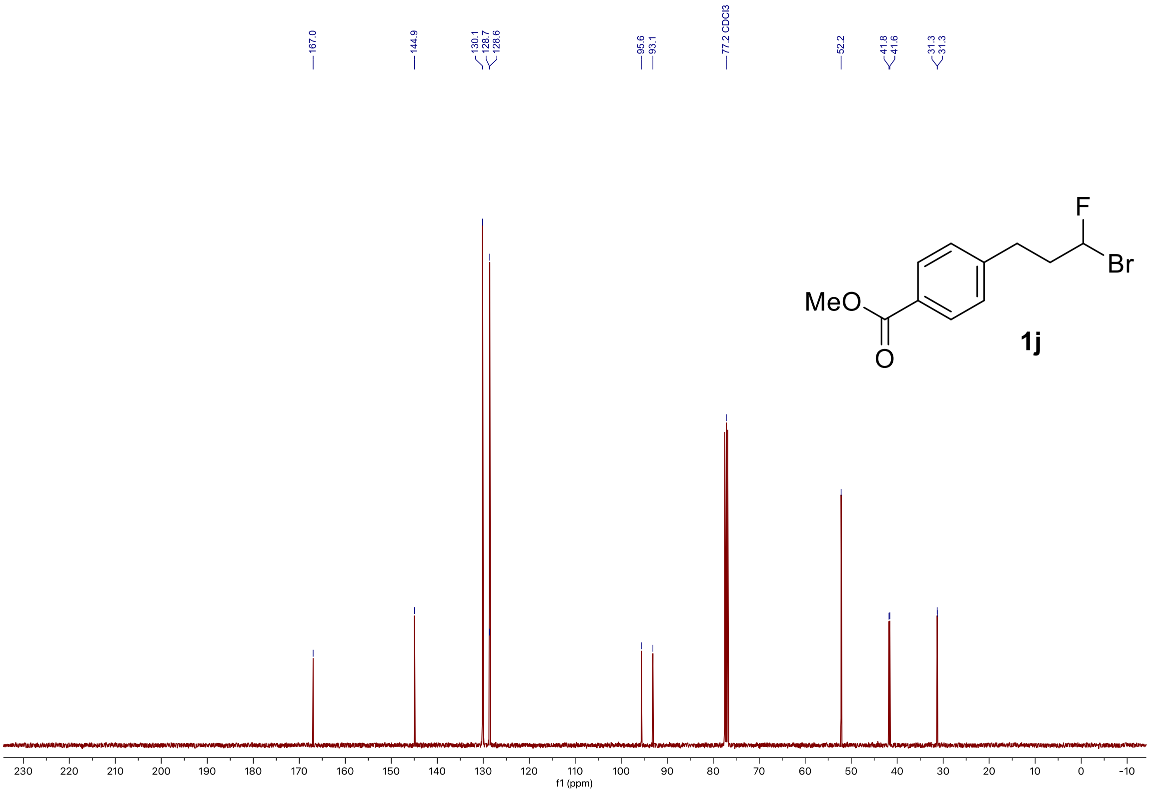

^{1}H NMR (400 MHz, $CDCl_3$)

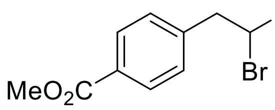

1n

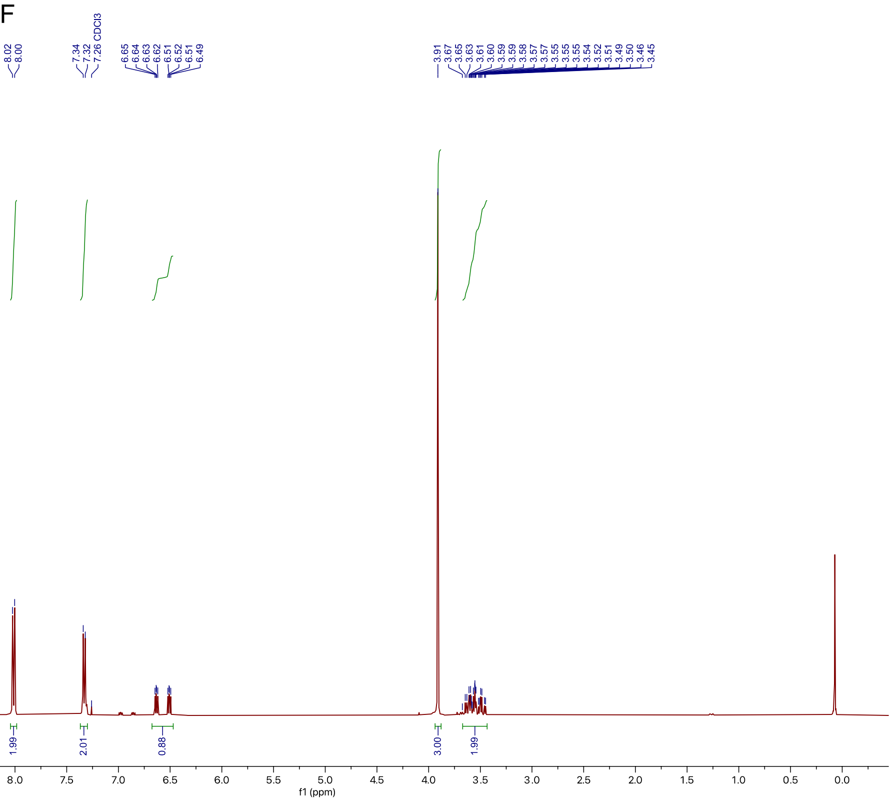

^{19}F NMR (376 MHz, $CDCl_3$)

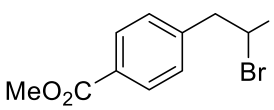

1n

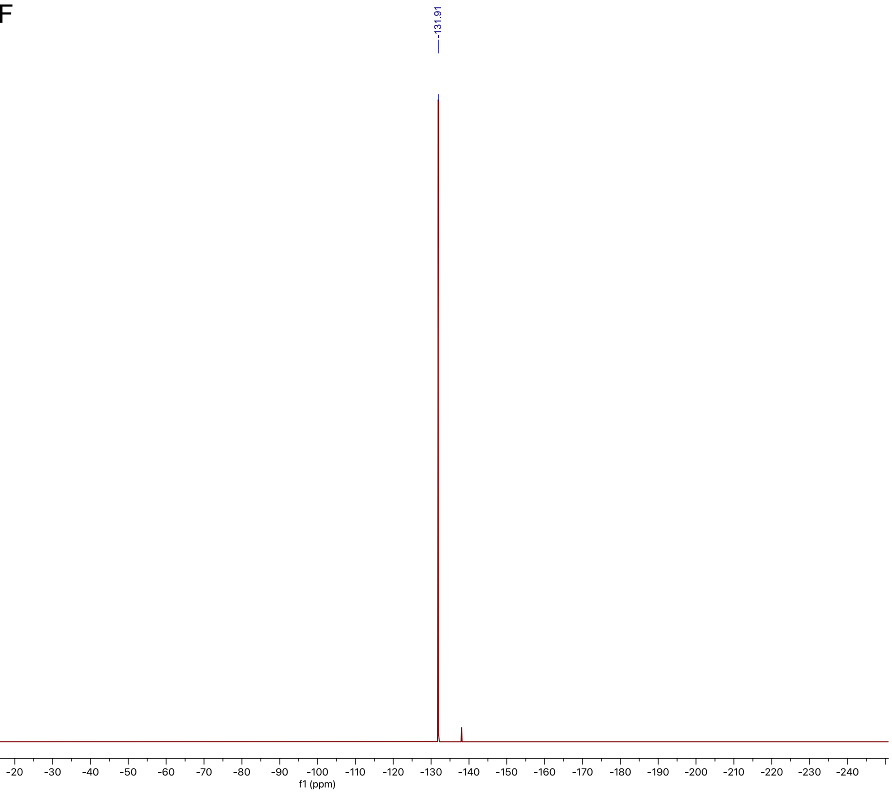

^{13}C NMR (101 MHz, $CDCl_3$)

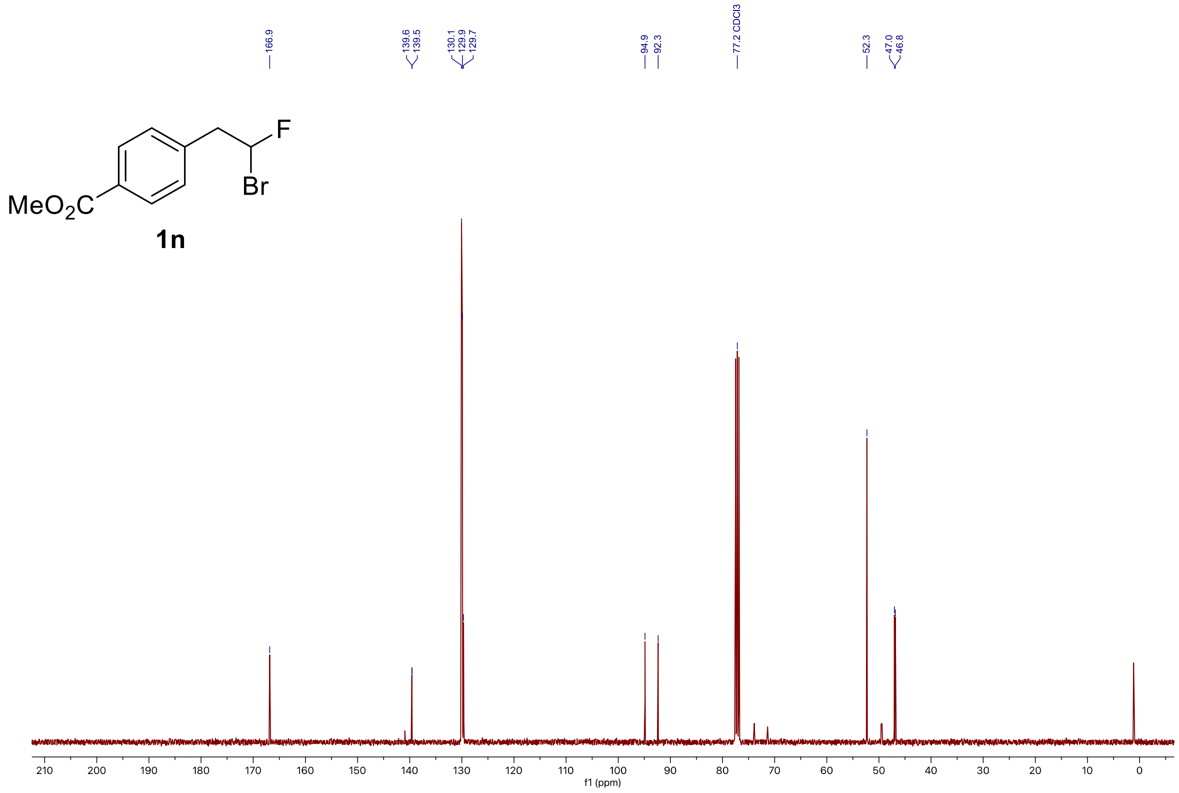

^{1}H NMR (400 MHz, $CDCl_3$)

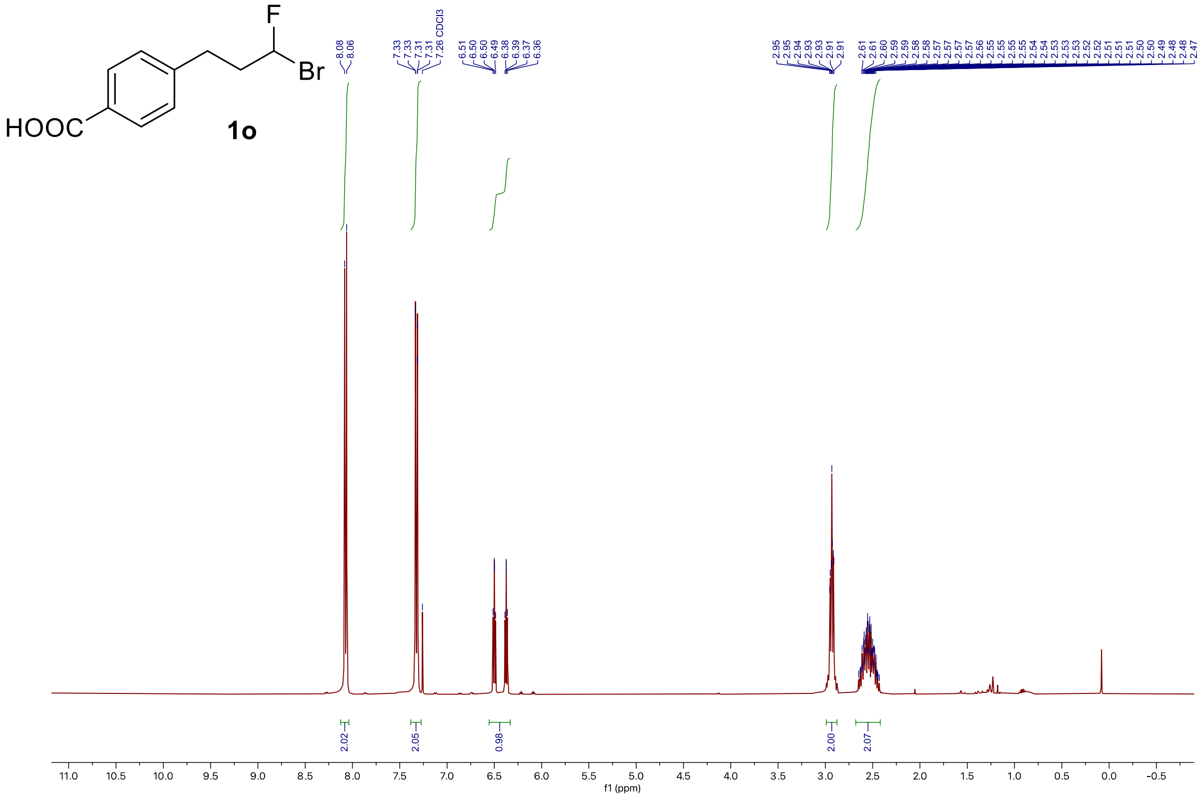

^{19}F NMR (376 MHz, $CDCl_3$)

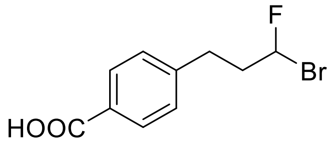

1o

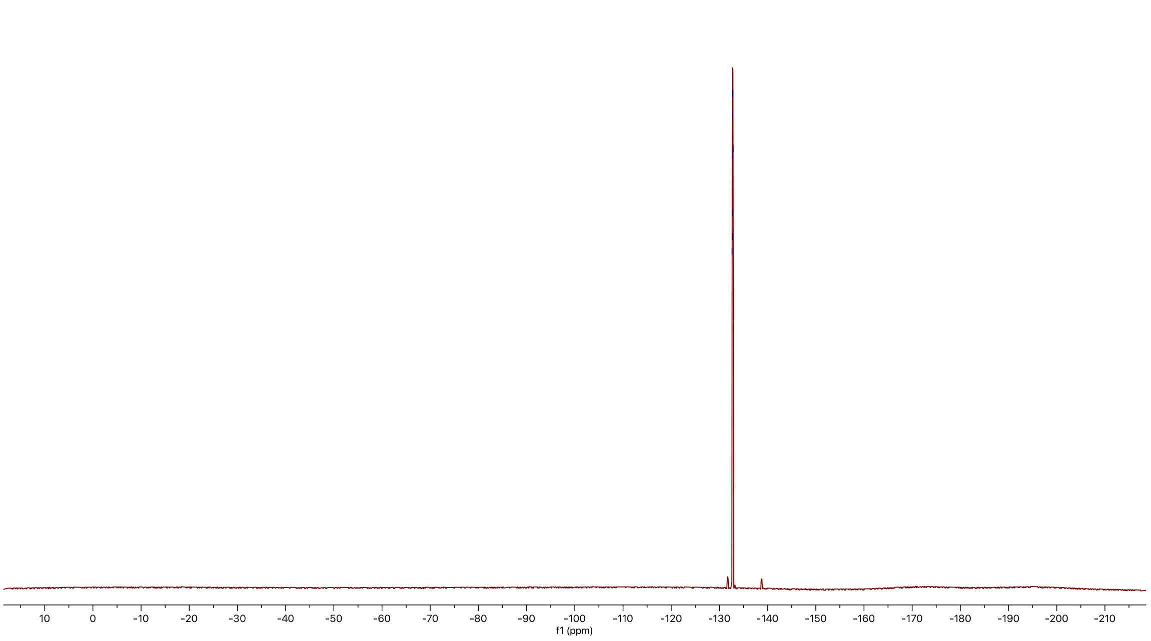

^{13}C NMR (101 MHz, $CDCl_3$)

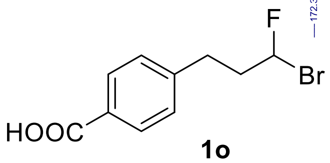

1o

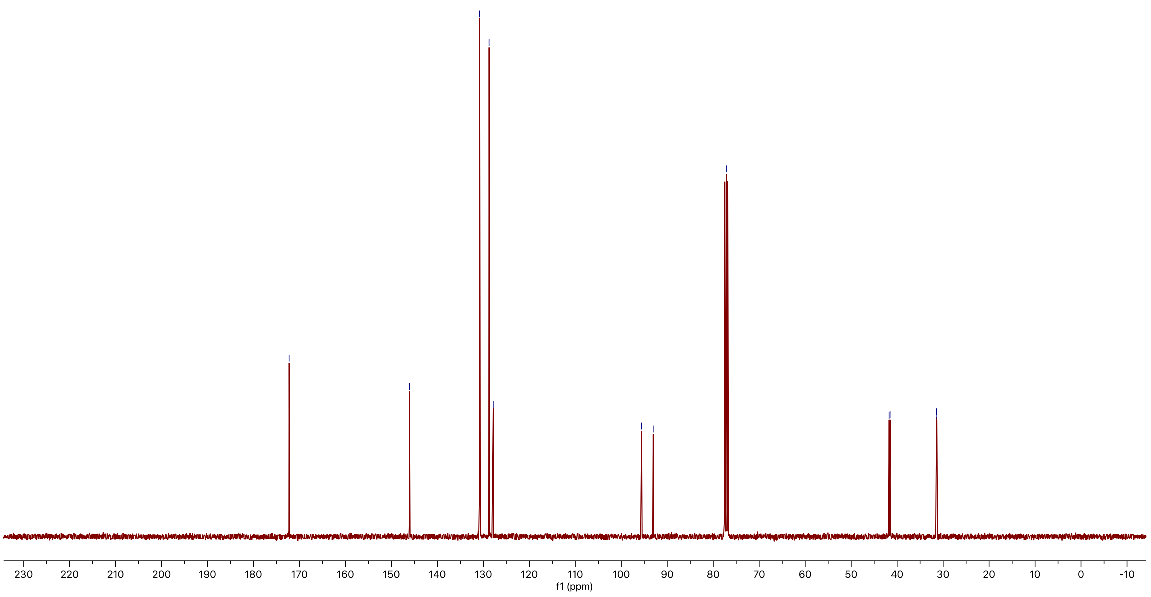

^{1}H NMR (400 MHz, $CDCl_3$)

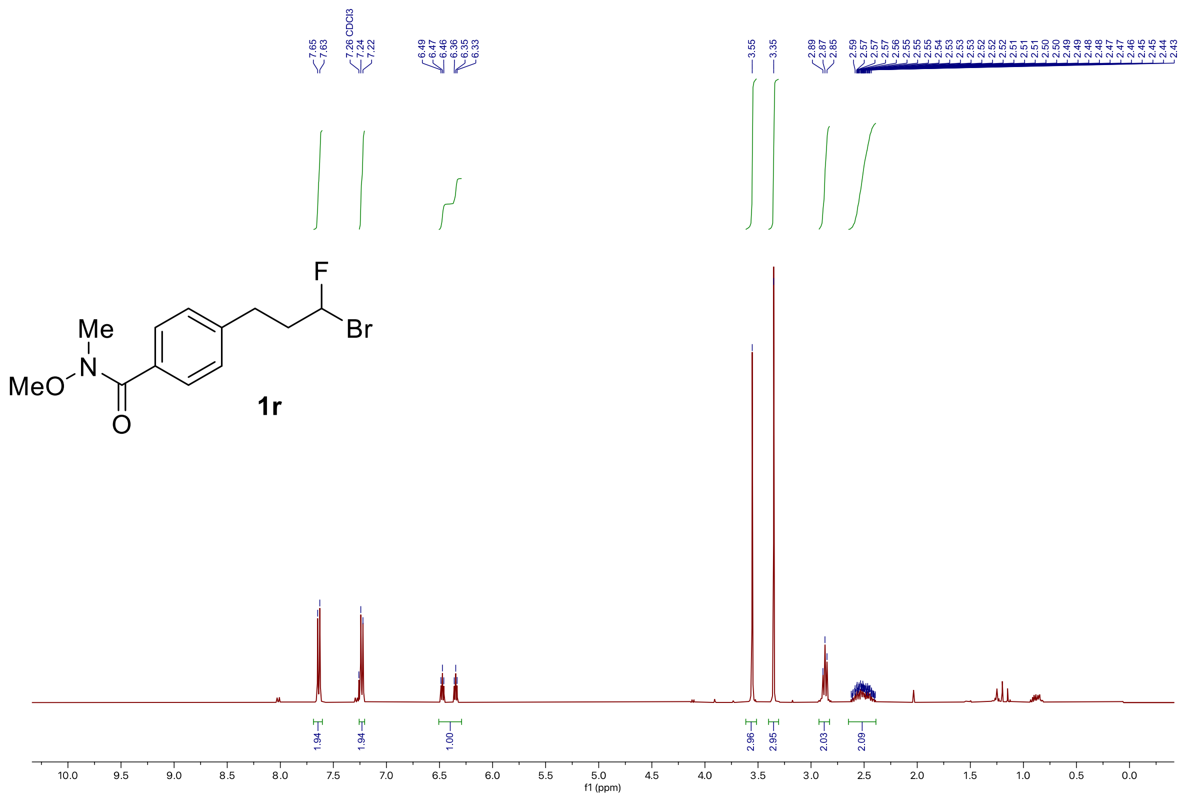

^{19}F NMR (376 MHz, $CDCl_3$)

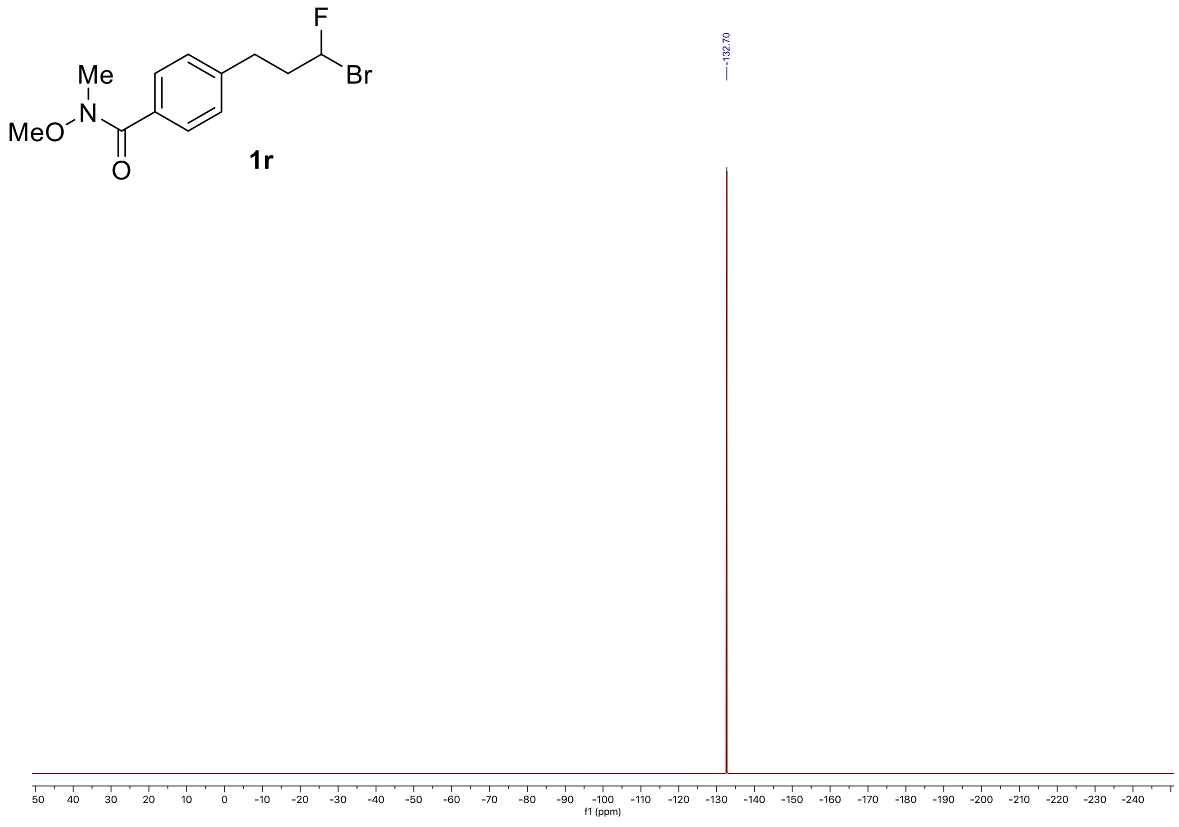

^{13}C NMR (101 MHz, $CDCl_3$)

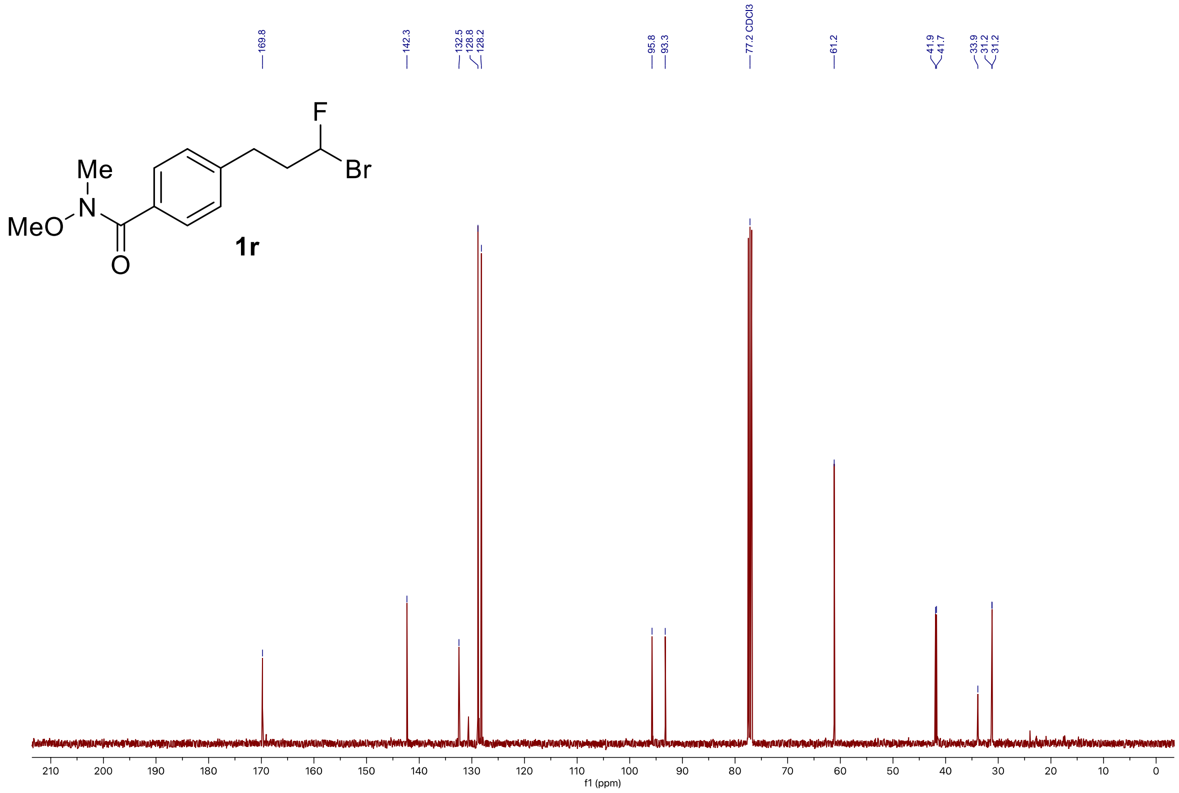

^{1}H NMR (400 MHz, $CDCl_3$)

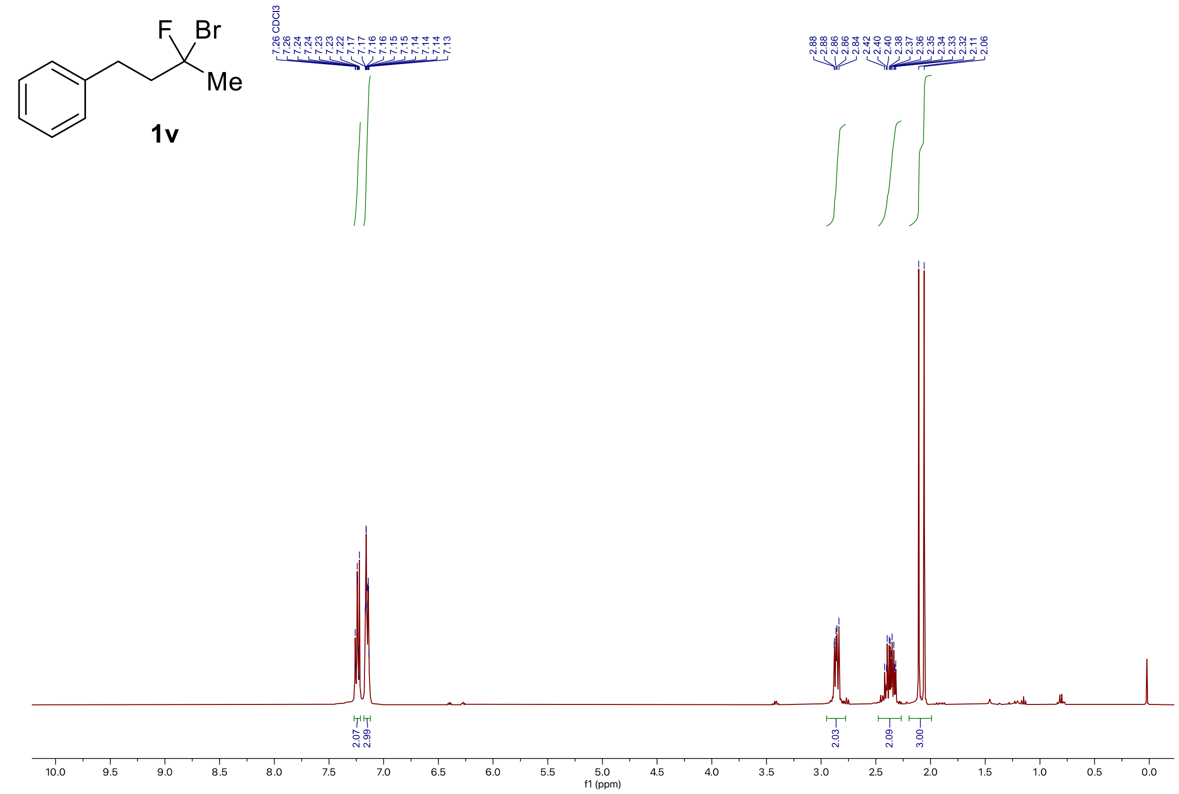

^{19}F NMR (376 MHz, $CDCl_3$)

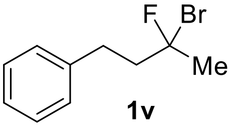

1v

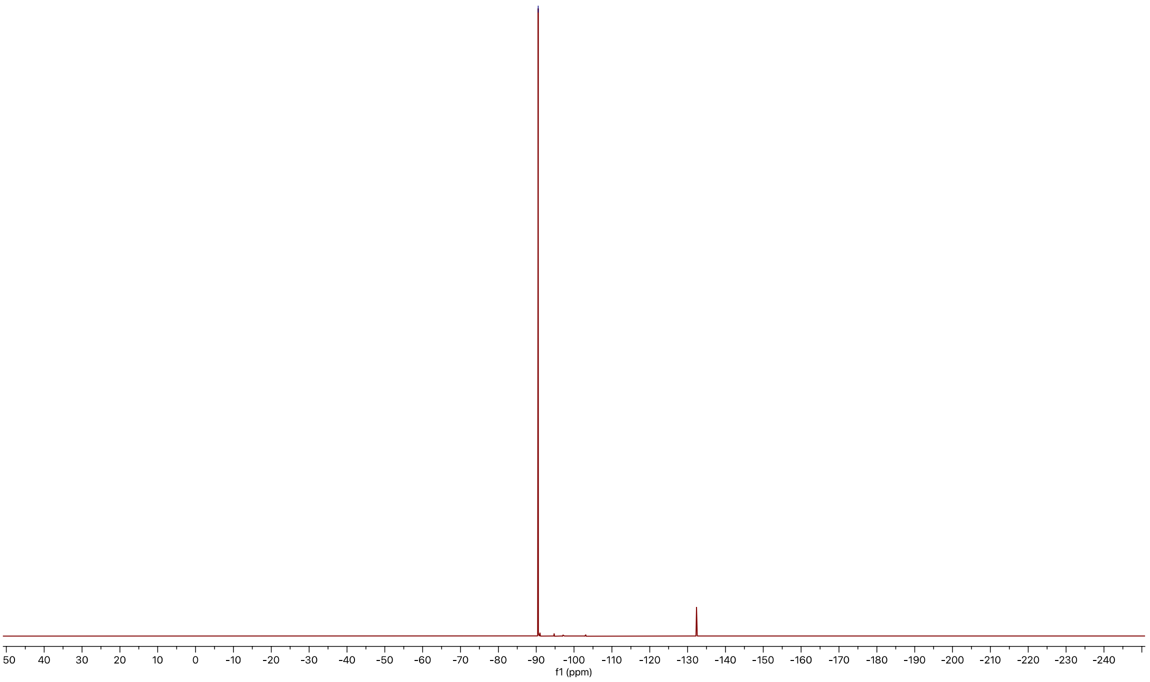

^{13}C NMR (101 MHz, $CDCl_3$)

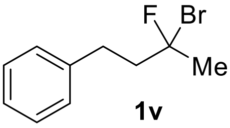

1v

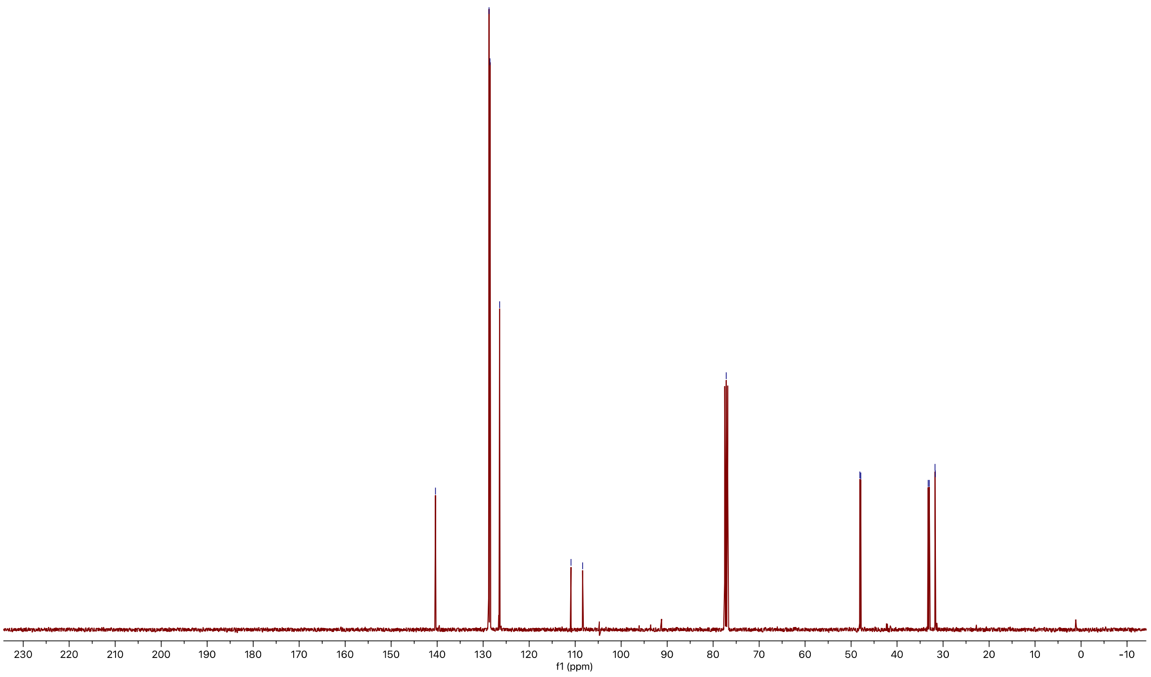

^{1}H NMR (400 MHz, $CDCl_3$)

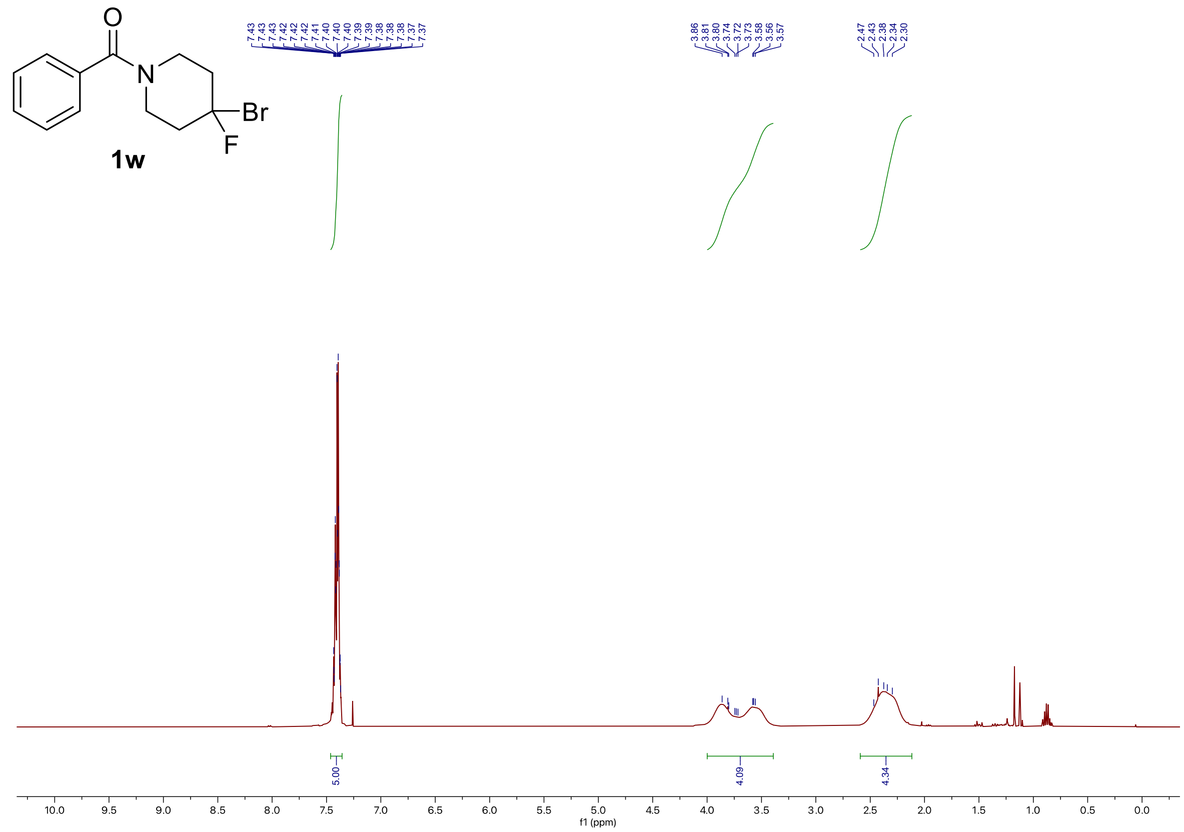

^{19}F NMR (376 MHz, $CDCl_3$)

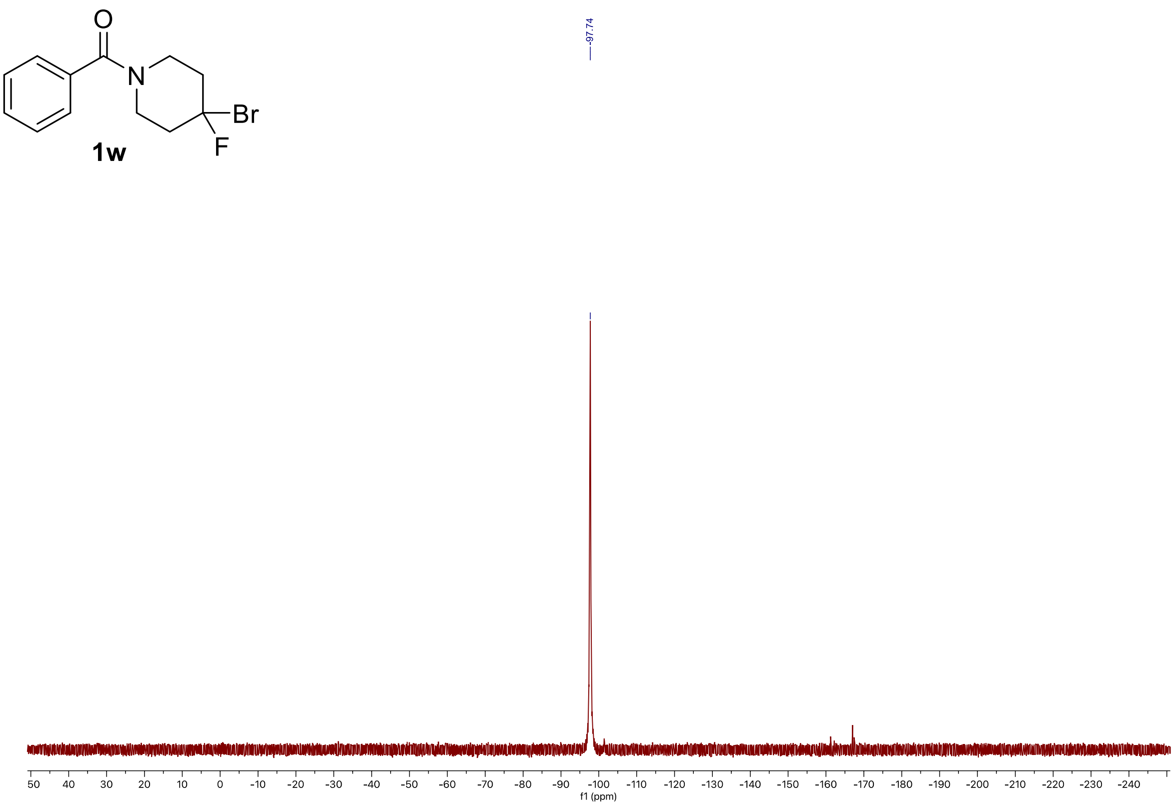

^{13}C NMR (101 MHz, $CDCl_3$)

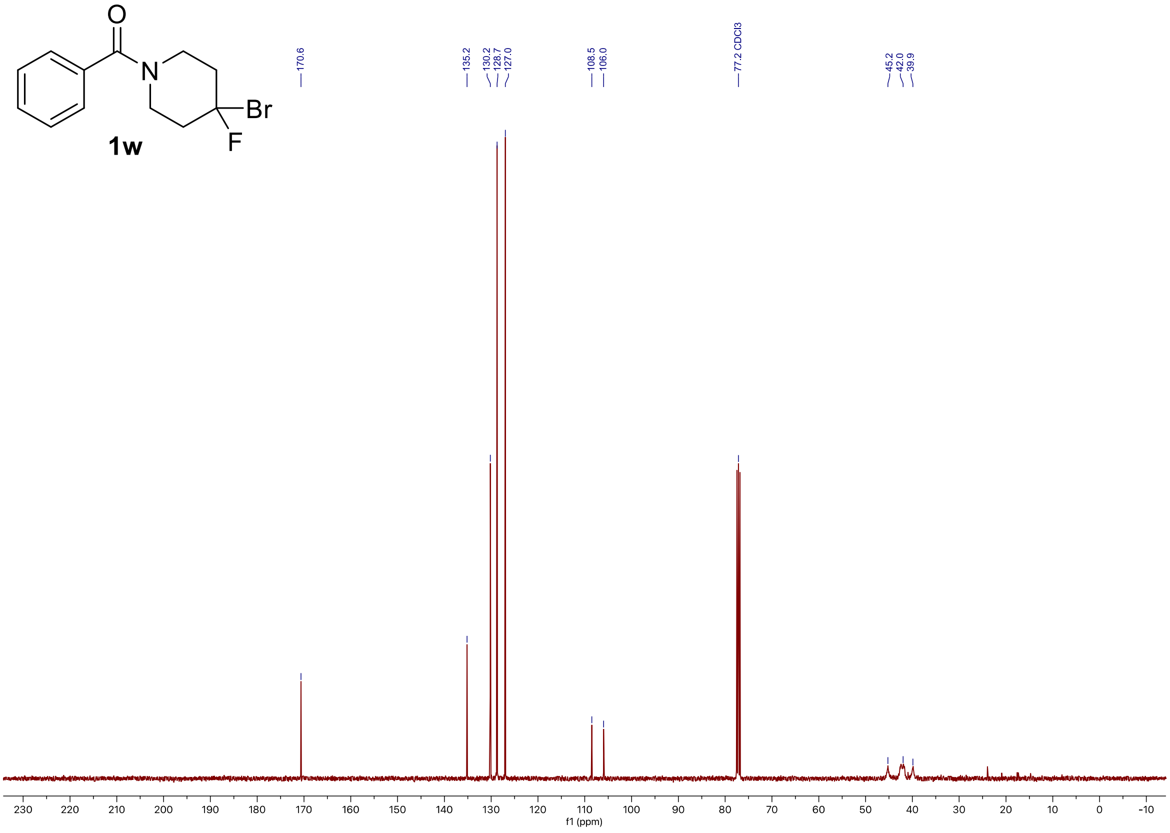

^{1}H NMR (400 MHz, $CDCl_3$)

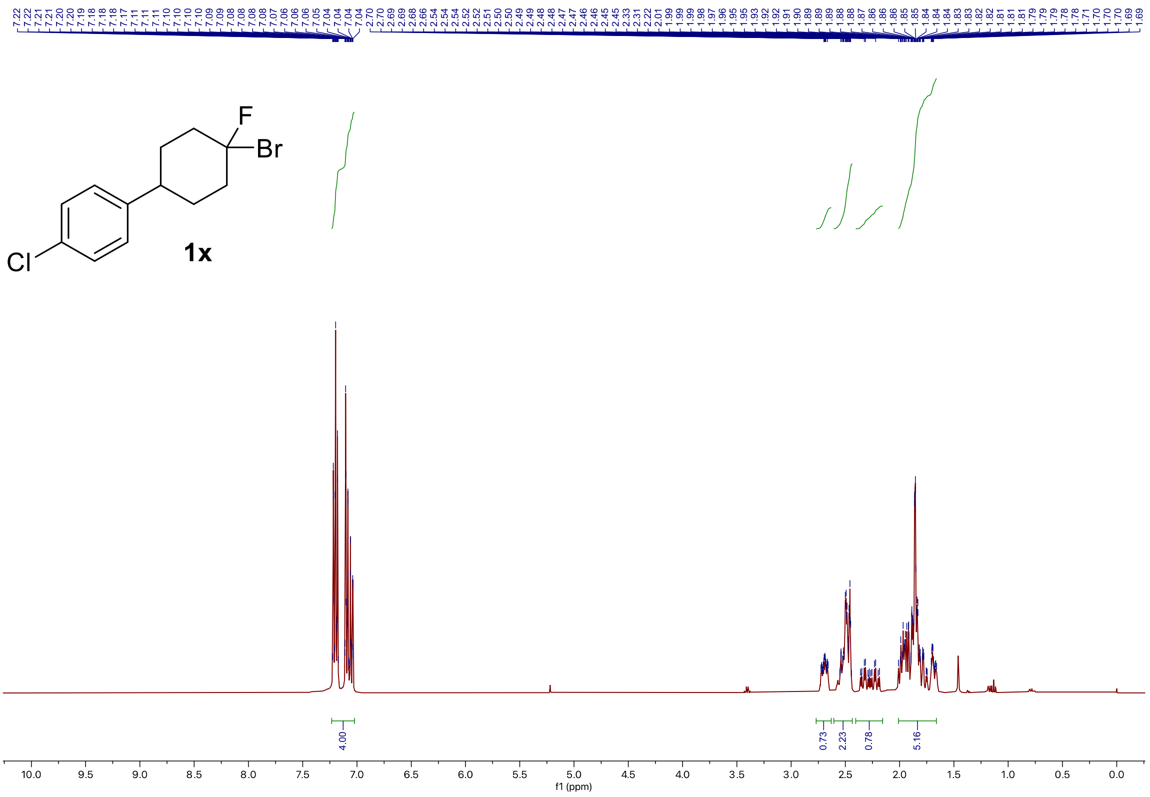

^{19}F NMR (376 MHz, $CDCl_3$)

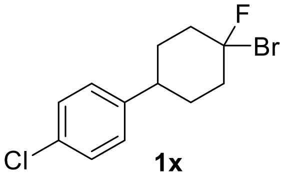

1x

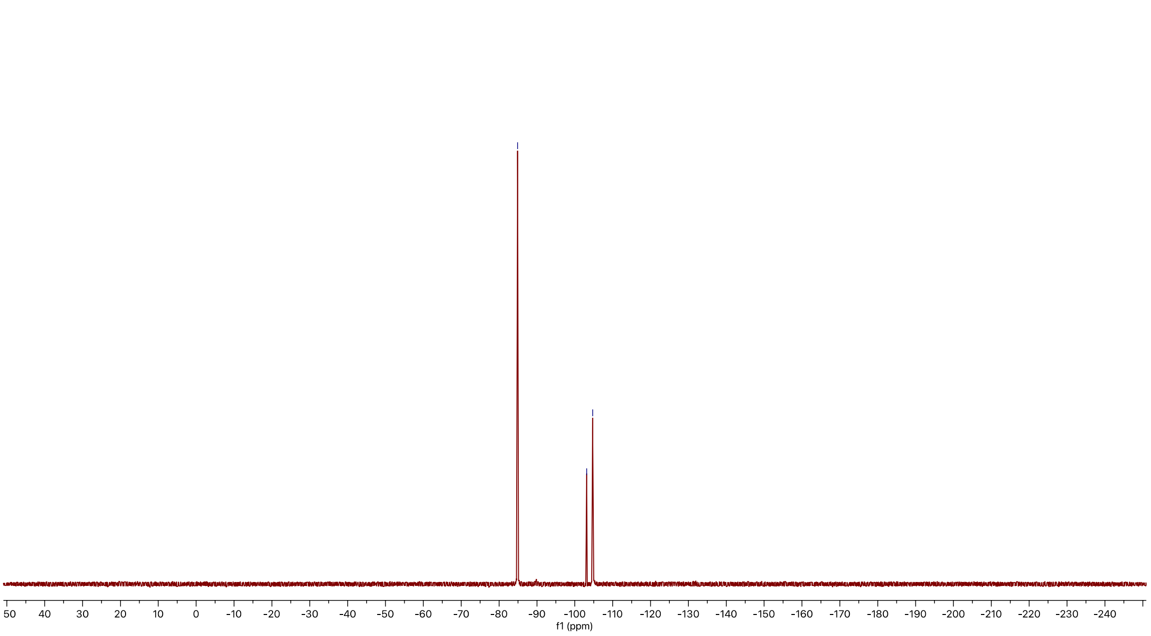

^{13}C NMR (101 MHz, $CDCl_3$)

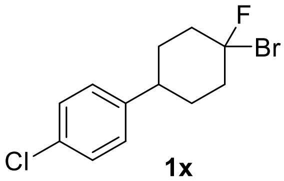

1x

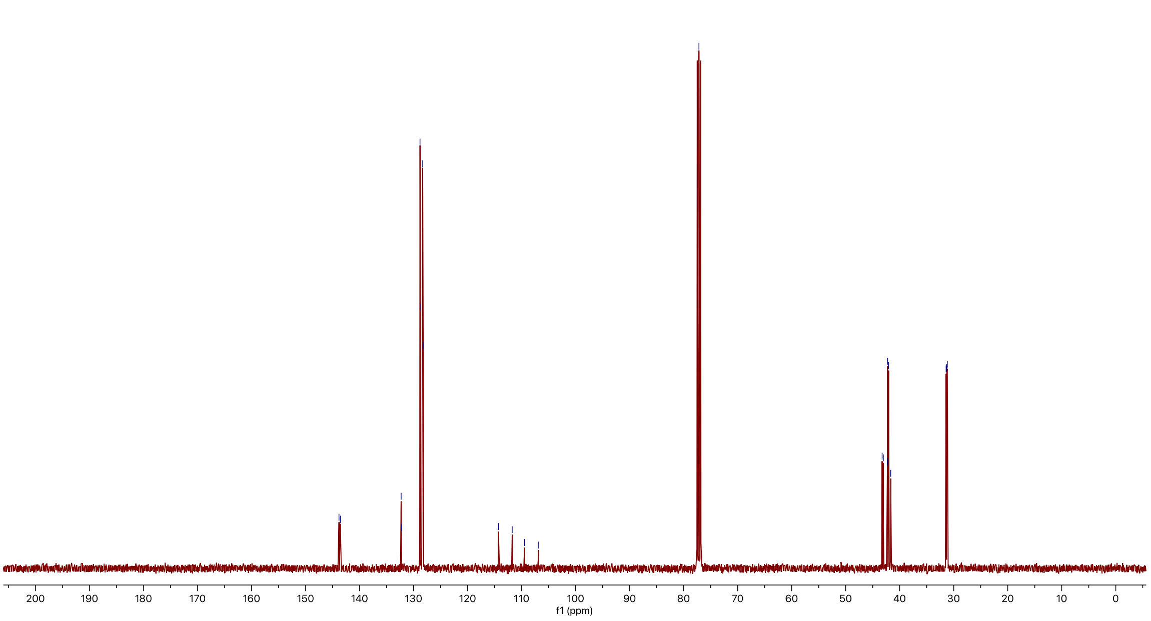

^{1}H NMR (400 MHz, CDCl$_3$)

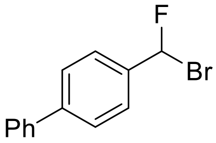

1y

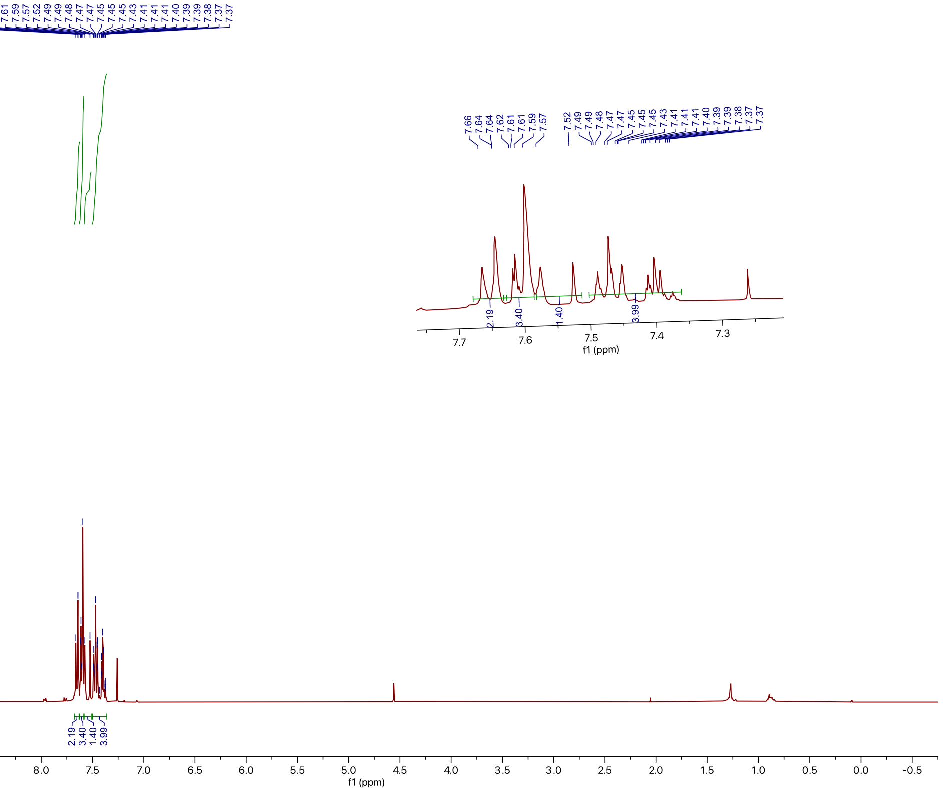

^{19}F NMR (376 MHz, CDCl$_3$)

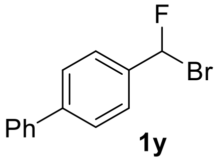

1y

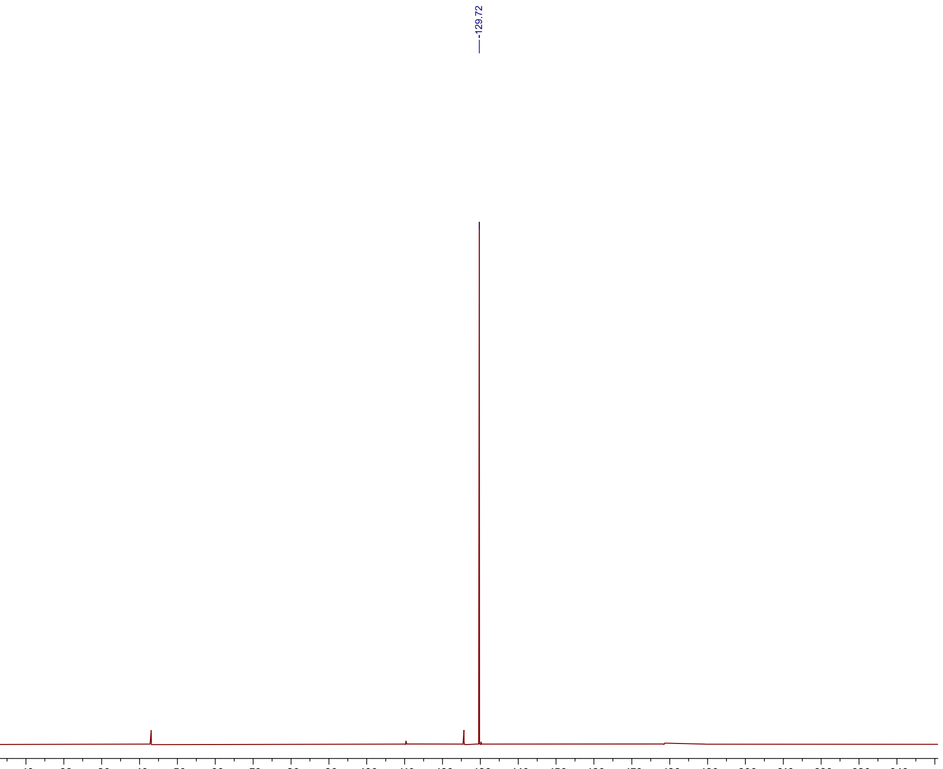

^{13}C NMR (101 MHz, $CDCl_3$)

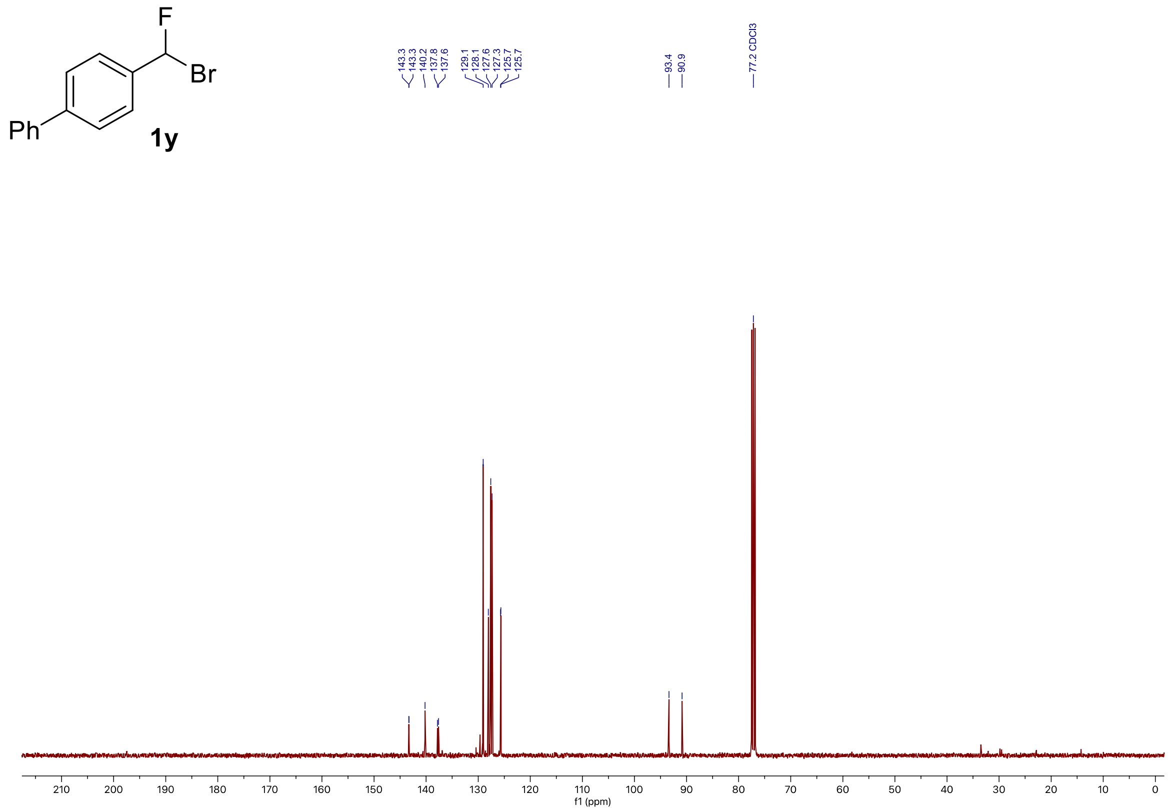

^{1}H NMR (400 MHz, $CDCl_3$)

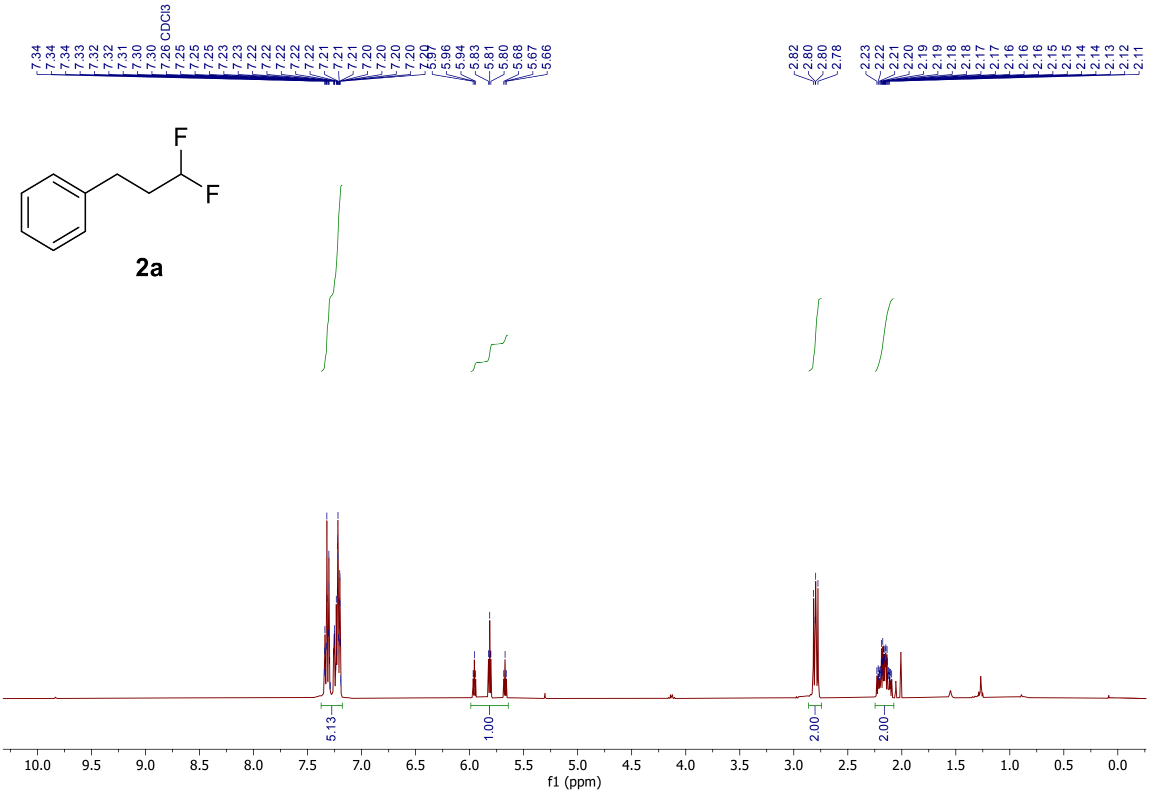

^{19}F NMR (376 MHz, $CDCl_3$)

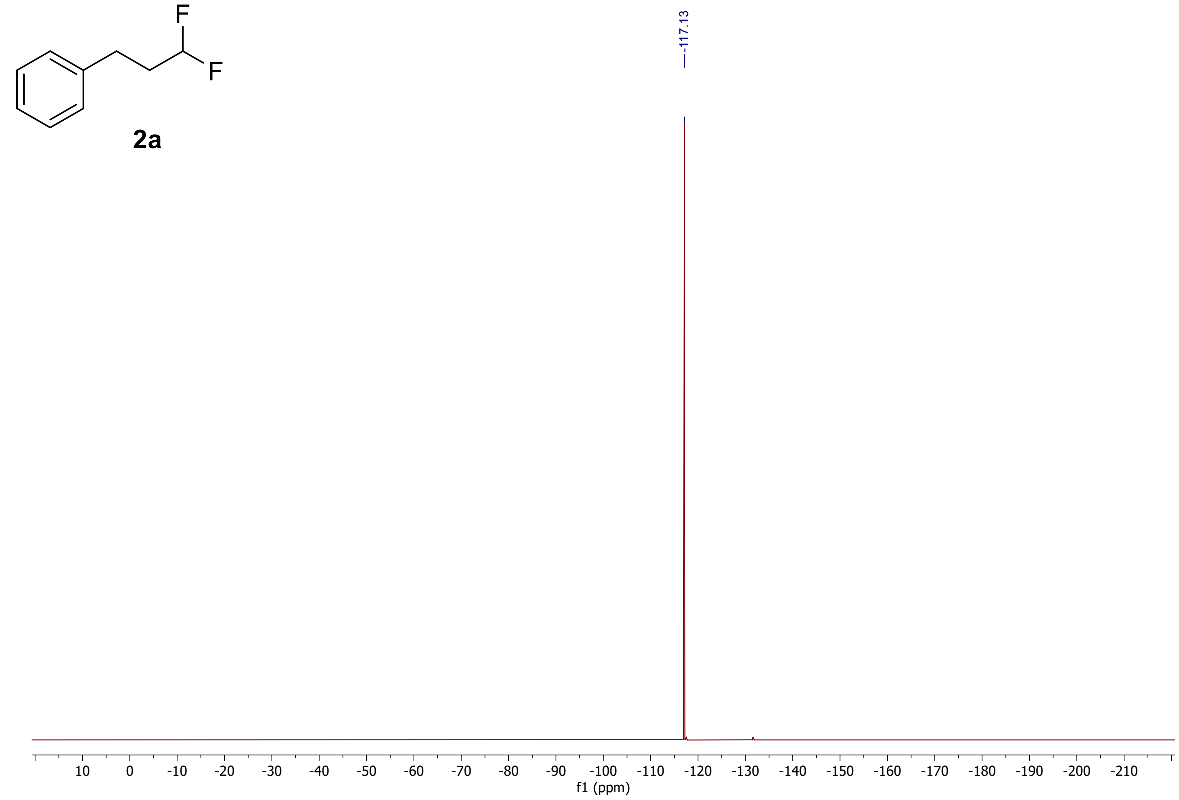

^{13}C NMR (101 MHz, $CDCl_3$)

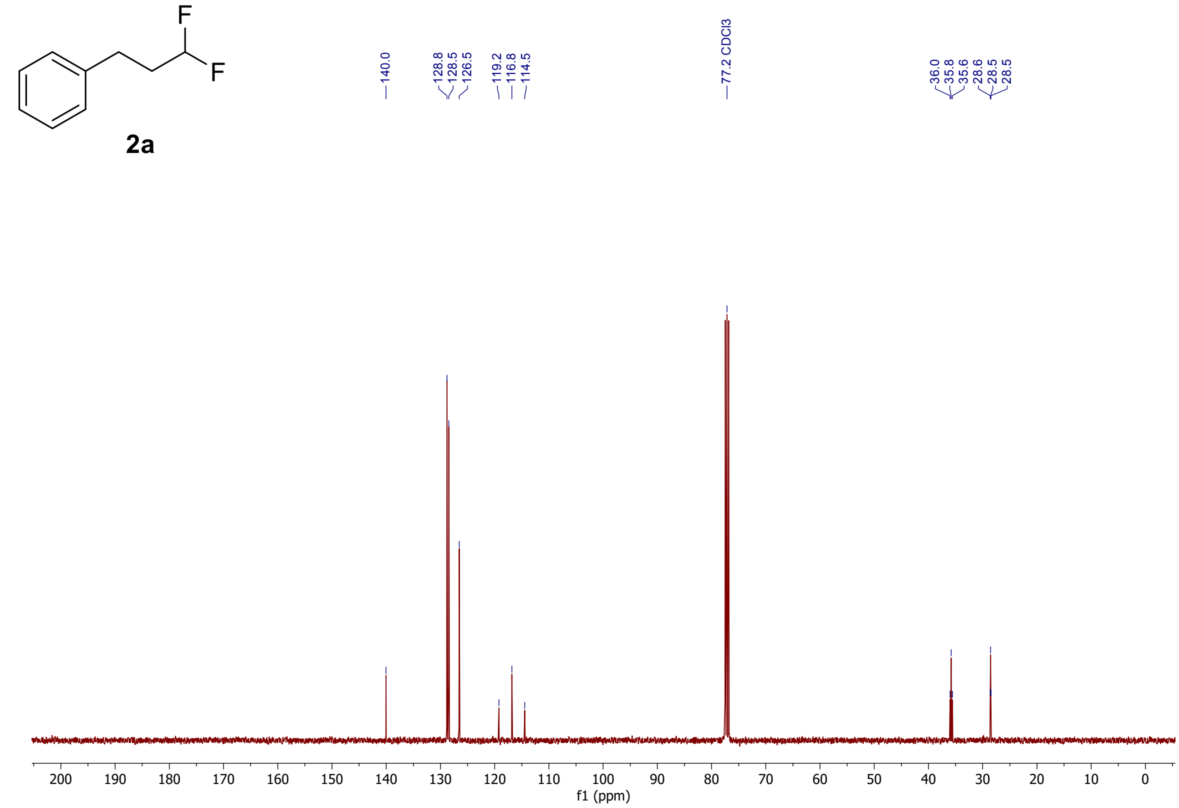

^{1}H NMR (400 MHz, CDCl$_3$)

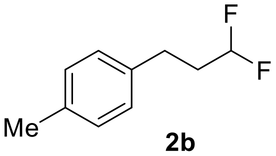

2b

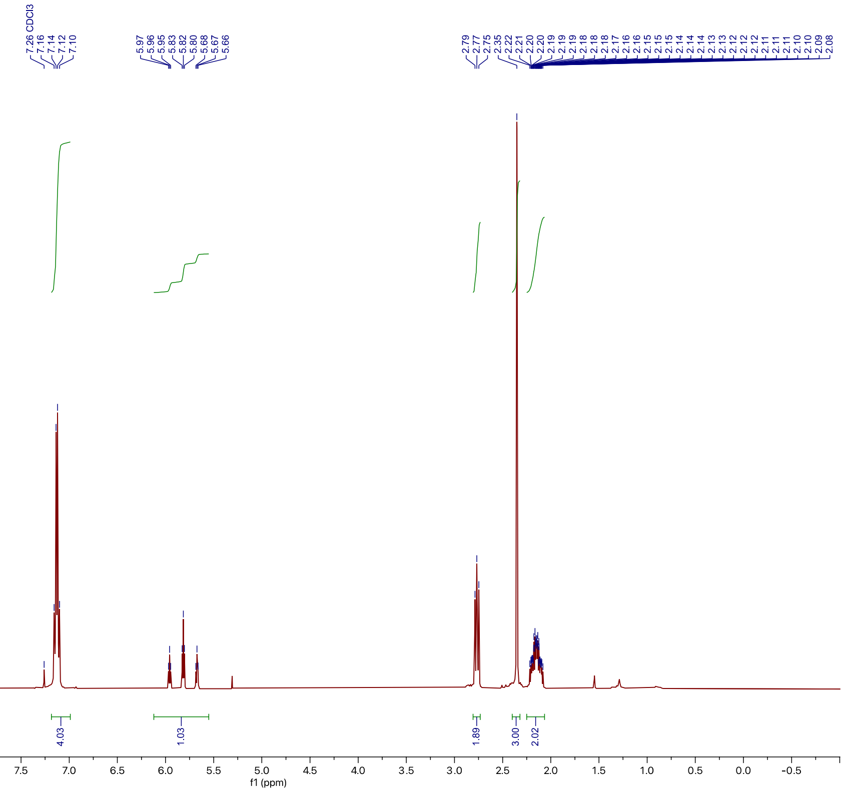

^{19}F NMR (376 MHz, CDCl$_3$)

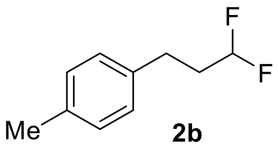

2b

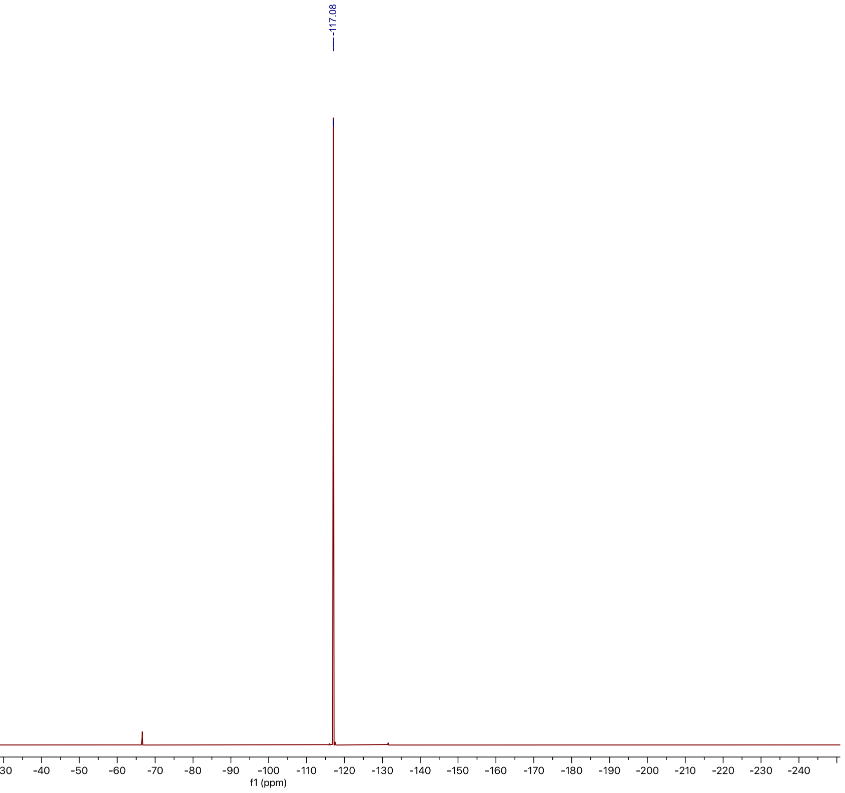

^{13}C NMR (101 MHz, CDCl$_3$)

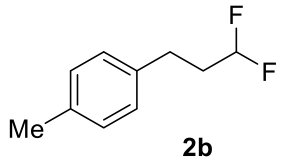

2b

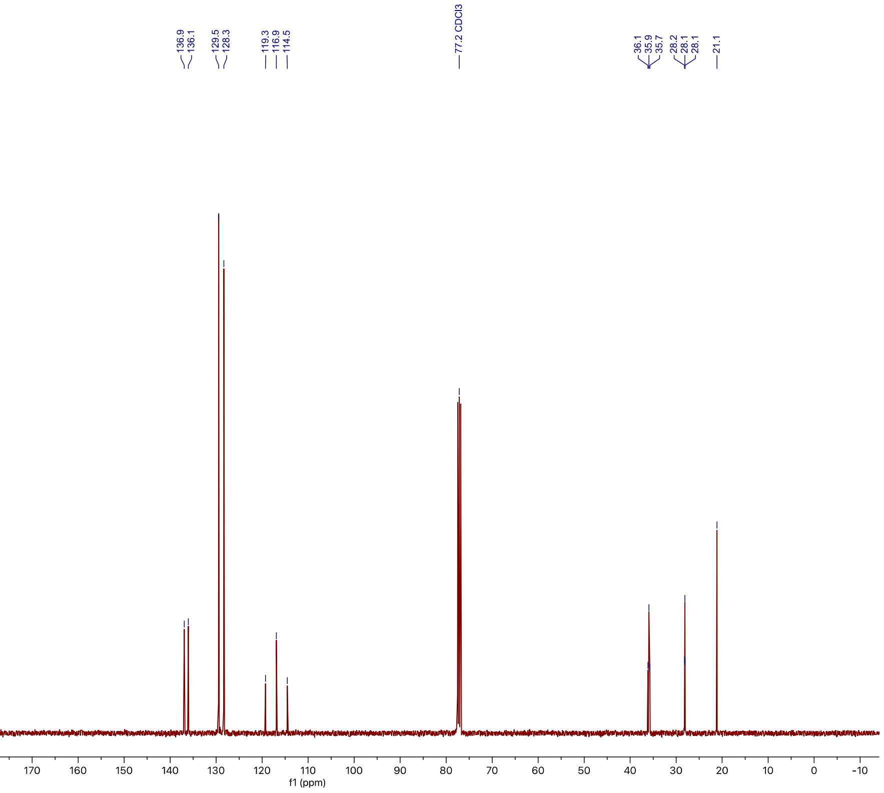

^{1}H NMR (500 MHz, CDCl$_3$)

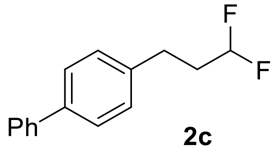

2c

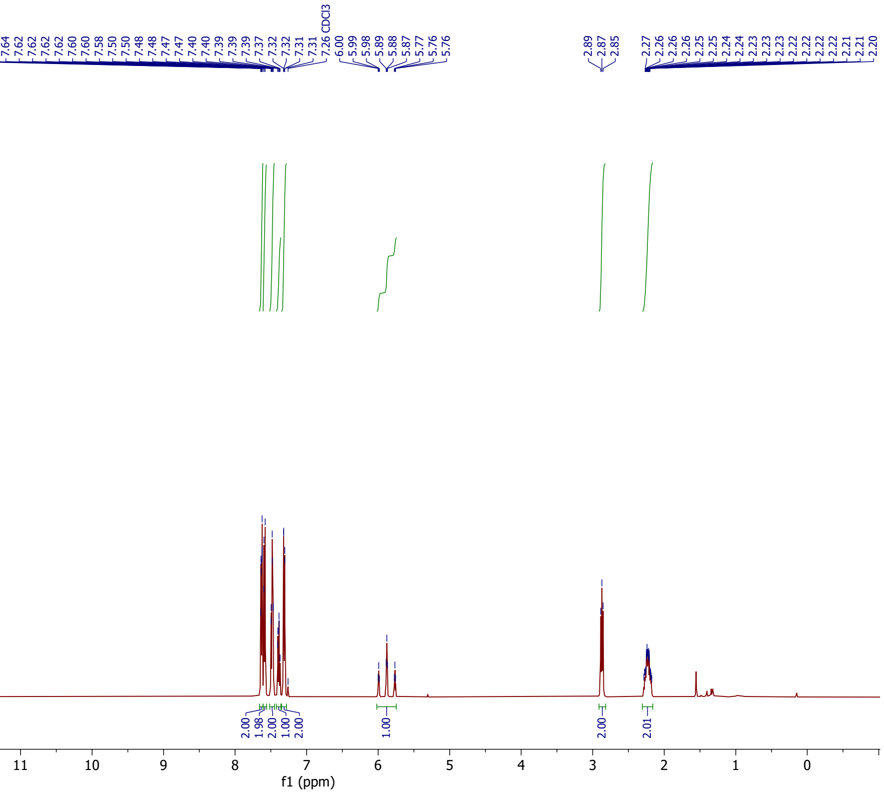

^{19}F NMR (471 MHz, $CDCl_3$)

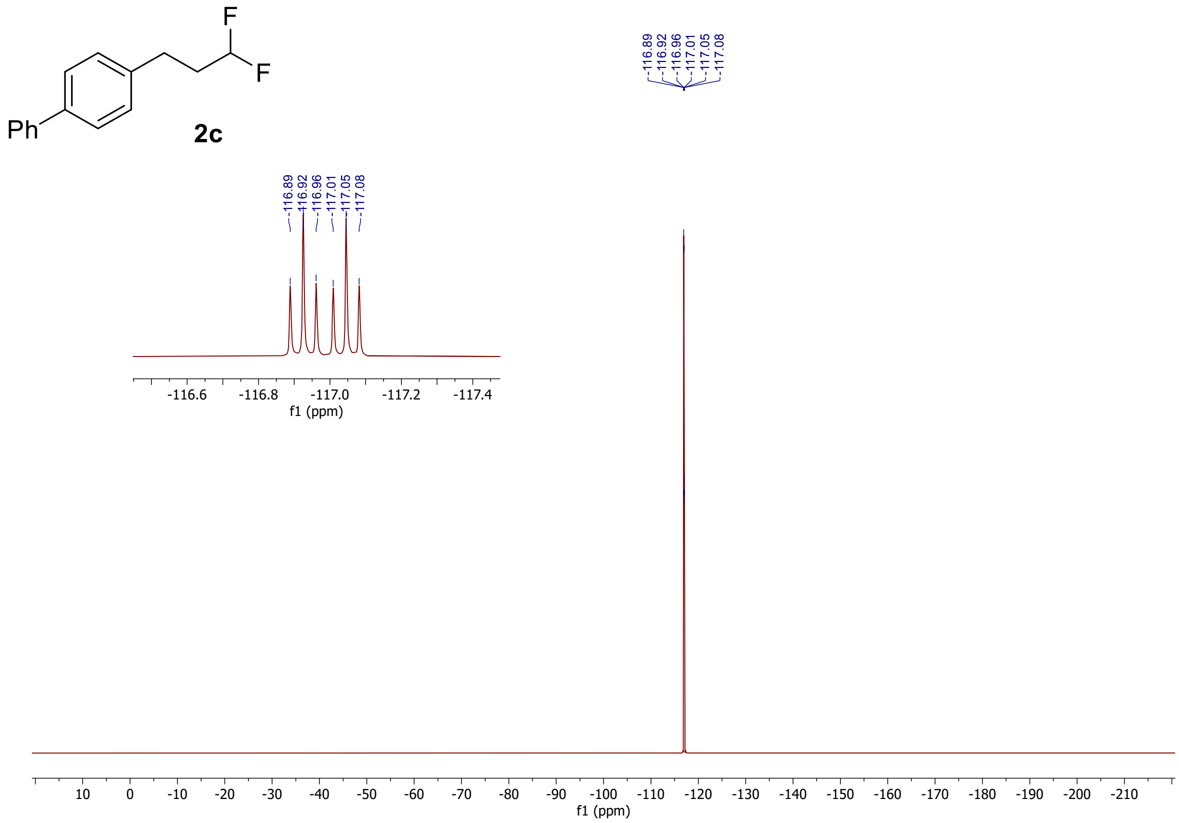

^{13}C NMR (126 MHz, $CDCl_3$)

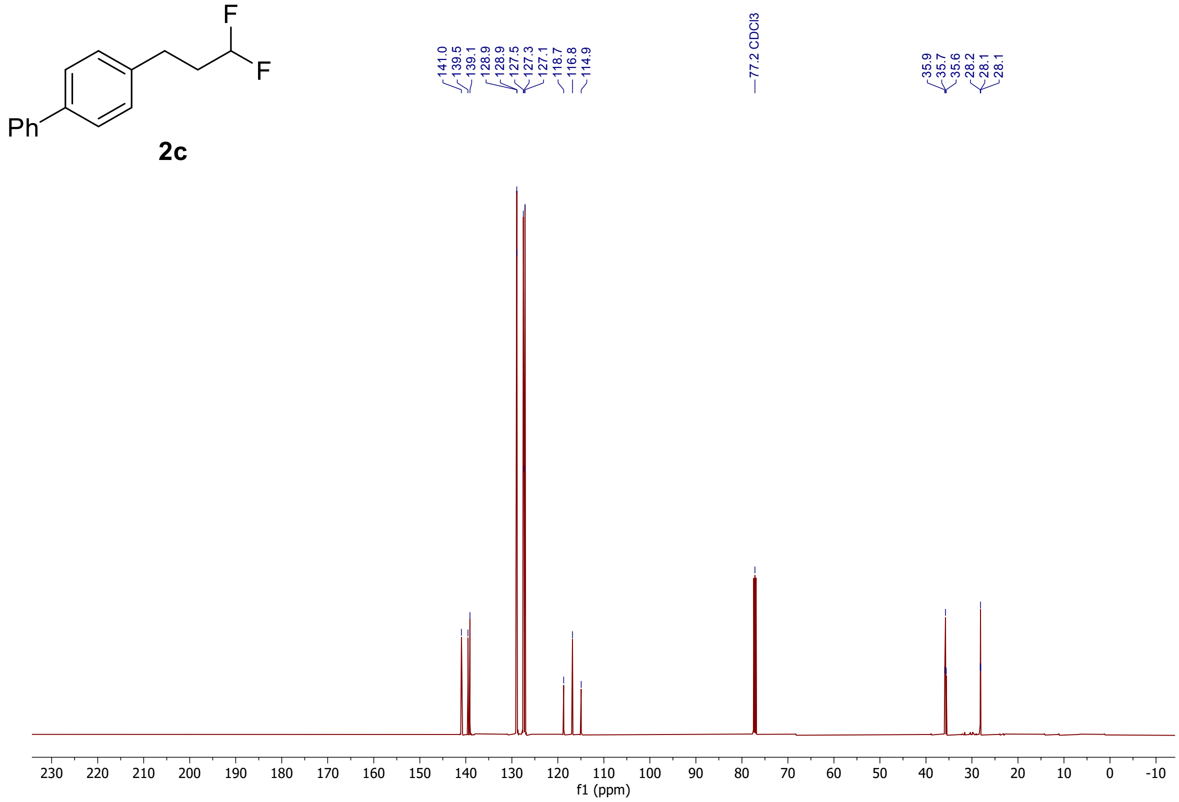

^{1}H NMR (400 MHz, $CDCl_3$)

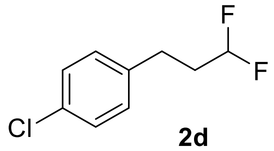

2d

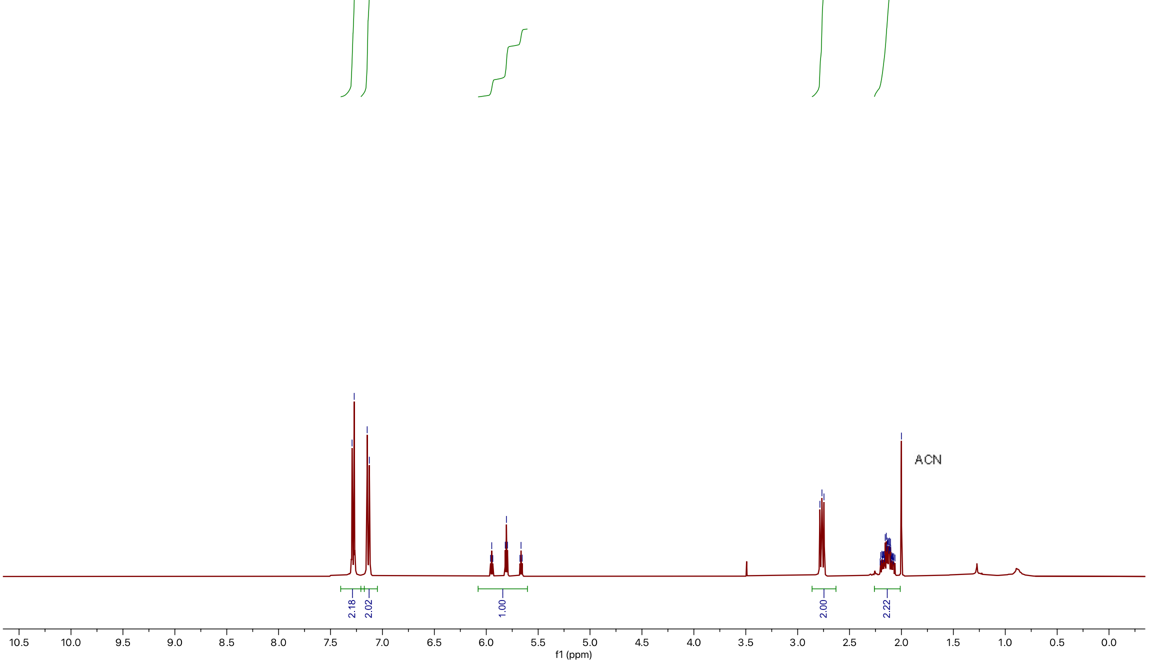

^{19}F NMR (376 MHz, $CDCl_3$)

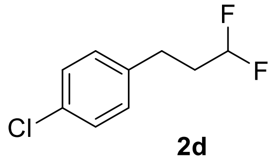

2d

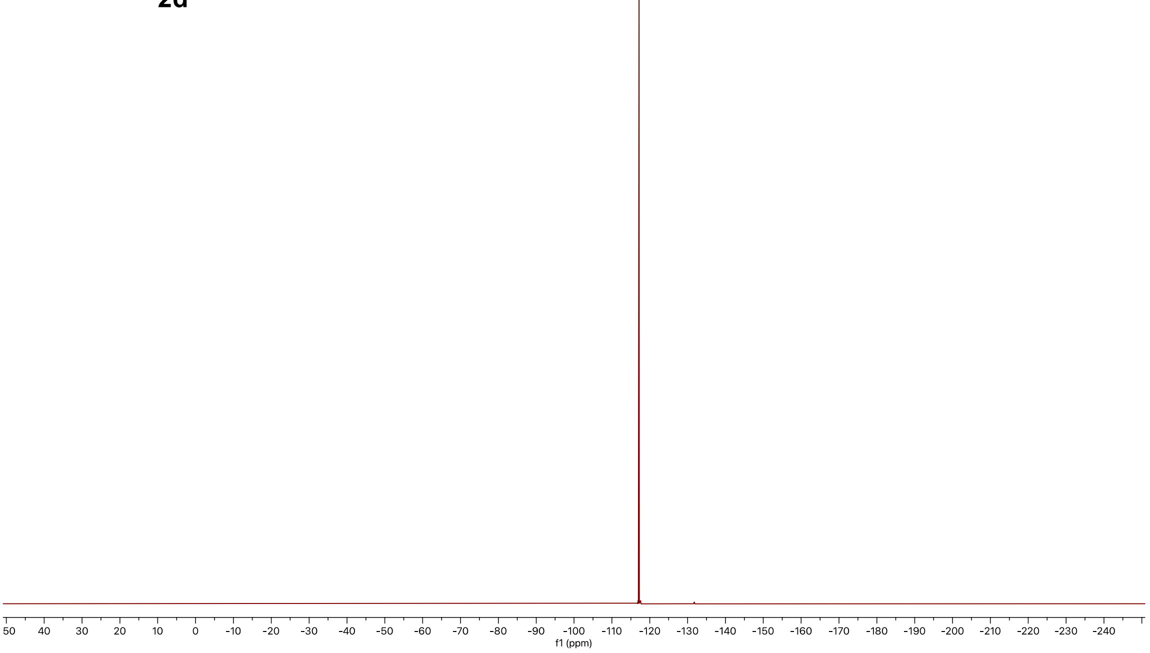

^{13}C NMR (101 MHz, $CDCl_3$)

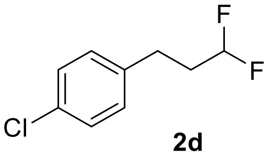

2d

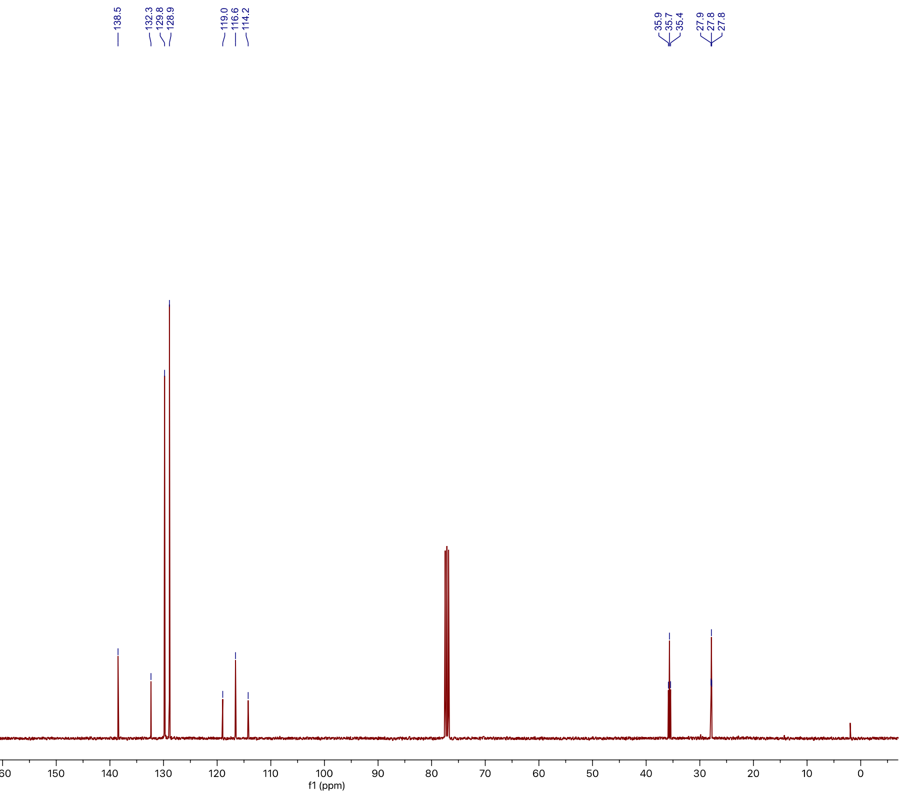

^{1}H NMR (400 MHz, $CDCl_3$)

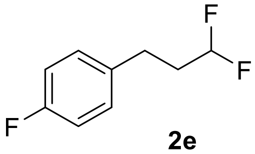

2e

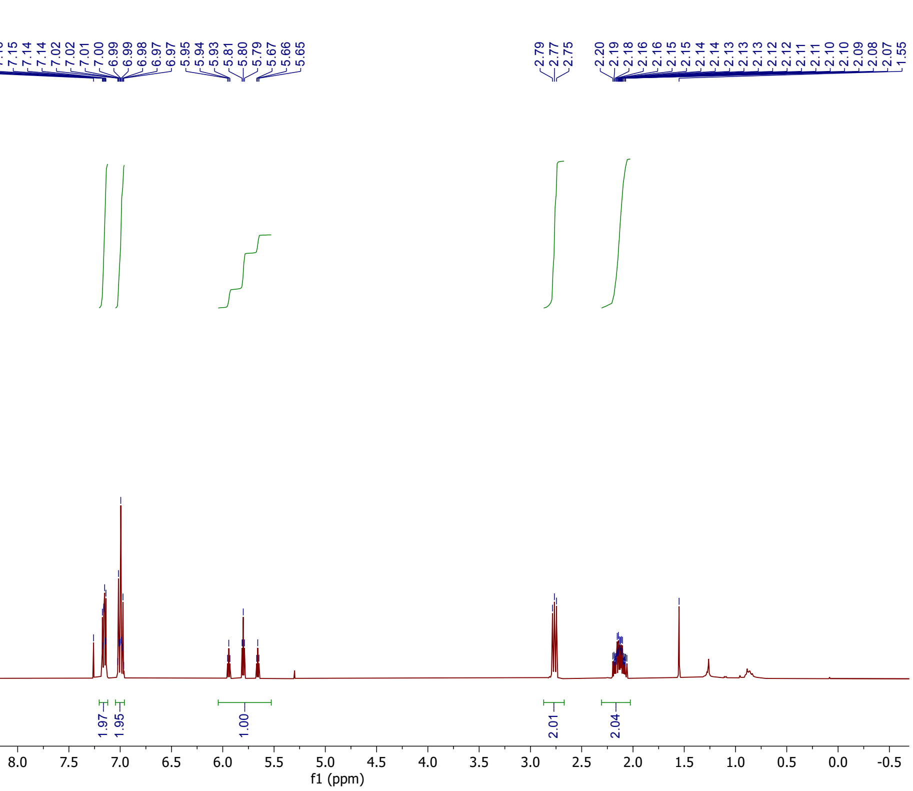

^{19}F NMR (376 MHz, CDCl$_3$)

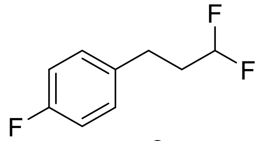

2e

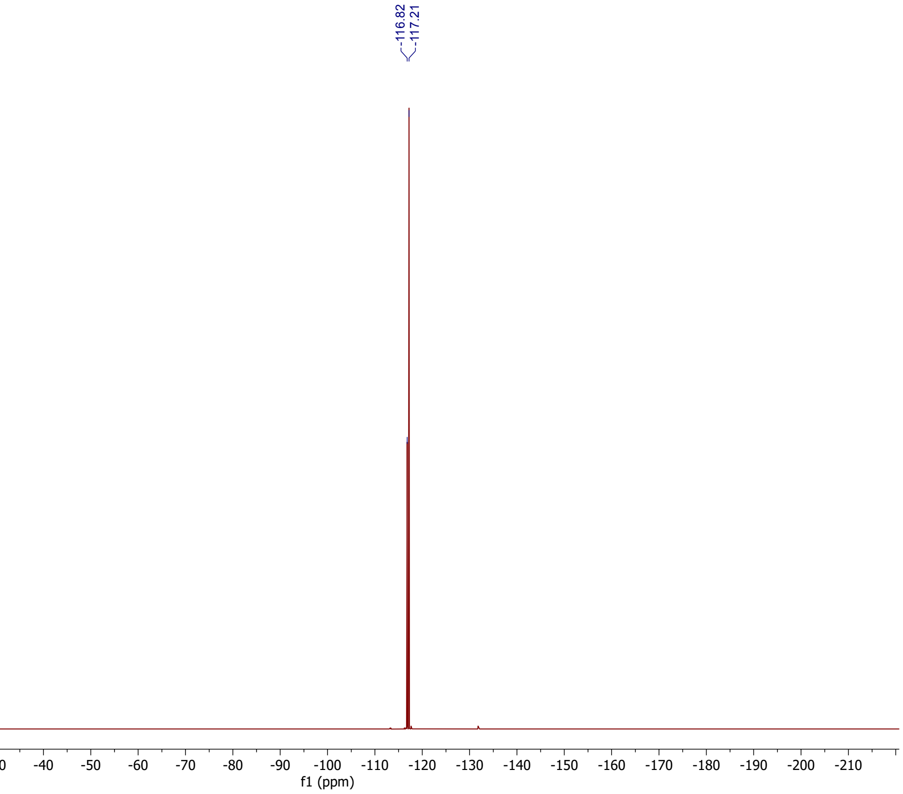

^{13}C NMR (101 MHz, CDCl$_3$)

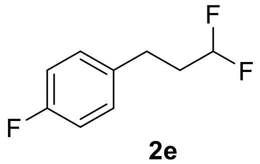

2e

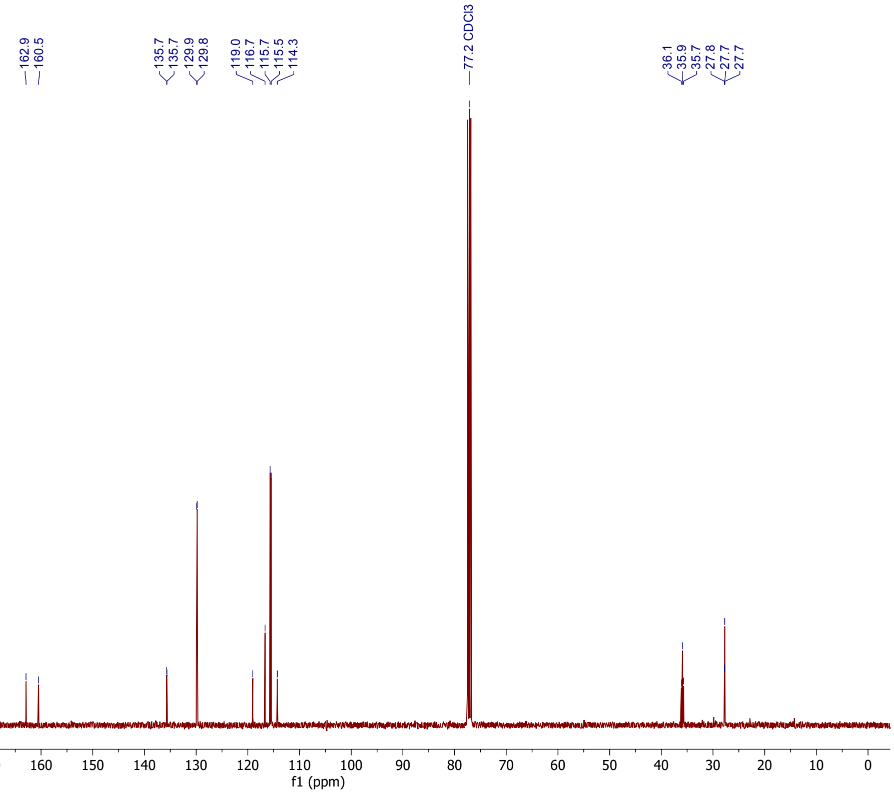

1H NMR (400 MHz, $CDCl_3$)

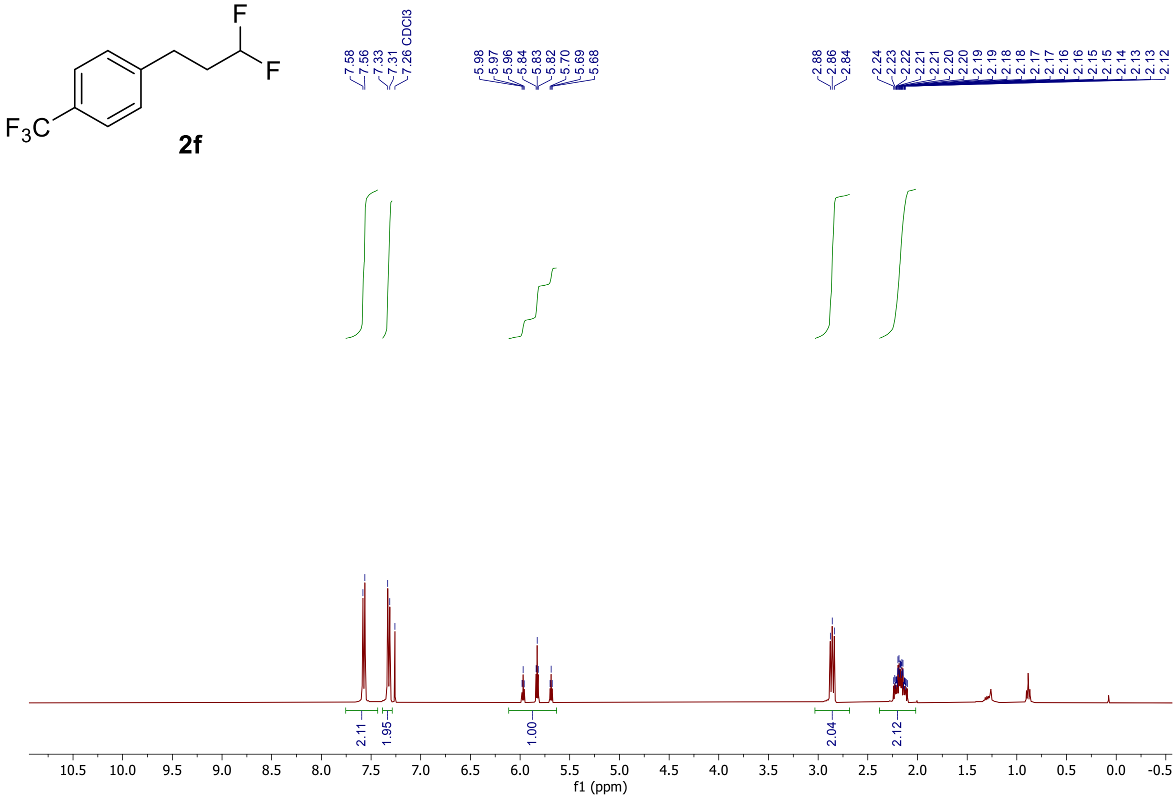

19F NMR (376 MHz, $CDCl_3$)

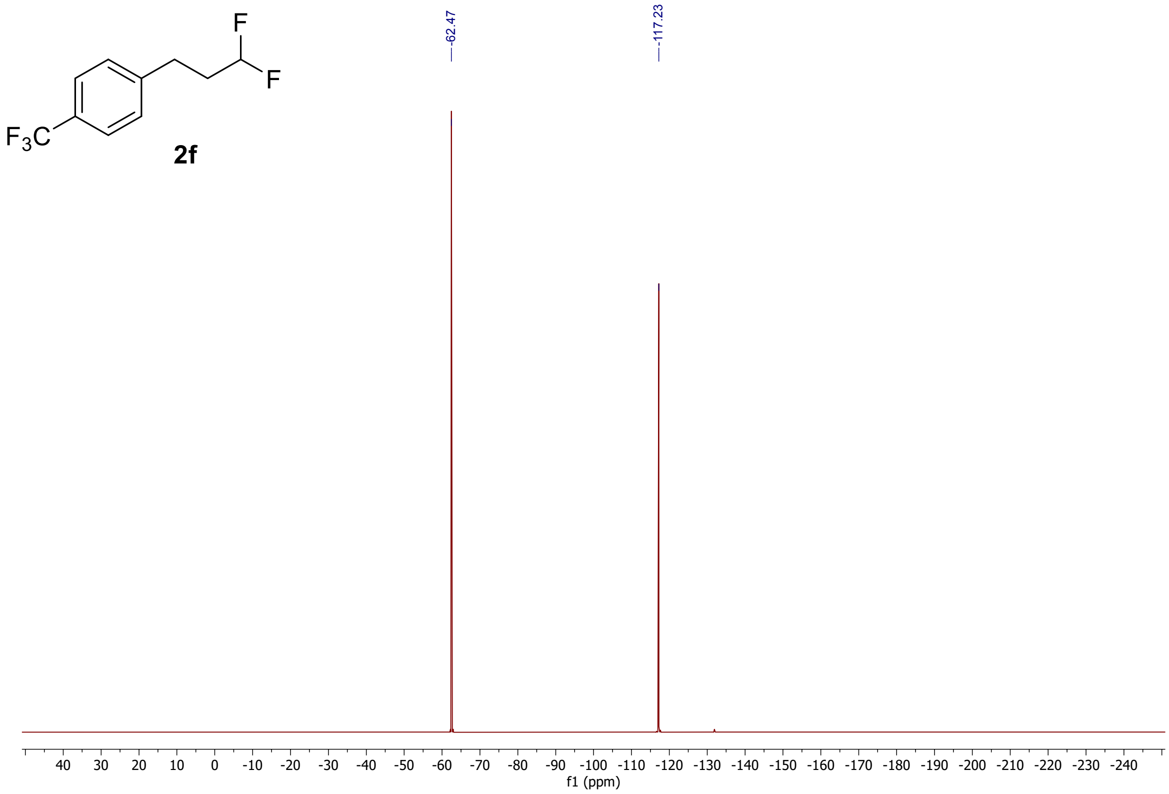

^{13}C NMR (101 MHz, $CDCl_3$)

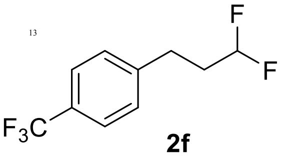

2f

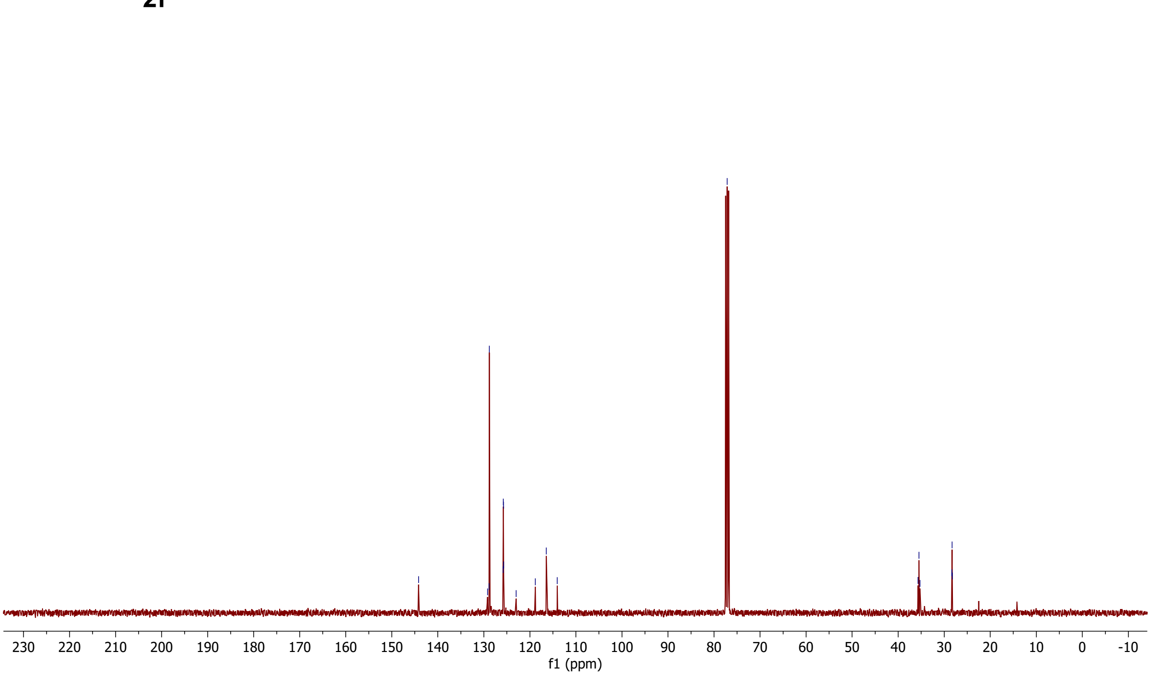

^{1}H NMR (300 MHz, $CDCl_3$)

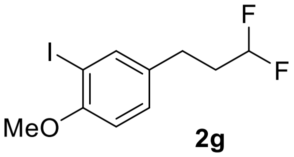

2g

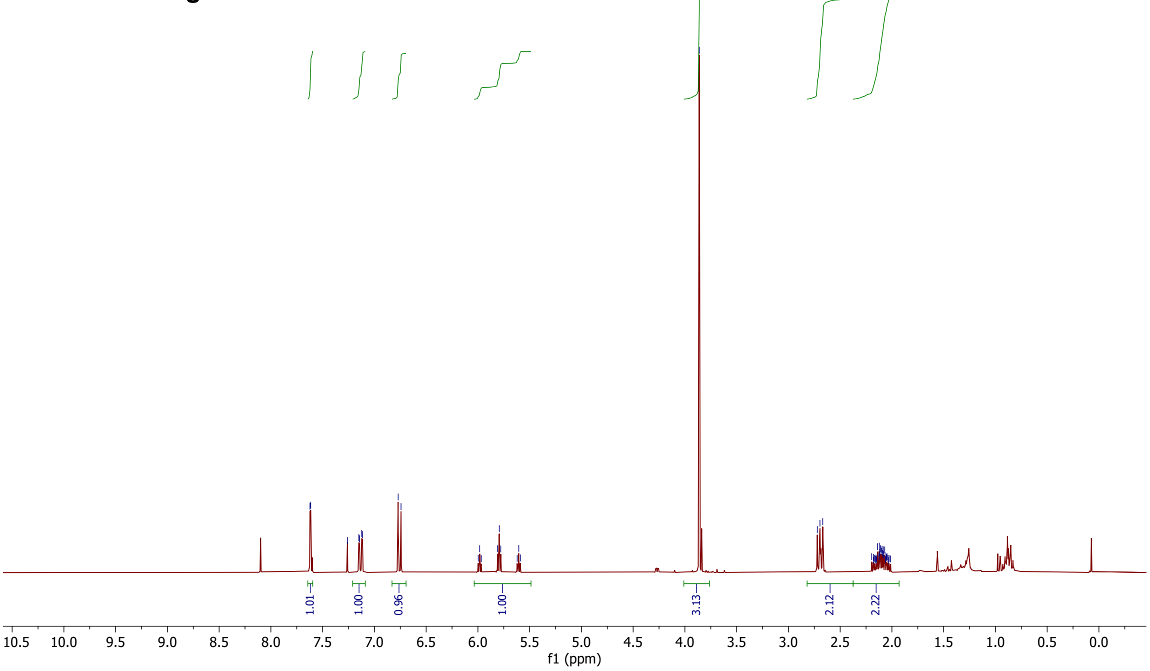

^{19}F NMR (471 MHz, $CDCl_3$)

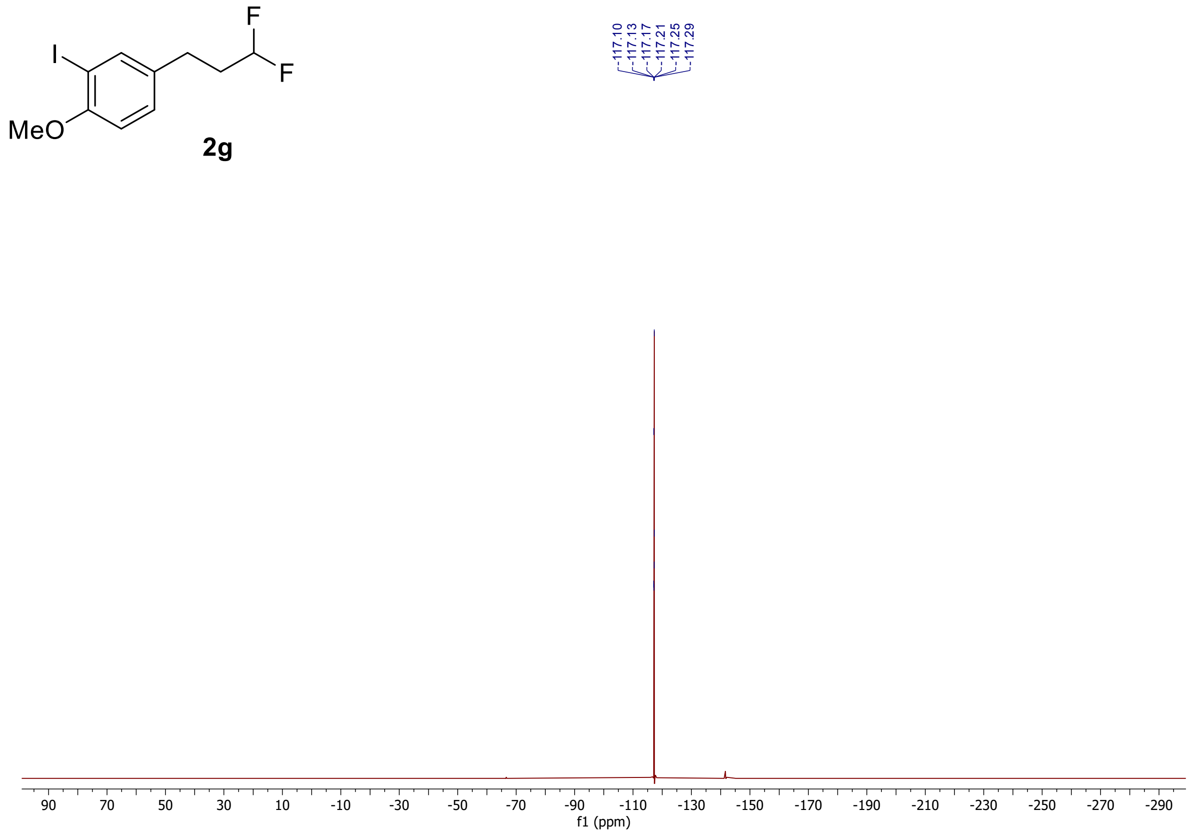

2g

^{13}C NMR (75 MHz, $CDCl_3$)

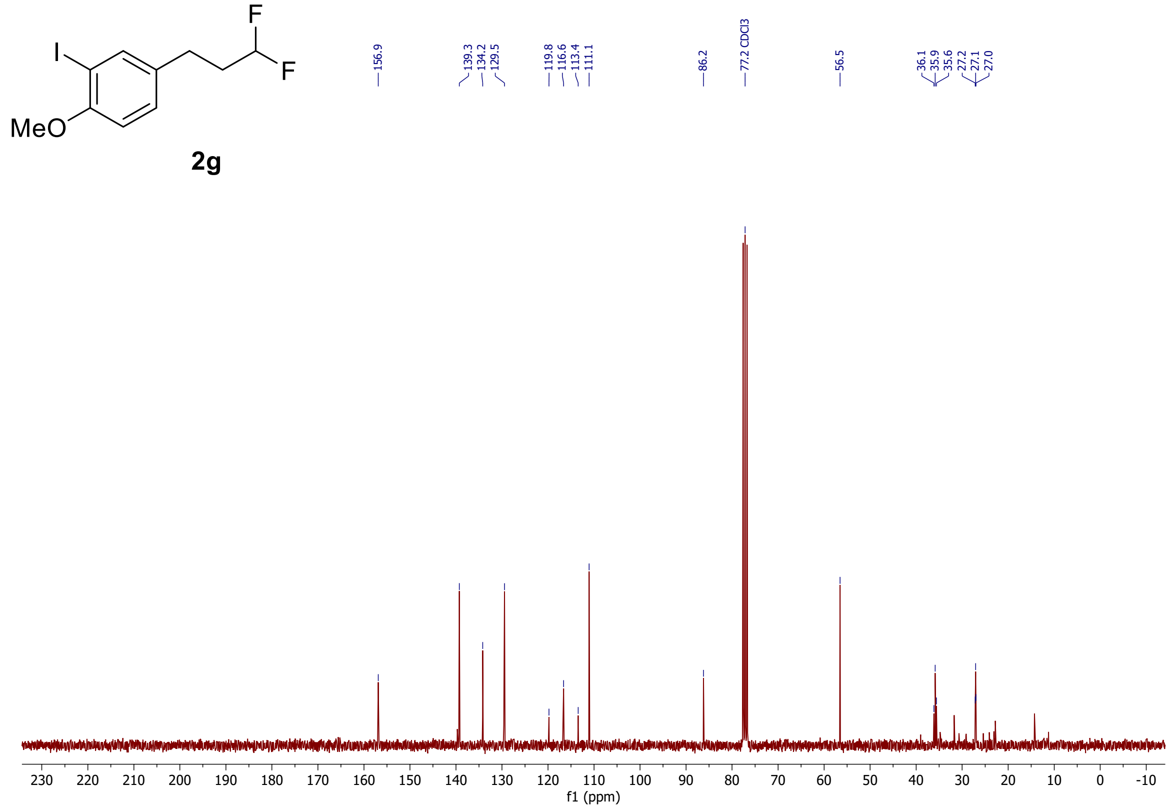

2g

^{1}H NMR (400 MHz, $CDCl_3$)

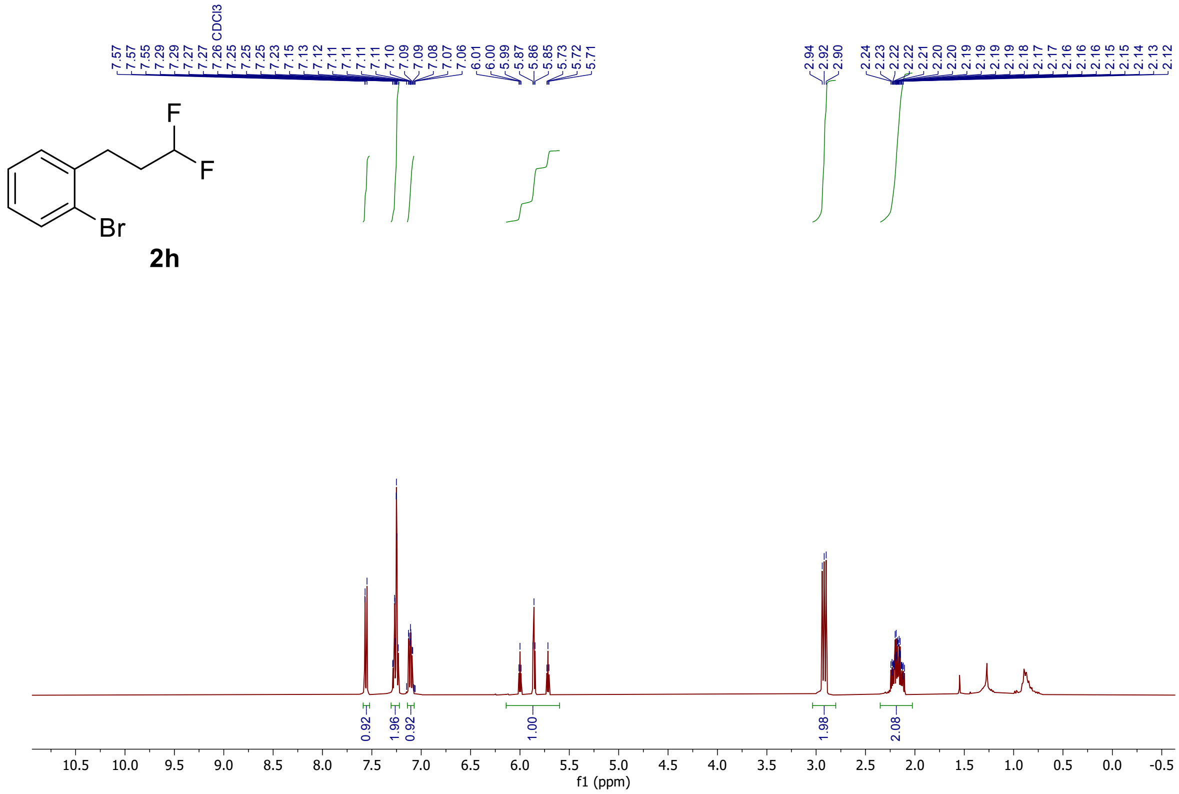

^{19}F NMR (400 MHz, $CDCl_3$)

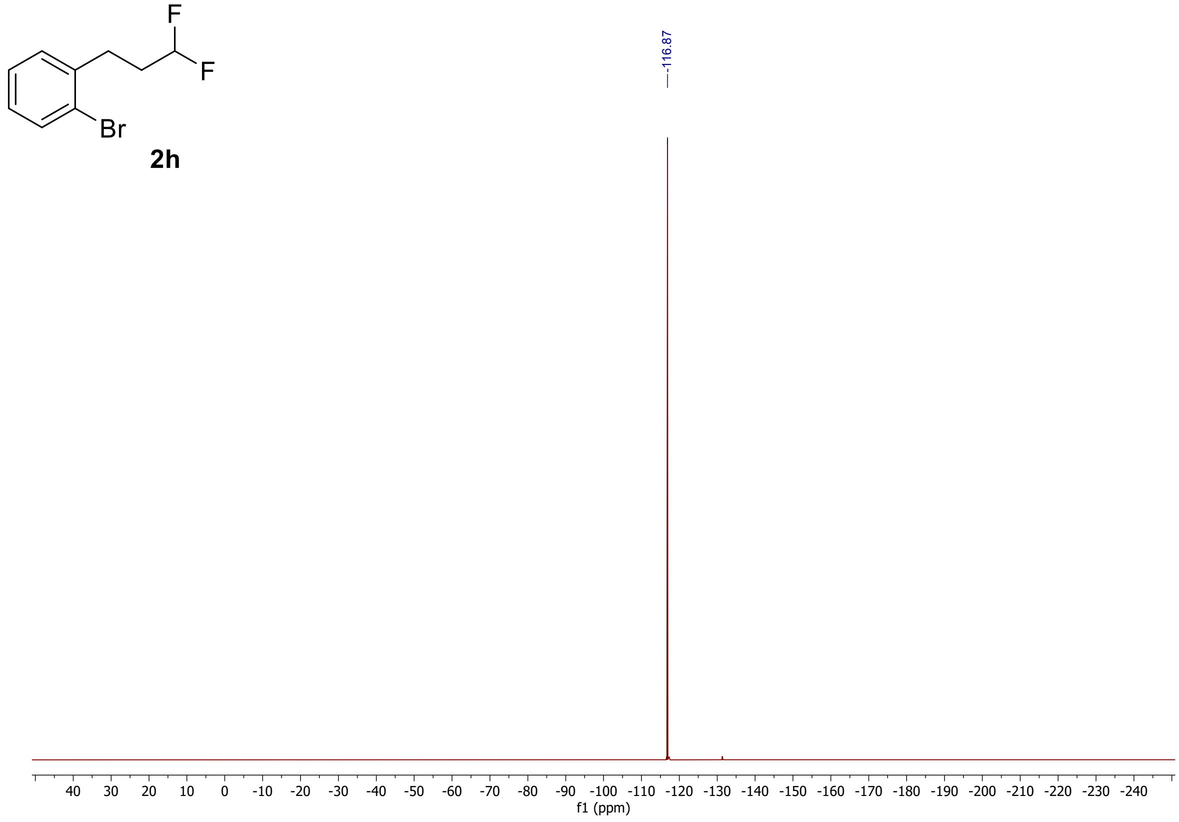

^{13}C NMR (101 MHz, $CDCl_3$)

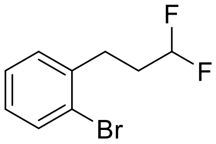

2h

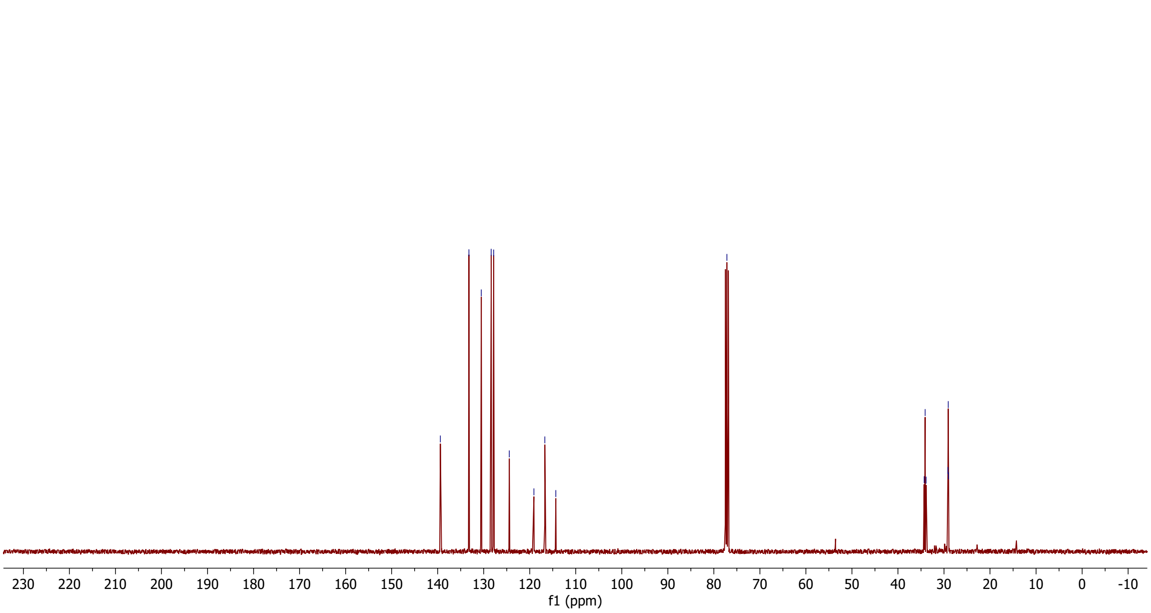

^{1}H NMR (400 MHz, $CDCl_3$)

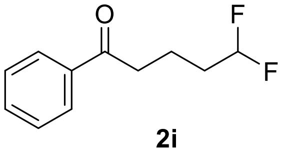

2i

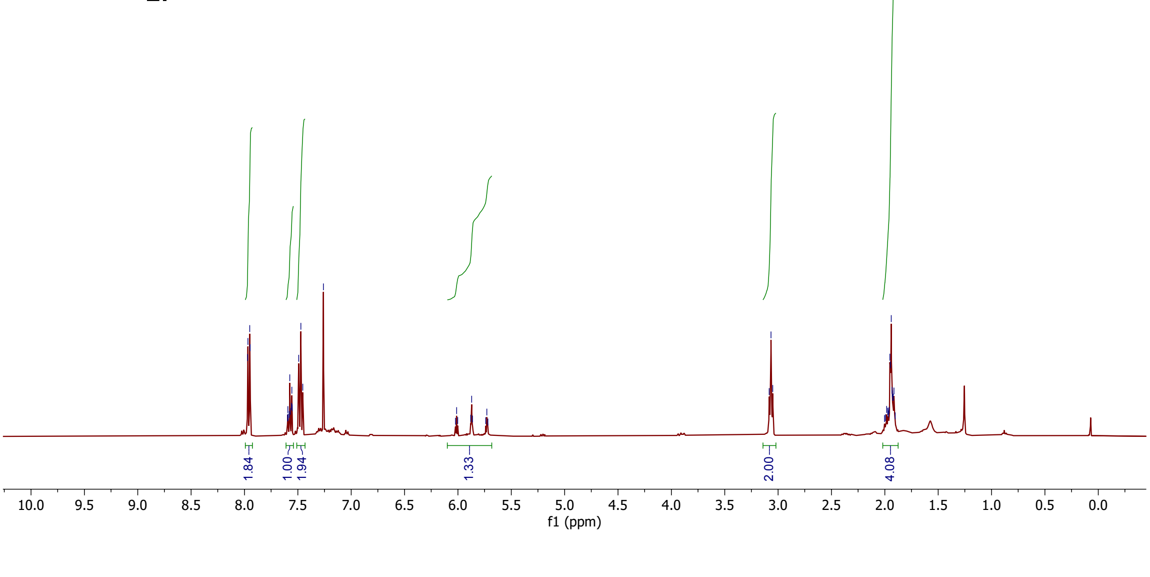

^{19}F NMR (376 MHz, CDCl$_3$)

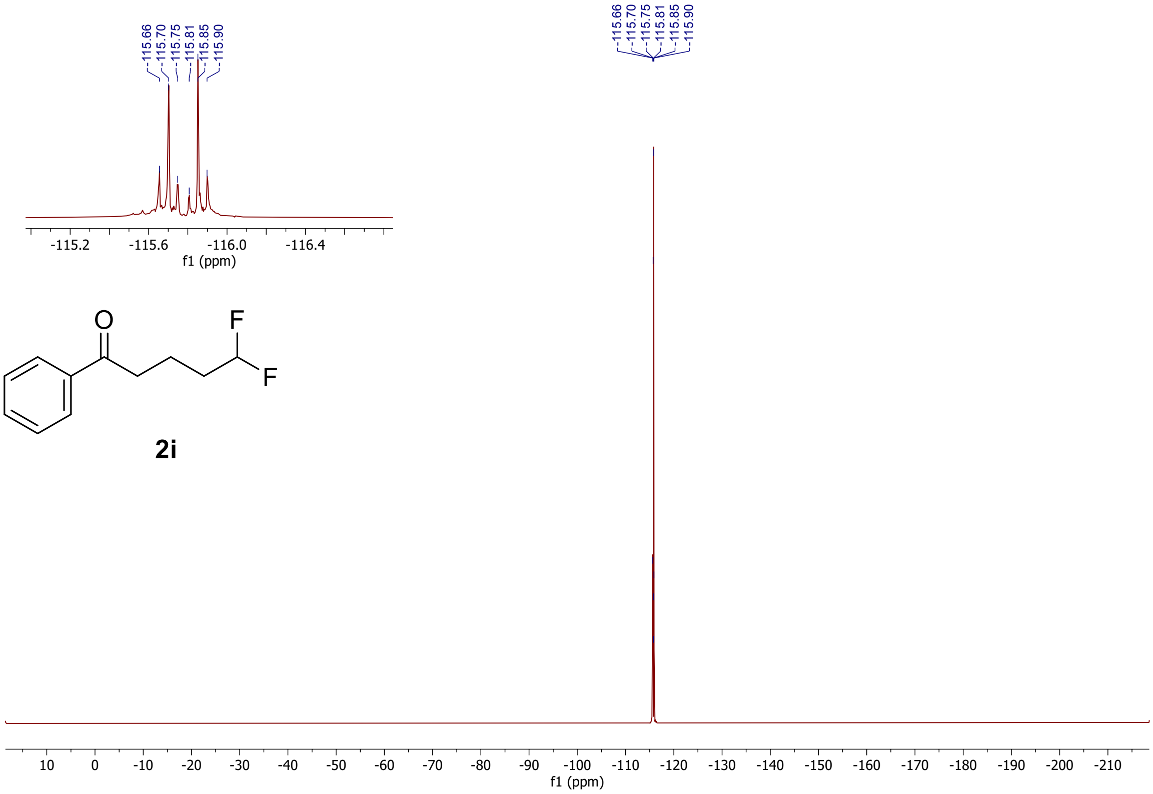

2i

^{13}C NMR (101 MHz, CDCl$_3$)

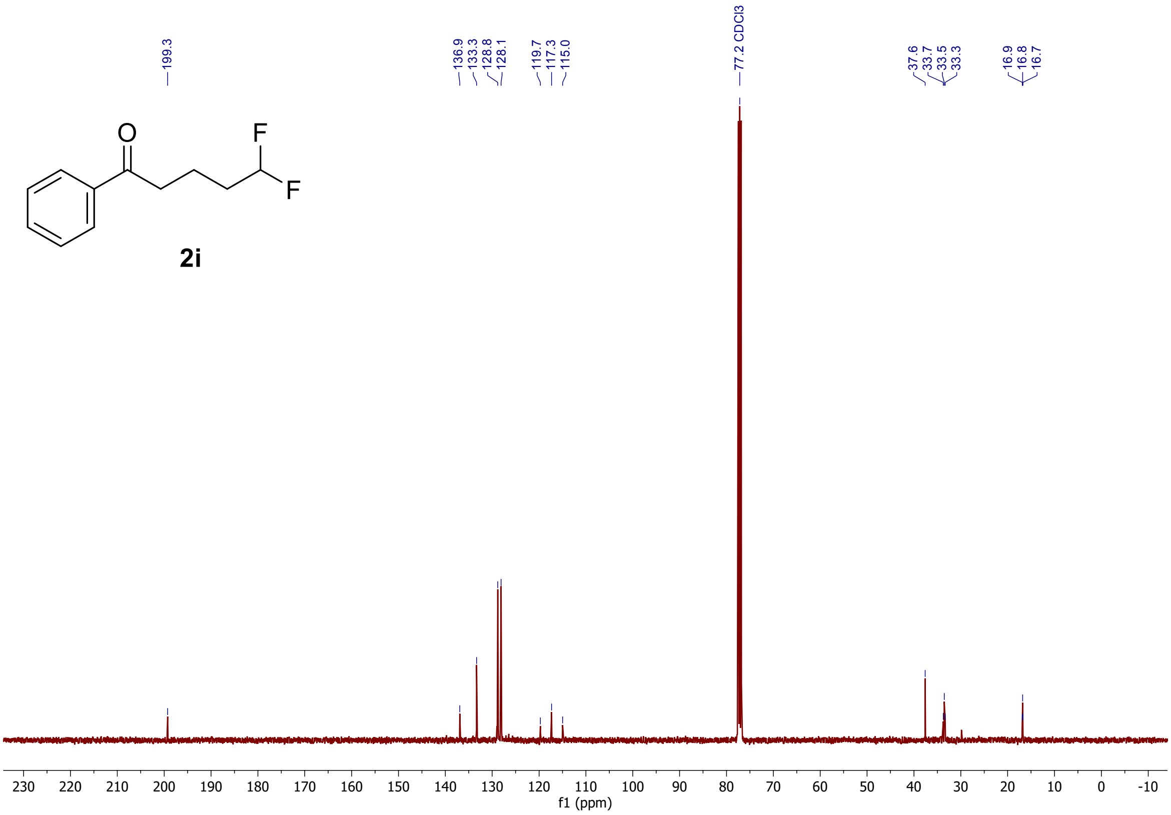

2i

^{1}H NMR (400 MHz, $CDCl_3$)

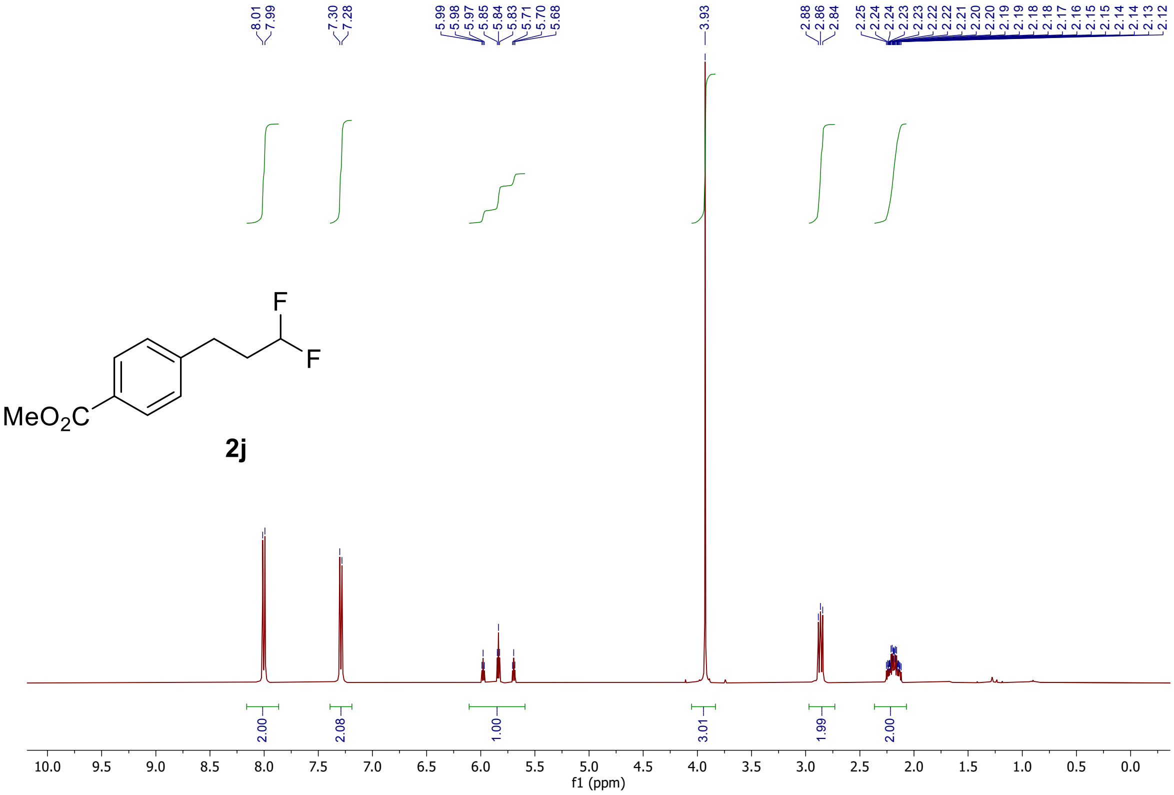

^{19}F NMR (376 MHz, $CDCl_3$)

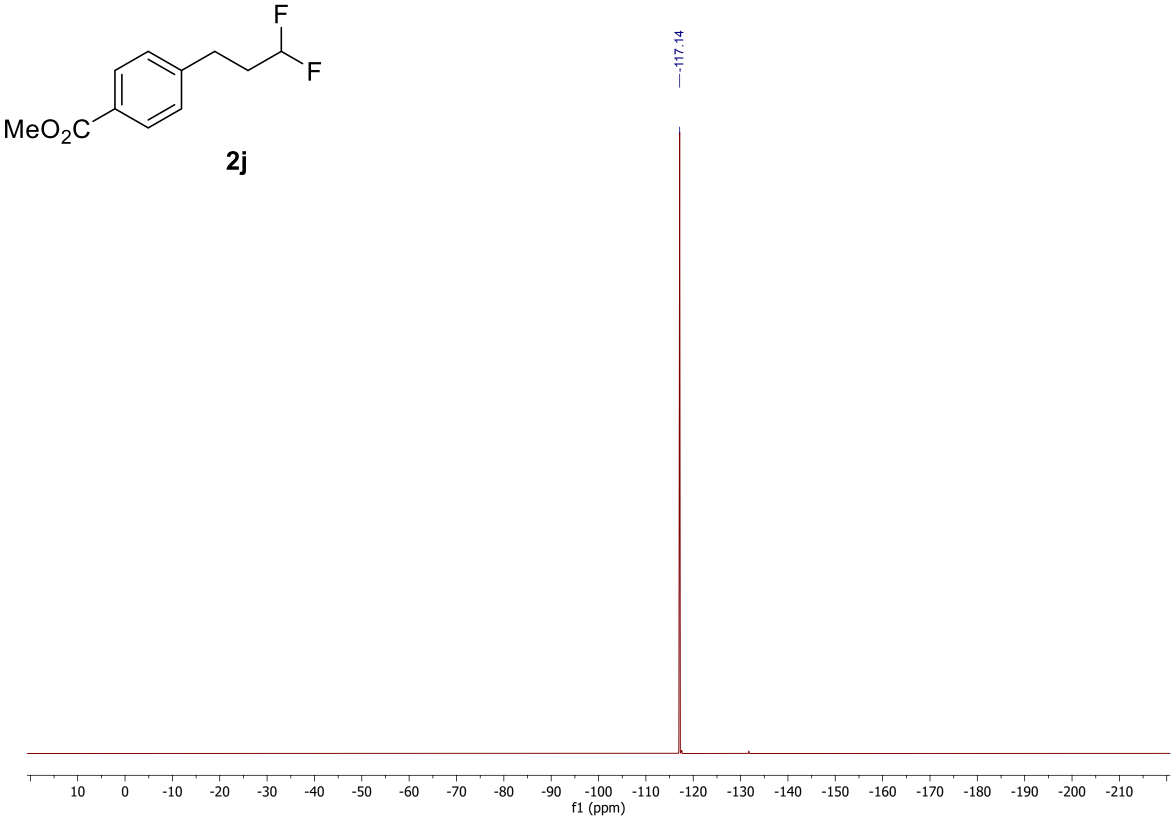

^{13}C NMR (101 MHz, $CDCl_3$)

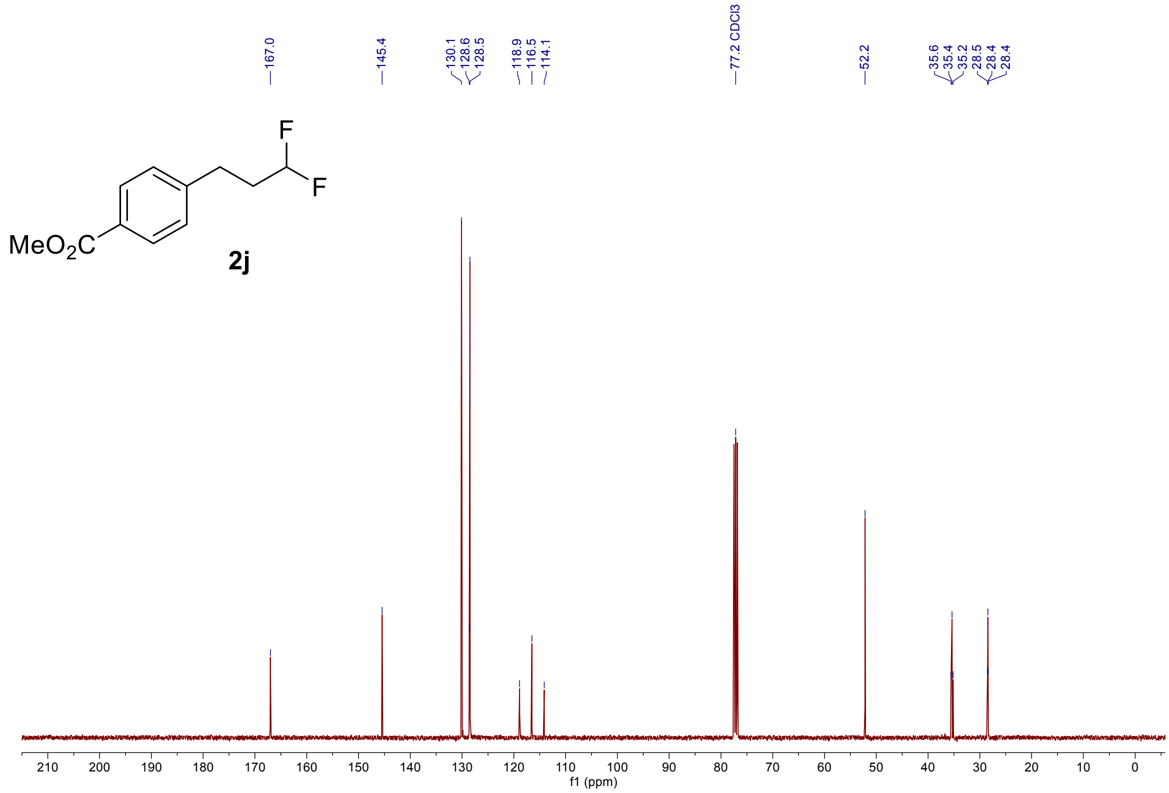

2j

^{1}H NMR (400 MHz, $CDCl_3$)

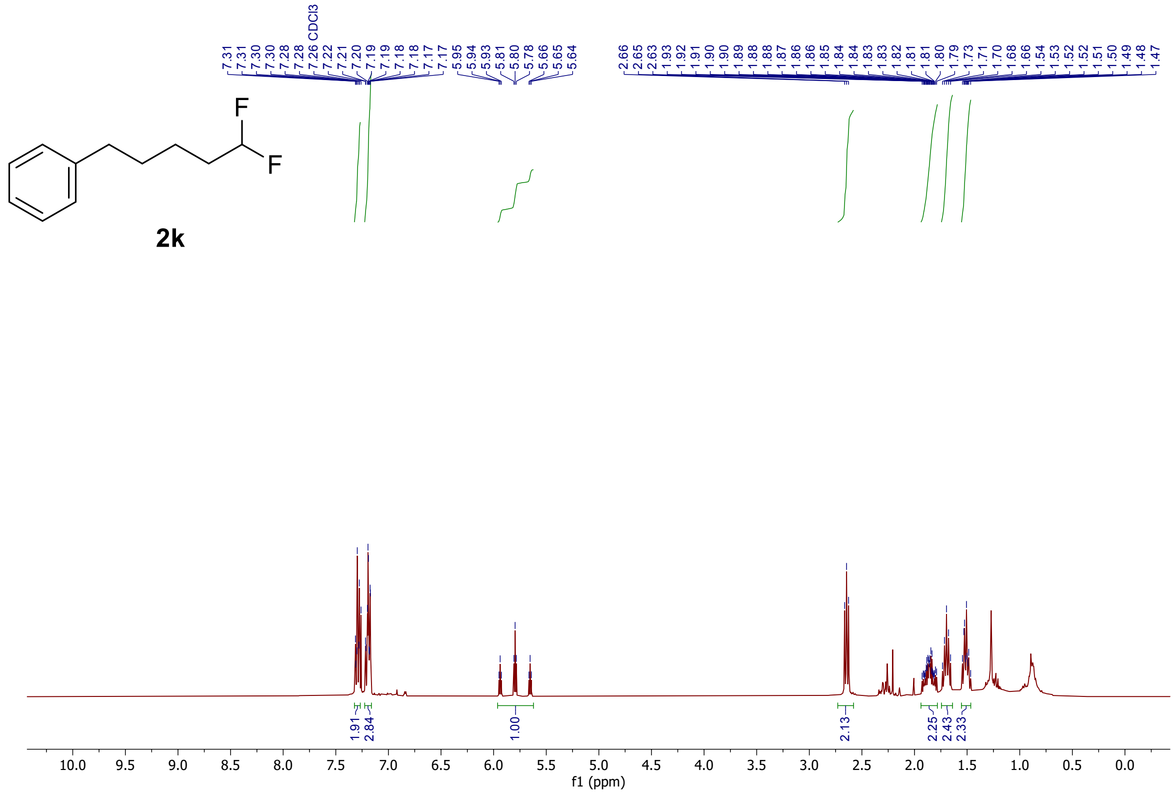

2k

^{19}F NMR (376 MHz, $CDCl_3$)

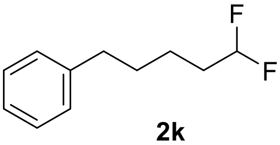

2k

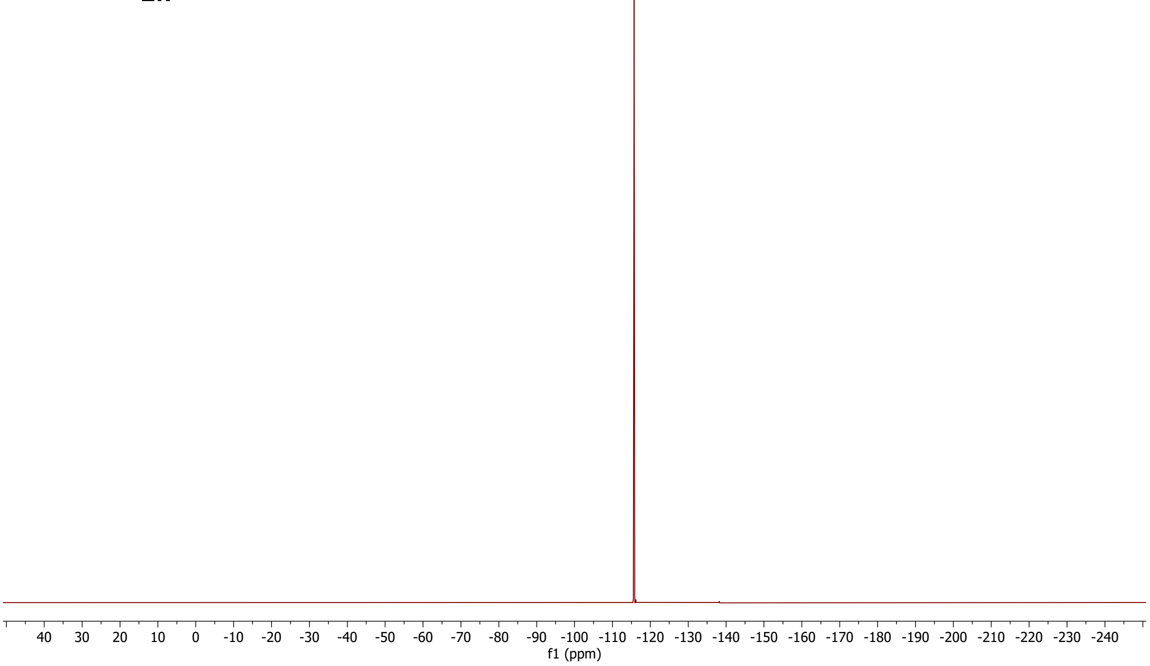

^{13}C NMR (101 MHz, $CDCl_3$)

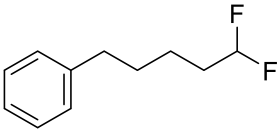

2k

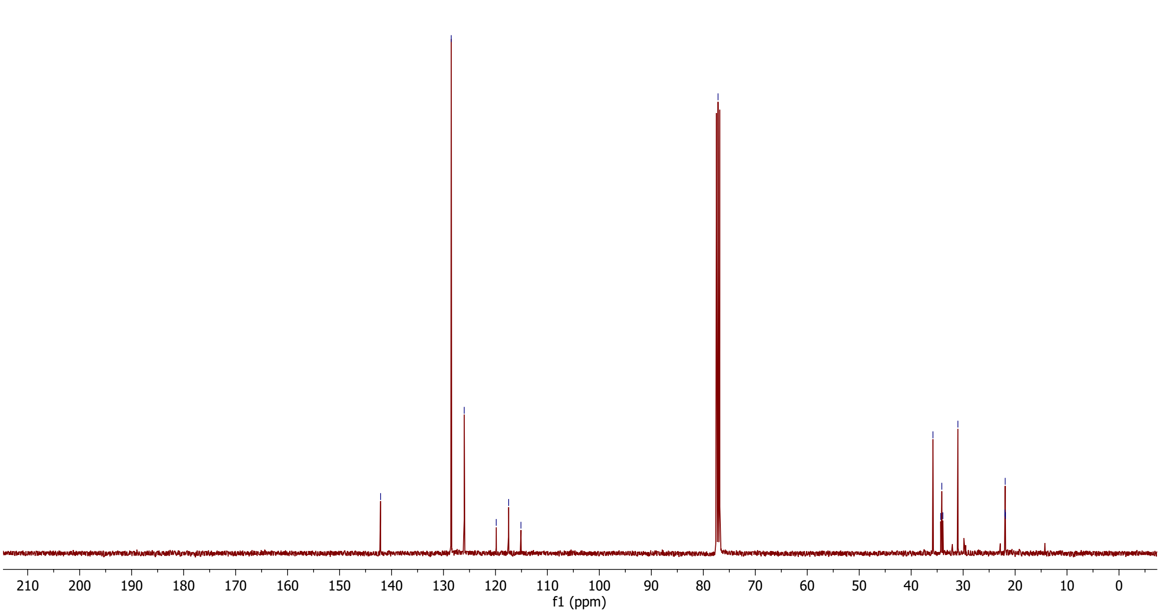

^{1}H NMR (400 MHz, $CDCl_3$)

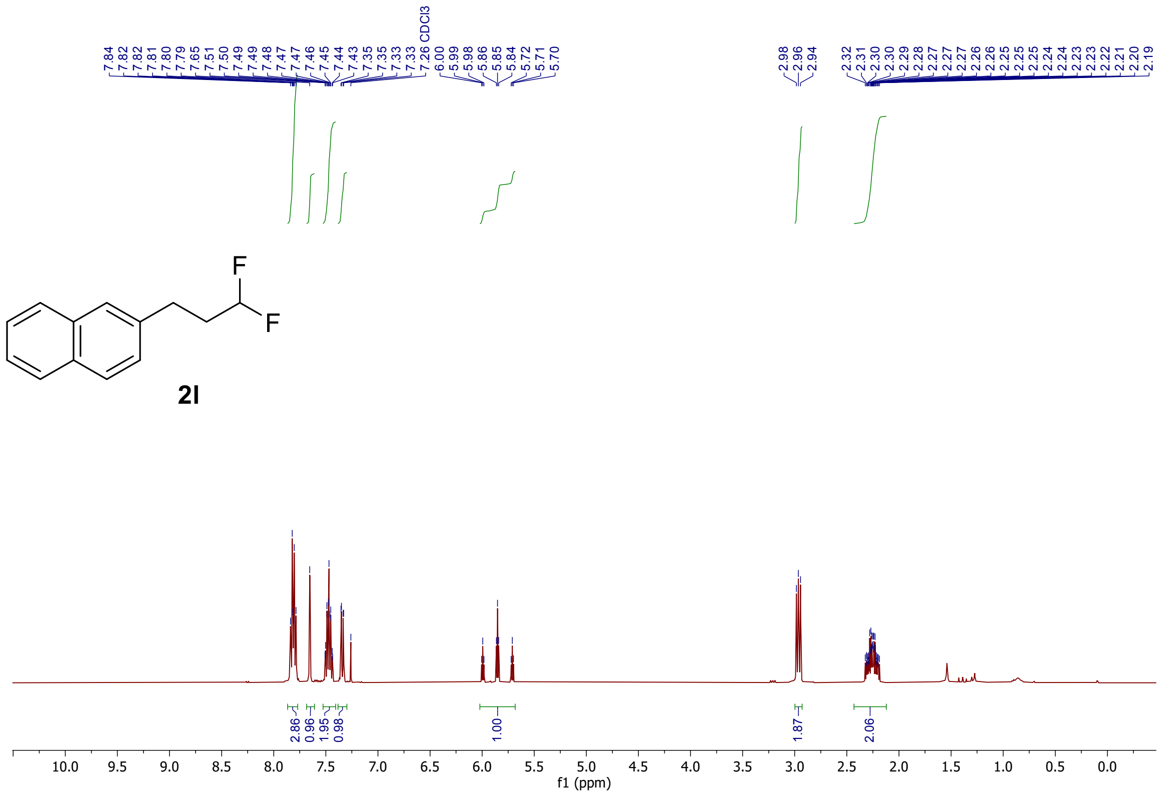

2l

^{19}F NMR (376 MHz, $CDCl_3$)

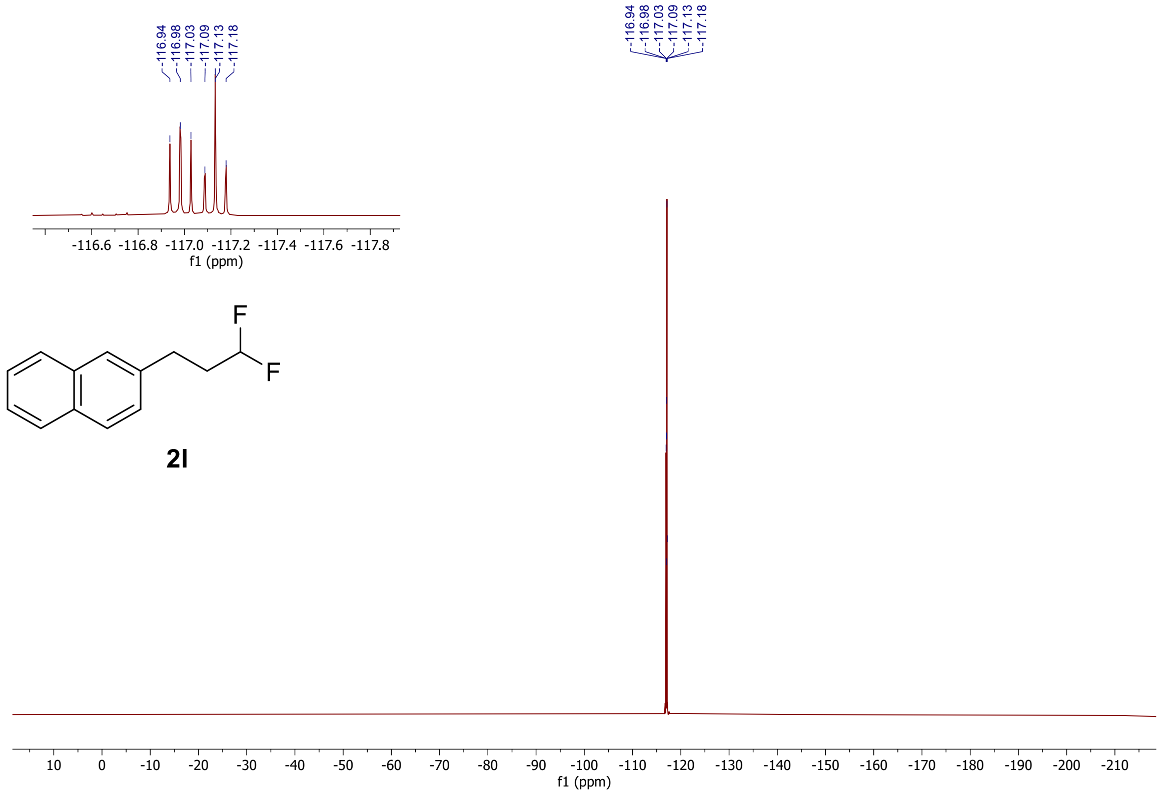

2l

^{13}C NMR (101 MHz, $CDCl_3$)

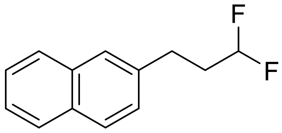

2l

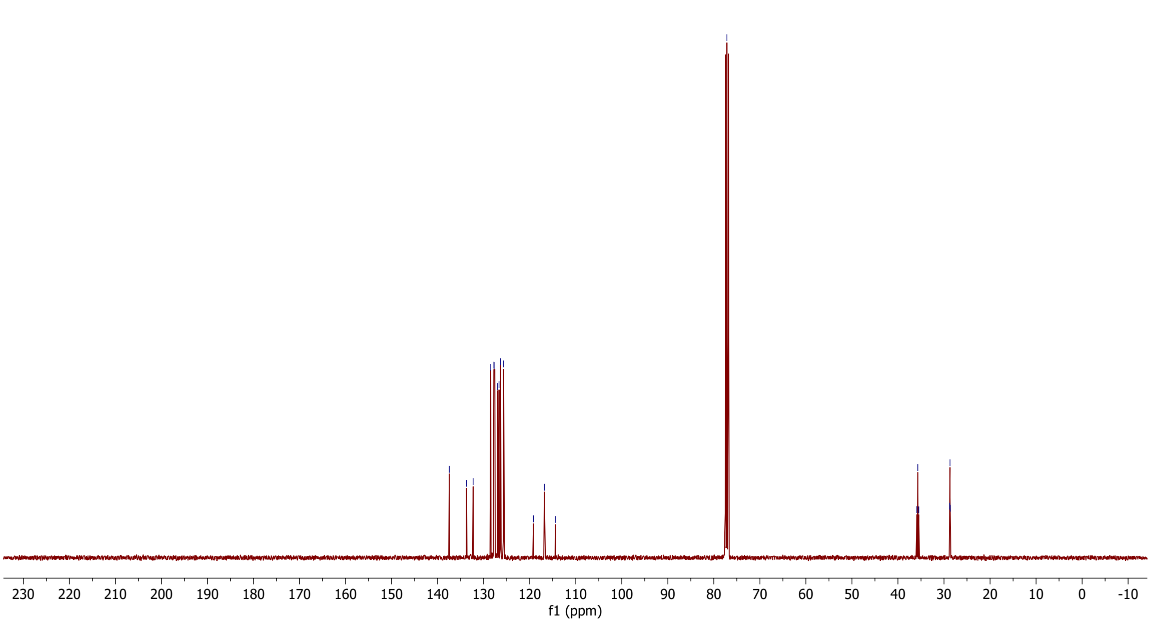

^{1}H NMR (400 MHz, $CDCl_3$)

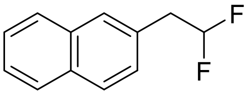

2m

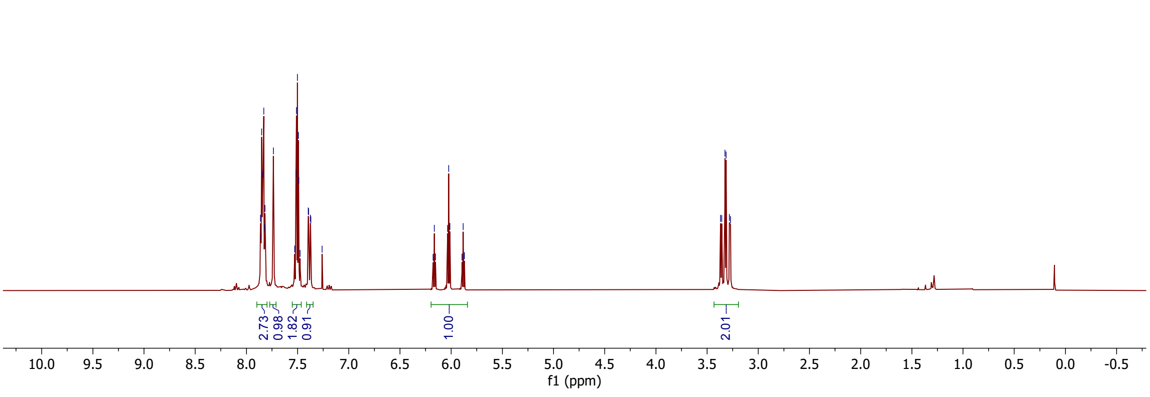

^{19}F NMR (471 MHz, $CDCl_3$)

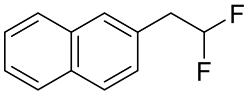

2m

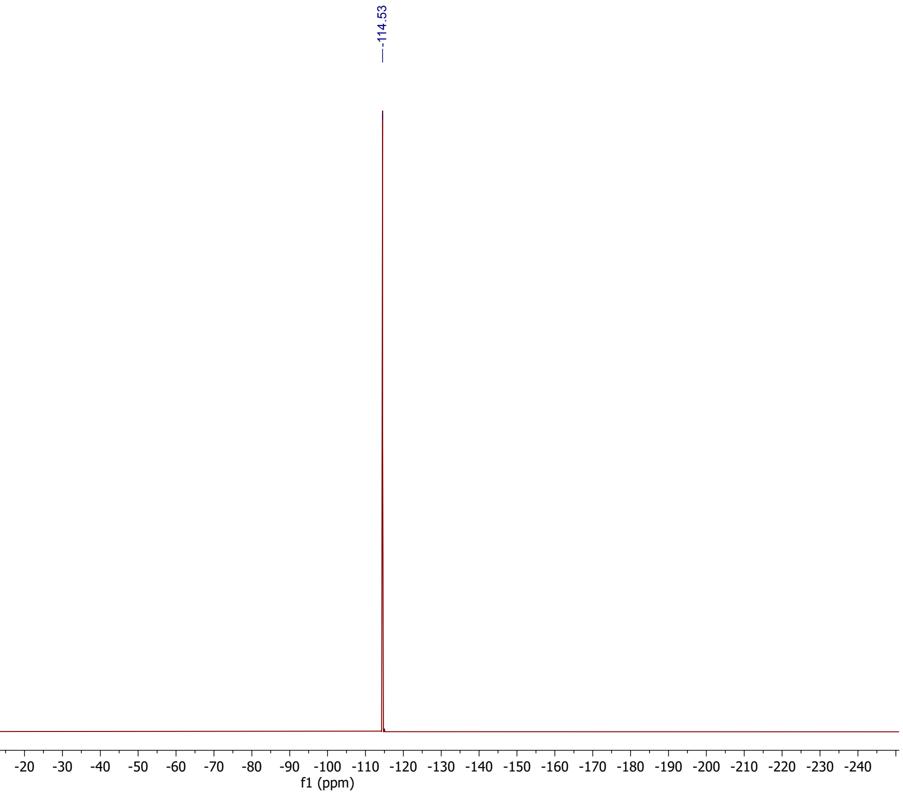

^{13}C NMR (101 MHz, $CDCl_3$)

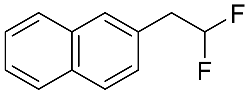

2m

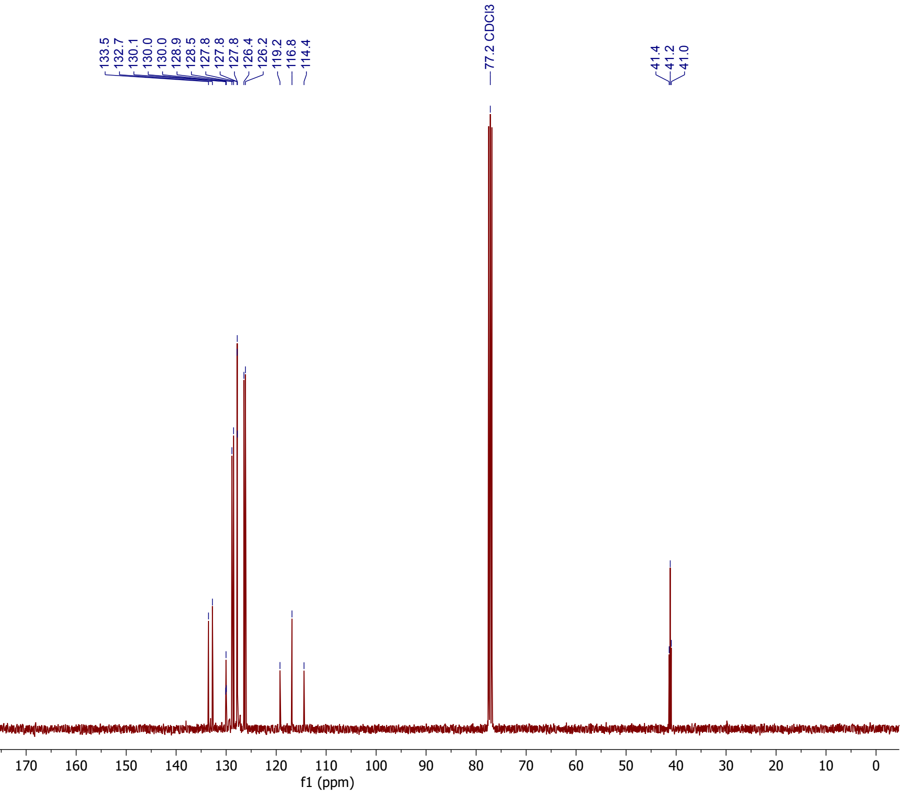

^{1}H NMR (400 MHz, $CDCl_3$)

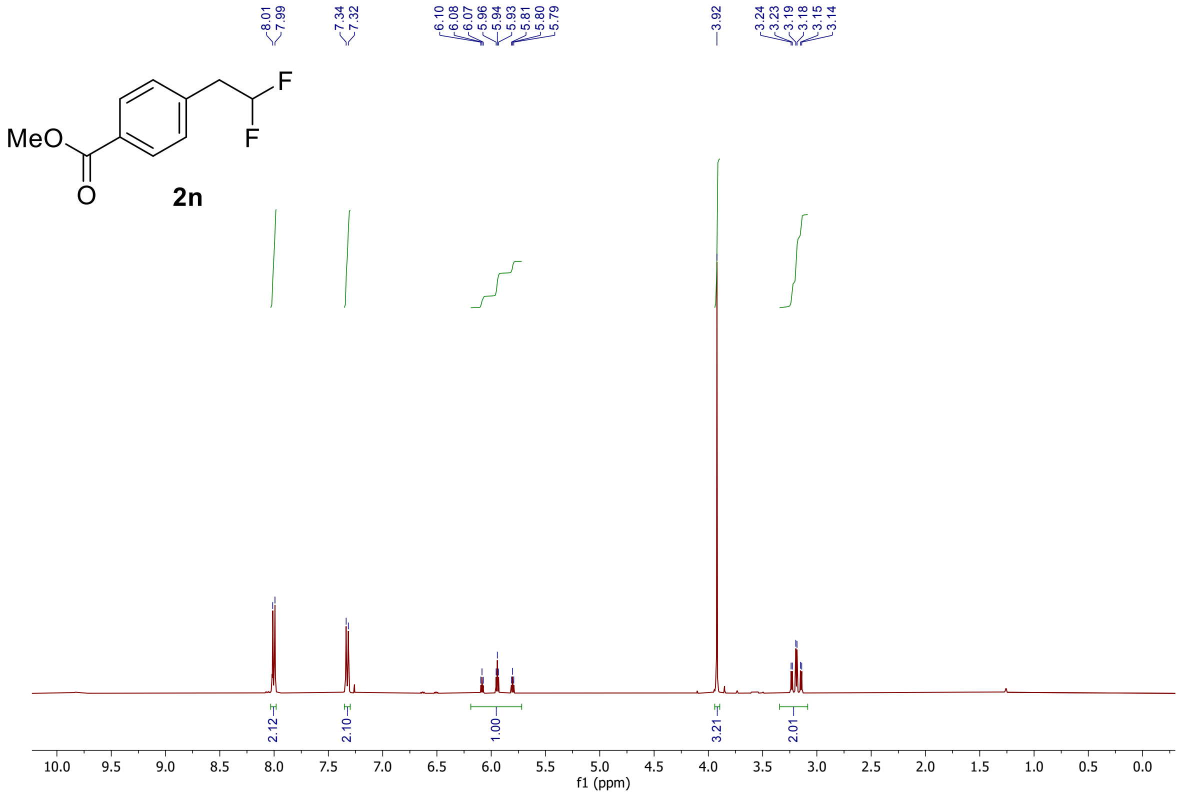

2n

^{19}F NMR (376 MHz, $CDCl_3$)

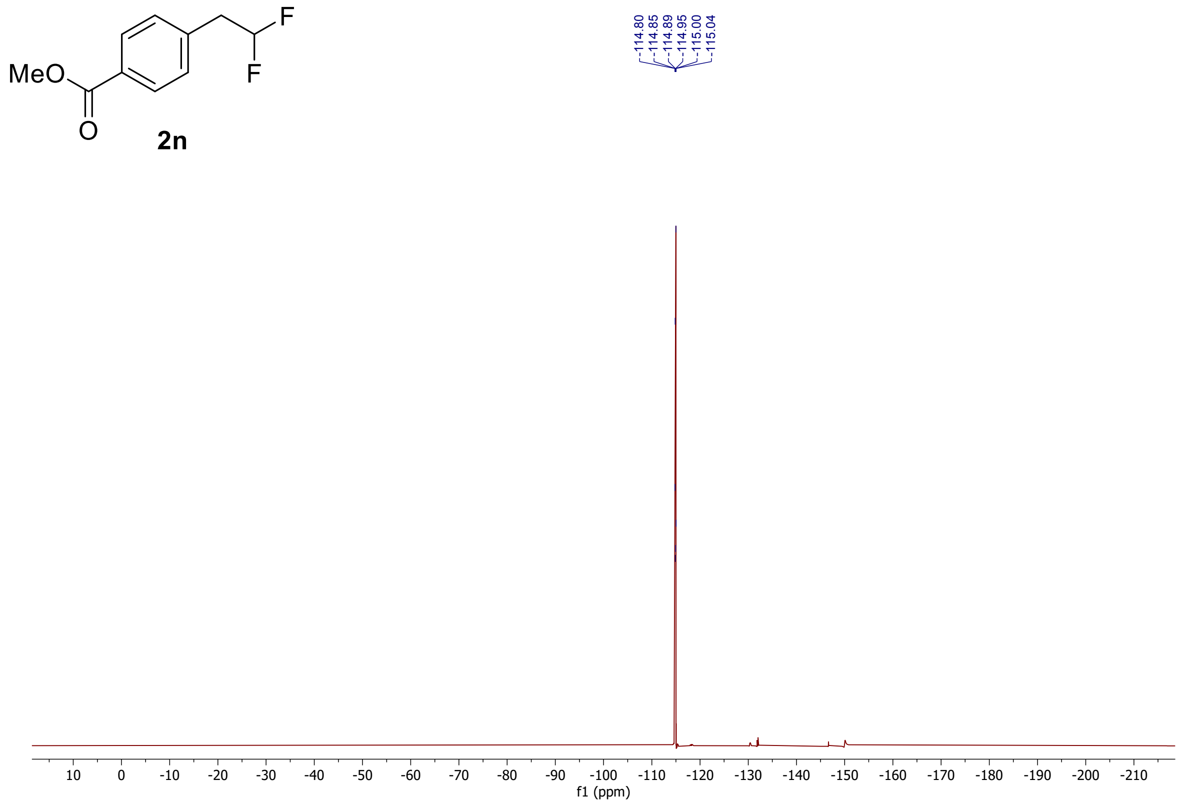

2n

^{13}C NMR (101 MHz, $CDCl_3$)

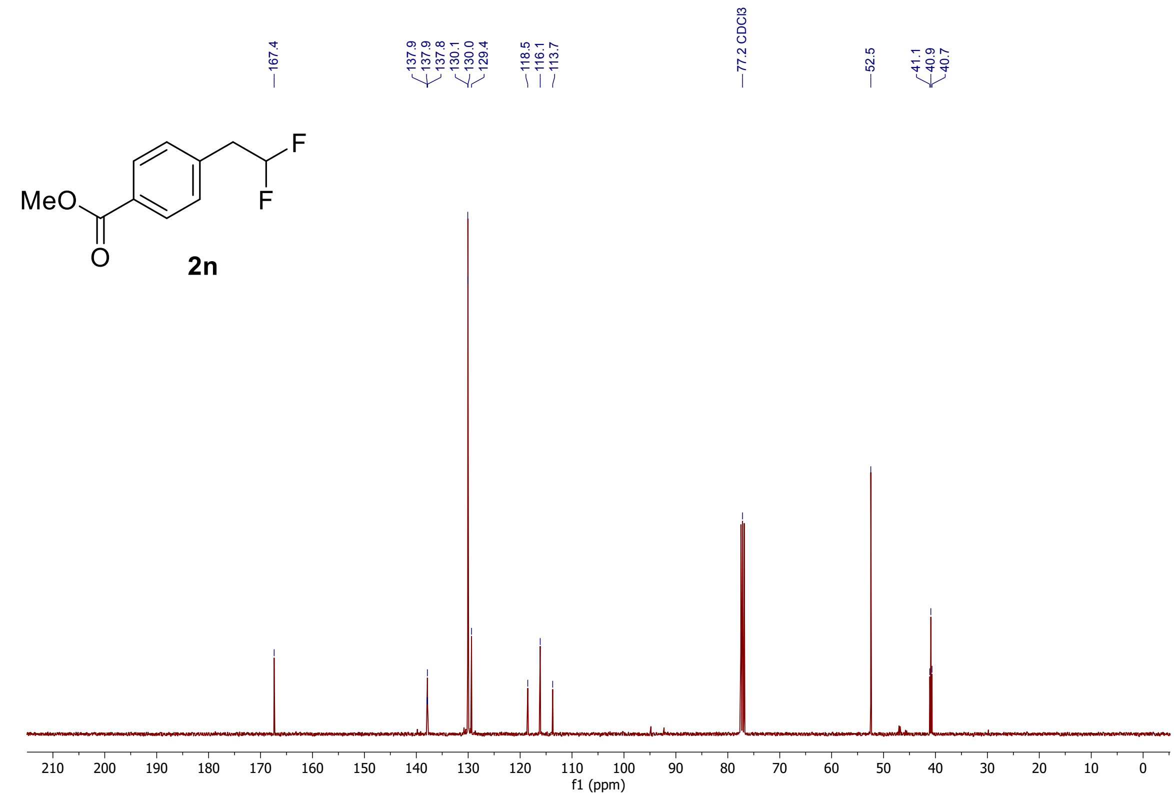

^{1}H NMR (400 MHz, $CDCl_3$)

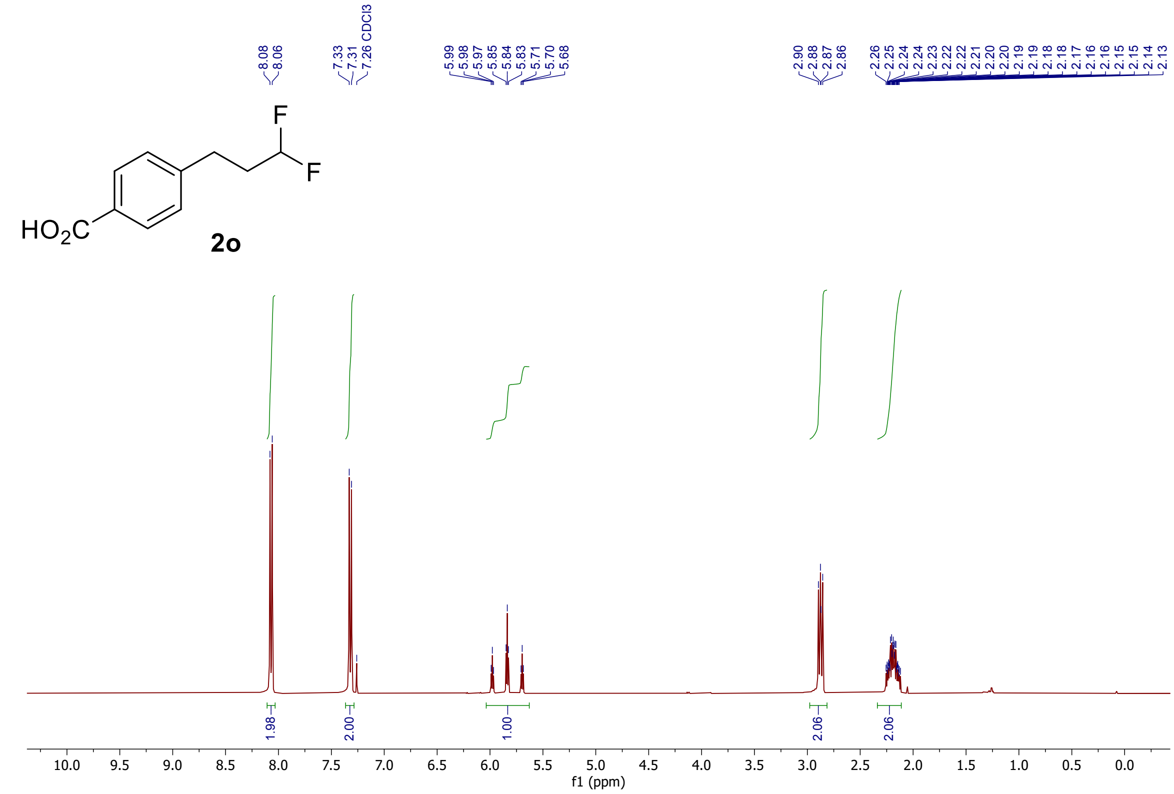

^{19}F NMR (376 MHz, $CDCl_3$)

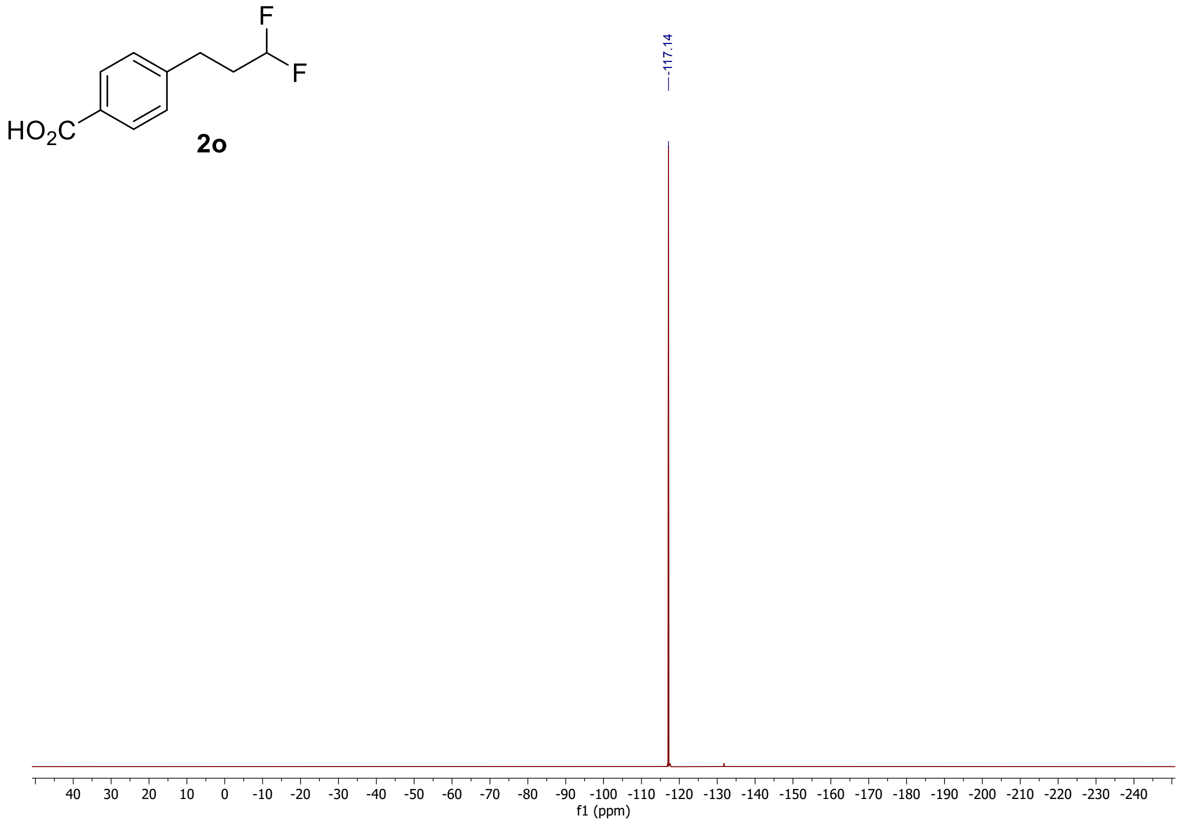

2o

^{13}C NMR (101 MHz, $CDCl_3$)

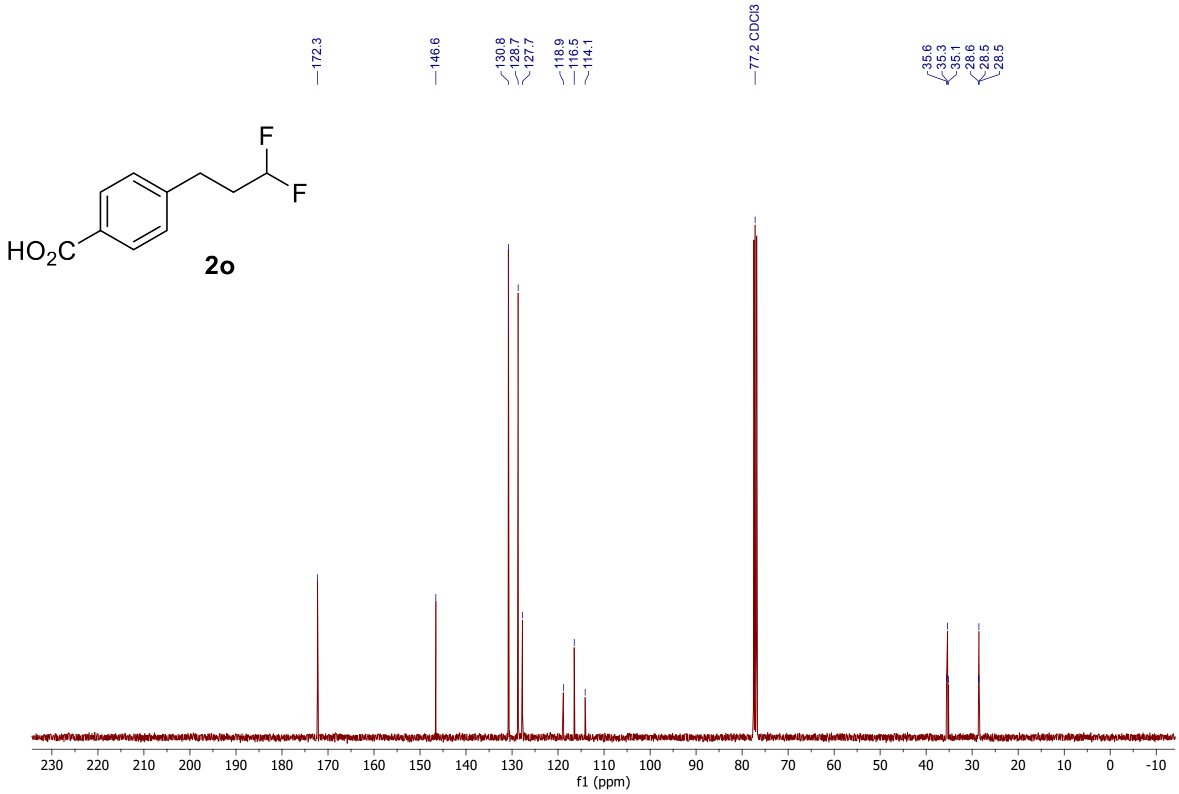

2o

^{1}H NMR (400 MHz, $CDCl_3$)

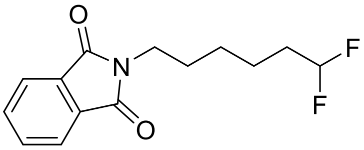

2p

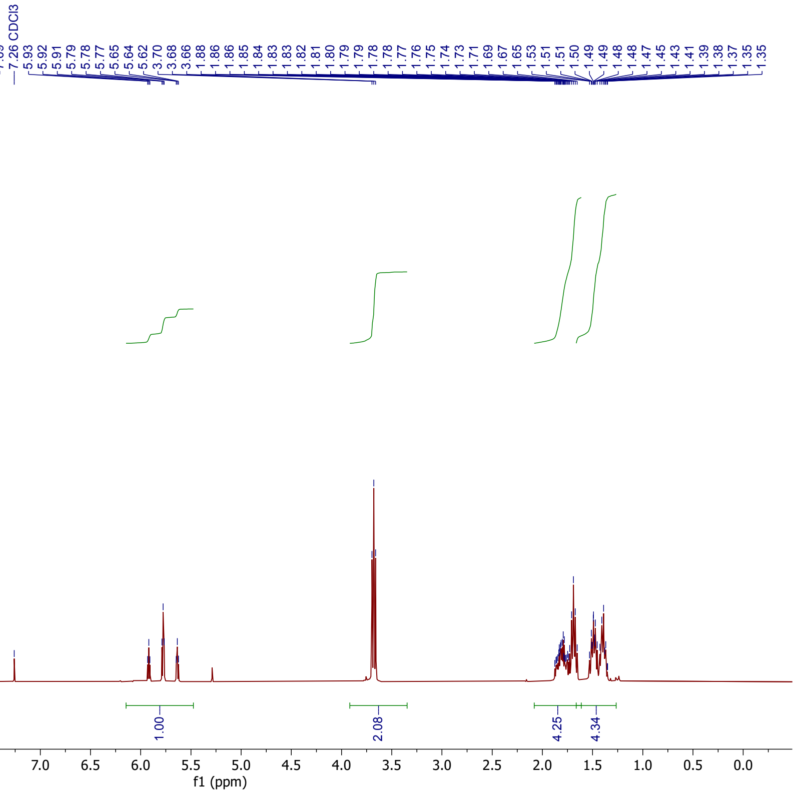

^{19}F NMR (376 MHz, $CDCl_3$)

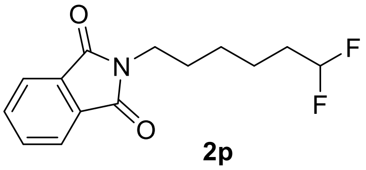

2p

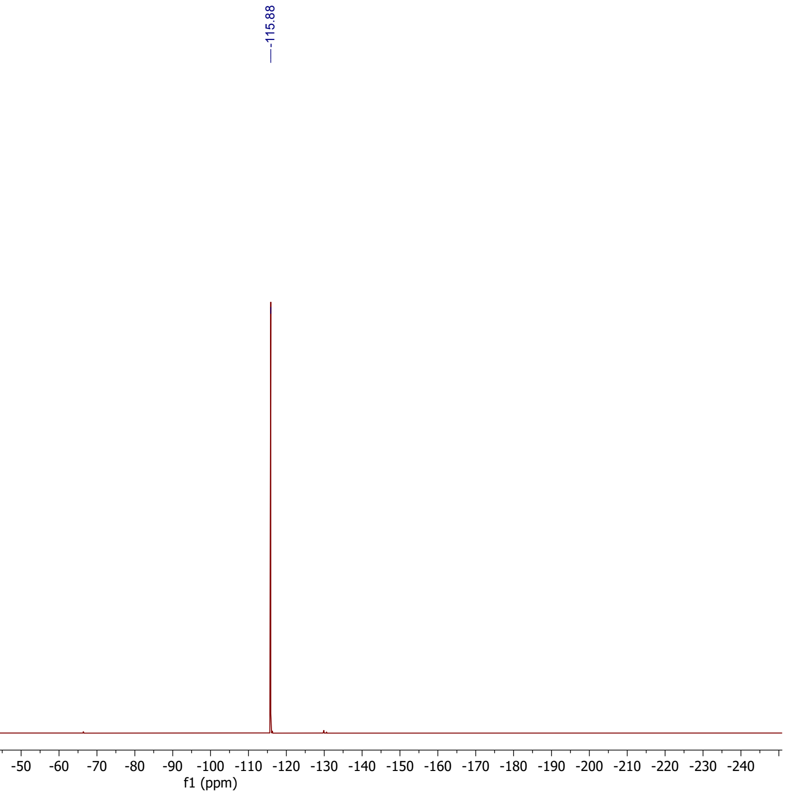

^{13}C NMR (101 MHz, CDCl$_3$)

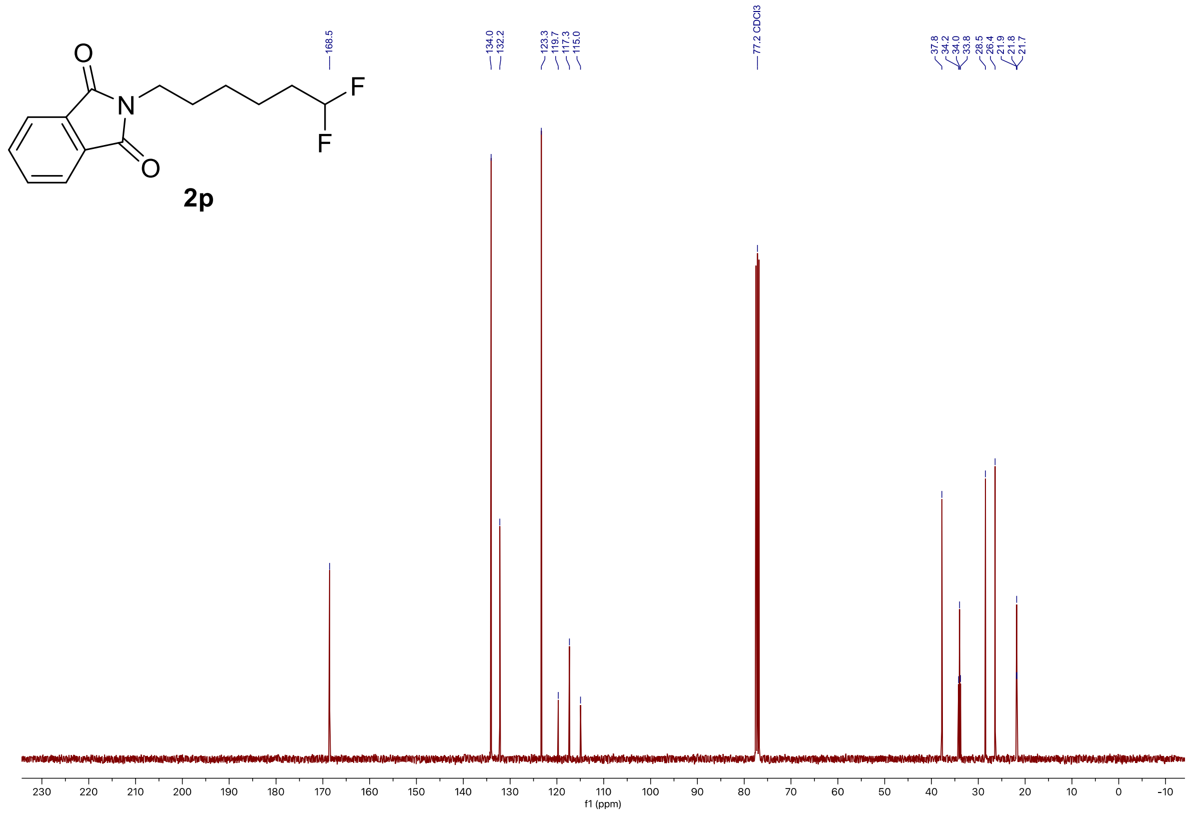

^{1}H NMR (400 MHz, CDCl$_3$)

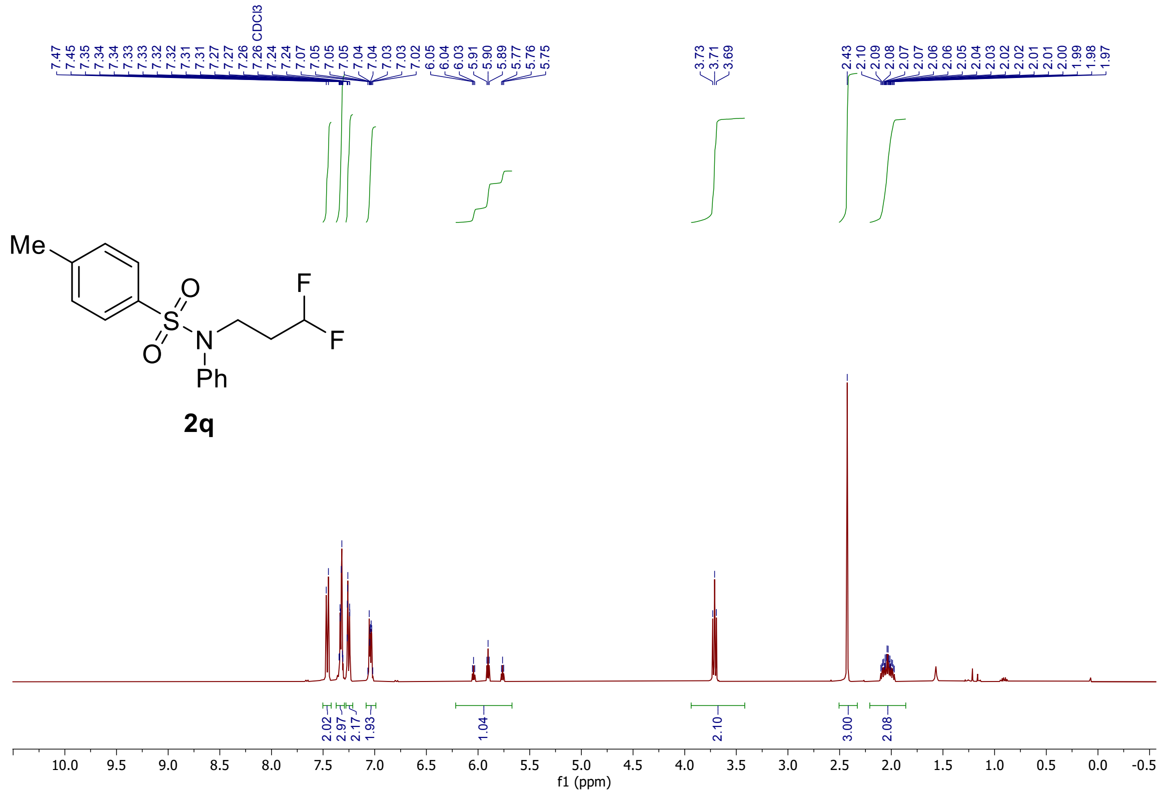

^{19}F NMR (376 MHz, CDCl$_3$)

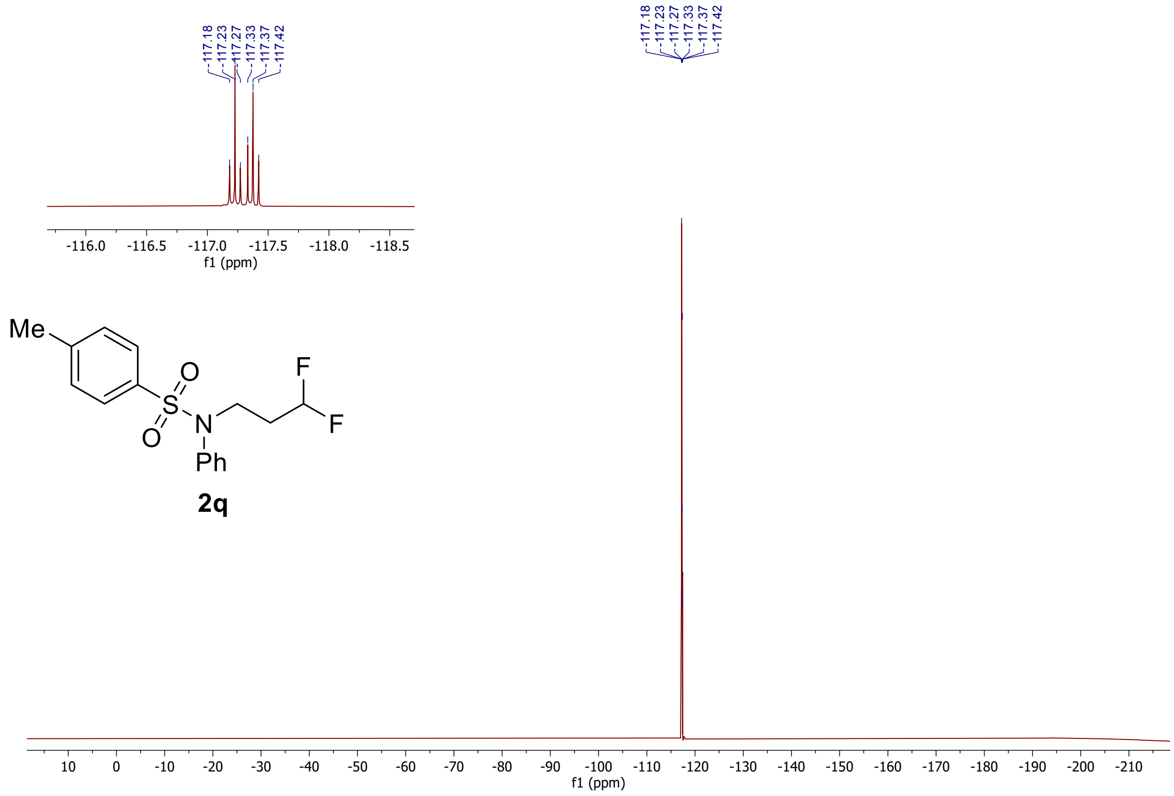

2q

^{13}C NMR (101 MHz, CDCl$_3$)

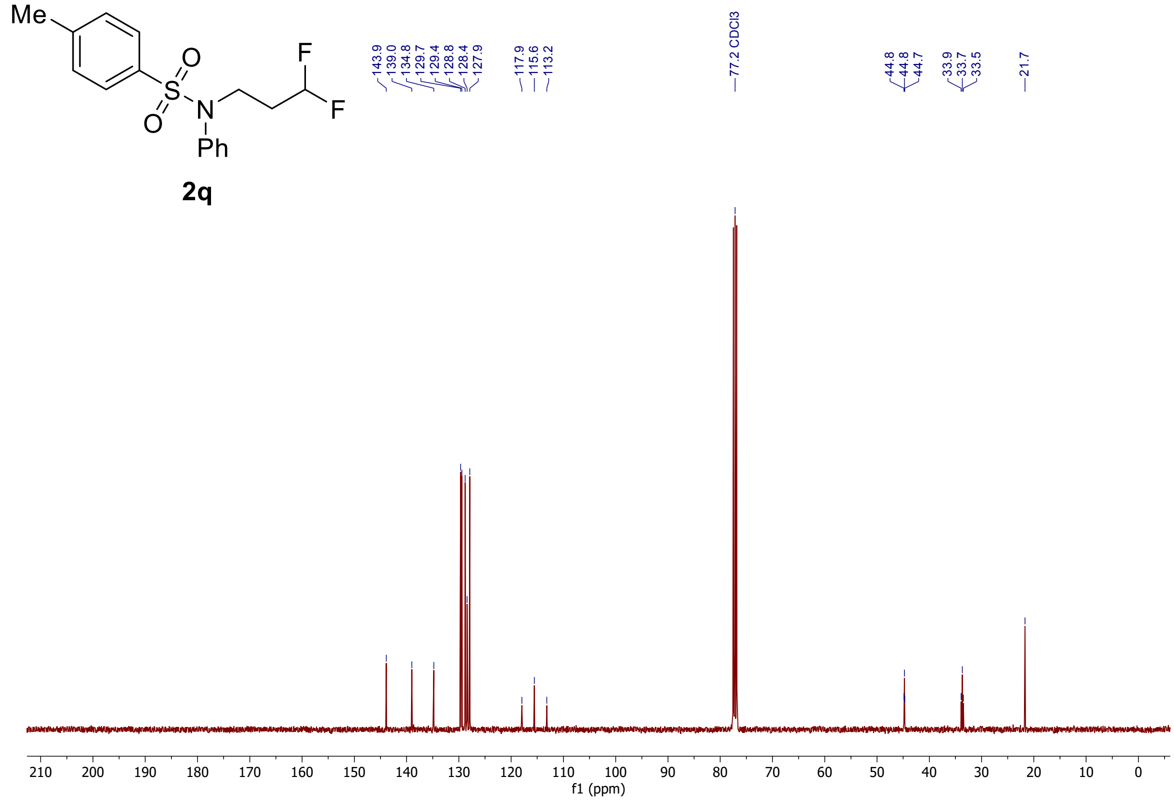

2q

^{1}H NMR (400 MHz, CDCl$_3$)

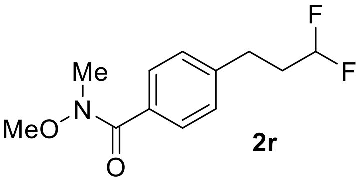

2r

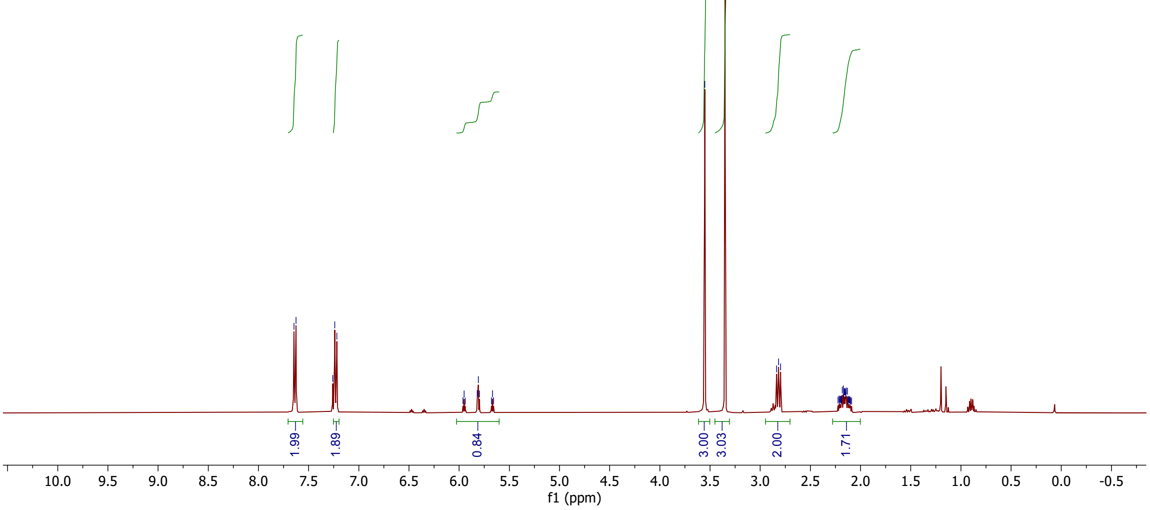

^{19}F NMR (376 MHz, CDCl$_3$)

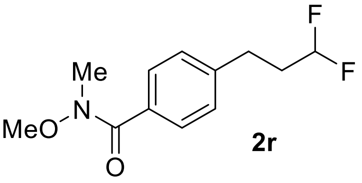

2r

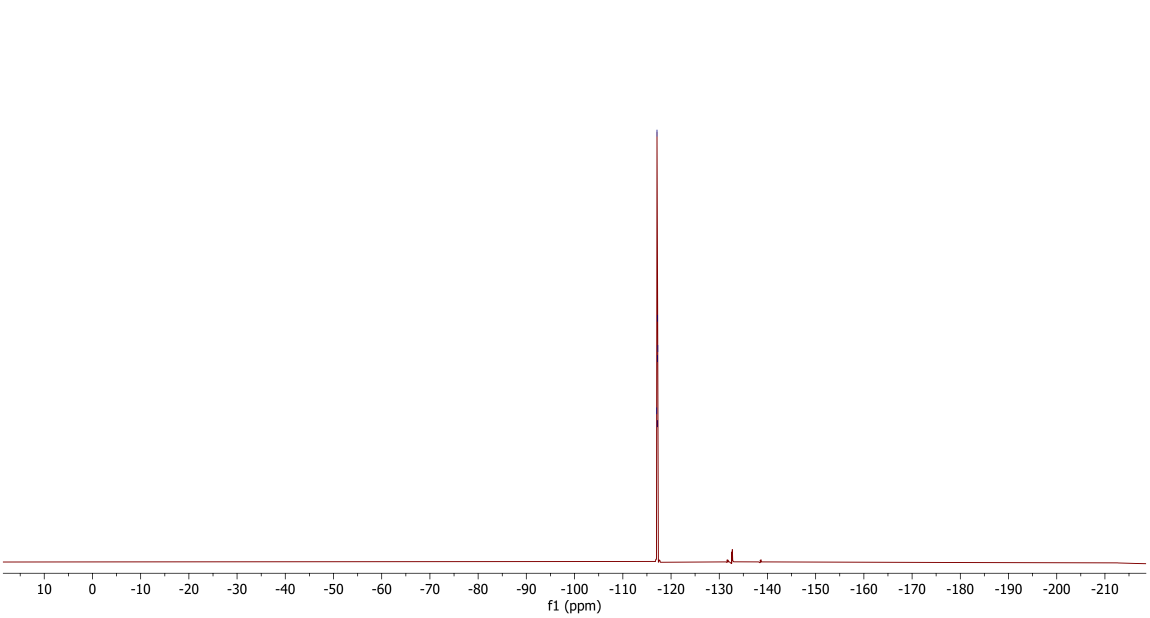

^{13}C NMR (101 MHz, $CDCl_3$)

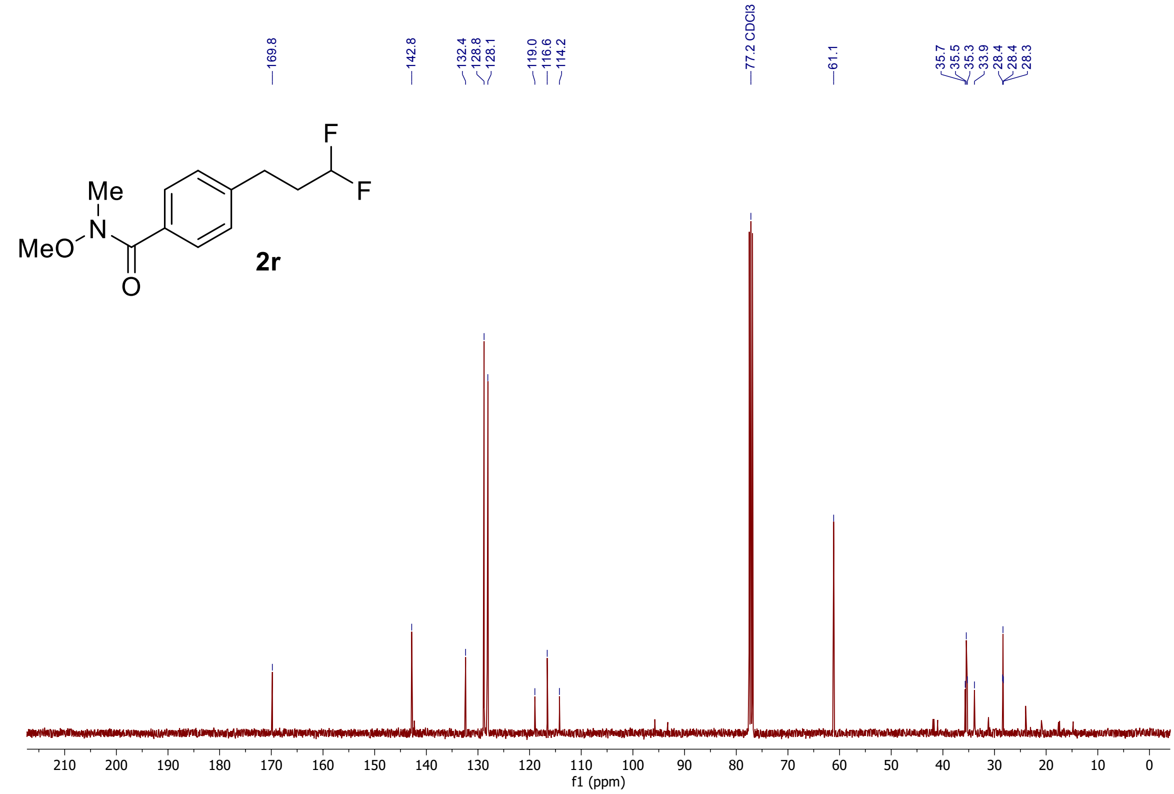

^{1}H NMR (400 MHz, $CDCl_3$)

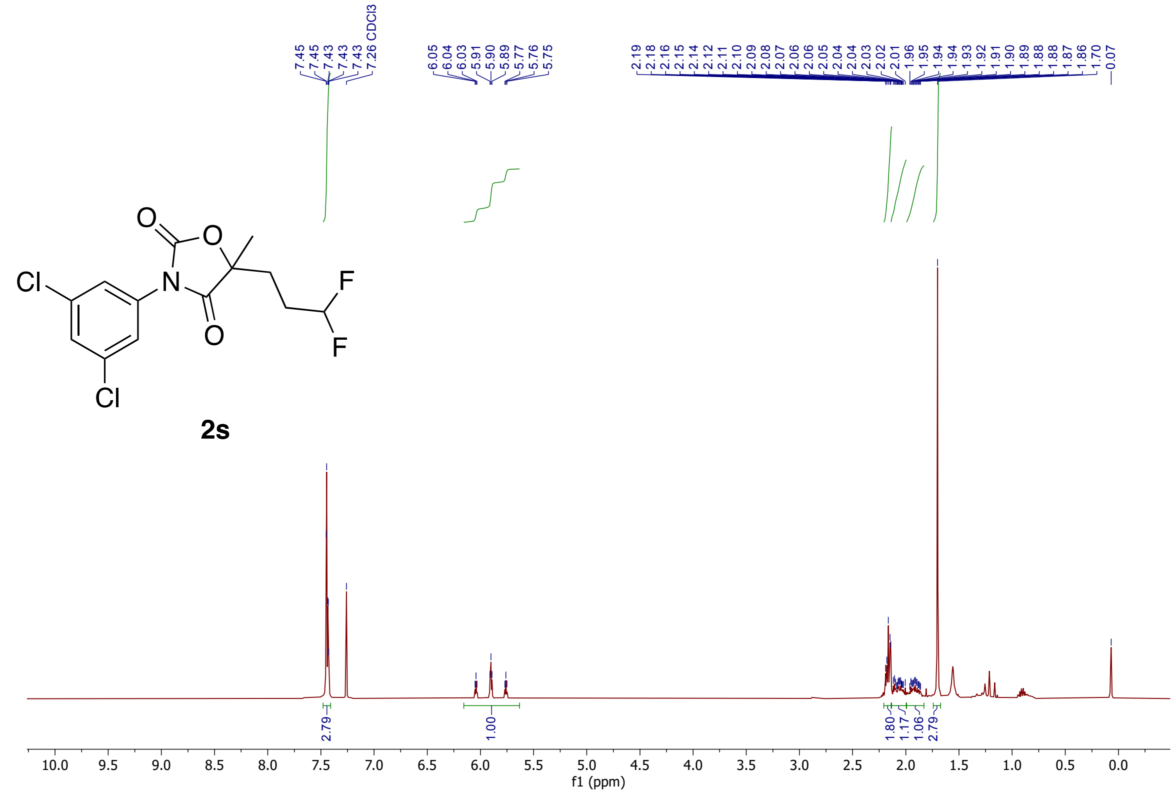

^{19}F NMR (376 MHz, CDCl$_3$)

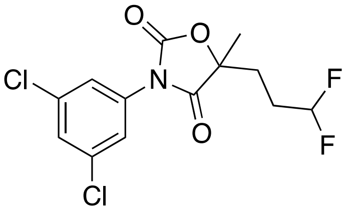

2s

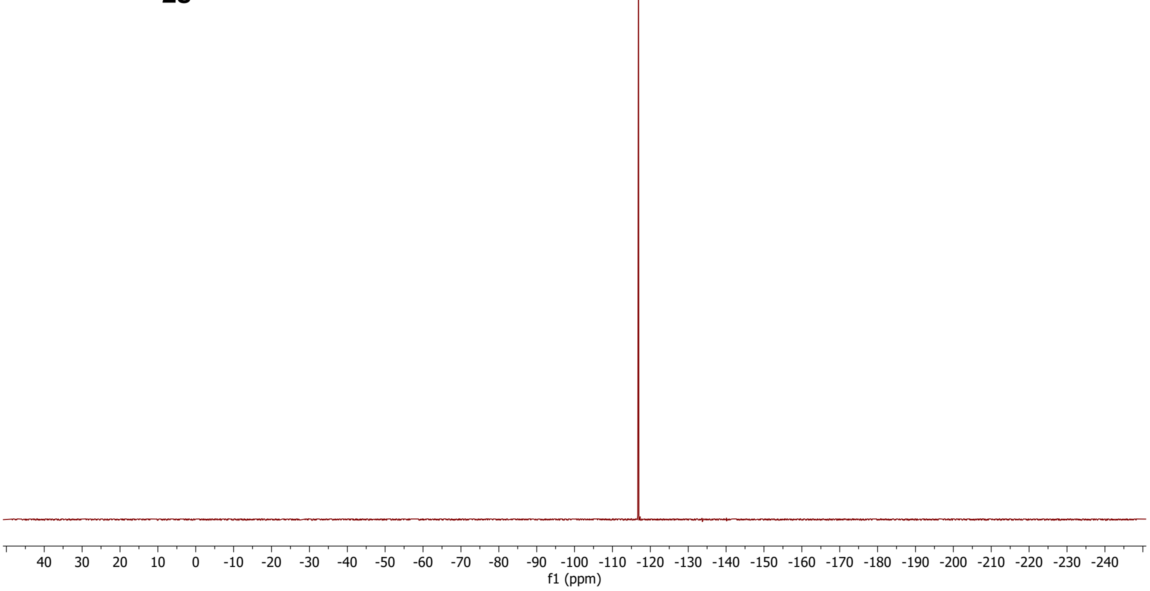

^{13}C NMR (126 MHz, CDCl$_3$)

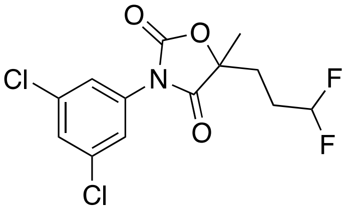

2s

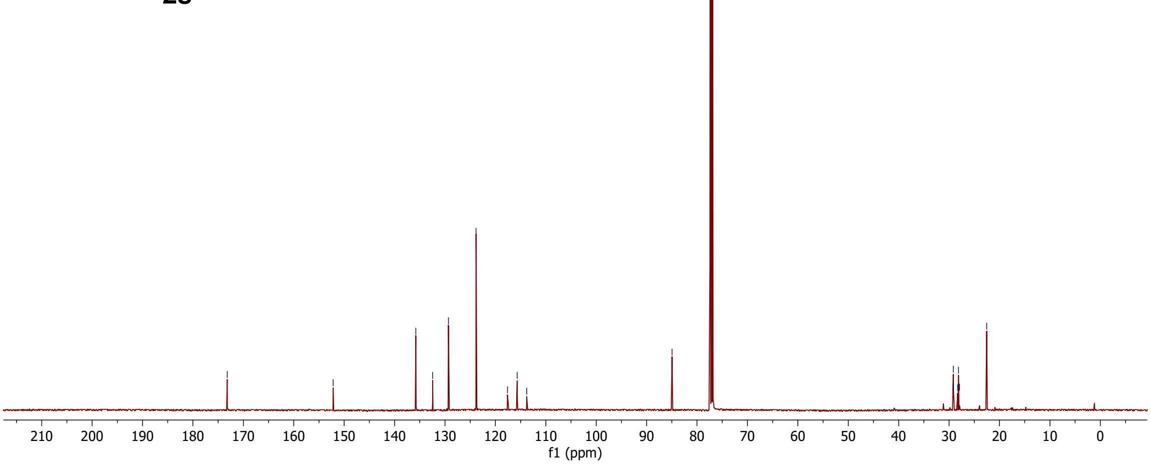

^{1}H NMR (400 MHz, $CDCl_3$)

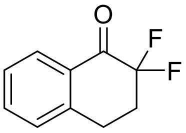

2t

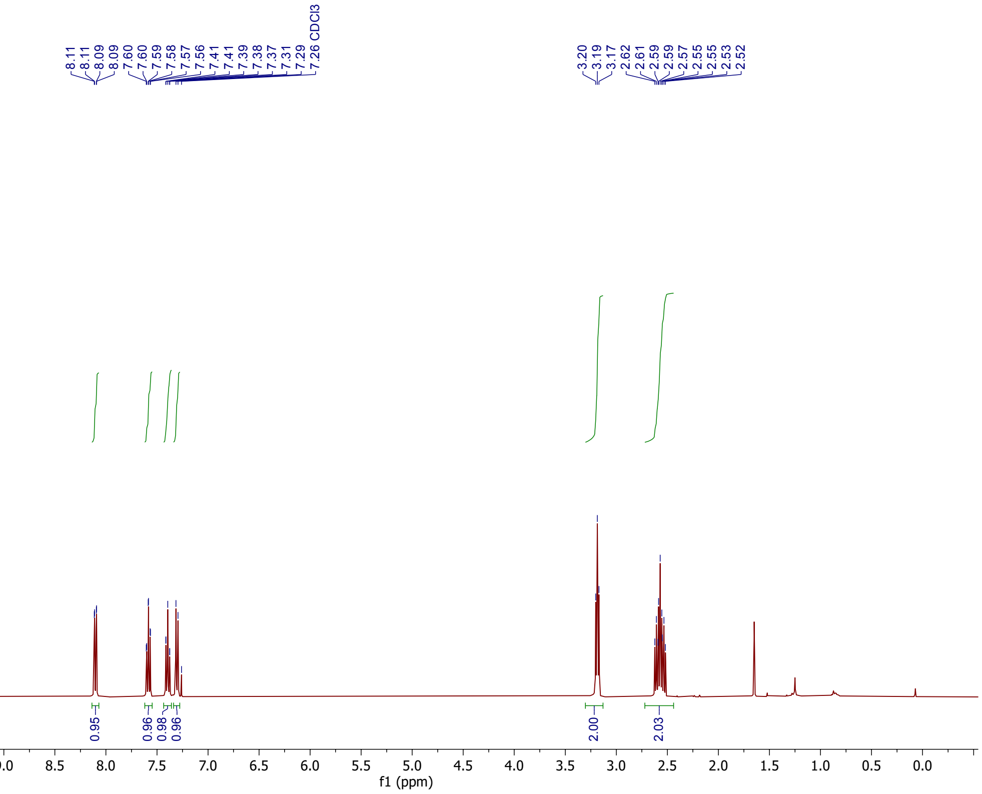

^{19}F NMR (376 MHz, $CDCl_3$)

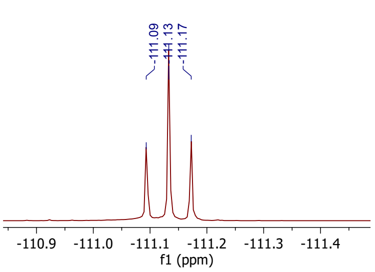

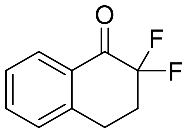

2t

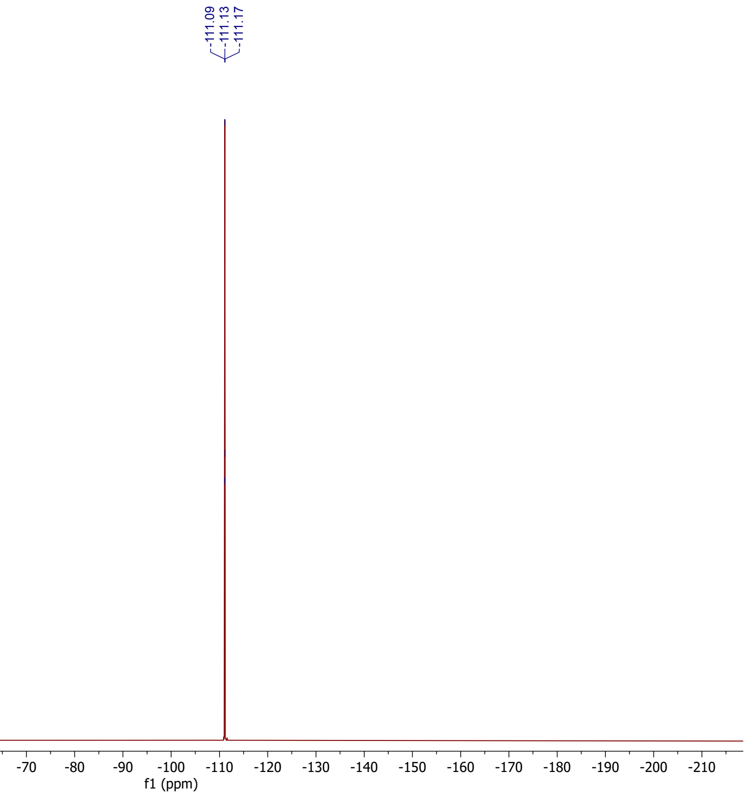

^{13}C NMR (101 MHz, $CDCl_3$)

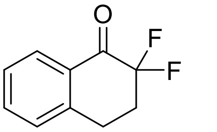

2t

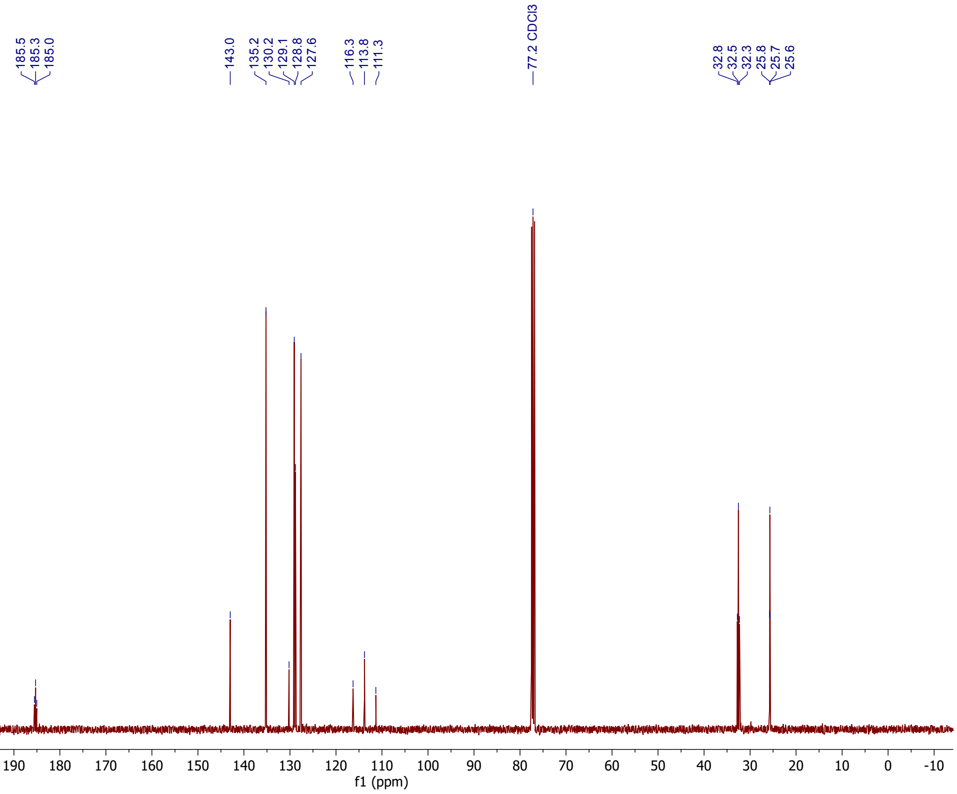

^{1}H NMR (400 MHz, $CDCl_3$)

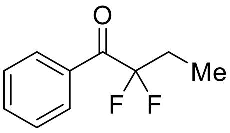

2u

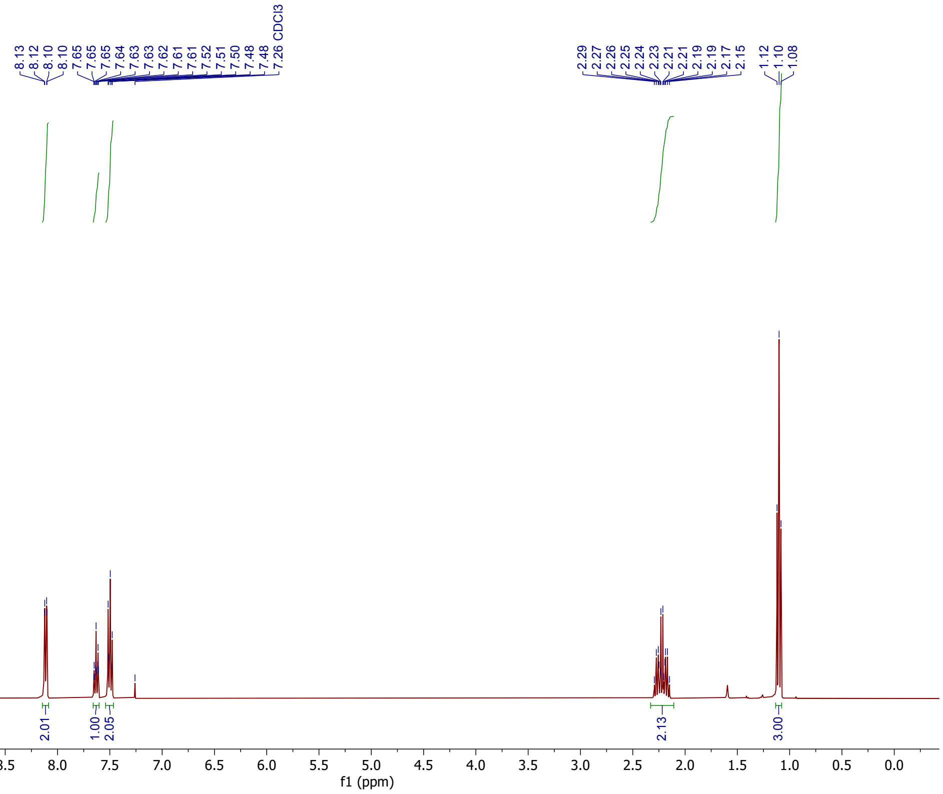

^{19}F NMR (376 MHz, $CDCl_3$)

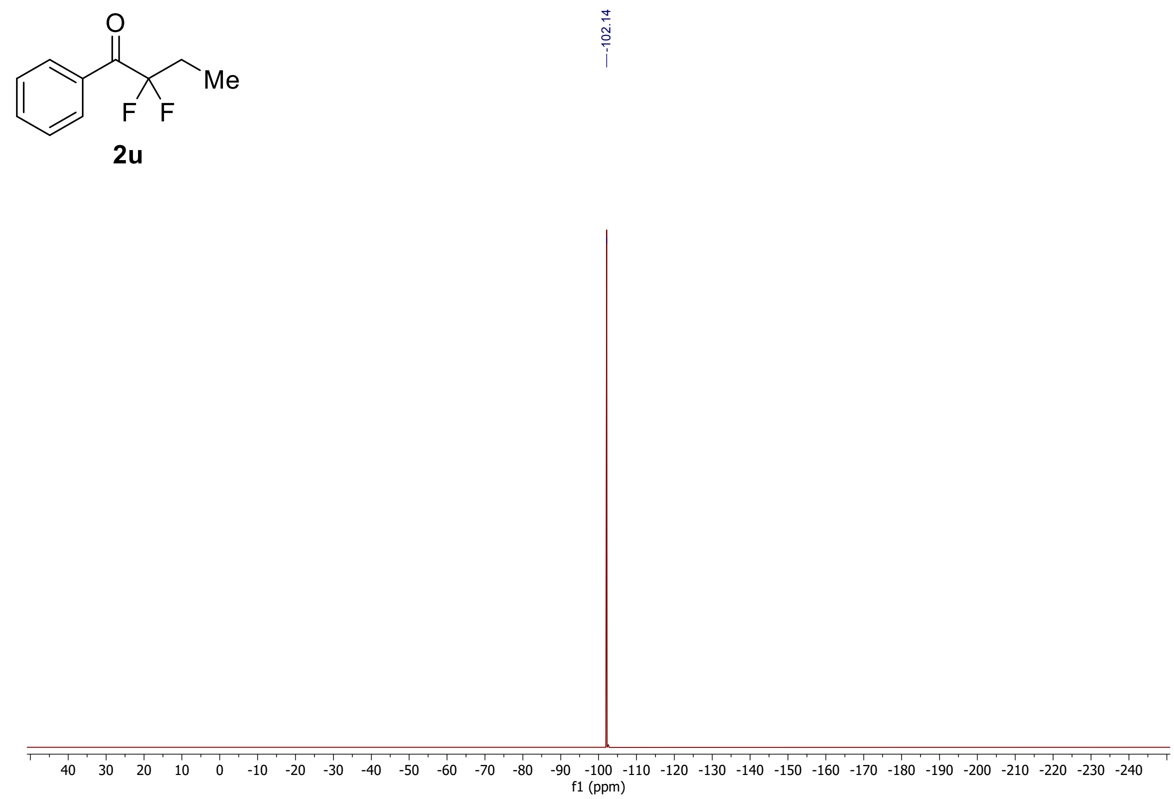

^{13}C NMR (101 MHz, $CDCl_3$)

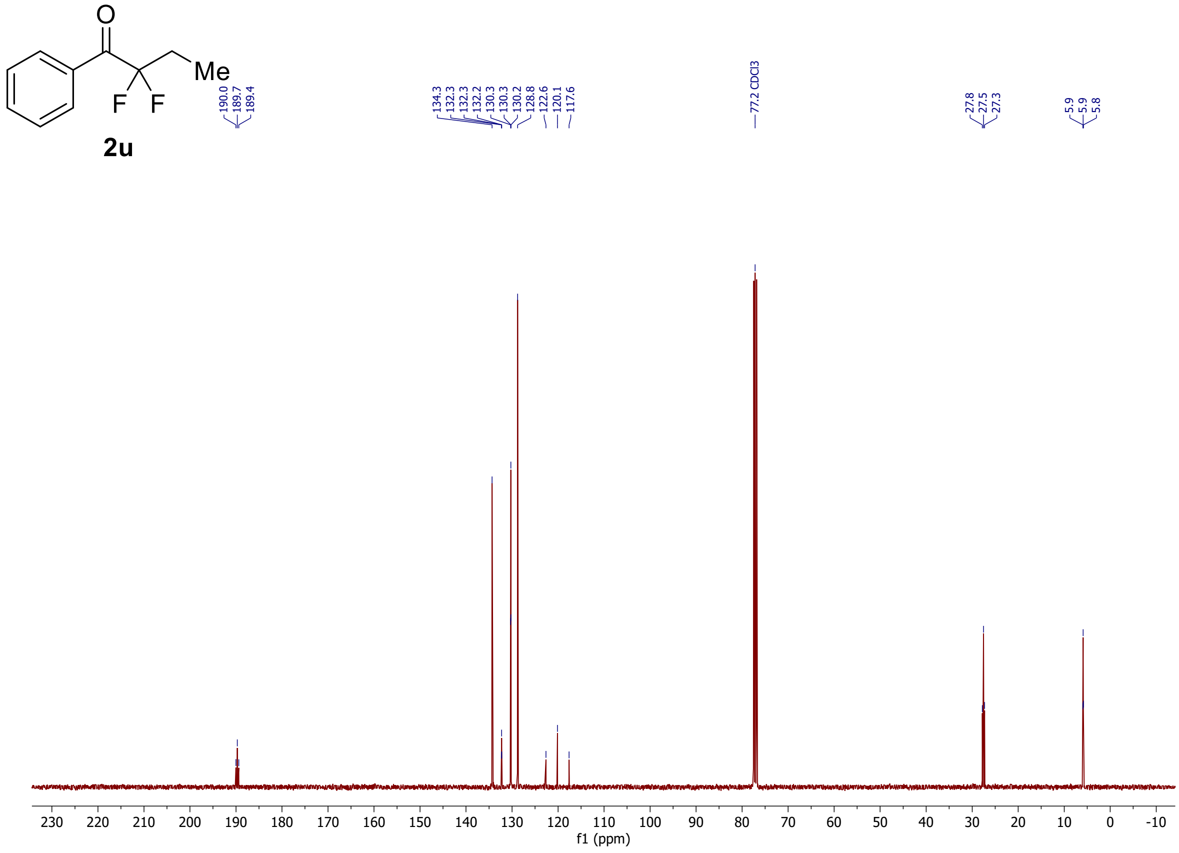

^1H NMR (400 MHz, $CDCl_3$)

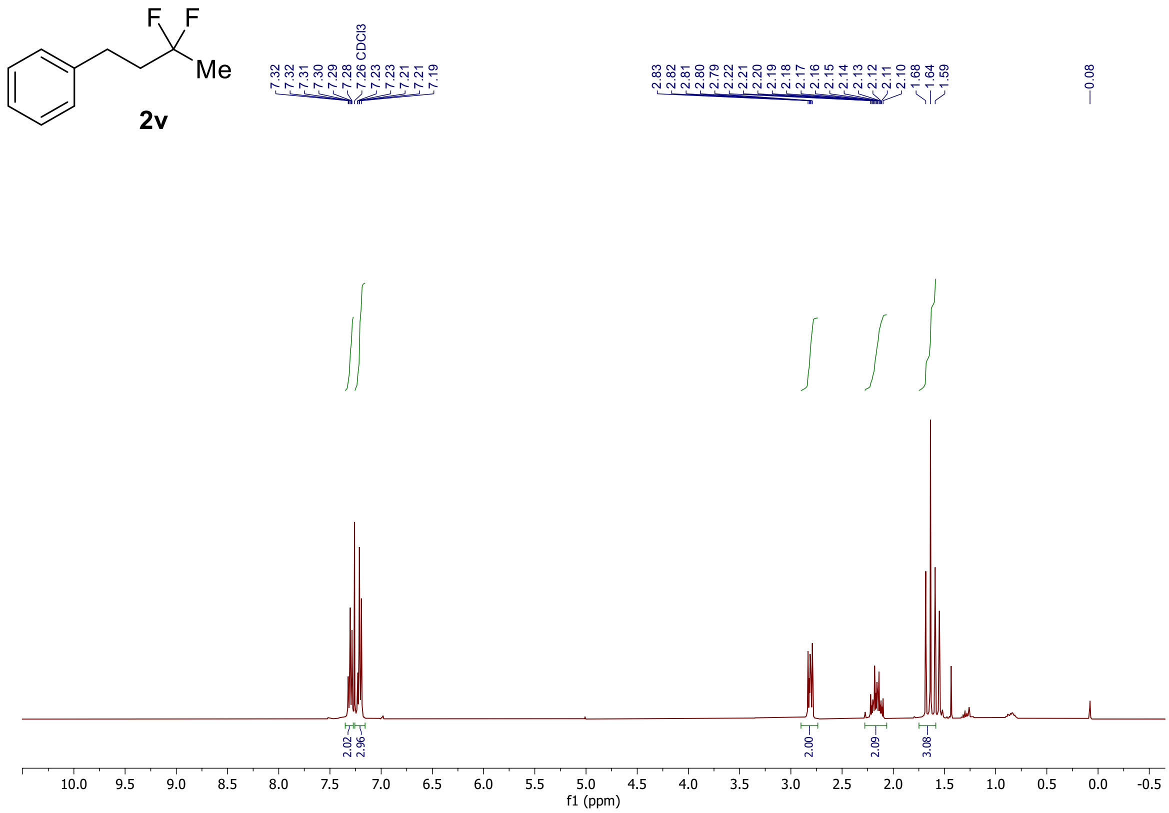

^{19}F NMR (376 MHz, $CDCl_3$)

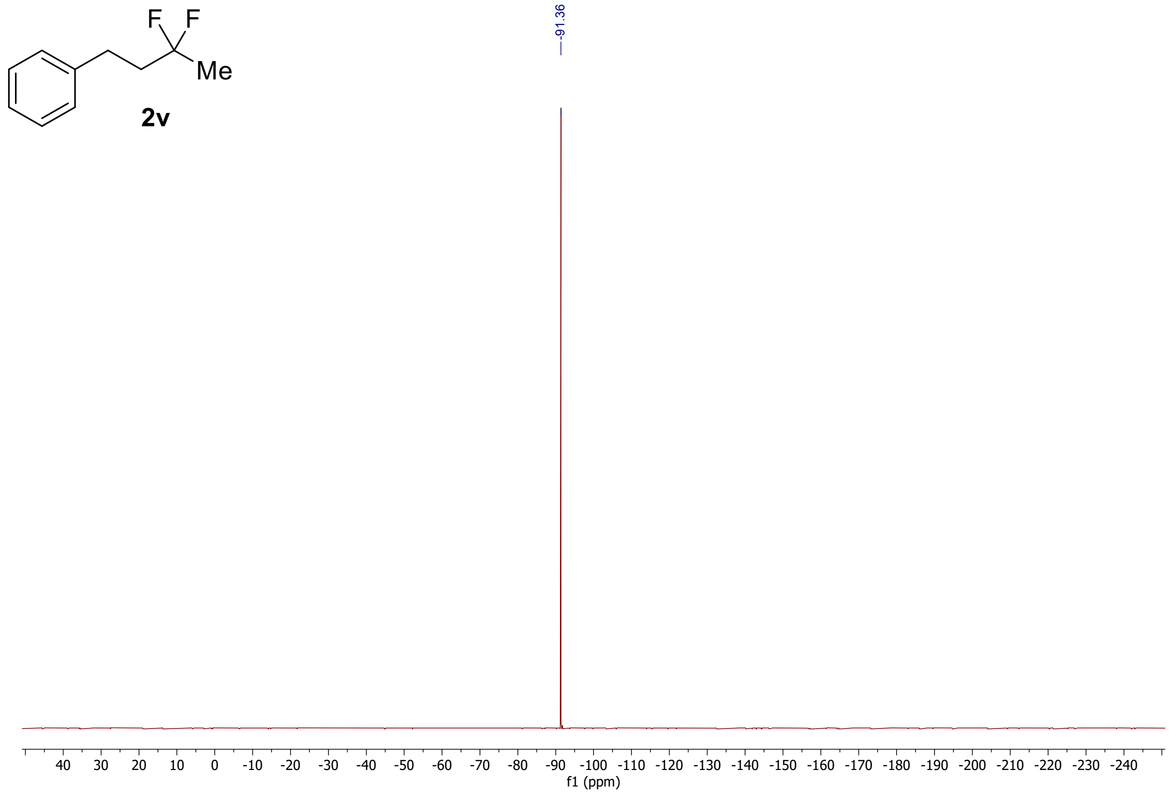

^{13}C NMR (101 MHz, CDCl$_3$)

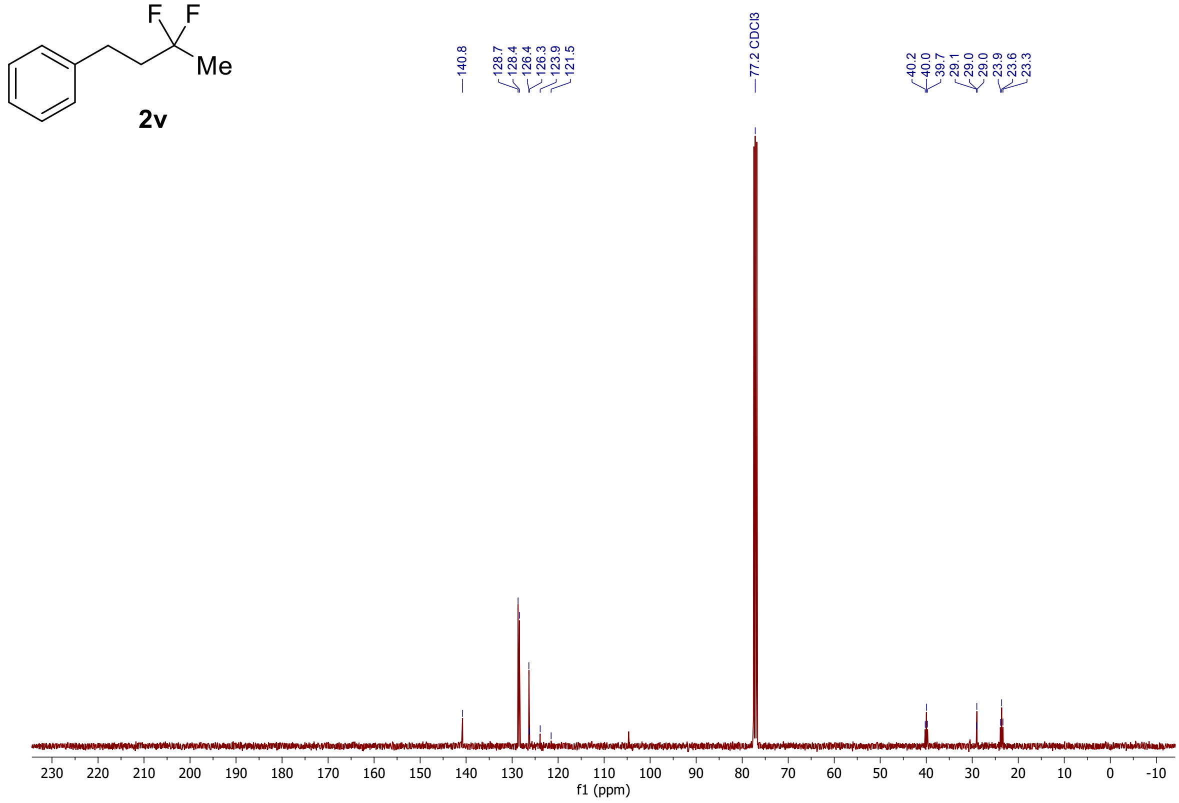

^{1}H NMR (400 MHz, CDCl$_3$)

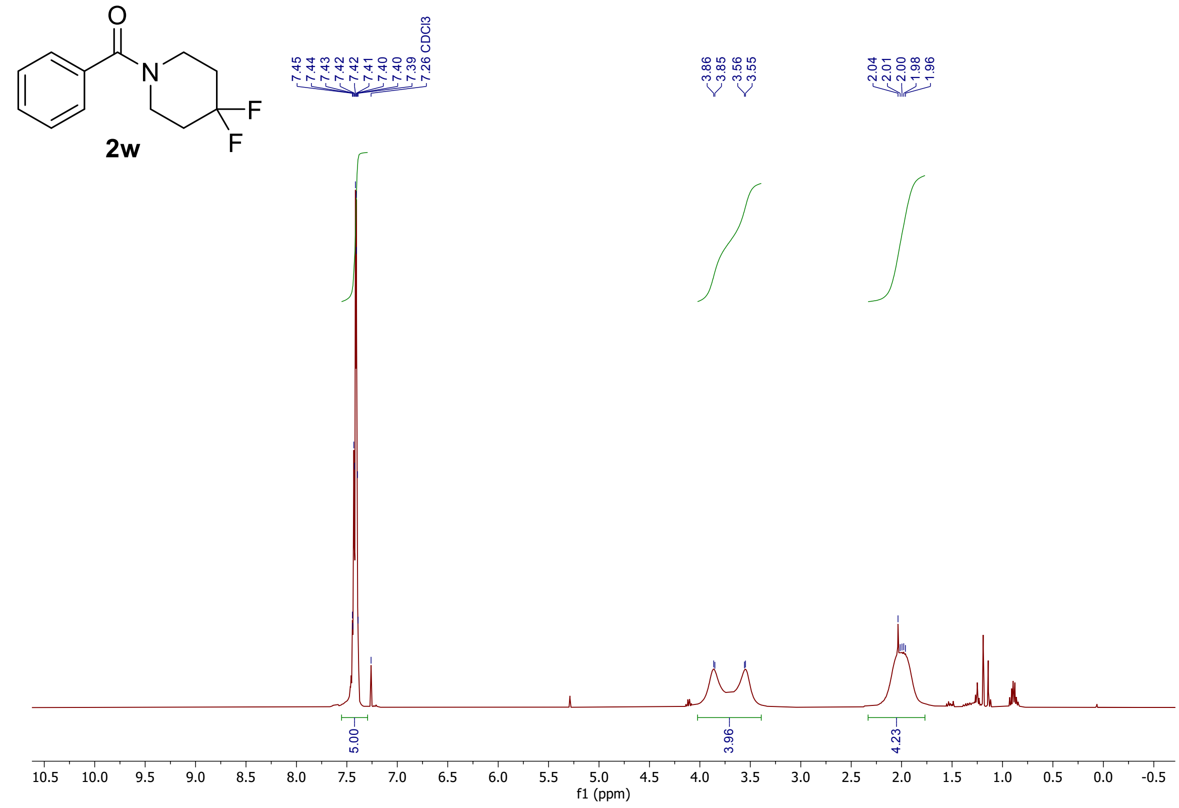

^{19}F NMR (376 MHz, $CDCl_3$)

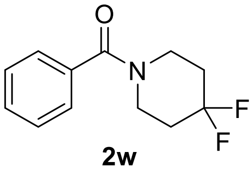

2w

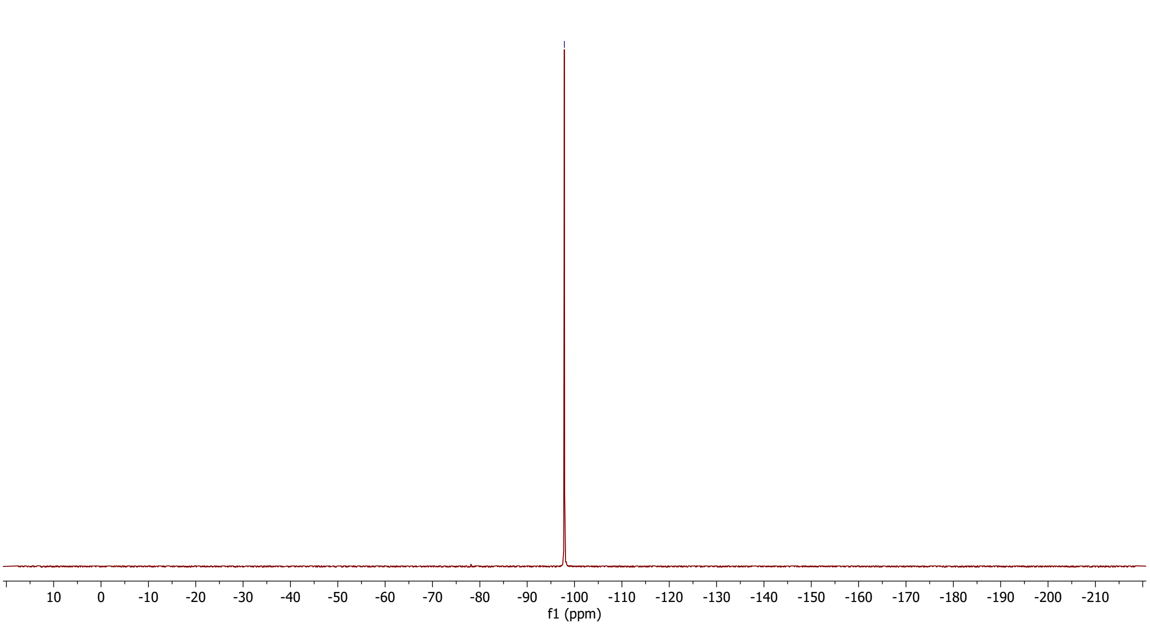

^{13}C NMR (101 MHz, $CDCl_3$)

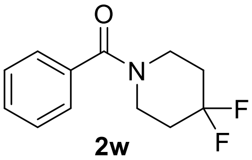

2w

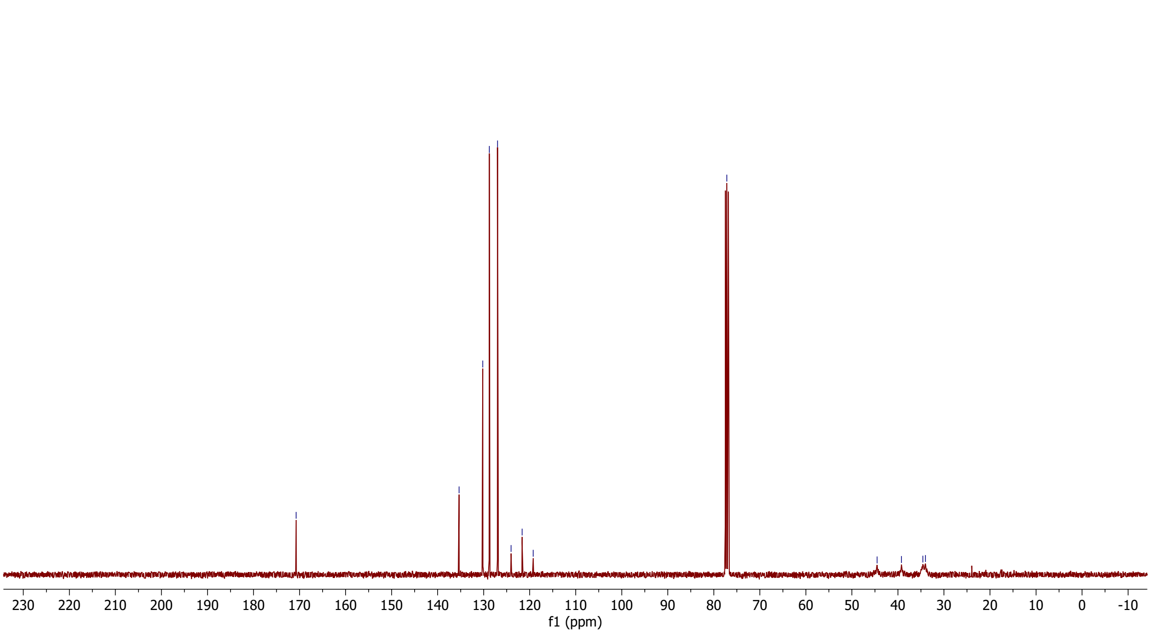

^{1}H NMR (400 MHz, $CDCl_3$)

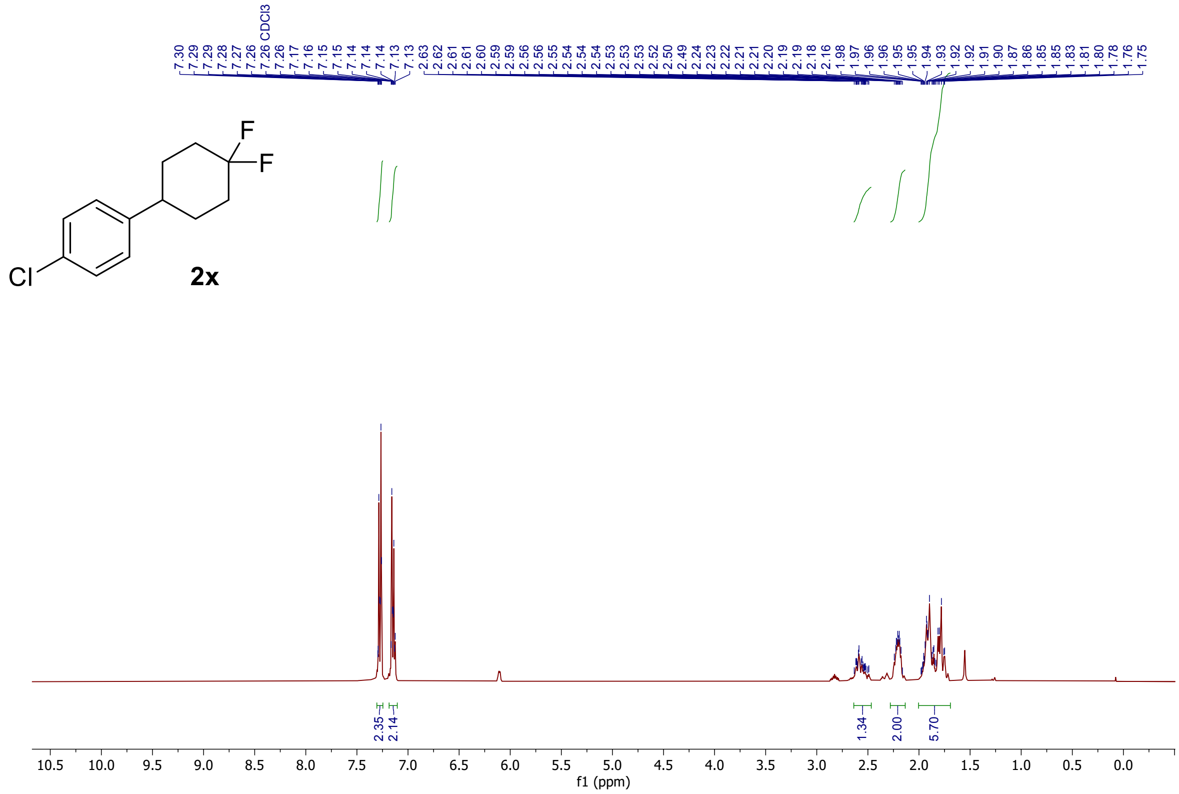

^{19}F NMR (376 MHz, $CDCl_3$)

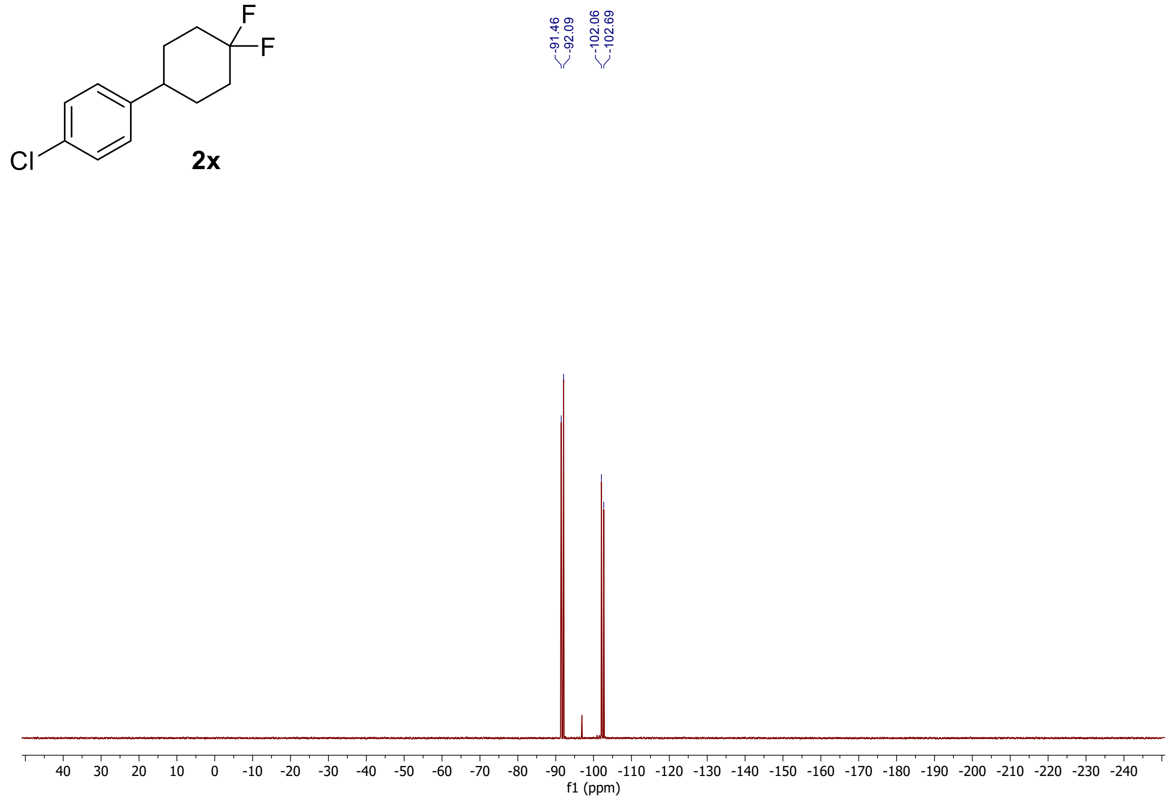

^{1}H NMR (400 MHz, $CDCl_3$)

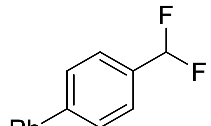

2y

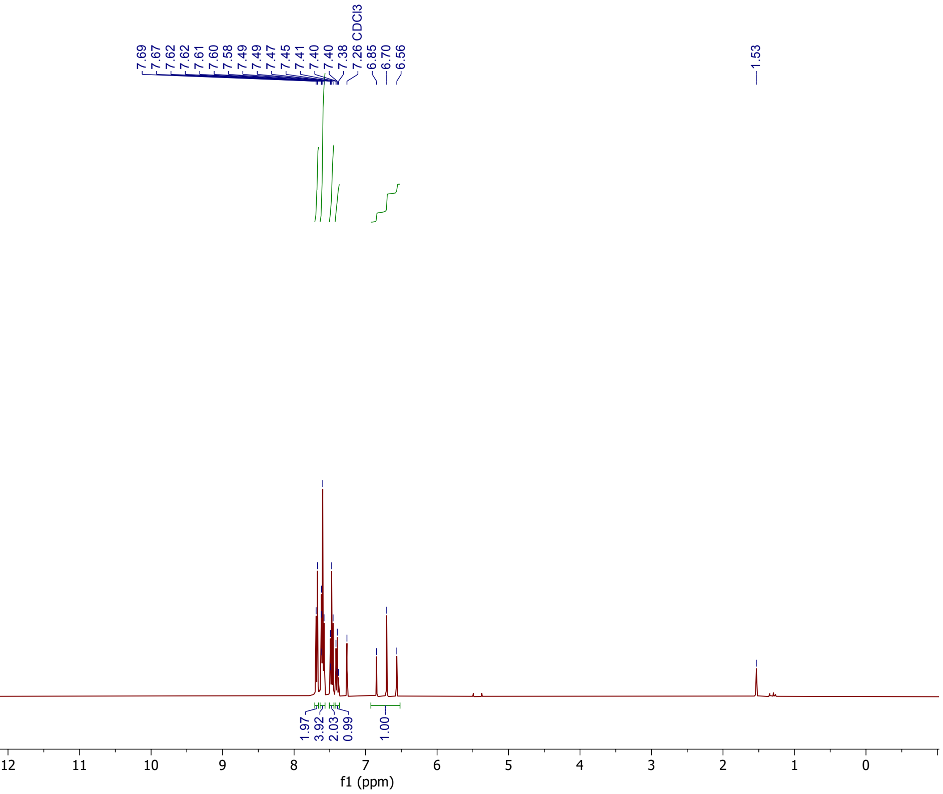

^{19}F NMR (376 MHz, $CDCl_3$)

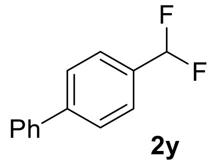

2y

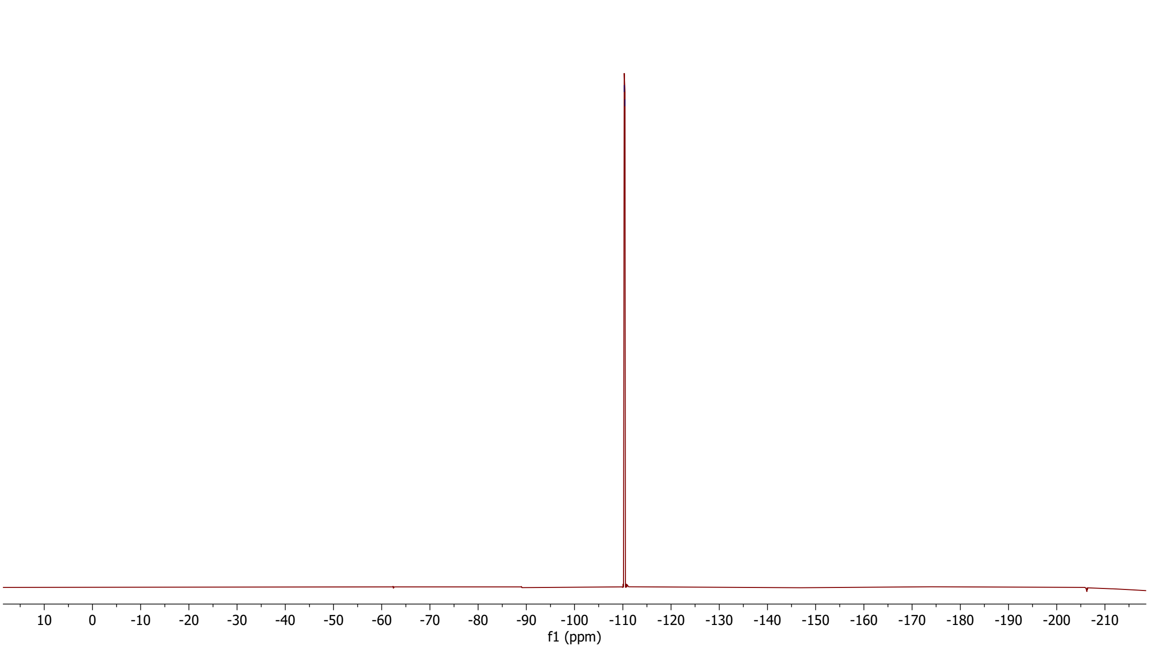

^{13}C NMR (101 MHz, $CDCl_3$)

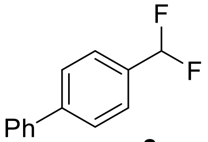

2y

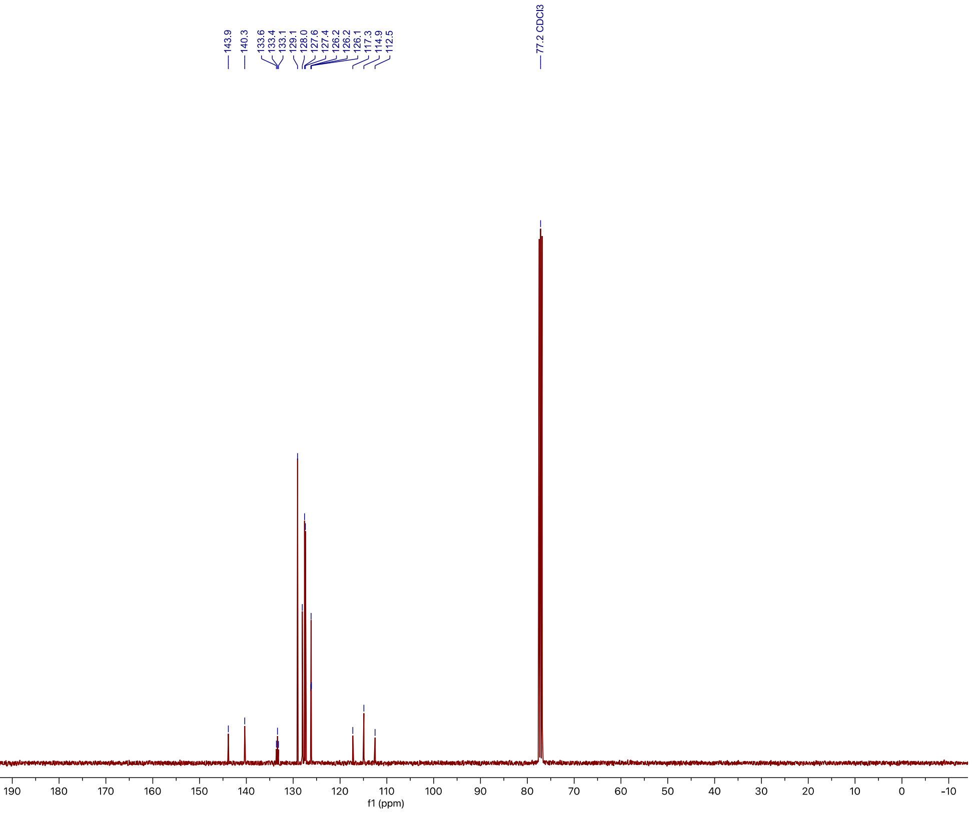

^{1}H NMR (400 MHz, $CDCl_3$)

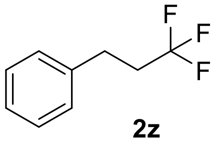

2z

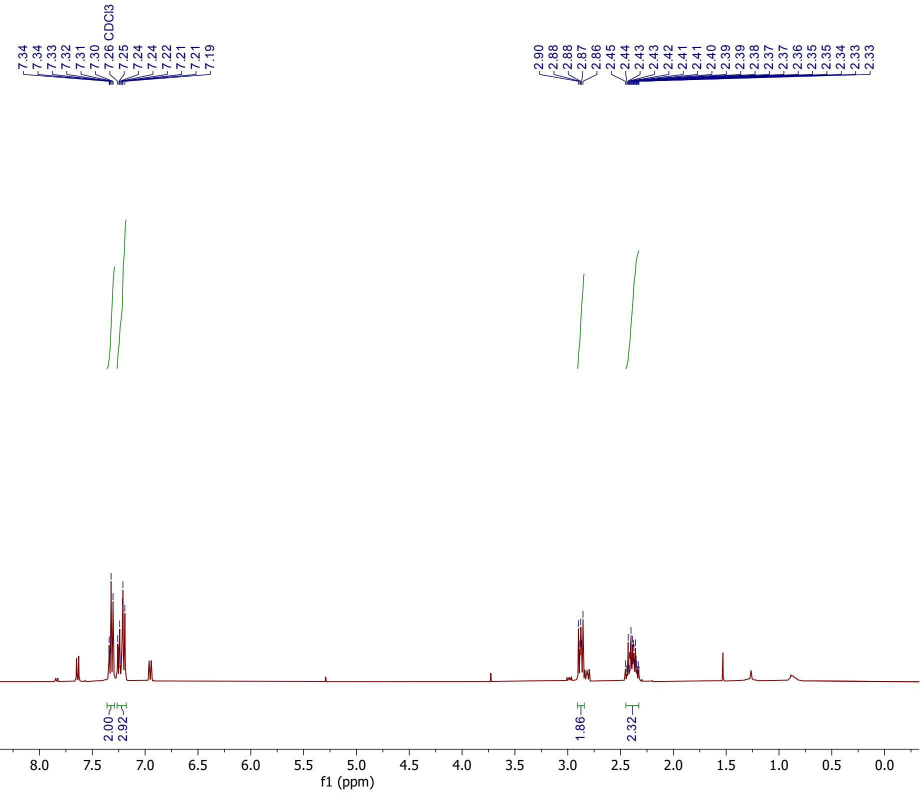

^{19}F NMR (376 MHz, $CDCl_3$)

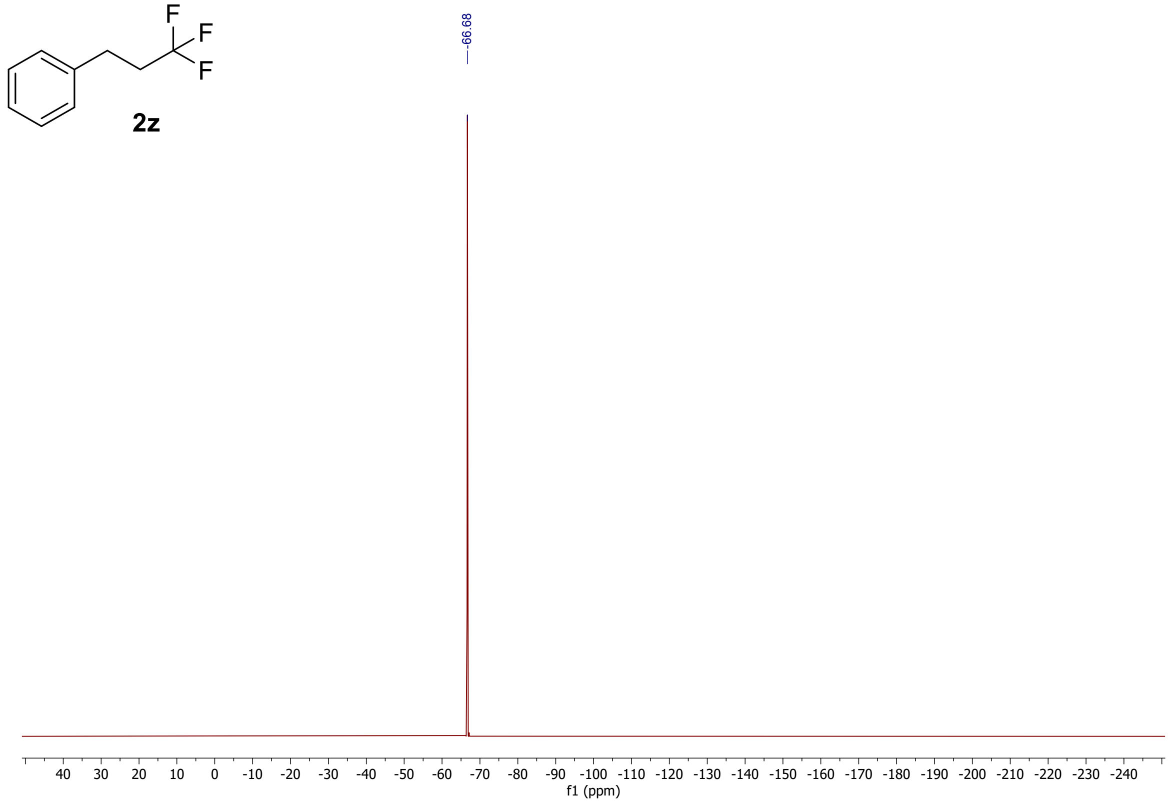

^{1}H NMR (400 MHz, $CDCl_3$)

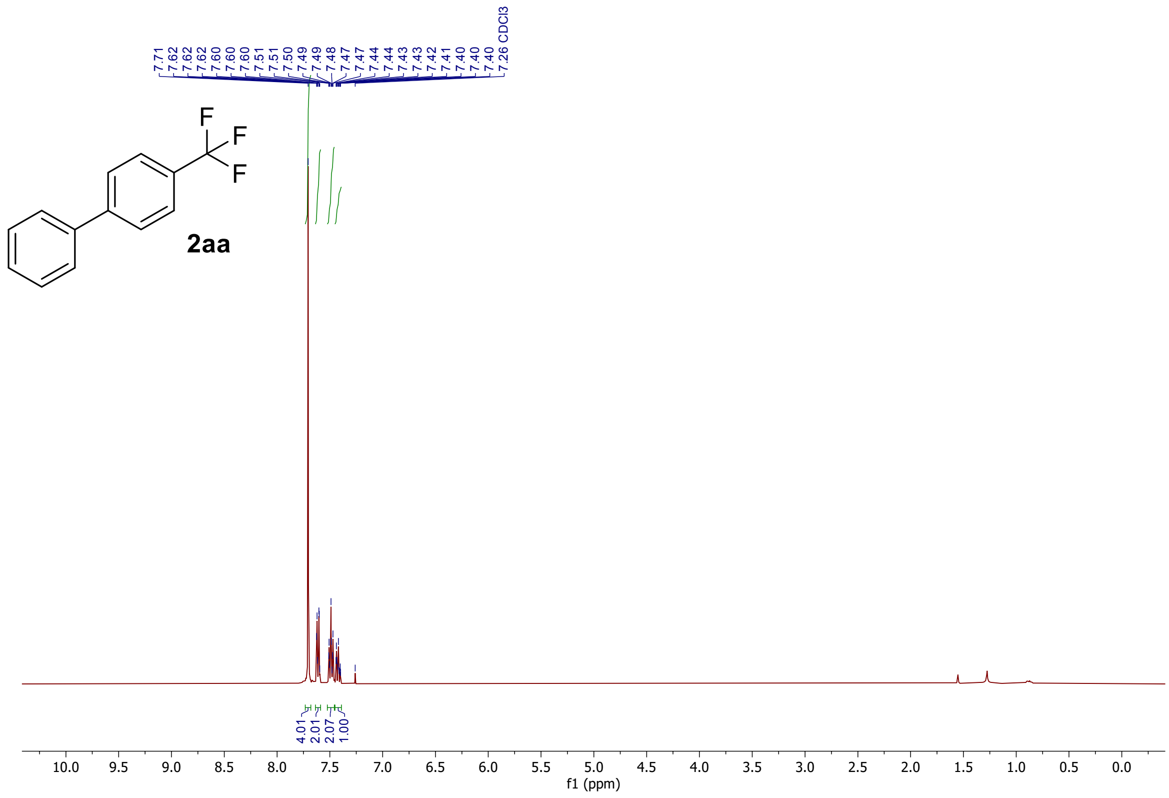

^{19}F NMR (376 MHz, $CDCl_3$)

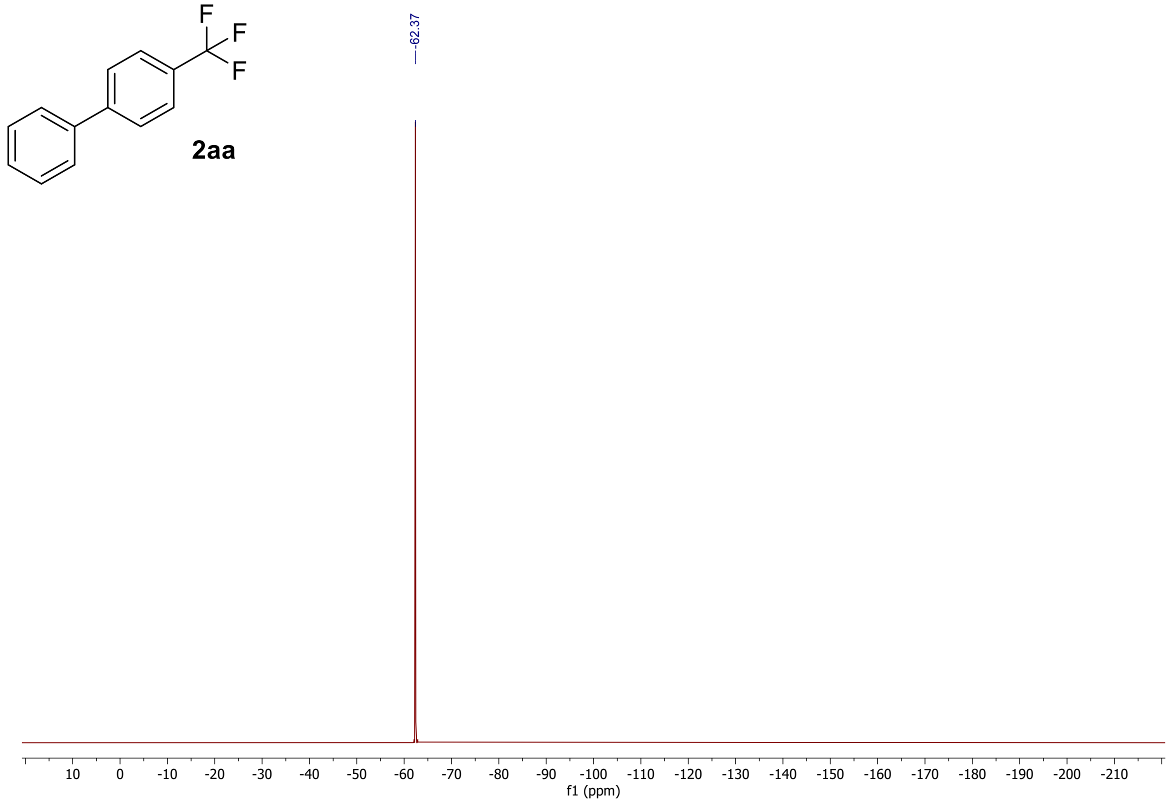

^{1}H NMR (400 MHz, $CDCl_3$)

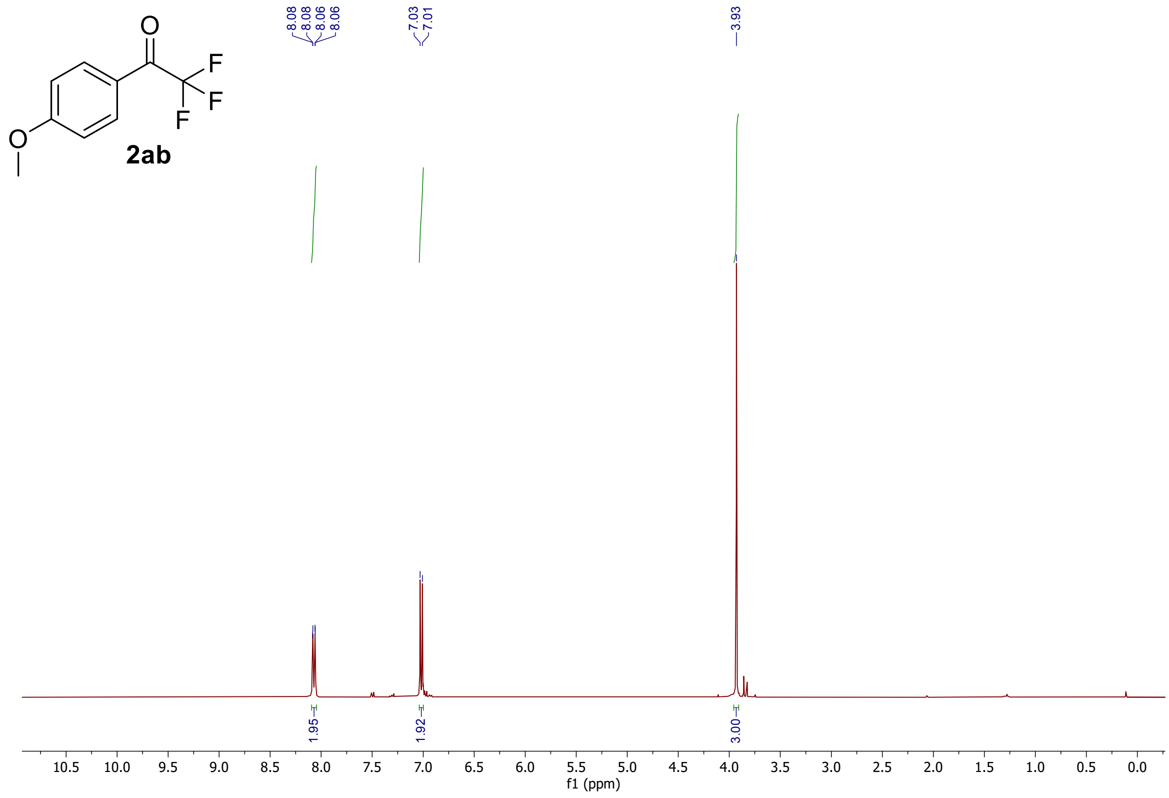

^{19}F NMR (376 MHz, $CDCl_3$)

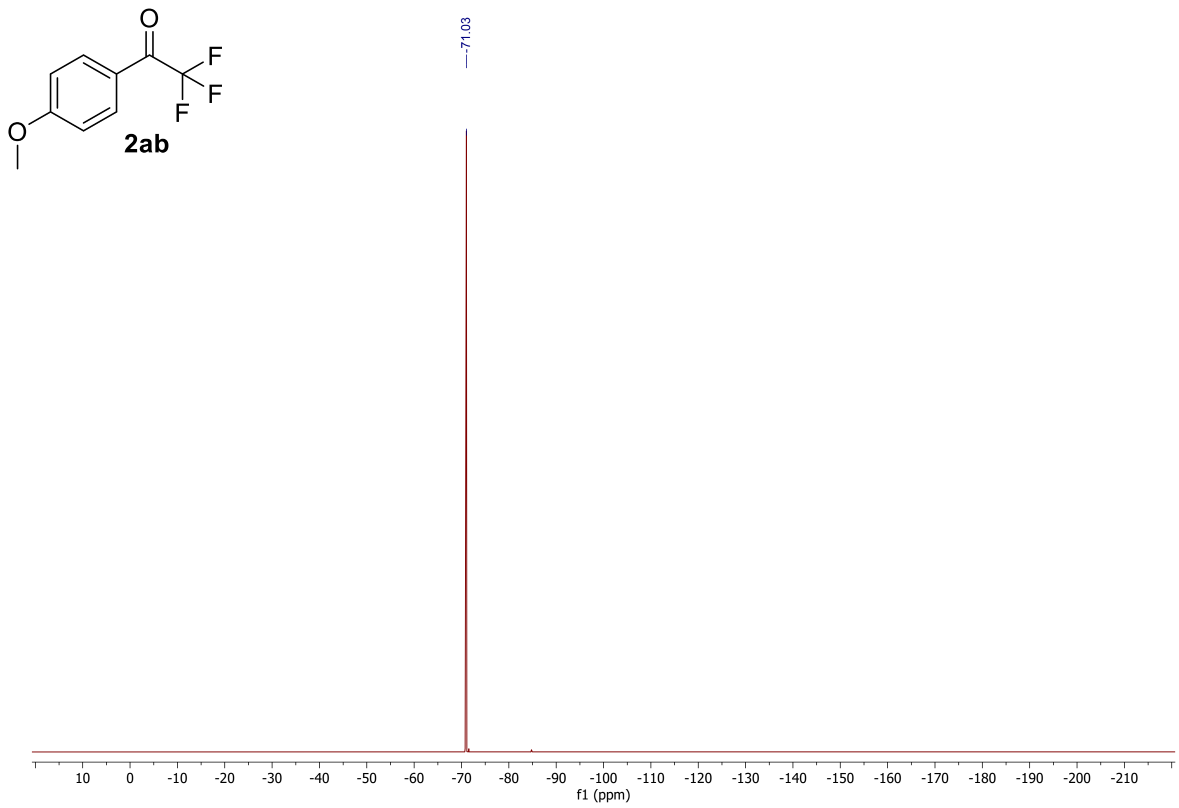

^{13}C NMR (101 MHz, $CDCl_3$)

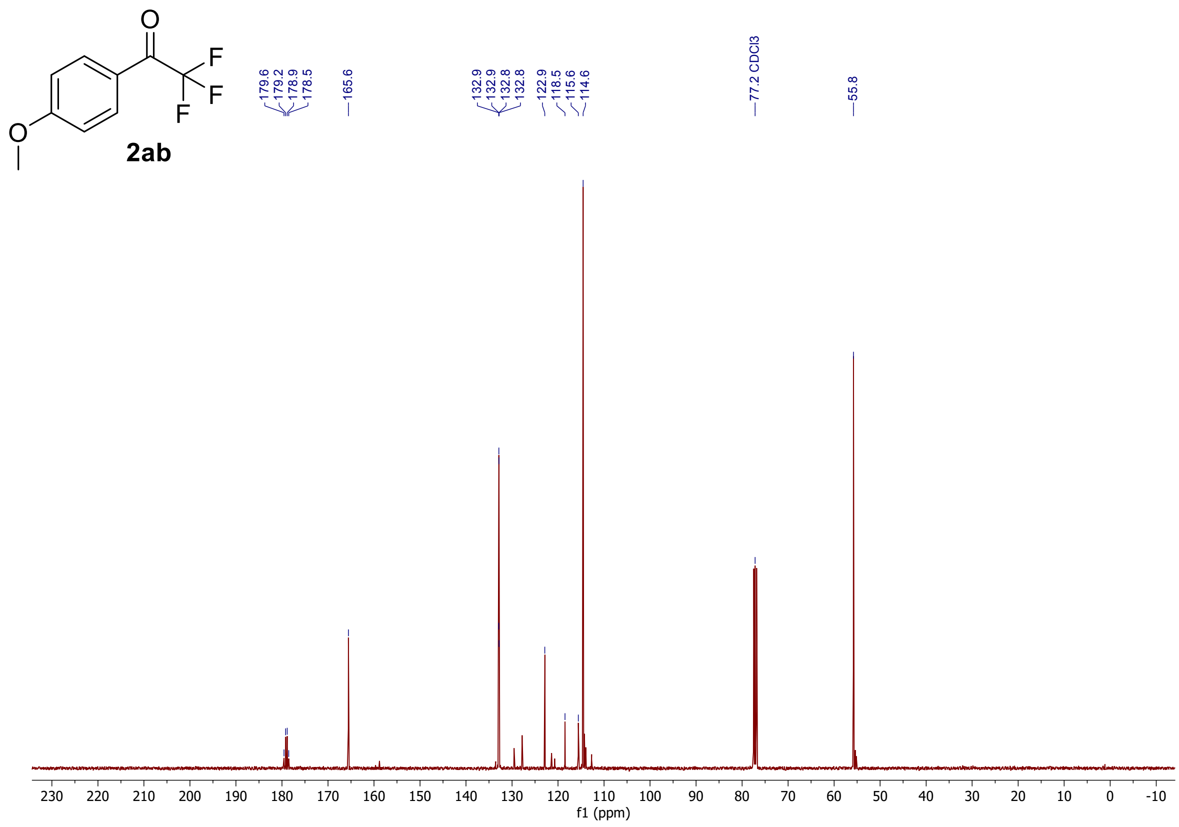

Supplement: Supplementary file 1 — ol5c00187_si_001.pdf [file ol5c00187_si_001.pdf]
